# Genetically proxied circulating PD-1/PD-L1 levels and broadly defined myocarditis: A bidirectional Mendelian randomization study with exploratory lipidomic analyses

**DOI:** 10.1097/MD.0000000000050475

**Published:** 2026-08-28

**Authors:** Fengyi Liu, Qian Yang

**Affiliations:** a Department of Cardiology, The Second Hospital of Dalian Medical University, Dalian, Liaoning, China.

**Keywords:** Mendelian randomization, myocarditis, plasma lipidomics, programmed cell death protein 1, programmed death-ligand 1

## Abstract

Myocarditis is an inflammatory myocardial disease with potentially severe outcomes. Programmed cell death protein 1 (PD-1) and programmed death-ligand 1 (PD-L1) regulate immune tolerance, but the association between lifelong genetically proxied circulating PD-1/PD-L1 levels and broadly defined myocarditis remains uncertain. We investigated these associations and explored related plasma lipid species. We conducted bidirectional 2-sample Mendelian randomization using proteomic genome-wide association data from the UK Biobank Pharma Proteomics Project and INTERVAL. FinnGen Release 10 was the primary broadly defined myocarditis outcome, and an independent myocarditis genome-wide association study (GCST90018882) provided outcome-level validation. Complementary estimators, heterogeneity and pleiotropy diagnostics, influence analyses, MR-RAPS, and supportive meta-analyses were performed. Associations with 179 plasma lipid species were examined in exploratory analyses. Higher genetically proxied circulating PD-L1 was inversely associated with broadly defined myocarditis in UKB-PPP (odds ratio [OR] 0.834, 95% confidence interval [CI] 0.698–0.995; *P* = .0441), and the independent INTERVAL analysis yielded a concordant inverse estimate (OR 0.619, 95% CI: 0.434–0.883; *P* = .0083); no clear association was observed for PD-1. The MR-RAPS estimate retained the inverse direction; estimates against the independent broadly defined myocarditis dataset were also inverse, and supportive meta-analyses across protein and outcome sources yielded inverse pooled estimates. Reverse MR did not support effects of broadly defined myocarditis liability on circulating PD-1 or PD-L1. Exploratory lipid analyses identified nominal associations requiring confirmation. Higher genetically proxied circulating PD-L1 may be associated with a lower risk of broadly defined myocarditis, supporting further investigation of PD-L1-related immune regulation. These findings do not directly estimate the effects of pharmacologic PD-1/PD-L1 blockade. The lipid findings are hypothesis-generating.

## 1. Introduction

Myocarditis is an inflammatory myocardial disease caused by infectious, autoimmune or other immune-mediated, toxic, drug-related, vaccine-associated, and idiopathic processes.^[1-6]^ This heterogeneous clinical entity includes acute, fulminant, chronic active, and recurrent forms, with presentations ranging from subclinical myocardial injury or chest pain to malignant arrhythmia, heart failure, cardiogenic shock, sudden cardiac death, and inflammatory dilated cardiomyopathy. Because etiologies and phenotypes are heterogeneous and tissue diagnosis is not always available, broadly defined biobank phenotypes capture clinically relevant disease but may combine biologically distinct subtypes. Identifying immune-related determinants may therefore clarify shared disease mechanisms and help prioritize mechanistic investigation. Previous bidirectional MR research has examined COVID-19 as a potential causal factor for myocarditis or pericarditis.^[7]^

The PD-1/PD-L1 pathway restrains T-cell activation and contributes to peripheral immune tolerance.^[8]^ Experimental evidence supports a protective role for cardiac PD-L1 signaling against T-cell-mediated injury.^[9]^ In clinical oncology, antibodies that block PD-1 or PD-L1 can cause immune-related adverse events, including rare but potentially fatal myocarditis.^[10-14]^ These observations provide clinically relevant background for investigating PD-1/PD-L1 biology in myocarditis. Protein quantitative trait loci (pQTLs) can be used in MR to examine the consequences of genetically proxied differences in circulating protein levels.^[15-18]^ However, such genetic proxies are not equivalent to pharmacologic blockade: they reflect lifelong differences in circulating protein concentrations rather than the dose, timing, tissue distribution, or target occupancy of monoclonal-antibody therapy.

Lipid metabolism is closely linked to immune-cell function and myocardial homeostasis.^[19-22]^ Variation in specific plasma lipid species may therefore provide clues to metabolic pathways that connect immune regulation with myocardial injury. The lipid component of this study was considered exploratory. We conducted bidirectional 2-sample MR to investigate associations of genetically proxied circulating PD-1 and PD-L1 levels with broadly defined myocarditis. UKB-PPP served as the discovery protein source and INTERVAL as an independent protein-source validation dataset; an independent myocarditis GWAS (GCST90018882) provided outcome-level validation, followed by supportive meta-analyses. We also explored bidirectional relationships with 179 plasma lipid species.

## 2. Materials and methods

### 2.1. Study overview

**Figure 1.**
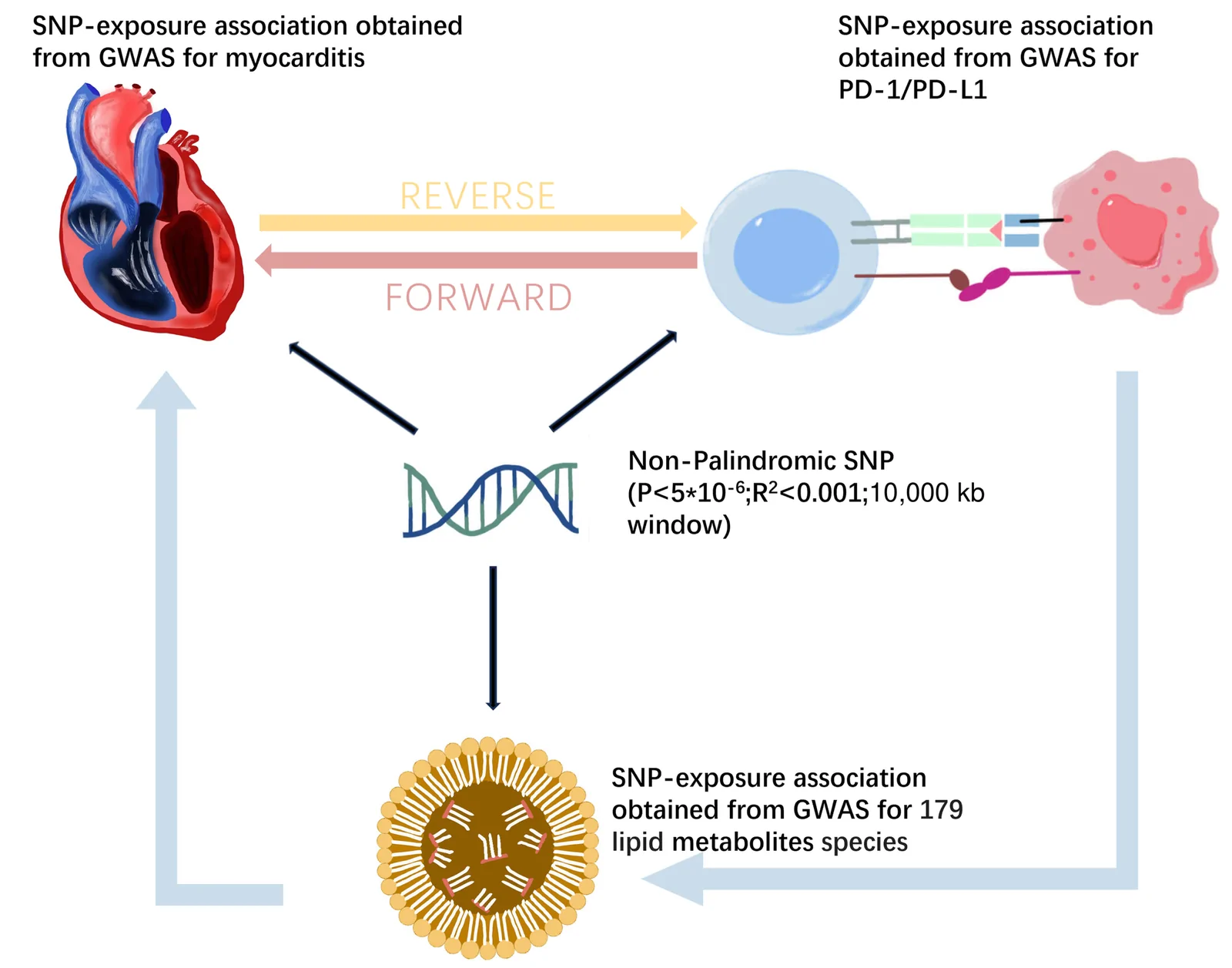
Study design for the bidirectional Mendelian randomization analyses. Genetic instruments were selected at *P* < 5 × 10^−6^ and clumped at *r*^2^ < 0.001 within 10,000 kb. GWAS = genome-wide association study, LD = linkage disequilibrium, SNP = single-nucleotide polymorphism.

The study workflow is shown in Figure 1. We used summary-level data in a bidirectional 2-sample MR framework. Forward analyses examined genetically proxied circulating PD-1 or PD-L1 as exposures and broadly defined myocarditis as the outcome; reverse analyses examined broadly defined myocarditis liability as the exposure and circulating proteins as outcomes. PD-1 and PD-L1 were prespecified comparison proteins, whereas the lipid analyses were exploratory. The design assumed that each genetic instrument was associated with its exposure, was independent of exposure–outcome confounders, and affected the outcome only through the exposure.

*Ethics statement*: This study used publicly available, de-identified GWAS summary statistics and did not access individual-level data. Therefore, additional institutional review board approval and informed consent were not required. Ethical approval and informed consent were obtained in the original studies contributing the summary-level data.

### 2.2. Data sources and outcome definitions

Discovery pQTL summary data were obtained from the UK Biobank Pharma Proteomics Project (UKB-PPP), which measured circulating proteins using the Olink Explore platform.^[23]^ Independent protein-source validation used INTERVAL SomaScan data from 3301 participants of European ancestry.^[24]^ The primary outcome was the FinnGen Release 10 registry phenotype for broadly defined myocarditis (1654 cases and 2,10,652 controls). An independent European cross-biobank GWAS of broadly defined myocarditis (GCST90018882; 633 cases and 4,27,278 controls) provided outcome-level validation from a different source.^[25]^ Plasma lipidomic summary statistics comprised 179 molecular species measured in 7174 Finnish participants.^[26]^ Dataset characteristics and analytical roles are summarized in Table 1.

**Table 1:** Characteristics of the datasets and their analytical roles.

Dataset	Analytical role	Assay or phenotype	Sample size	Ancestry
UKB-PPP	Discovery protein source	Olink Explore proteomics	33,436 (PD-L1); 33,902 (PD-1)	Predominantly European
INTERVAL	Independent protein-source validation	SomaScan proteomics	3301	European
FinnGen Release 10	Primary myocarditis outcome	Registry-based broadly defined myocarditis	1654 cases; 2,10,652 controls	Finnish/European
GCST90018882	Independent myocarditis outcome validation	Cross-biobank broadly defined myocarditis GWAS	633 cases; 4,27,278 controls	European
Ottensmann et al.	Exploratory lipid exposures and outcomes	179 molecular plasma lipid species	7174	Finnish/European

GWAS = genome-wide association study, PD-1 = programmed cell death protein 1, PD-L1 = programmed death-ligand 1, UKB-PPP = UK Biobank Pharma Proteomics Project.

FinnGen was selected as the primary myocarditis outcome because it provided publicly accessible summary statistics for a documented registry-based phenotype and was expected to have limited overlap with the proteomic cohorts. The phenotype encompasses broadly defined myocarditis and does not distinguish viral, autoimmune, toxic, vaccine-related, drug-induced, or immune checkpoint inhibitor-associated etiologies. The independent cross-biobank dataset was therefore used for outcome-level validation rather than as a replacement for FinnGen.

### 2.3. Instrument selection and strength

For the primary analyses, variants associated with each exposure at *P* < 5 × 10^−6^ were selected to retain enough independent instruments for stable multi-variant estimation. Relaxed thresholds have been used in previous MR studies of PD-1/PD-L1-related traits when conventional genome-wide significance yields too few instruments.^[27]^ Variants were clumped at *r*^2^ < 0.001 within a 10,000-kb window; exposure and outcome alleles were harmonized; and ambiguous palindromic variants were excluded. Potential associations of selected variants with alternative phenotypes were screened in PhenoScanner.^[28,29]^

Instrument strength was evaluated using the proportion of variance explained and F statistics. For continuous protein traits, the variance explained by an individual variant was approximated as *R*^2^ = 2 × effect-allele frequency × (1 − effect-allele frequency) × β^2^, and the corresponding individual *F* statistic was calculated as β^2^/SE^2^. Mean *F* statistics were summarized across each instrument set, and variants with *F* < 10 were excluded. To assess dependence on the relaxed selection threshold, a secondary analysis used the conventional *P* < 5 × 10^−8^ threshold. This analysis prioritized stronger exposure associations but retained fewer variants and was, therefore, expected to be less precise. Instrument-level data and strength metrics are provided in Tables S1, S2 and S3, Supplemental Digital Content 1–3.

The primary instrument sets were not restricted exclusively to cis variants. For instrument annotation, cis-pQTLs were defined as variants within ± 1 Mb of PDCD1 or CD274; variants outside these regions were classified as trans-pQTLs. Broader pQTL sets can improve instrument coverage and precision and have been evaluated alongside cis-only analyses in proteome-wide MR,^[18]^ although trans variants may be more susceptible to horizontal pleiotropy. We, therefore, describe the analysis as pQTL-based, avoid target-specific claims that require cis-only instruments, and address pleiotropy through phenotype screening and complementary sensitivity analyses. Comparable analyses using INTERVAL data assessed consistency across assay platforms and cohorts.

### 2.4. Mendelian randomization and sensitivity analyses

Inverse-variance weighting (IVW) was the primary estimator. MR-Egger, weighted-median, and weighted-mode estimates were used as complementary analyses when the number of instruments permitted. Cochran *Q* statistics assessed heterogeneity, and the MR-Egger intercept tested directional horizontal pleiotropy. A 2-sided *P* < .05 for the relevant diagnostic test was considered statistical evidence of heterogeneity or directional pleiotropy. Leave-one-out and funnel-plot analyses examined whether individual variants disproportionately influenced an estimate.

The MR-PRESSO global test assessed horizontal pleiotropy, with outlier and distortion tests applied when pleiotropic outliers were detected.^[30]^ Radial IVW analysis was additionally used to identify variants contributing disproportionately to heterogeneity, and estimates before and after removal of radial outliers were compared.^[31]^ Cochran *Q* and MR-Egger intercept diagnostics were repeated in the conventional-threshold analysis when the number of instruments permitted. A nonsignificant diagnostic test was interpreted as no statistical evidence of the corresponding violation, not as proof that heterogeneity or pleiotropy was absent. For the exploratory lipid analyses, diagnostic *P* values were interpreted descriptively and separately from the primary protein-outcome analysis because of the large number of correlated comparisons.

To examine a many-weak-instrument setting in the primary UKB-PPP PD-L1-to-FinnGen Release 10 analysis, we additionally applied MR-RAPS. This method allows the inclusion of many modest instruments and provides robust inference under systematic and idiosyncratic pleiotropy.^[32]^ Its direction and magnitude were compared with the primary IVW estimate. MR-RAPS was not repeated for secondary or validation datasets. Analyses were conducted in R using TwoSampleMR and the corresponding MR-RAPS implementation.

### 2.5. Meta-analysis and outcome validation

Supportive meta-analysis of PD-L1 was undertaken in 2 prespecified stages. First, the UKB-PPP and INTERVAL PD-L1 estimates for FinnGen Release 10 were combined to evaluate consistency across 2 protein sources with a common myocarditis outcome. Second, PD-L1 estimates from both protein sources were synthesized across FinnGen Release 10 and the independent myocarditis GWAS to evaluate consistency across both protein and outcome sources. Log-odds estimates were pooled using fixed-effect inverse-variance weighting. Because each supportive meta-analysis contained few estimates, a modified Hartung-Knapp (mHK) adjustment was examined as a small-sample sensitivity analysis. Cochran *Q* and *I*^2^ were summarized descriptively and were not used to select the effect model. Potential dependence among estimates sharing an exposure or outcome source was assessed in a variance-covariance sensitivity analysis that assumed a moderate pairwise correlation (ρ = 0.50). Leave-one-estimate-out analyses examined whether omitting any single estimate reversed the pooled direction. Because the exact covariance could not be quantified, all pooled estimates were interpreted as supportive summaries rather than independent causal estimates.

### 2.6. Statistical analysis

All tests were 2-sided. For the 2 prespecified protein exposures, nominal *P* < .05 defined the primary evidence threshold. Benjamini-Hochberg false discovery rate (BH-FDR) correction across the PD-1/PD-L1 family was calculated as a secondary multiplicity assessment and is reported as a dedicated sensitivity-analysis result in Table S5, Supplemental Digital Content 4 rather than as a separate adjusted-P column. The conventional instrument-significance threshold was also treated as a secondary analysis. The 179-lipid analyses remained exploratory: nominal associations were retained to describe the original signal pattern, whereas multiplicity-adjusted findings were used only to qualify interpretation. Effects for binary myocarditis outcomes are presented as ORs with 95% CIs; effects for continuous protein or lipid outcomes are presented as beta coefficients with 95% CIs.

## 3. Results

### 3.1. Instrument selection and strength

At the primary instrument threshold, 28 SNPs were available for UKB-PPP PD-L1/CD274 and 59 for UKB-PPP PD-1/PDCD1; INTERVAL contributed 5 PD-L1 and 13 PD-1 instruments. The reverse analyses used FinnGen Release 10 myocarditis instruments, and 1887 instruments were available across the 179 lipid traits. All retained instruments had *F* statistics >10. Individual *R*^2^ and *F* statistics and instrument-set summaries are provided in Tables S1–S3, Supplemental Digital Content 1–3.

### 3.2. Genetically proxied circulating PD-1/PD-L1 and broadly defined myocarditis

**Figure 2.**
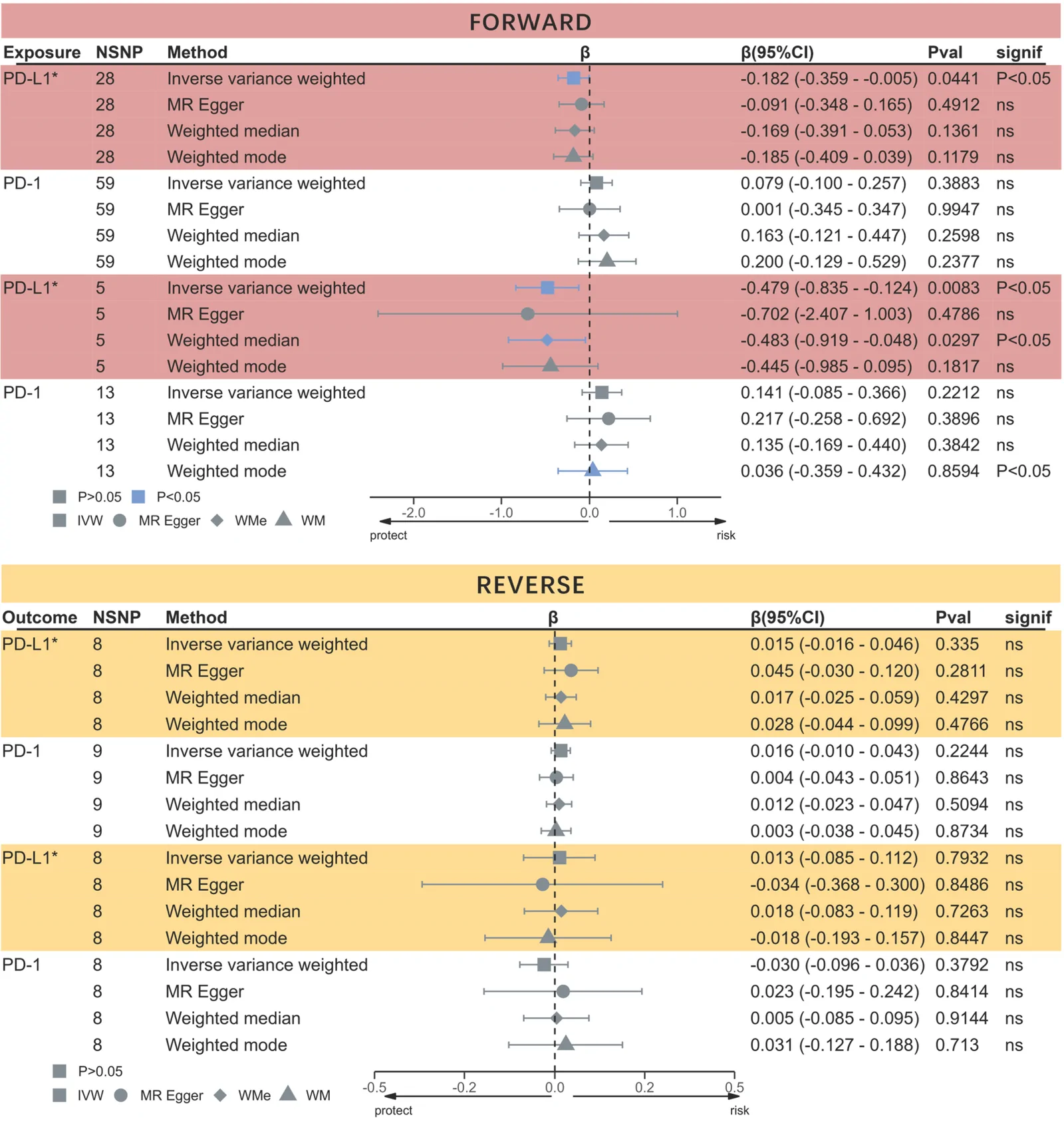
Bidirectional Mendelian randomization estimates for circulating PD-1/PD-L1 and broadly defined myocarditis. Forward analyses used 28 UKB-PPP PD-L1 SNPs, 59 UKB-PPP PD-1 SNPs, 5 INTERVAL PD-L1 SNPs, and 13 INTERVAL PD-1 SNPs. ORs are shown when myocarditis is the outcome; beta coefficients are shown for continuous protein outcomes. Blue symbols denote *P* < .05. CI = confidence interval, IVW = inverse-variance weighted, MR = Mendelian randomization, PD-1 = programmed cell death protein 1, PD-L1 = programmed death-ligand 1, SNP = single-nucleotide polymorphism, UKB-PPP = UK Biobank Pharma Proteomics Project.

In the UKB-PPP discovery analysis, higher genetically proxied circulating PD-L1 was inversely associated with FinnGen Release 10 myocarditis (βIVW = −0.182, 95% CI: −0.359 to −0.005; *P* = .0441; OR 0.834, 95% CI: 0.698–0.995; 28 SNPs), whereas genetically proxied circulating PD-1 showed no clear association (βIVW = 0.079, 95% CI: −0.100 to 0.257; *P* = .3883; OR 1.082, 95% CI: 0.905–1.293; 59 SNPs). In the independent INTERVAL protein-source analysis against FinnGen Release 10, the PD-L1 estimate was also inverse (βIVW = −0.479, 95% CI: −0.835 to −0.124; *P* = .0083; OR 0.619, 95% CI: 0.434–0.883; 5 SNPs), whereas the PD-1 estimate was not significant (βIVW = 0.141, 95% CI: −0.085 to 0.366; *P* = .2212; OR 1.151, 95% CI: 0.919–1.442; 13 SNPs). These principal results are shown in Figure 2, and complete forward and reverse protein results are provided in Table S4, Supplemental Digital Content 5. The conventional-threshold analysis, BH-FDR correction, complementary estimators, heterogeneity and pleiotropy diagnostics, and influence analyses were also performed; their detailed results are provided in Table S5, Supplemental Digital Content 4.

### 3.3. Robustness and sensitivity analyses

At the conventional *P* < 5 × 10^−8^ threshold, the UKB-PPP PD-L1 estimate remained inverse but was less precise because fewer instruments were retained. The BH-FDR analysis across the 2 prespecified proteins was used as a secondary multiplicity assessment. Full numerical results for the strict-threshold and BH-FDR analyses are provided in Table S5, Supplemental Digital Content 4.

For the primary 28-SNP PD-L1 estimate, the IVW Q test, MR-Egger intercept, and MR-PRESSO global test provided no statistical evidence of heterogeneity or directional horizontal pleiotropy, and MR-PRESSO detected no outlier. Radial IVW identified 1 potentially influential variant, but its removal preserved the inverse estimate. Leave-one-out and complementary-estimator analyses likewise did not indicate that a single variant drove the overall direction. Detailed diagnostic and influence results are provided in Table S5, Supplemental Digital Content 4; scatter and leave-one-out plots are provided in Figures S1 and S2, Supplemental Digital Content 6 and 7.

For the primary UKB-PPP PD-L1-to-FinnGen Release 10 comparison, MR-RAPS yielded an OR of 0.843 (95% CI: 0.718–0.989; *P* = .0366). The estimate was similar in magnitude to the primary IVW result and retained the inverse direction. MR-RAPS was applied only to the primary dataset (Tables 2 and S5, Supplemental Digital Content 4).

**Table 2:** Independent outcome validation, MR-RAPS sensitivity analysis, and supportive meta-analysis of circulating PD-L1 and myocarditis.

Analysis	Method (SNPs/estimates)	OR (95% CI)	*P* value	Sensitivity analysis/ interpretation
UKB-PPP PD-L1 → GCST90018882 myocarditis	IVW (18 SNPs)	0.781 (0.642–0.951)	.0138	Inverse estimate
INTERVAL PD-L1 → GCST90018882 myocarditis	Wald ratio (1 SNP)	0.544 (0.310–0.955)	.034	Inverse estimate
UKB-PPP PD-L1 → FinnGen Release 10 myocarditis	MR-RAPS (28 SNPs)	0.843 (0.718–0.989)	.0366	Directionally consistent with primary IVW
UKB-PPP and INTERVAL PD-L1 estimates for FinnGen Release 10	Fixed-effect pooling (2 estimates)	0.838 (0.705–0.996)	.045	mHK: OR 0.838 (0.733–0.958); *P* = .038
Both PD-L1 protein sources across FinnGen Release 10 and GCST90018882	Fixed-effect pooling (4 estimates)	0.830 (0.715–0.963)	.014	mHK: OR 0.797 (0.651–0.975); *P* = .037

CI = confidence interval, IVW = inverse-variance weighted, mHK = modified Hartung-Knapp, MR-RAPS = Mendelian randomization robust adjusted profile score, OR = odds ratio, PD-L1 = programmed death-ligand 1, SNP = single-nucleotide polymorphism, UKB-PPP = UK Biobank Pharma Proteomics Project. The mHK results are sensitivity estimates based on only 2 or 4 contributing estimates and should be interpreted cautiously.

### 3.4. Outcome validation and meta-analysis

Against the independent myocarditis GWAS, UKB-PPP PD-L1 instruments yielded an IVW OR of 0.781 (95% CI: 0.642–0.951; *P* = .0138; 18 SNPs), whereas the single available INTERVAL PD-L1 instrument produced a Wald-ratio estimate of OR 0.544 (95% CI: 0.310–0.955; *P* = .034). Both outcome-validation analyses yielded inverse estimates with nominal *P* < .05 (Tables 2 and S6, Supplemental Digital Content 8).

When the UKB-PPP and INTERVAL PD-L1 estimates for FinnGen Release 10 were combined, the fixed-effect pooled estimate was OR 0.838 (95% CI: 0.705–0.996; *P* = .045), and the mHK sensitivity estimate was OR 0.838 (95% CI: 0.733–0.958; *P* = .038). When estimates from both protein sources were synthesized across FinnGen Release 10 and the independent myocarditis GWAS, the fixed-effect pooled estimate was OR 0.830 (95% CI: 0.715–0.963; *P* = .014), and the mHK sensitivity estimate was OR 0.797 (95% CI: 0.651–0.975; *P* = .037; Tables 2 and S6, Supplemental Digital Content 8).

Cochran *Q* and *I*^2^ showed no clear evidence of between-estimate heterogeneity in either synthesis, although the small number of contributing estimates limited diagnostic power. Under the moderate-correlation scenario (ρ = 0.50), the inverse associations were retained (*P* = .047 for the 2-estimate supportive meta-analysis and *P* = .045 for the 4-estimate supportive meta-analysis), and leave-one-estimate-out analyses did not reverse the pooled direction. Detailed results are provided in Table S6, Supplemental Digital Content 8; these supportive meta-analyses were interpreted as supportive evidence of directional consistency.

### 3.5. Reverse Mendelian randomization

Reverse MR did not support an effect of FinnGen Release 10 myocarditis liability on circulating PD-L1 (UKB-PPP β = 0.015, 95% CI: −0.016 to 0.046; *P* = .335) or PD-1 (β = 0.016, 95% CI: −0.010 to 0.043; *P* = .224). The Release 10 reverse estimates are shown in Figure 2, and complete results are provided in Table S4, Supplemental Digital Content 5. Corresponding scatter and leave-one-out plots are provided in Figures S3 and S4, Supplemental Digital Content 9 and 10.

### 3.6. Exploratory lipidomic Mendelian randomization

In the exploratory analyses, genetically proxied circulating PD-L1 was nominally associated with selected diacylglycerol, triacylglycerol, and cholesteryl ester species, including inverse associations with cholesteryl ester (27:1/20:2) and cholesteryl ester (27:1/20:5) and positive associations with triacylglycerol (51:1) and diacylglycerol (18:1_18:3; Figs. 3 and S5, Supplemental Digital Content 11). Selected phosphatidylcholine, phosphatidylethanolamine, sphingomyelin, and triacylglycerol species were nominally associated with broadly defined myocarditis (Figs. 4 and S6, Supplemental Digital Content 12). These exploratory findings were retained solely for hypothesis generation; the complete nominal results and multiplicity summary are provided in Table S7, Supplemental Digital Content 13.

**Figure 3.**
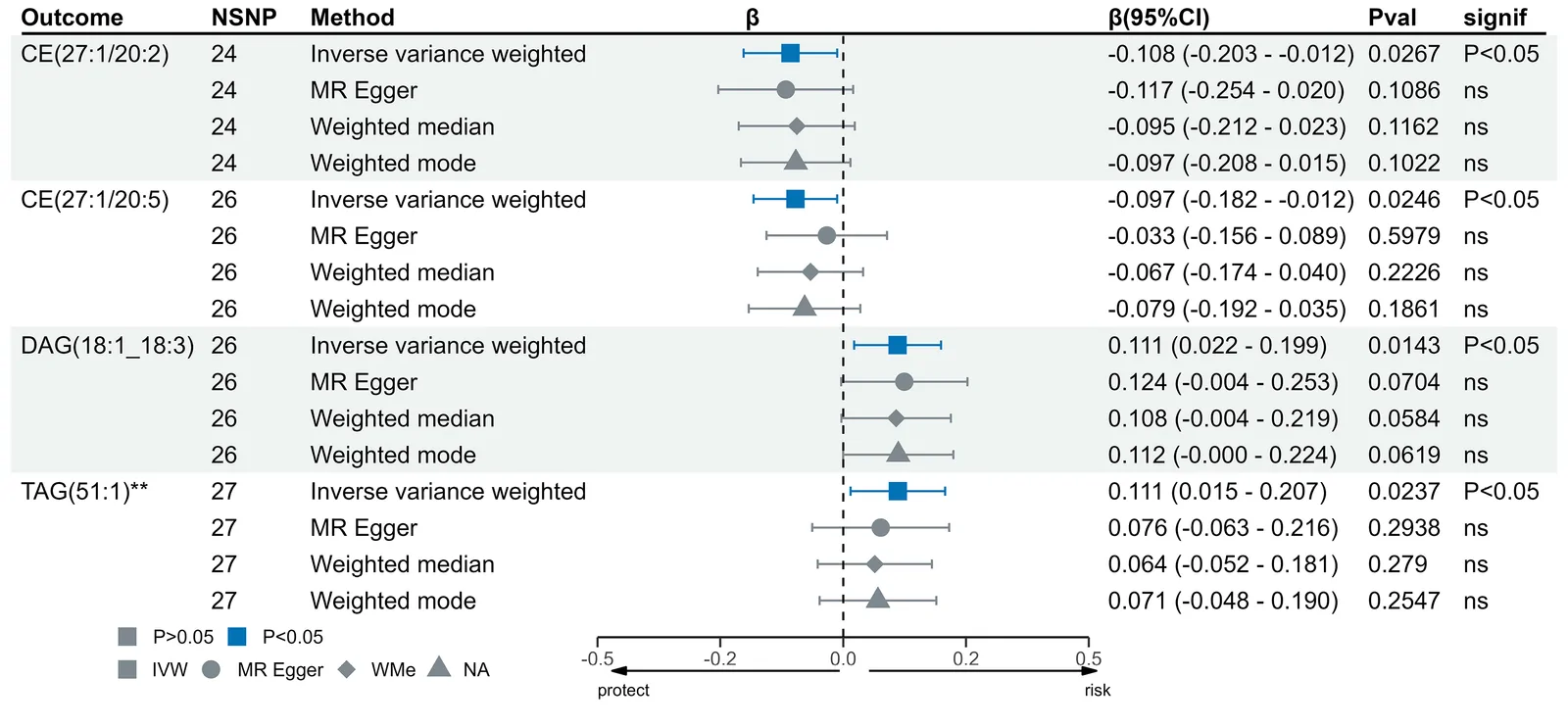
Selected associations of genetically proxied circulating PD-L1 with plasma lipid species. CE = cholesteryl ester, CI = confidence interval, DAG = diacylglycerol, IVW = inverse-variance weighted, MR = Mendelian randomization, SNP = single-nucleotide polymorphism, TAG = triacylglycerol.

**Figure 4.**
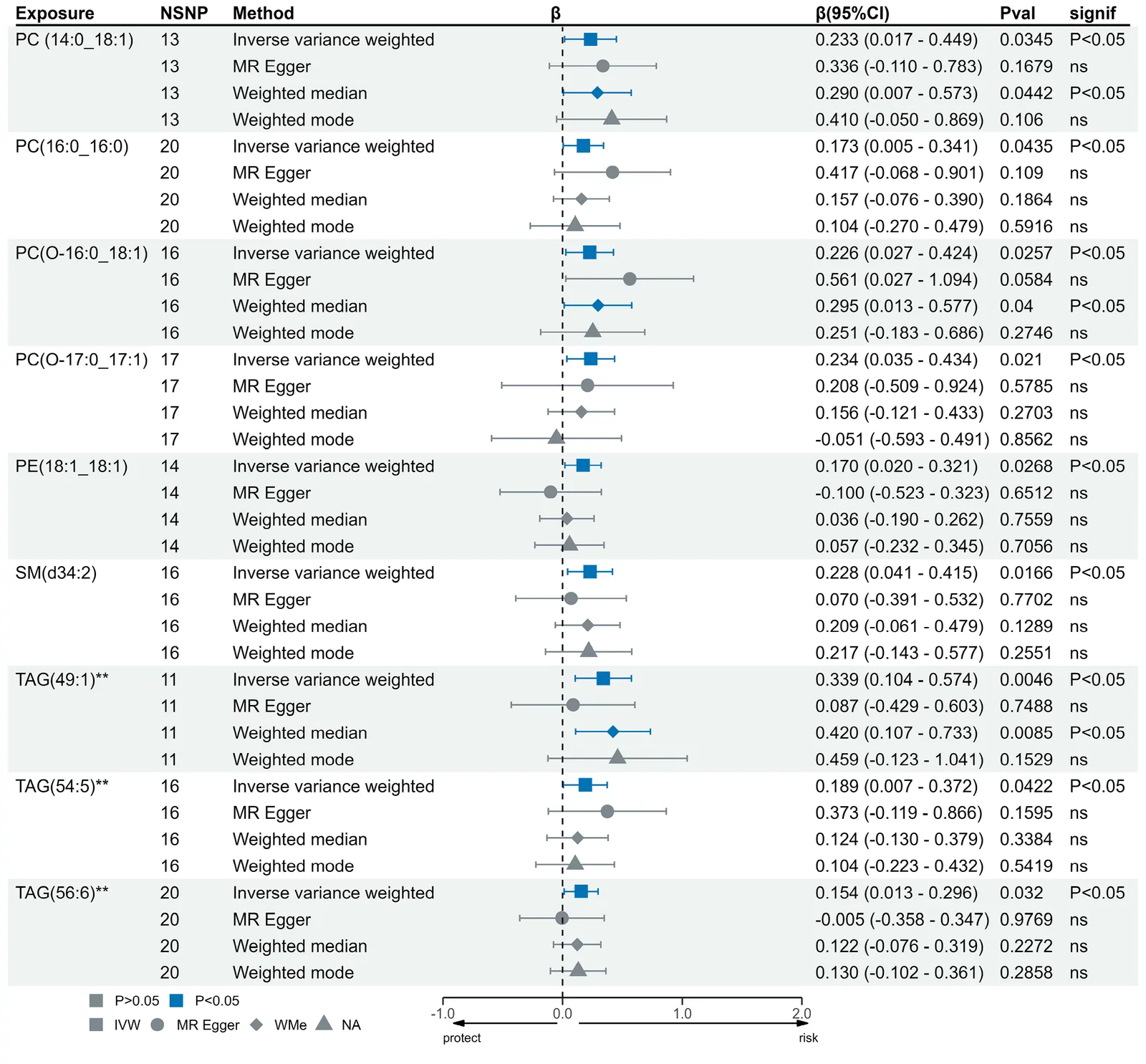
Selected associations of genetically proxied plasma lipid species with broadly defined myocarditis. CI = confidence interval, IVW = inverse-variance weighted, MR = Mendelian randomization, PC = phosphatidylcholine, PE = phosphatidylethanolamine, SM = sphingomyelin, SNP = single-nucleotide polymorphism, TAG = triacylglycerol.

## 4. Discussion

This bidirectional MR study provides suggestive human genetic evidence of an inverse association between circulating PD-L1 and broadly defined myocarditis. The UKB-PPP discovery and INTERVAL protein-source estimates for PD-L1 against FinnGen Release 10 were both inverse, and both estimates against the independent myocarditis GWAS were protective in direction. Fixed-effect syntheses across protein sources and across both protein and outcome sources retained inverse pooled estimates, while mHK, moderate-correlation, and leave-one-estimate-out analyses did not reverse the direction. MR-RAPS was also directionally consistent with IVW, whereas reverse MR did not support an effect of myocarditis liability on circulating PD-1 or PD-L1. The conventional-threshold analysis retained the inverse direction but, as expected with fewer variants, was less precise. Together, these findings support the original biological signal while more clearly defining its uncertainty.

The inverse direction is biologically compatible with the inhibitory function of the PD-1/PD-L1 pathway in T-cell activation and peripheral tolerance.^[8]^ PD-L1 engagement of PD-1 limits effector T-cell signaling, cytokine production, and tissue-directed immune injury. In the heart, endothelial PD-L1 has been shown experimentally to restrain CD8+ T-cell-mediated damage, providing a mechanistic link between this checkpoint pathway and myocardial immune homeostasis.^[9]^ A lifelong genetically proxied increase in circulating PD-L1 may therefore reflect a more inhibitory immune milieu or related regulatory processes that reduce susceptibility to inflammatory myocardial injury. Although circulating protein measurements do not identify the relevant cardiac cell type, the genetic association supports prioritization of this pathway in mechanistic myocarditis research.

Clinical and pharmacovigilance studies have established that pharmacologic PD-1/PD-L1 blockade can precipitate rare but severe myocarditis.^[10,12-14]^ This observation is directionally compatible with a protective role for intact PD-1/PD-L1 signaling but does not represent the exposure evaluated by MR. Genetic instruments reflect small lifelong differences in circulating protein concentrations, whereas immune checkpoint inhibitors impose time-limited, high-affinity target blockade in patients with cancer and may be combined with other therapies. The present findings therefore inform pathway biology and broadly defined myocarditis rather than providing a quantitative estimate of checkpoint-inhibitor-associated myocarditis risk.

This study also extends earlier MR research linking genetically proxied circulating PD-1/PD-L1 traits to cancer, circulating biomarkers, lipid species, and the gut microbiome.^[27,33-35]^ By focusing on myocarditis and using 2 proteomic sources, an independent outcome dataset, bidirectional analyses, and supportive meta-analysis, the current work examines a clinically relevant cardiac phenotype not addressed by those studies. Directional consistency across protein and outcome sources is informative because UKB-PPP used Olink whereas INTERVAL used SomaScan. Nevertheless, differences in assay chemistry, epitope recognition, instrument composition, and cohort characteristics mean that cross-source agreement should be interpreted as complementary evidence rather than exact replication.

The lipid analyses identified selected molecular species nominally associated with PD-L1 or myocarditis and offer exploratory clues regarding immune-metabolic interactions. Lipid composition can influence immune-cell activation, membrane signaling, mitochondrial function, and myocardial energetics.^[19-22,36]^ Findings involving cholesteryl esters, diacylglycerols, triacylglycerols, phospholipids, and sphingomyelins may therefore help nominate pathways for future investigation. However, these associations do not demonstrate mediation between PD-L1 and myocarditis and require independent replication and mechanistic validation.

Strengths of this study include its bidirectional design, large-scale proteomic summary data, evaluation across different assay platforms and myocarditis sources, explicit assessment of instrument strength, multiple pleiotropy and influence diagnostics, an additional weak-instrument-robust estimator, and prespecified supportive meta-analyses. These components address distinct sources of uncertainty and collectively support a more nuanced interpretation of the principal PD-L1 finding without displacing the prespecified primary estimate.

Several limitations merit consideration. First, the primary UKB-PPP analysis suggested an inverse association between genetically proxied circulating PD-L1 and broadly defined myocarditis, and this direction was supported by analyses using an independent myocarditis outcome dataset and by the supportive meta-analysis. However, when the 2 prespecified protein hypotheses, PD-1 and PD-L1, were considered within the same testing family, adjustment for multiple testing attenuated the statistical evidence, and the PD-L1 association no longer met the conventional threshold for statistical significance. The finding should therefore be interpreted as suggestive rather than confirmatory genetic evidence. In addition, the study was based on summary-level genetic data and lacked direct experimental or prospective clinical validation. Further confirmation is warranted in independent, well-phenotyped, preferably multicenter myocarditis cohorts with serial measurements of circulating PD-L1. Second, selecting instruments at *P* < 5 × 10^−6^ increased instrument availability but was less stringent than conventional genome-wide significance and may be more susceptible to weak-instrument bias, winner’s-curse effects, and horizontal pleiotropy.^[32,37,38]^ All retained instruments had *F* > 10, the conventional-threshold estimate remained inverse but imprecise, and MR-RAPS produced a similar inverse estimate; however, these analyses reduce but do not eliminate residual weak-instrument or pleiotropy-related bias. The pQTL sets were also not exclusively cis. Trans-pQTLs can improve coverage and precision but may act through pathways unrelated to PD-1/PD-L1; accordingly, the estimates should be interpreted as circulating-protein MR rather than purely target-specific cis-pQTL effects.^[17,18]^ Third, the FinnGen and cross-biobank outcomes represent broadly defined myocarditis and cannot distinguish infectious, autoimmune, toxic, vaccine-related, drug-induced, or immune checkpoint inhibitor-associated disease. Such etiologic heterogeneity may dilute or mask subtype-specific associations, and the findings cannot be extrapolated directly to checkpoint-inhibitor myocarditis. Fourth, genetically proxied circulating soluble PD-1/PD-L1 levels may not represent membrane-bound expression in myocardium, endothelium, or infiltrating immune cells and do not reproduce drug dose, timing, tissue distribution, or target occupancy. Fifth, Olink and SomaScan differ in assay reagents and epitope recognition, while cohort composition and instrument availability also varied. Each meta-analysis contained few estimates, limiting the power of *Q* and *I*^2^; the mHK and correlation sensitivity results should therefore be interpreted cautiously. Exact overlap and covariance between the myocarditis outcomes could not be quantified, and participant overlap can bias 2-sample MR estimates, particularly when instruments are weak.^[39]^ Although the ρ = 0.50 and leave-one-estimate-out analyses preserved direction, the pooled estimates remain supportive and may be more precise than warranted if samples overlap. Finally, the predominantly European ancestry of the datasets limits generalizability, and summary-level data precluded evaluation of nonlinear effects, sex- or age-specific associations, clinical subgroups, interactions, and individual-level confounding structures. The lipid analyses involved many comparisons, were exploratory, and did not include formal mediation, tissue-level validation, or experimental confirmation.

## 5. Conclusion

The discovery association, outcome-level validation, and supportive meta-analyses provide suggestive genetic evidence that higher genetically proxied circulating PD-L1 may be associated with a lower risk of broadly defined myocarditis. Although these findings do not directly estimate the effects of PD-1/PD-L1 inhibitor therapy, they support the biological relevance of PD-L1-related immune regulation in myocarditis and provide a rationale for further mechanistic and translational investigation. The exploratory lipid findings additionally identify potentially informative immune-metabolic pathways that warrant targeted confirmation.

## Acknowledgments

We sincerely thank the original GWAS investigators and consortia for generating and sharing the summary statistics. The authors also thank International Science Editing for professional English-language editing by a native-English specialist.

## Author contributions

**Conceptualization:** Fengyi Liu, Qian Yang.

**Data curation:** Fengyi Liu.

**Formal analysis:** Fengyi Liu.

**Funding acquisition:** Qian Yang.

**Project administration:** Qian Yang.

**Visualization:** Fengyi Liu.

**Writing – original draft:** Fengyi Liu.

**Writing – review & editing:** Qian Yang.


























