# Supplementary material for: Genetically proxied circulating PD-1/PD-L1 levels and broadly defined myocarditis: A bidirectional Mendelian randomization study with exploratory lipidomic analyses
Source: Medicine (Baltimore). 2026 Aug 28;105(35):e50475. doi: 10.1097/MD.0000000000050475 (PMC13529170; doi:10.1097/MD.0000000000050475)
Supplement: Supplementary file 1 [file medi-105-e50475-s001.pdf]

Supplementary Table 1. Instrument-level data and strength metrics for PD-1 and PD-L1.

Protein	Source	SNP	Chromosome	Position	Effect allele	Other allele	Effect-allele frequency	Beta (exposure)	SE (exposure)	P value (exposure)	R^2	F statistic	F > 10	Primary outcome
PD-L1	UKB-PPP	rs10435743	9	5,384,069	T	A	0.2015	-0.0818	0.00931285	1.5300E-18	0.002155	72.213	TRUE	FinnGen Release 10 broadly defined myocarditis
PD-L1	UKB-PPP	rs10815242	9	5,550,882	C	T	0.1794	-0.0527	0.00983298	8.5600E-08	0.000816	27.320	TRUE	FinnGen Release 10 broadly defined myocarditis
PD-L1	UKB-PPP	rs11057837	12	124,827,174	T	C	0.1025	0.0559	0.0119035	2.6200E-06	0.000576	19.259	TRUE	FinnGen Release 10 broadly defined myocarditis
PD-L1	UKB-PPP	rs11221995	11	130,198,659	T	C	0.4308	-0.0364	0.0072805	5.8100E-07	0.000649	21.719	TRUE	FinnGen Release 10 broadly defined myocarditis
PD-L1	UKB-PPP	rs11242113	5	132,441,542	A	G	0.1856	0.0487	0.00932268	1.7900E-07	0.000716	23.956	TRUE	FinnGen Release 10 broadly defined myocarditis
PD-L1	UKB-PPP	rs112635299	14	94,371,805	T	G	0.0217	0.2161	0.0247018	2.1800E-18	0.001984	66.451	TRUE	FinnGen Release 10 broadly defined myocarditis
PD-L1	UKB-PPP	rs117150152	18	44,693,688	C	T	0.0063	0.2562	0.0513221	5.9600E-07	0.000818	27.385	TRUE	FinnGen Release 10 broadly defined myocarditis
PD-L1	UKB-PPP	rs13105210	4	144,341,775	T	C	0.3049	-0.0374	0.00783545	1.8100E-06	0.000593	19.836	TRUE	FinnGen Release 10 broadly defined myocarditis
PD-L1	UKB-PPP	rs1354034	3	56,815,721	C	T	0.6041	0.0486	0.00731308	3.0600E-11	0.001129	37.791	TRUE	FinnGen Release 10 broadly defined myocarditis
PD-L1	UKB-PPP	rs144000478	4	71,290,126	A	G	0.0149	0.1440	0.0310279	3.4500E-06	0.000607	20.318	TRUE	FinnGen Release 10 broadly defined myocarditis
PD-L1	UKB-PPP	rs17652117	11	28,462,098	G	A	0.2284	-0.0411	0.00857193	1.6500E-06	0.000595	19.901	TRUE	FinnGen Release 10 broadly defined myocarditis
PD-L1	UKB-PPP	rs2165109	2	111,061,081	C	A	0.2609	-0.0444	0.008168	5.4900E-08	0.000760	25.426	TRUE	FinnGen Release 10 broadly defined myocarditis
PD-L1	UKB-PPP	rs258330	16	89,663,166	G	T	0.4184	0.0351	0.00731517	1.5500E-06	0.000601	20.109	TRUE	FinnGen Release 10 broadly defined myocarditis
PD-L1	UKB-PPP	rs2817479	1	245,810,554	T	G	0.5343	-0.0366	0.00783196	3.0200E-06	0.000666	22.266	TRUE	FinnGen Release 10 broadly defined myocarditis
PD-L1	UKB-PPP	rs3184504	12	111,446,804	C	T	0.5170	-0.0824	0.00716682	1.2700E-30	0.003394	113.868	TRUE	FinnGen Release 10 broadly defined myocarditis
PD-L1	UKB-PPP	rs4657041	1	161,509,069	C	T	0.5357	0.0607	0.00727502	6.8700E-17	0.001835	61.474	TRUE	FinnGen Release 10 broadly defined myocarditis
PD-L1	UKB-PPP	rs4759076	12	54,336,088	C	T	0.4575	0.0404	0.00728561	2.8700E-08	0.000811	27.147	TRUE	FinnGen Release 10 broadly defined myocarditis
PD-L1	UKB-PPP	rs579459	9	133,278,724	C	T	0.2077	-0.0481	0.00923441	1.9000E-07	0.000761	25.476	TRUE	FinnGen Release 10 broadly defined myocarditis
PD-L1	UKB-PPP	rs61747728	1	179,557,079	T	C	0.0379	0.1037	0.0187373	3.1600E-08	0.000783	26.216	TRUE	FinnGen Release 10 broadly defined myocarditis
PD-L1	UKB-PPP	rs61772249	1	7,508,016	A	G	0.3278	-0.0358	0.00781303	4.6600E-06	0.000564	18.875	TRUE	FinnGen Release 10 broadly defined myocarditis
PD-L1	UKB-PPP	rs6993770	8	105,569,300	T	A	0.2865	-0.0607	0.00795314	2.2600E-14	0.001507	50.477	TRUE	FinnGen Release 10 broadly defined myocarditis
PD-L1	UKB-PPP	rs7025653	9	5,505,508	C	A	0.1433	0.1587	0.0107864	5.2200E-49	0.006187	208.131	TRUE	FinnGen Release 10 broadly defined myocarditis
PD-L1	UKB-PPP	rs704	17	28,367,840	A	G	0.4733	-0.0337	0.00718601	2.6500E-06	0.000568	18.997	TRUE	FinnGen Release 10 broadly defined myocarditis
PD-L1	UKB-PPP	rs72916825	6	52,163,881	A	T	0.1102	-0.0525	0.011458	4.6000E-06	0.000541	18.087	TRUE	FinnGen Release 10 broadly defined myocarditis
PD-L1	UKB-PPP	rs78444298	1	184,702,964	A	G	0.0193	0.1960	0.0263345	9.9600E-14	0.001455	48.726	TRUE	FinnGen Release 10 broadly defined myocarditis
PD-L1	UKB-PPP	rs8178824	17	66,228,657	T	C	0.0299	0.1505	0.021156	1.1300E-12	0.001315	44.035	TRUE	FinnGen Release 10 broadly defined myocarditis
PD-L1	UKB-PPP	rs822335	9	5,448,218	C	T	0.6608	0.3111	0.00797119	1.0000E-200	0.043387	1516.398	TRUE	FinnGen Release 10 broadly defined myocarditis
PD-L1	UKB-PPP	rs892090	19	55,027,704	G	T	0.8341	0.0474	0.00970582	1.0400E-06	0.000622	20.801	TRUE	FinnGen Release 10 broadly defined myocarditis
PD-1	UKB-PPP	rs1036230	19	10,629,334	A	G	0.7773	-0.0791	0.00875117	1.5100E-19	0.002169	73.687	TRUE	FinnGen Release 10 broadly defined myocarditis
PD-1	UKB-PPP	rs10788639	10	80,473,469	G	C	0.7243	0.0451	0.00822773	4.3000E-08	0.000811	27.528	TRUE	FinnGen Release 10 broadly defined myocarditis
PD-1	UKB-PPP	rs10975133	9	5,486,390	G	T	0.2301	0.0465	0.00865569	7.7700E-08	0.000766	25.994	TRUE	FinnGen Release 10 broadly defined myocarditis
PD-1	UKB-PPP	rs10985070	9	120,873,843	A	C	0.5661	-0.0416	0.00737568	1.7300E-08	0.000849	28.815	TRUE	FinnGen Release 10 broadly defined myocarditis
PD-1	UKB-PPP	rs11072115	15	69,743,076	T	G	0.4765	0.0418	0.00729575	1.0300E-08	0.000870	29.530	TRUE	FinnGen Release 10 broadly defined myocarditis
PD-1	UKB-PPP	rs111326703	3	110,783,904	G	A	0.0224	0.1185	0.0245408	1.3800E-06	0.000614	20.841	TRUE	FinnGen Release 10 broadly defined myocarditis
PD-1	UKB-PPP	rs111452679	1	1,224,783	A	G	0.1099	-0.0621	0.0116326	9.4200E-08	0.000754	25.586	TRUE	FinnGen Release 10 broadly defined myocarditis
PD-1	UKB-PPP	rs11247968	2	241,991,374	G	A	0.4859	-0.0610	0.00755406	6.4100E-16	0.001862	63.235	TRUE	FinnGen Release 10 broadly defined myocarditis
PD-1	UKB-PPP	rs1155060	2	190,701,713	G	A	0.5877	-0.0439	0.00755463	6.3100E-09	0.000933	31.662	TRUE	FinnGen Release 10 broadly defined myocarditis
PD-1	UKB-PPP	rs11571291	2	203,856,409	C	T	0.4240	-0.0577	0.00750267	1.4700E-14	0.001626	55.211	TRUE	FinnGen Release 10 broadly defined myocarditis
PD-1	UKB-PPP	rs11888817	2	62,303,845	C	T	0.6420	0.0536	0.00772104	3.7400E-12	0.001322	44.890	TRUE	FinnGen Release 10 broadly defined myocarditis
PD-1	UKB-PPP	rs12433460	14	105,796,336	A	C	0.2688	-0.0450	0.00910023	7.7200E-07	0.000795	26.981	TRUE	FinnGen Release 10 broadly defined myocarditis

Protein	Source	SNP	Chromosome	Position	Effect allele	Other allele	Effect-allele frequency	Beta (exposure)	SE (exposure)	P value (exposure)	R²	F statistic	F > 10	Primary outcome
PD-1	UKB-PPP	rs12449427	17	7,269,960	C	G	0.2540	0.0592	0.0083954	1.8000E-12	0.001327	45.052	TRUE	FinnGen Release 10 broadly defined myocarditis
PD-1	UKB-PPP	rs12570524	10	5,713,869	T	C	0.2318	-0.0426	0.00861818	7.7000E-07	0.000646	21.925	TRUE	FinnGen Release 10 broadly defined myocarditis
PD-1	UKB-PPP	rs12667978	7	129,137,407	T	C	0.5715	-0.0351	0.00732787	1.6800E-06	0.000603	20.454	TRUE	FinnGen Release 10 broadly defined myocarditis
PD-1	UKB-PPP	rs12722592	10	6,016,453	C	T	0.1185	0.0533	0.0113052	2.4100E-06	0.000594	20.145	TRUE	FinnGen Release 10 broadly defined myocarditis
PD-1	UKB-PPP	rs12749014	1	156,775,707	T	C	0.6898	-0.0407	0.00785938	2.3100E-07	0.000707	23.998	TRUE	FinnGen Release 10 broadly defined myocarditis
PD-1	UKB-PPP	rs12772559	10	69,424,600	C	G	0.2564	0.0841	0.00837156	9.8700E-24	0.002696	91.628	TRUE	FinnGen Release 10 broadly defined myocarditis
PD-1	UKB-PPP	rs13152362	4	122,319,464	A	G	0.2314	-0.0405	0.00867658	3.0900E-06	0.000583	19.765	TRUE	FinnGen Release 10 broadly defined myocarditis
PD-1	UKB-PPP	rs1327264	6	51,304,738	C	A	0.4441	0.0361	0.00735233	8.8200E-07	0.000645	21.882	TRUE	FinnGen Release 10 broadly defined myocarditis
PD-1	UKB-PPP	rs146219482	1	114,143,032	G	A	0.0127	0.1637	0.0327275	5.7200E-07	0.000670	22.732	TRUE	FinnGen Release 10 broadly defined myocarditis
PD-1	UKB-PPP	rs150699287	12	26,464,351	T	A	0.0136	0.1495	0.0326426	4.6600E-06	0.000600	20.368	TRUE	FinnGen Release 10 broadly defined myocarditis
PD-1	UKB-PPP	rs150816167	1	179,602,727	C	T	0.0417	0.1162	0.0183866	2.6100E-10	0.001080	36.646	TRUE	FinnGen Release 10 broadly defined myocarditis
PD-1	UKB-PPP	rs17825630	3	56,916,027	A	G	0.1434	0.0613	0.0108321	1.5100E-08	0.000924	31.338	TRUE	FinnGen Release 10 broadly defined myocarditis
PD-1	UKB-PPP	rs191998647	12	74,983,136	C	G	0.0096	-0.1895	0.0376305	4.7400E-07	0.000684	23.196	TRUE	FinnGen Release 10 broadly defined myocarditis
PD-1	UKB-PPP	rs2075876	21	44,289,270	A	G	0.1099	-0.0597	0.0116334	2.8400E-07	0.000698	23.665	TRUE	FinnGen Release 10 broadly defined myocarditis
PD-1	UKB-PPP	rs2084108	19	4,019,969	C	T	0.3083	-0.0478	0.0080121	2.4100E-09	0.000975	33.086	TRUE	FinnGen Release 10 broadly defined myocarditis
PD-1	UKB-PPP	rs212388	6	159,069,404	T	C	0.5829	0.0366	0.00736102	6.4000E-07	0.000653	22.155	TRUE	FinnGen Release 10 broadly defined myocarditis
PD-1	UKB-PPP	rs246890	5	127,405,252	T	C	0.9875	0.1615	0.03475	3.3500E-06	0.000646	21.899	TRUE	FinnGen Release 10 broadly defined myocarditis
PD-1	UKB-PPP	rs2476601	1	113,834,946	G	A	0.8993	-0.0857	0.0120865	1.3200E-12	0.001331	45.192	TRUE	FinnGen Release 10 broadly defined myocarditis
PD-1	UKB-PPP	rs2659005	17	81,244,914	T	C	0.4417	0.0645	0.00733644	1.4100E-18	0.002054	69.775	TRUE	FinnGen Release 10 broadly defined myocarditis
PD-1	UKB-PPP	rs3131622	6	31,452,723	G	T	0.4501	0.0544	0.00729162	9.0700E-14	0.001462	49.643	TRUE	FinnGen Release 10 broadly defined myocarditis
PD-1	UKB-PPP	rs3184504	12	111,446,804	C	T	0.5165	-0.0993	0.00723582	7.3000E-43	0.004925	167.791	TRUE	FinnGen Release 10 broadly defined myocarditis
PD-1	UKB-PPP	rs34290285	2	241,759,225	A	G	0.2564	0.1496	0.00854821	1.5100E-68	0.008531	291.686	TRUE	FinnGen Release 10 broadly defined myocarditis
PD-1	UKB-PPP	rs35073649	17	49,345,148	T	C	0.3677	-0.0472	0.00753494	3.8700E-10	0.001034	35.097	TRUE	FinnGen Release 10 broadly defined myocarditis
PD-1	UKB-PPP	rs37471	1	43,811,876	T	C	0.3522	0.0709	0.0075663	7.5200E-21	0.002292	77.868	TRUE	FinnGen Release 10 broadly defined myocarditis
PD-1	UKB-PPP	rs3748022	1	206,496,132	T	C	0.2271	0.0431	0.00862267	5.6600E-07	0.000653	22.156	TRUE	FinnGen Release 10 broadly defined myocarditis
PD-1	UKB-PPP	rs3835195	11	320,115	A	G	0.8829	-0.0568	0.0117995	1.5000E-06	0.000666	22.606	TRUE	FinnGen Release 10 broadly defined myocarditis
PD-1	UKB-PPP	rs389436	22	17,811,756	A	T	0.3101	-0.0364	0.00790227	4.1800E-06	0.000566	19.198	TRUE	FinnGen Release 10 broadly defined myocarditis
PD-1	UKB-PPP	rs4686837	3	187,021,889	A	G	0.3125	-0.0419	0.00782698	8.8200E-08	0.000753	25.558	TRUE	FinnGen Release 10 broadly defined myocarditis
PD-1	UKB-PPP	rs4997754	18	48,786,185	T	C	0.1428	0.0478	0.010404	4.3200E-06	0.000560	18.985	TRUE	FinnGen Release 10 broadly defined myocarditis
PD-1	UKB-PPP	rs531968977	16	89,463,083	A	C	0.0377	-0.0962	0.0198729	1.3000E-06	0.000671	22.772	TRUE	FinnGen Release 10 broadly defined myocarditis
PD-1	UKB-PPP	rs6489785	12	120,925,921	C	T	0.6088	-0.0491	0.0074415	3.9800E-11	0.001151	39.051	TRUE	FinnGen Release 10 broadly defined myocarditis
PD-1	UKB-PPP	rs6791438	3	170,031,236	G	A	0.3656	-0.0536	0.00763585	2.3200E-12	0.001331	45.168	TRUE	FinnGen Release 10 broadly defined myocarditis
PD-1	UKB-PPP	rs6871748	5	35,885,880	C	T	0.2733	-0.0514	0.00817878	3.2900E-10	0.001049	35.611	TRUE	FinnGen Release 10 broadly defined myocarditis
PD-1	UKB-PPP	rs6908626	6	90,296,024	T	G	0.1812	0.0494	0.0094724	1.8800E-07	0.000723	24.524	TRUE	FinnGen Release 10 broadly defined myocarditis
PD-1	UKB-PPP	rs7161799	15	58,478,324	T	C	0.0765	0.1289	0.0137268	5.8400E-21	0.002350	79.856	TRUE	FinnGen Release 10 broadly defined myocarditis
PD-1	UKB-PPP	rs73119137	2	242,062,249	T	C	0.0109	0.2022	0.0356259	1.3800E-08	0.000880	29.872	TRUE	FinnGen Release 10 broadly defined myocarditis
PD-1	UKB-PPP	rs7519225	1	198,313,778	T	C	0.1298	0.0553	0.0108739	3.6600E-07	0.000691	23.447	TRUE	FinnGen Release 10 broadly defined myocarditis
PD-1	UKB-PPP	rs7528810	1	37,643,929	T	G	0.6087	0.0343	0.00750539	4.7800E-06	0.000561	19.045	TRUE	FinnGen Release 10 broadly defined myocarditis
PD-1	UKB-PPP	rs75960776	2	241,862,555	C	T	0.1606	-0.3050	0.0101508	2.7400E-198	0.025077	871.976	TRUE	FinnGen Release 10 broadly defined myocarditis
PD-1	UKB-PPP	rs76428106	13	28,029,870	C	T	0.0129	0.2089	0.0334641	4.3000E-10	0.001108	37.597	TRUE	FinnGen Release 10 broadly defined myocarditis
PD-1	UKB-PPP	rs7853407	9	1,868,131	T	C	0.1061	0.0587	0.0118249	6.8600E-07	0.000654	22.175	TRUE	FinnGen Release 10 broadly defined myocarditis

Protein	Source	SNP	Chromosome	Position	Effect allele	Other allele	Effect-allele frequency	Beta (exposure)	SE (exposure)	P value (exposure)	R^2	F statistic	F > 10	Primary outcome
PD-1	UKB-PPP	rs80224494	5	138,246,693	T	C	0.0205	-0.1311	0.0255282	2.8400E-07	0.000691	23.448	TRUE	FinnGen Release 10 broadly defined myocarditis
PD-1	UKB-PPP	rs80504	22	42,275,561	C	T	0.7523	0.0417	0.0083886	6.7900E-07	0.000647	21.953	TRUE	FinnGen Release 10 broadly defined myocarditis
PD-1	UKB-PPP	rs9292	11	10,506,936	G	A	0.9872	0.1487	0.0323585	4.3000E-06	0.000558	18.928	TRUE	FinnGen Release 10 broadly defined myocarditis
PD-1	UKB-PPP	rs9494336	6	135,694,749	T	C	0.3130	0.0370	0.00784045	2.3600E-06	0.000589	19.979	TRUE	FinnGen Release 10 broadly defined myocarditis
PD-1	UKB-PPP	rs9683415	4	40,290,296	G	A	0.6660	-0.0361	0.00775396	3.2400E-06	0.000580	19.660	TRUE	FinnGen Release 10 broadly defined myocarditis
PD-1	UKB-PPP	rs9929994	16	11,084,388	G	A	0.3574	-0.0380	0.00755761	4.8300E-07	0.000665	22.543	TRUE	FinnGen Release 10 broadly defined myocarditis
PD-1	INTERVAL	rs10444703					0.1408	0.1717	0.0372	3.8019E-06		23.696	TRUE	FinnGen Release 10 broadly defined myocarditis
PD-1	INTERVAL	rs111808226					0.0104	-0.6272	0.136	3.9811E-06		26.827	TRUE	FinnGen Release 10 broadly defined myocarditis
PD-1	INTERVAL	rs117444695					0.0131	0.5472	0.1115	9.3325E-07		25.663	TRUE	FinnGen Release 10 broadly defined myocarditis
PD-1	INTERVAL	rs12073392					0.8127	-0.1455	0.0314	3.7153E-06		21.398	TRUE	FinnGen Release 10 broadly defined myocarditis
PD-1	INTERVAL	rs147652769					0.0174	0.5534	0.1068	2.1878E-07		34.853	TRUE	FinnGen Release 10 broadly defined myocarditis
PD-1	INTERVAL	rs34777990					0.2661	-0.1367	0.0299	4.8978E-06		24.255	TRUE	FinnGen Release 10 broadly defined myocarditis
PD-1	INTERVAL	rs5030928					0.2621	0.1509	0.0293	2.6915E-07		29.313	TRUE	FinnGen Release 10 broadly defined myocarditis
PD-1	INTERVAL	rs55720497					0.0226	-0.4143	0.0832	6.3096E-07		25.185	TRUE	FinnGen Release 10 broadly defined myocarditis
PD-1	INTERVAL	rs5757973					0.8903	0.3788	0.0399	1.9953E-21		95.147	TRUE	FinnGen Release 10 broadly defined myocarditis
PD-1	INTERVAL	rs71362039					0.0384	0.3356	0.0663	4.1687E-07		27.656	TRUE	FinnGen Release 10 broadly defined myocarditis
PD-1	INTERVAL	rs72692455					0.3194	0.1246	0.027	3.9811E-06		22.421	TRUE	FinnGen Release 10 broadly defined myocarditis
PD-1	INTERVAL	rs77326121					0.0345	0.3256	0.0682	1.8197E-06		23.446	TRUE	FinnGen Release 10 broadly defined myocarditis
PD-1	INTERVAL	rs9958590					0.2966	0.1239	0.0271	4.8978E-06		21.266	TRUE	FinnGen Release 10 broadly defined myocarditis
PD-L1	INTERVAL	rs11011804					0.0353	0.3091	0.0671	4.0738E-06		21.608	TRUE	FinnGen Release 10 broadly defined myocarditis
PD-L1	INTERVAL	rs13322229					0.1322	0.1742	0.0369	2.3988E-06		23.131	TRUE	FinnGen Release 10 broadly defined myocarditis
PD-L1	INTERVAL	rs140094912					0.0354	-0.3337	0.0719	3.5481E-06		25.281	TRUE	FinnGen Release 10 broadly defined myocarditis
PD-L1	INTERVAL	rs822341					0.7347	0.1731	0.0278	4.4668E-10		38.990	TRUE	FinnGen Release 10 broadly defined myocarditis
PD-L1	INTERVAL	rs9859911					0.9012	0.1981	0.0417	2.0893E-06		23.217	TRUE	FinnGen Release 10 broadly defined myocarditis

Note: Protein labels were verified using the exposure identifiers: CD274 = PD-L1 and PDCD1 = PD-1. All listed instruments had F > 10. Abbreviations: F, F statistic; PD-1, programmed cell death protein 1; PD-L1, programmed death-ligand 1; SE, standard error; SNP, single-nucleotide polymorphism; UKB-PPP, UK Biobank Pharma Proteomics Project.
