# Supplementary material for: Genetically proxied circulating PD-1/PD-L1 levels and broadly defined myocarditis: A bidirectional Mendelian randomization study with exploratory lipidomic analyses
Source: Medicine (Baltimore). 2026 Aug 28;105(35):e50475. doi: 10.1097/MD.0000000000050475 (PMC13529170; doi:10.1097/MD.0000000000050475)
Supplement: Supplementary file 2 [file medi-105-e50475-s002.pdf]

Supplementary Table 2. FinnGen Release 10 myocarditis instruments used in reverse Mendelian randomization.

Outcome	SNP	Chromosome	Position	Effect allele	Other allele	Effect-allele frequency	Beta	SE	P value	Sample size	F statistic	F > 10
FinnGen Release 10 broadly defined myocarditis	rs56172343	1	29,012,121	C	G	0.0321	0.4368	0.0864	4.2635E-07	212,306	25.571	TRUE
FinnGen Release 10 broadly defined myocarditis	rs112136957	6	32,714,388	C	T	0.0105	0.7118	0.1407	4.1809E-07	212,306	25.609	TRUE
FinnGen Release 10 broadly defined myocarditis	rs56196802	7	18,834,248	T	C	0.0011	1.5756	0.3289	1.6671E-06	212,306	22.945	TRUE
FinnGen Release 10 broadly defined myocarditis	rs1886854	13	95,643,882	A	G	0.0806	0.2762	0.0603	4.6968E-06	212,306	20.957	TRUE
FinnGen Release 10 broadly defined myocarditis	rs12709211	16	8,025,269	G	T	0.4228	0.1842	0.0351	1.5874E-07	212,306	27.480	TRUE
FinnGen Release 10 broadly defined myocarditis	rs34255015	10	52,436,957	T	C	0.4139	0.1826	0.0354	2.4428E-07	212,306	26.647	TRUE
FinnGen Release 10 broadly defined myocarditis	rs3783113	13	110,182,399	T	C	0.3427	0.1683	0.0364	3.6788E-06	212,306	21.425	TRUE
FinnGen Release 10 broadly defined myocarditis	rs78045619	10	132,112,047	A	G	0.0111	0.7411	0.1328	2.4286E-08	212,306	31.118	TRUE
FinnGen Release 10 broadly defined myocarditis	rs2088236	12	116,787,273	T	C	0.5941	0.1736	0.0365	1.9543E-06	212,306	22.639	TRUE
FinnGen Release 10 broadly defined myocarditis	rs147708194	16	385,513	G	C	0.0296	-0.5659	0.1235	4.5684E-06	212,306	21.010	TRUE

Note: F statistics were calculated as beta squared divided by SE squared. All listed instruments had F > 10. Abbreviations: F, F statistic; MR, Mendelian randomization; SE, standard error; SNP, single-nucleotide polymorphism.
