# Supplementary material for: Genetically proxied circulating PD-1/PD-L1 levels and broadly defined myocarditis: A bidirectional Mendelian randomization study with exploratory lipidomic analyses
Source: Medicine (Baltimore). 2026 Aug 28;105(35):e50475. doi: 10.1097/MD.0000000000050475 (PMC13529170; doi:10.1097/MD.0000000000050475)
Supplement: Supplementary file 3 [file medi-105-e50475-s003.pdf]

Supplementary Table 3. Instrument-level data for the 179 plasma lipid species.

Lipid GWAS ID	Lipid species	SNP	Chromosome	Position	Effect allele	Other allele	Effect-allele frequency	Beta	SE	P value	Sample size	F statistic	F > 10
ebi-PL179-GCST90277253	Ceramide (d40:1) levels	rs6535831	4	151,996,582	T	G	0.8609	0.1143	0.0244	2.8476E-06	7,117	21.952	TRUE
ebi-PL179-GCST90277253	Ceramide (d40:1) levels	rs114910162	2	141,867,363	T	C	0.0495	0.1953	0.0399	9.7409E-07	7,117	24.018	TRUE
ebi-PL179-GCST90277253	Ceramide (d40:1) levels	rs7157785	14	63,768,838	T	G	0.1255	0.1476	0.0252	5.0971E-09	7,117	34.236	TRUE
ebi-PL179-GCST90277253	Ceramide (d40:1) levels	rs293566	20	32,510,074	C	T	0.4668	-0.0853	0.0170	5.5773E-07	7,117	25.097	TRUE
ebi-PL179-GCST90277253	Ceramide (d40:1) levels	rs867212	22	46,667,433	G	A	0.2218	-0.0983	0.0203	1.2565E-06	7,117	23.527	TRUE
ebi-PL179-GCST90277253	Ceramide (d40:1) levels	rs28505920	8	118,268,727	A	T	0.1714	0.1233	0.0223	3.5753E-08	7,117	30.432	TRUE
ebi-PL179-GCST90277253	Ceramide (d40:1) levels	rs56143221	10	73,731,455	A	G	0.1442	-0.1111	0.0241	4.1374E-06	7,117	21.231	TRUE
ebi-PL179-GCST90277253	Ceramide (d40:1) levels	rs12927793	16	89,171,782	A	G	0.5300	-0.0792	0.0167	2.1276E-06	7,117	22.513	TRUE
ebi-PL179-GCST90277253	Ceramide (d40:1) levels	rs1260326	2	27,508,073	C	T	0.6509	-0.0995	0.0175	1.4003E-08	7,117	32.260	TRUE
ebi-PL179-GCST90277253	Ceramide (d40:1) levels	rs1346910	9	71,460,729	T	C	0.8229	-0.1025	0.0221	3.4564E-06	7,117	21.578	TRUE
ebi-PL179-GCST90277253	Ceramide (d40:1) levels	rs117873718	12	67,485,111	C	T	0.0326	-0.2229	0.0483	4.0327E-06	7,117	21.281	TRUE
ebi-PL179-GCST90277253	Ceramide (d40:1) levels	rs58489806	19	19,346,108	T	C	0.0757	-0.1785	0.0316	1.7162E-08	7,117	31.859	TRUE
ebi-PL179-GCST90277253	Ceramide (d40:1) levels	rs12366015	11	117,120,135	G	A	0.7168	-0.0881	0.0186	2.2529E-06	7,117	22.399	TRUE
ebi-PL179-GCST90277253	Ceramide (d40:1) levels	rs2236124	20	13,126,078	C	T	0.1464	0.1277	0.0235	5.6019E-08	7,117	29.557	TRUE
ebi-PL179-GCST90277253	Ceramide (d40:1) levels	rs364585	20	12,982,070	G	A	0.6614	-0.2633	0.0174	1.0184E-50	7,117	227.920	TRUE
ebi-PL179-GCST90277253	Ceramide (d40:1) levels	rs56406916	20	13,295,543	A	G	0.0571	0.1730	0.0375	3.9556E-06	7,117	21.318	TRUE
ebi-PL179-GCST90277253	Ceramide (d40:1) levels	rs11982486	7	95,426,286	C	T	0.2405	0.0894	0.0195	4.7664E-06	7,117	20.960	TRUE
ebi-PL179-GCST90277253	Ceramide (d40:1) levels	rs187429064	19	19,269,704	G	A	0.0535	-0.2320	0.0379	1.0244E-09	7,117	37.373	TRUE
ebi-PL179-GCST90277254	Ceramide (d40:2) levels	rs61834063	1	240,877,505	G	A	0.2091	-0.1129	0.0238	2.0967E-06	5,287	22.554	TRUE
ebi-PL179-GCST90277254	Ceramide (d40:2) levels	rs12878001	14	63,772,911	G	T	0.1241	0.2160	0.0287	5.6663E-14	5,287	56.773	TRUE
ebi-PL179-GCST90277254	Ceramide (d40:2) levels	rs111745969	15	92,566,992	A	G	0.1348	-0.1349	0.0287	2.5870E-06	5,287	22.146	TRUE
ebi-PL179-GCST90277254	Ceramide (d40:2) levels	rs12974674	19	38,559,208	C	G	0.2405	0.1057	0.0224	2.5449E-06	5,287	22.174	TRUE
ebi-PL179-GCST90277254	Ceramide (d40:2) levels	rs147060788	2	240,329,826	C	T	0.0341	0.2487	0.0533	3.0758E-06	5,287	21.811	TRUE
ebi-PL179-GCST90277254	Ceramide (d40:2) levels	rs61751862	19	48,629,610	C	G	0.0305	0.2500	0.0540	3.7499E-06	5,287	21.429	TRUE
ebi-PL179-GCST90277254	Ceramide (d40:2) levels	rs17748108	13	76,834,291	C	T	0.1228	0.1446	0.0302	1.7207E-06	5,287	22.939	TRUE
ebi-PL179-GCST90277254	Ceramide (d40:2) levels	rs438568	20	12,978,039	G	A	0.6658	-0.2277	0.0202	3.6565E-29	5,287	127.150	TRUE
ebi-PL179-GCST90277254	Ceramide (d40:2) levels	rs115833524	2	167,928,158	T	C	0.0418	0.2311	0.0494	2.9188E-06	5,287	21.910	TRUE
ebi-PL179-GCST90277254	Ceramide (d40:2) levels	rs139456652	11	6,300,702	A	G	0.0588	0.2136	0.0432	8.0052E-07	5,287	24.414	TRUE
ebi-PL179-GCST90277254	Ceramide (d40:2) levels	rs964184	11	116,778,201	C	G	0.8490	-0.1400	0.0267	1.6424E-07	5,287	27.483	TRUE
ebi-PL179-GCST90277254	Ceramide (d40:2) levels	rs6559102	8	6,166,747	A	G	0.0131	-0.4183	0.0881	2.1195E-06	5,287	22.535	TRUE
ebi-PL179-GCST90277254	Ceramide (d40:2) levels	rs117671667	11	36,075,190	T	C	0.0341	0.2675	0.0571	2.8260E-06	5,287	21.972	TRUE
ebi-PL179-GCST90277255	Ceramide (d42:1) levels	rs78573518	2	200,379,367	G	A	0.0068	-0.5001	0.1076	3.3768E-06	7,174	21.623	TRUE
ebi-PL179-GCST90277255	Ceramide (d42:1) levels	rs114822153	4	72,372,827	A	G	0.0296	0.2374	0.0510	3.2122E-06	7,174	21.717	TRUE
ebi-PL179-GCST90277255	Ceramide (d42:1) levels	rs6977389	7	95,390,043	A	G	0.5603	-0.0778	0.0169	4.1034E-06	7,174	21.249	TRUE
ebi-PL179-GCST90277255	Ceramide (d42:1) levels	rs56368930	14	33,070,106	C	G	0.0124	0.3797	0.0827	4.4415E-06	7,174	21.096	TRUE
ebi-PL179-GCST90277255	Ceramide (d42:1) levels	rs11105228	12	89,259,912	A	G	0.3371	-0.0860	0.0176	1.1250E-06	7,174	23.742	TRUE
ebi-PL179-GCST90277255	Ceramide (d42:1) levels	rs10033592	4	40,237,784	G	T	0.6553	-0.0821	0.0177	3.3592E-06	7,174	21.632	TRUE
ebi-PL179-GCST90277255	Ceramide (d42:1) levels	rs186176314	5	16,573,000	C	T	0.0225	0.2693	0.0577	3.1319E-06	7,174	21.767	TRUE
ebi-PL179-GCST90277255	Ceramide (d42:1) levels	rs2945048	19	38,556,464	A	T	0.2408	0.0926	0.0194	1.8782E-06	7,174	22.753	TRUE
ebi-PL179-GCST90277255	Ceramide (d42:1) levels	rs7123454	11	116,833,462	A	C	0.8115	-0.1110	0.0216	2.8681E-07	7,174	26.384	TRUE
ebi-PL179-GCST90277255	Ceramide (d42:1) levels	rs8100204	19	19,282,905	A	G	0.1707	-0.1661	0.0224	1.2941E-13	7,174	55.063	TRUE
ebi-PL179-GCST90277255	Ceramide (d42:1) levels	rs117888980	9	107,021,609	G	T	0.0186	-0.2944	0.0636	3.7397E-06	7,174	21.426	TRUE
ebi-PL179-GCST90277256	Ceramide (d42:2) levels	rs6503695	17	42,347,515	C	T	0.3140	0.0838	0.0180	3.2764E-06	7,173	21.680	TRUE
ebi-PL179-GCST90277256	Ceramide (d42:2) levels	rs12356523	10	130,351,351	A	G	0.1906	0.0980	0.0211	3.3149E-06	7,173	21.659	TRUE
ebi-PL179-GCST90277256	Ceramide (d42:2) levels	rs5141	11	116,831,407	C	T	0.8637	-0.1248	0.0246	3.8671E-07	7,173	25.805	TRUE
ebi-PL179-GCST90277256	Ceramide (d42:2) levels	rs1042034	2	21,002,409	T	C	0.7276	0.1026	0.0186	3.5362E-08	7,173	30.454	TRUE
ebi-PL179-GCST90277256	Ceramide (d42:2) levels	rs142985829	3	102,651,475	T	C	0.0121	0.3622	0.0791	4.7875E-06	7,173	20.951	TRUE
ebi-PL179-GCST90277256	Ceramide (d42:2) levels	rs55817533	2	120,863,835	C	A	0.1156	0.1301	0.0262	7.0464E-07	7,173	24.645	TRUE
ebi-PL179-GCST90277256	Ceramide (d42:2) levels	rs79098941	16	52,635,159	C	T	0.0239	0.2639	0.0559	2.3885E-06	7,173	22.288	TRUE
ebi-PL179-GCST90277256	Ceramide (d42:2) levels	rs117704733	17	37,499,294	T	C	0.0876	0.1366	0.0297	4.3864E-06	7,173	21.119	TRUE
ebi-PL179-GCST90277256	Ceramide (d42:2) levels	rs116327692	2	137,364,592	T	C	0.0143	0.3466	0.0715	1.2782E-06	7,173	23.492	TRUE
ebi-PL179-GCST90277256	Ceramide (d42:2) levels	rs1169306	12	121,000,508	T	C	0.3596	-0.0983	0.0174	1.5140E-08	7,173	32.106	TRUE
ebi-PL179-GCST90277256	Ceramide (d42:2) levels	rs10761716	10	63,122,540	G	C	0.4611	-0.0786	0.0167	2.5498E-06	7,173	22.163	TRUE
ebi-PL179-GCST90277256	Ceramide (d42:2) levels	rs779714	3	7,468,297	T	C	0.8701	0.1207	0.0262	4.0316E-06	7,173	21.283	TRUE
ebi-PL179-GCST90277256	Ceramide (d42:2) levels	rs11713732	3	54,513,590	T	C	0.1602	0.1123	0.0237	2.1201E-06	7,173	22.520	TRUE
ebi-PL179-GCST90277256	Ceramide (d42:2) levels	rs1572595	10	8,406,642	T	C	0.3821	-0.0838	0.0173	1.2353E-06	7,173	23.560	TRUE
ebi-PL179-GCST90277256	Ceramide (d42:2) levels	rs12763964	10	70,843,134	C	T	0.2057	-0.1320	0.0207	1.8820E-10	7,173	40.705	TRUE
ebi-PL179-GCST90277256	Ceramide (d42:2) levels	rs78709083	21	28,580,399	T	C	0.0392	0.2041	0.0438	3.1866E-06	7,173	21.734	TRUE

Lipid GWAS ID	Lipid species	SNP	Chromosome	Position	Effect allele	Other allele	Effect-allele frequency	Beta	SE	P value	Sample size	F statistic	F > 10
ebi-PL179-GCST90277256	Ceramide (d42:2) levels	rs5767288	22	46,600,357	A	G	0.1903	-0.1414	0.0214	4.3075E-11	7,173	43.598	TRUE
ebi-PL179-GCST90277256	Ceramide (d42:2) levels	rs72999033	19	19,255,823	T	C	0.0624	-0.1992	0.0344	7.1197E-09	7,173	33.581	TRUE
ebi-PL179-GCST90277257	Cholesterol levels	rs11730766	4	72,366,511	T	C	0.0287	0.2529	0.0516	9.6734E-07	7,166	24.033	TRUE
ebi-PL179-GCST90277257	Cholesterol levels	rs182695896	4	73,947,510	C	A	0.0253	0.2682	0.0533	5.0217E-07	7,166	25.300	TRUE
ebi-PL179-GCST90277257	Cholesterol levels	rs180958041	2	170,149,397	T	C	0.0124	-0.3653	0.0771	2.2171E-06	7,166	22.433	TRUE
ebi-PL179-GCST90277257	Cholesterol levels	rs2237478	7	50,678,097	T	C	0.1985	-0.0993	0.0211	2.5160E-06	7,166	22.190	TRUE
ebi-PL179-GCST90277257	Cholesterol levels	rs10812660	9	27,742,537	A	T	0.3751	0.0860	0.0179	1.5675E-06	7,166	23.100	TRUE
ebi-PL179-GCST90277257	Cholesterol levels	rs78721266	9	96,213,656	G	A	0.0440	0.1908	0.0413	3.9038E-06	7,166	21.349	TRUE
ebi-PL179-GCST90277257	Cholesterol levels	rs429358	19	44,908,684	C	T	0.1892	0.1056	0.0215	9.2638E-07	7,166	24.116	TRUE
ebi-PL179-GCST90277257	Cholesterol levels	rs112411704	9	2,958,367	G	A	0.0175	0.3032	0.0660	4.5115E-06	7,166	21.068	TRUE
ebi-PL179-GCST90277257	Cholesterol levels	rs12562141	1	181,952,433	C	T	0.0832	-0.1392	0.0303	4.5242E-06	7,166	21.062	TRUE
ebi-PL179-GCST90277257	Cholesterol levels	rs1271671	12	31,810,895	A	G	0.0279	0.2352	0.0513	4.6983E-06	7,166	20.993	TRUE
ebi-PL179-GCST90277257	Cholesterol levels	rs11857386	15	58,428,969	C	G	0.4711	-0.0794	0.0173	4.5688E-06	7,166	21.041	TRUE
ebi-PL179-GCST90277257	Cholesterol levels	rs118068660	19	11,079,868	T	C	0.0946	-0.1614	0.0283	1.1609E-08	7,166	32.625	TRUE
ebi-PL179-GCST90277257	Cholesterol levels	rs114243848	19	49,823,108	C	A	0.0423	-0.2278	0.0422	7.1656E-08	7,166	29.079	TRUE
ebi-PL179-GCST90277257	Cholesterol levels	rs12552499	9	121,135,217	G	A	0.0474	-0.1888	0.0401	2.5017E-06	7,166	22.199	TRUE
ebi-PL179-GCST90277257	Cholesterol levels	rs16963800	16	14,576,910	C	T	0.1602	0.1103	0.0228	1.3562E-06	7,166	23.380	TRUE
ebi-PL179-GCST90277257	Cholesterol levels	rs73005445	19	15,830,645	G	A	0.2534	-0.0900	0.0192	2.9391E-06	7,166	21.890	TRUE
ebi-PL179-GCST90277257	Cholesterol levels	rs669552	3	172,335,874	G	A	0.1560	-0.1082	0.0233	3.3989E-06	7,166	21.606	TRUE
ebi-PL179-GCST90277257	Cholesterol levels	rs11858279	15	58,420,774	C	T	0.3026	0.0877	0.0182	1.4326E-06	7,166	23.274	TRUE
ebi-PL179-GCST90277257	Cholesterol levels	rs57405165	20	40,665,923	T	C	0.1549	-0.1069	0.0230	3.5246E-06	7,166	21.542	TRUE
ebi-PL179-GCST90277257	Cholesterol levels	rs2879630	10	51,918,584	A	G	0.1890	-0.1012	0.0212	1.8010E-06	7,166	22.833	TRUE
ebi-PL179-GCST90277257	Cholesterol levels	rs1800961	20	44,413,724	T	C	0.0524	-0.1796	0.0374	1.5708E-06	7,166	23.096	TRUE
ebi-PL179-GCST90277258	Diacylglycerol (16:0_18:1) levels	rs72753379	9	120,350,676	T	C	0.0392	0.2315	0.0477	1.2761E-06	6,293	23.501	TRUE
ebi-PL179-GCST90277258	Diacylglycerol (16:0_18:1) levels	rs118099052	12	44,462,630	T	A	0.0146	0.3787	0.0763	7.2173E-07	6,293	24.602	TRUE
ebi-PL179-GCST90277258	Diacylglycerol (16:0_18:1) levels	rs117614422	15	40,252,330	G	C	0.0650	-0.1735	0.0379	4.7040E-06	6,293	20.989	TRUE
ebi-PL179-GCST90277258	Diacylglycerol (16:0_18:1) levels	rs11775636	8	142,752,432	A	G	0.1575	-0.1220	0.0242	4.5969E-07	6,293	25.478	TRUE
ebi-PL179-GCST90277258	Diacylglycerol (16:0_18:1) levels	rs55857911	1	230,598,232	T	C	0.0925	0.1484	0.0320	3.6608E-06	6,293	21.471	TRUE
ebi-PL179-GCST90277258	Diacylglycerol (16:0_18:1) levels	rs1884216	14	36,679,967	A	C	0.6226	-0.0848	0.0184	4.2921E-06	6,293	21.165	TRUE
ebi-PL179-GCST90277258	Diacylglycerol (16:0_18:1) levels	rs36094040	12	72,431,183	C	T	0.3482	-0.0871	0.0190	4.6383E-06	6,293	21.016	TRUE
ebi-PL179-GCST90277258	Diacylglycerol (16:0_18:1) levels	rs180999317	7	157,355,450	A	G	0.0047	-0.6535	0.1375	2.0383E-06	6,293	22.598	TRUE
ebi-PL179-GCST90277259	Diacylglycerol (16:0_18:2) levels	rs34606692	5	149,755,706	T	C	0.1963	-0.1138	0.0230	7.3775E-07	6,075	24.565	TRUE
ebi-PL179-GCST90277259	Diacylglycerol (16:0_18:2) levels	rs7165355	15	38,675,336	C	G	0.1925	-0.1073	0.0232	3.9177E-06	6,075	21.342	TRUE
ebi-PL179-GCST90277259	Diacylglycerol (16:0_18:2) levels	rs57747810	12	19,924,761	A	C	0.0117	-0.4042	0.0844	1.7001E-06	6,075	22.950	TRUE
ebi-PL179-GCST90277259	Diacylglycerol (16:0_18:2) levels	rs6687291	1	230,586,281	A	G	0.3199	0.0981	0.0198	7.5383E-07	6,075	24.522	TRUE
ebi-PL179-GCST90277259	Diacylglycerol (16:0_18:2) levels	rs78504564	2	42,953,102	T	C	0.0049	-0.6302	0.1337	2.4885E-06	6,075	22.216	TRUE
ebi-PL179-GCST90277259	Diacylglycerol (16:0_18:2) levels	rs17145750	7	73,612,048	T	C	0.1407	-0.1339	0.0261	3.1352E-07	6,075	26.221	TRUE
ebi-PL179-GCST90277259	Diacylglycerol (16:0_18:2) levels	rs758398	6	28,352,897	T	C	0.1830	-0.1162	0.0234	7.4185E-07	6,075	24.552	TRUE
ebi-PL179-GCST90277259	Diacylglycerol (16:0_18:2) levels	rs2004776	1	230,712,956	T	C	0.1965	-0.1075	0.0229	2.8338E-06	6,075	21.966	TRUE
ebi-PL179-GCST90277259	Diacylglycerol (16:0_18:2) levels	rs112122274	9	136,257,994	G	A	0.0360	-0.2528	0.0527	1.6795E-06	6,075	22.972	TRUE
ebi-PL179-GCST90277259	Diacylglycerol (16:0_18:2) levels	rs11182480	12	44,386,579	C	T	0.0178	0.3407	0.0712	1.7761E-06	6,075	22.865	TRUE
ebi-PL179-GCST90277259	Diacylglycerol (16:0_18:2) levels	rs6810359	3	188,792,426	T	G	0.0528	-0.1924	0.0415	3.6124E-06	6,075	21.499	TRUE
ebi-PL179-GCST90277259	Diacylglycerol (16:0_18:2) levels	rs4806901	19	3,057,222	C	T	0.1505	0.1214	0.0264	4.1842E-06	6,075	21.218	TRUE
ebi-PL179-GCST90277259	Diacylglycerol (16:0_18:2) levels	rs35986054	17	1,623,049	C	T	0.1962	0.1068	0.0232	4.2997E-06	6,075	21.163	TRUE
ebi-PL179-GCST90277259	Diacylglycerol (16:0_18:2) levels	rs9344829	6	64,177,173	T	C	0.3732	0.0896	0.0188	1.8221E-06	6,075	22.817	TRUE
ebi-PL179-GCST90277259	Diacylglycerol (16:0_18:2) levels	rs140314539	2	220,215,369	A	G	0.0214	0.3084	0.0638	1.3775E-06	6,075	23.356	TRUE
ebi-PL179-GCST90277259	Diacylglycerol (16:0_18:2) levels	rs148163011	9	71,279,650	G	C	0.0242	-0.2813	0.0600	2.8251E-06	6,075	21.972	TRUE
ebi-PL179-GCST90277259	Diacylglycerol (16:0_18:2) levels	rs111934503	8	130,543,708	C	T	0.0046	0.6693	0.1396	1.6621E-06	6,075	22.994	TRUE
ebi-PL179-GCST90277259	Diacylglycerol (16:0_18:2) levels	rs144838649	21	44,845,081	T	C	0.0047	-0.5803	0.1250	3.5158E-06	6,075	21.551	TRUE
ebi-PL179-GCST90277260	Diacylglycerol (16:1_18:1) levels	rs77134026	7	36,495,127	A	G	0.0306	0.2580	0.0553	3.2016E-06	6,215	21.731	TRUE
ebi-PL179-GCST90277260	Diacylglycerol (16:1_18:1) levels	rs140283718	19	30,685,856	A	T	0.0257	-0.2659	0.0571	3.2890E-06	6,215	21.679	TRUE
ebi-PL179-GCST90277260	Diacylglycerol (16:1_18:1) levels	rs2524075	6	31,276,082	T	C	0.2080	-0.1072	0.0218	9.2197E-07	6,215	24.132	TRUE
ebi-PL179-GCST90277260	Diacylglycerol (16:1_18:1) levels	rs111750990	12	96,298,692	C	A	0.0414	-0.2143	0.0456	2.6533E-06	6,215	22.091	TRUE
ebi-PL179-GCST90277260	Diacylglycerol (16:1_18:1) levels	rs7910473	10	62,379,823	T	C	0.9773	0.2848	0.0610	3.1279E-06	6,215	21.775	TRUE
ebi-PL179-GCST90277260	Diacylglycerol (16:1_18:1) levels	rs7955732	12	72,331,215	T	G	0.4229	-0.0886	0.0183	1.2441E-06	6,215	23.552	TRUE
ebi-PL179-GCST90277260	Diacylglycerol (16:1_18:1) levels	rs2182380	6	43,900,875	G	A	0.0306	0.2607	0.0544	1.6494E-06	6,215	23.008	TRUE
ebi-PL179-GCST90277260	Diacylglycerol (16:1_18:1) levels	rs10502551	18	30,887,893	G	C	0.0509	-0.2128	0.0416	3.2136E-07	6,215	26.174	TRUE
ebi-PL179-GCST90277261	Diacylglycerol (18:1_18:1) levels	rs268	8	19,956,018	G	A	0.0227	0.2815	0.0580	1.2597E-06	6,884	23.525	TRUE

Lipid GWAS ID	Lipid species	SNP	Chromosome	Position	Effect allele	Other allele	Effect-allele frequency	Beta	SE	P value	Sample size	F statistic	F > 10
ebi-PL179-GCST90277261	Diacylglycerol (18:1_18:1) levels	rs10169217	2	212,010,621	G	A	0.6760	-0.0983	0.0182	7.1819E-08	6,884	29.076	TRUE
ebi-PL179-GCST90277261	Diacylglycerol (18:1_18:1) levels	rs9865586	3	132,015,999	G	A	0.1650	-0.1068	0.0226	2.3340E-06	6,884	22.334	TRUE
ebi-PL179-GCST90277261	Diacylglycerol (18:1_18:1) levels	rs139500046	12	96,850,670	C	A	0.0112	-0.3929	0.0807	1.1337E-06	6,884	23.727	TRUE
ebi-PL179-GCST90277261	Diacylglycerol (18:1_18:1) levels	rs116011373	3	87,298,641	A	G	0.0488	-0.1861	0.0398	3.0554E-06	6,884	21.816	TRUE
ebi-PL179-GCST90277261	Diacylglycerol (18:1_18:1) levels	rs116796527	4	62,344,980	T	C	0.0143	-0.3373	0.0718	2.6808E-06	6,884	22.068	TRUE
ebi-PL179-GCST90277261	Diacylglycerol (18:1_18:1) levels	rs147782497	18	78,352,143	A	C	0.0242	0.2585	0.0565	4.7478E-06	6,884	20.968	TRUE
ebi-PL179-GCST90277261	Diacylglycerol (18:1_18:1) levels	rs62046299	16	82,009,735	C	T	0.0165	0.3639	0.0722	4.7819E-07	6,884	25.397	TRUE
ebi-PL179-GCST90277261	Diacylglycerol (18:1_18:1) levels	rs3770813	2	36,549,412	T	G	0.0277	-0.2459	0.0521	2.3693E-06	6,884	22.305	TRUE
ebi-PL179-GCST90277261	Diacylglycerol (18:1_18:1) levels	rs7782961	7	6,295,952	T	A	0.2319	0.0946	0.0204	3.5090E-06	6,884	21.550	TRUE
ebi-PL179-GCST90277261	Diacylglycerol (18:1_18:1) levels	rs17596144	1	220,805,303	T	C	0.1517	0.1310	0.0239	4.2595E-08	6,884	30.093	TRUE
ebi-PL179-GCST90277261	Diacylglycerol (18:1_18:1) levels	rs76950187	2	128,427,075	G	A	0.0745	0.1597	0.0326	9.5849E-07	6,884	24.051	TRUE
ebi-PL179-GCST90277261	Diacylglycerol (18:1_18:1) levels	rs62291573	4	4,999,649	C	T	0.2144	0.1067	0.0215	6.9090E-07	6,884	24.684	TRUE
ebi-PL179-GCST90277261	Diacylglycerol (18:1_18:1) levels	rs35332062	7	73,597,712	A	G	0.1220	-0.1438	0.0259	3.1125E-08	6,884	30.704	TRUE
ebi-PL179-GCST90277261	Diacylglycerol (18:1_18:1) levels	rs157582	19	44,892,962	T	C	0.2233	0.0944	0.0205	4.4334E-06	6,884	21.100	TRUE
ebi-PL179-GCST90277262	Diacylglycerol (18:1_18:2) levels	rs55788915	16	23,267,056	A	C	0.0310	-0.2515	0.0528	1.9228E-06	6,613	22.710	TRUE
ebi-PL179-GCST90277262	Diacylglycerol (18:1_18:2) levels	rs11668758	19	44,971,423	T	C	0.3066	0.0962	0.0191	4.5436E-07	6,613	25.497	TRUE
ebi-PL179-GCST90277262	Diacylglycerol (18:1_18:2) levels	rs10773708	12	129,796,667	A	G	0.6826	-0.0871	0.0188	3.7145E-06	6,613	21.442	TRUE
ebi-PL179-GCST90277262	Diacylglycerol (18:1_18:2) levels	rs1233660	6	28,292,472	A	T	0.2205	-0.0977	0.0209	3.0009E-06	6,613	21.852	TRUE
ebi-PL179-GCST90277262	Diacylglycerol (18:1_18:2) levels	rs75008187	6	64,441,454	A	G	0.1278	0.1288	0.0263	9.7992E-07	6,613	24.011	TRUE
ebi-PL179-GCST90277262	Diacylglycerol (18:1_18:2) levels	rs7239207	18	62,009,100	C	T	0.6941	-0.0862	0.0189	4.9906E-06	6,613	20.874	TRUE
ebi-PL179-GCST90277262	Diacylglycerol (18:1_18:2) levels	rs1874124	1	220,801,995	T	C	0.1519	0.1181	0.0244	1.3132E-06	6,613	23.445	TRUE
ebi-PL179-GCST90277262	Diacylglycerol (18:1_18:2) levels	rs4608457	19	20,106,360	T	C	0.0453	-0.1996	0.0418	1.8090E-06	6,613	22.825	TRUE
ebi-PL179-GCST90277262	Diacylglycerol (18:1_18:2) levels	rs12365864	11	116,618,319	G	A	0.1663	0.1235	0.0235	1.5815E-07	6,613	27.544	TRUE
ebi-PL179-GCST90277262	Diacylglycerol (18:1_18:2) levels	rs79624003	7	73,598,455	G	A	0.1220	-0.1723	0.0265	8.9798E-11	6,613	42.166	TRUE
ebi-PL179-GCST90277262	Diacylglycerol (18:1_18:2) levels	rs72694990	1	150,251,390	A	G	0.1287	-0.1205	0.0261	4.0015E-06	6,613	21.298	TRUE
ebi-PL179-GCST90277262	Diacylglycerol (18:1_18:2) levels	rs75596627	8	43,650,502	A	G	0.1140	0.1279	0.0279	4.6235E-06	6,613	21.020	TRUE
ebi-PL179-GCST90277262	Diacylglycerol (18:1_18:2) levels	rs117416257	10	5,793,091	T	C	0.0046	-0.6045	0.1307	3.8301E-06	6,613	21.384	TRUE
ebi-PL179-GCST90277262	Diacylglycerol (18:1_18:2) levels	rs17007519	4	141,672,748	T	C	0.0219	-0.3011	0.0604	6.2376E-07	6,613	24.882	TRUE
ebi-PL179-GCST90277262	Diacylglycerol (18:1_18:2) levels	rs72836628	10	112,142,696	T	C	0.2966	-0.0902	0.0190	2.1129E-06	6,613	22.529	TRUE
ebi-PL179-GCST90277262	Diacylglycerol (18:1_18:2) levels	rs11684166	2	111,060,064	A	G	0.1765	0.1063	0.0229	3.4906E-06	6,613	21.561	TRUE
ebi-PL179-GCST90277262	Diacylglycerol (18:1_18:2) levels	rs141116593	6	154,880,792	G	C	0.0740	0.1553	0.0331	2.8213E-06	6,613	21.971	TRUE
ebi-PL179-GCST90277262	Diacylglycerol (18:1_18:2) levels	rs2352723	1	230,156,121	C	T	0.7685	-0.1123	0.0204	3.7632E-08	6,613	30.336	TRUE
ebi-PL179-GCST90277263	Diacylglycerol (18:1_18:3) levels	rs10749053	10	110,816,937	C	T	0.8736	-0.1422	0.0276	2.5667E-07	6,572	26.605	TRUE
ebi-PL179-GCST90277263	Diacylglycerol (18:1_18:3) levels	rs6958772	7	10,662,626	C	G	0.2441	0.0956	0.0202	2.2678E-06	6,572	22.391	TRUE
ebi-PL179-GCST90277263	Diacylglycerol (18:1_18:3) levels	rs6583035	1	107,643,461	C	T	0.1619	-0.1116	0.0237	2.4618E-06	6,572	22.233	TRUE
ebi-PL179-GCST90277263	Diacylglycerol (18:1_18:3) levels	rs55865364	1	196,932,199	G	A	0.0318	0.2313	0.0502	4.2209E-06	6,572	21.196	TRUE
ebi-PL179-GCST90277263	Diacylglycerol (18:1_18:3) levels	rs13237829	7	74,460,551	A	G	0.5028	-0.0808	0.0173	3.0328E-06	6,572	21.834	TRUE
ebi-PL179-GCST90277263	Diacylglycerol (18:1_18:3) levels	rs7599400	2	36,315,434	A	C	0.0080	0.4897	0.0960	3.4563E-07	6,572	26.024	TRUE
ebi-PL179-GCST90277263	Diacylglycerol (18:1_18:3) levels	rs180761622	8	32,454,379	T	C	0.0331	0.2351	0.0491	1.7349E-06	6,572	22.908	TRUE
ebi-PL179-GCST90277263	Diacylglycerol (18:1_18:3) levels	rs114441702	4	141,972,167	G	A	0.0265	-0.2574	0.0561	4.5235E-06	6,572	21.062	TRUE
ebi-PL179-GCST90277263	Diacylglycerol (18:1_18:3) levels	rs112075774	16	23,263,670	A	G	0.0770	-0.1648	0.0339	1.2067E-06	6,572	23.609	TRUE
ebi-PL179-GCST90277263	Diacylglycerol (18:1_18:3) levels	rs117518401	3	133,895,761	T	C	0.1732	0.1120	0.0232	1.4773E-06	6,572	23.219	TRUE
ebi-PL179-GCST90277263	Diacylglycerol (18:1_18:3) levels	rs77442856	5	153,405,520	A	G	0.1223	-0.1233	0.0268	4.2136E-06	6,572	21.199	TRUE
ebi-PL179-GCST90277272	Phosphatidylcholine (14:0_16:0) levels	rs77191266	6	86,624,181	T	C	0.0367	0.2418	0.0526	4.4274E-06	5,497	21.111	TRUE
ebi-PL179-GCST90277272	Phosphatidylcholine (14:0_16:0) levels	rs9507859	13	27,228,661	T	C	0.7646	0.1106	0.0223	7.1340E-07	5,497	24.632	TRUE
ebi-PL179-GCST90277272	Phosphatidylcholine (14:0_16:0) levels	rs74939791	8	82,931,796	G	T	0.0298	0.2697	0.0567	2.0271E-06	5,497	22.616	TRUE
ebi-PL179-GCST90277272	Phosphatidylcholine (14:0_16:0) levels	rs7239464	18	33,463,076	T	C	0.2894	0.0990	0.0210	2.4286E-06	5,497	22.268	TRUE
ebi-PL179-GCST90277272	Phosphatidylcholine (14:0_16:0) levels	rs2014300	21	34,985,564	G	A	0.8877	0.1410	0.0303	3.4160E-06	5,497	21.612	TRUE
ebi-PL179-GCST90277272	Phosphatidylcholine (14:0_16:0) levels	rs6050	4	154,586,438	C	T	0.3232	0.0948	0.0203	3.2427E-06	5,497	21.709	TRUE
ebi-PL179-GCST90277272	Phosphatidylcholine (14:0_16:0) levels	rs28416404	15	57,731,057	C	T	0.0793	-0.1659	0.0354	2.8395E-06	5,497	21.967	TRUE
ebi-PL179-GCST90277272	Phosphatidylcholine (14:0_16:0) levels	rs1230353	19	20,083,148	T	C	0.1846	-0.1144	0.0250	4.9181E-06	5,497	20.909	TRUE
ebi-PL179-GCST90277273	Phosphatidylcholine (14:0_18:1) levels	rs571968630	18	20,932,590	C	G	0.0114	0.5470	0.0796	6.7383E-12	7,064	47.258	TRUE
ebi-PL179-GCST90277273	Phosphatidylcholine (14:0_18:1) levels	rs147585949	16	20,715,373	A	G	0.0054	-0.5821	0.1233	2.3725E-06	7,064	22.303	TRUE
ebi-PL179-GCST90277273	Phosphatidylcholine (14:0_18:1) levels	rs6531968	4	87,278,332	G	T	0.5445	-0.0786	0.0170	3.7860E-06	7,064	21.404	TRUE
ebi-PL179-GCST90277273	Phosphatidylcholine (14:0_18:1) levels	rs2946493	8	12,966,162	C	T	0.2061	0.0987	0.0211	2.9080E-06	7,064	21.909	TRUE
ebi-PL179-GCST90277273	Phosphatidylcholine (14:0_18:1) levels	rs113668788	9	137,711,224	A	G	0.1079	0.1347	0.0282	1.8143E-06	7,064	22.817	TRUE
ebi-PL179-GCST90277273	Phosphatidylcholine (14:0_18:1) levels	rs2379129	20	62,310,086	A	C	0.3995	-0.0905	0.0173	1.6524E-07	7,064	27.458	TRUE
ebi-PL179-GCST90277273	Phosphatidylcholine (14:0_18:1) levels	rs12446515	16	56,953,103	T	C	0.2786	0.0971	0.0187	2.2956E-07	7,064	26.819	TRUE

Lipid GWAS ID	Lipid species	SNP	Chromosome	Position	Effect allele	Other allele	Effect-allele frequency	Beta	SE	P value	Sample size	F statistic	F > 10
ebi-PL179-GCST90277273	Phosphatidylcholine (14:0_18:1) levels	rs62189362	2	227,401,238	C	T	0.1077	0.1243	0.0271	4.4485E-06	7,064	21.092	TRUE
ebi-PL179-GCST90277273	Phosphatidylcholine (14:0_18:1) levels	rs7484827	12	47,729,780	G	A	0.2411	0.0903	0.0196	4.2106E-06	7,064	21.199	TRUE
ebi-PL179-GCST90277273	Phosphatidylcholine (14:0_18:1) levels	rs78414457	13	37,381,120	T	G	0.0744	0.1548	0.0328	2.4322E-06	7,064	22.254	TRUE
ebi-PL179-GCST90277273	Phosphatidylcholine (14:0_18:1) levels	rs2686446	3	195,507,255	C	G	0.7113	0.0899	0.0186	1.4095E-06	7,064	23.305	TRUE
ebi-PL179-GCST90277273	Phosphatidylcholine (14:0_18:1) levels	rs75785594	18	21,353,224	G	A	0.0100	0.4190	0.0856	1.0123E-06	7,064	23.944	TRUE
ebi-PL179-GCST90277273	Phosphatidylcholine (14:0_18:1) levels	rs2070474	22	24,495,324	G	C	0.5485	0.0854	0.0170	5.0966E-07	7,064	25.272	TRUE
ebi-PL179-GCST90277274	Phosphatidylcholine (14:0_18:2) levels	rs950668	15	71,629,500	A	G	0.4829	-0.0812	0.0177	4.4970E-06	6,866	21.073	TRUE
ebi-PL179-GCST90277274	Phosphatidylcholine (14:0_18:2) levels	rs1262295	1	201,408,310	G	A	0.8643	0.1169	0.0253	3.8588E-06	6,866	21.366	TRUE
ebi-PL179-GCST90277274	Phosphatidylcholine (14:0_18:2) levels	rs12137781	1	79,358,390	A	G	0.0590	-0.1704	0.0368	3.8313E-06	6,866	21.381	TRUE
ebi-PL179-GCST90277274	Phosphatidylcholine (14:0_18:2) levels	rs4665972	2	27,375,230	C	T	0.6203	-0.1147	0.0175	6.2379E-11	6,866	42.878	TRUE
ebi-PL179-GCST90277274	Phosphatidylcholine (14:0_18:2) levels	rs12113381	7	1,103,491	T	C	0.1203	0.1229	0.0264	3.2442E-06	6,866	21.701	TRUE
ebi-PL179-GCST90277274	Phosphatidylcholine (14:0_18:2) levels	rs174548	11	61,803,876	G	C	0.3836	0.1841	0.0175	1.1453E-25	6,866	110.578	TRUE
ebi-PL179-GCST90277274	Phosphatidylcholine (14:0_18:2) levels	rs150543432	18	21,887,951	T	A	0.0135	0.3538	0.0757	3.0480E-06	6,866	21.821	TRUE
ebi-PL179-GCST90277274	Phosphatidylcholine (14:0_18:2) levels	rs500246	8	37,124,499	G	A	0.8953	-0.1306	0.0279	2.9782E-06	6,866	21.866	TRUE
ebi-PL179-GCST90277274	Phosphatidylcholine (14:0_18:2) levels	rs6089695	20	62,232,970	T	C	0.4193	-0.0827	0.0173	1.7938E-06	6,866	22.842	TRUE
ebi-PL179-GCST90277274	Phosphatidylcholine (14:0_18:2) levels	rs7281206	21	37,359,926	C	T	0.5062	0.0881	0.0169	1.9297E-07	6,866	27.156	TRUE
ebi-PL179-GCST90277274	Phosphatidylcholine (14:0_18:2) levels	rs6045639	20	1,953,902	G	A	0.3237	0.0917	0.0183	5.2044E-07	6,866	25.233	TRUE
ebi-PL179-GCST90277275	Phosphatidylcholine (15:0_18:1) levels	rs11075022	16	11,827,406	C	T	0.5700	0.0857	0.0178	1.5971E-06	6,468	23.070	TRUE
ebi-PL179-GCST90277275	Phosphatidylcholine (15:0_18:1) levels	rs10917404	1	23,639,339	T	C	0.2266	-0.0980	0.0211	3.3197E-06	6,468	21.662	TRUE
ebi-PL179-GCST90277275	Phosphatidylcholine (15:0_18:1) levels	rs6866162	5	174,328,650	C	T	0.1265	0.1341	0.0277	1.3377E-06	6,468	23.410	TRUE
ebi-PL179-GCST90277275	Phosphatidylcholine (15:0_18:1) levels	rs581080	9	15,305,380	C	G	0.8562	0.1169	0.0249	2.7522E-06	6,468	22.021	TRUE
ebi-PL179-GCST90277275	Phosphatidylcholine (15:0_18:1) levels	rs72815248	16	54,459,960	T	C	0.1221	-0.1299	0.0271	1.6577E-06	6,468	22.996	TRUE
ebi-PL179-GCST90277275	Phosphatidylcholine (15:0_18:1) levels	rs12916925	15	38,647,674	A	T	0.4607	0.0810	0.0176	4.4136E-06	6,468	21.110	TRUE
ebi-PL179-GCST90277275	Phosphatidylcholine (15:0_18:1) levels	rs12926159	16	88,735,092	A	G	0.0128	-0.4314	0.0796	6.1035E-08	6,468	29.402	TRUE
ebi-PL179-GCST90277275	Phosphatidylcholine (15:0_18:1) levels	rs142477396	6	21,056,212	A	G	0.1745	-0.1069	0.0231	3.7698E-06	6,468	21.411	TRUE
ebi-PL179-GCST90277275	Phosphatidylcholine (15:0_18:1) levels	rs1532085	15	58,391,167	G	A	0.5734	-0.0894	0.0177	4.6714E-07	6,468	25.441	TRUE
ebi-PL179-GCST90277276	Phosphatidylcholine (15:0_18:2) levels	rs10468017	15	58,386,313	T	C	0.3375	0.1119	0.0181	7.4147E-10	6,639	38.020	TRUE
ebi-PL179-GCST90277276	Phosphatidylcholine (15:0_18:2) levels	rs17231506	16	56,960,616	T	C	0.2783	0.1428	0.0192	1.2029E-13	6,639	55.230	TRUE
ebi-PL179-GCST90277276	Phosphatidylcholine (15:0_18:2) levels	rs1911704	2	41,471,833	C	G	0.3491	-0.0835	0.0182	4.6489E-06	6,639	21.010	TRUE
ebi-PL179-GCST90277276	Phosphatidylcholine (15:0_18:2) levels	rs16968712	13	106,475,436	C	T	0.1192	-0.1319	0.0281	2.6305E-06	6,639	22.106	TRUE
ebi-PL179-GCST90277276	Phosphatidylcholine (15:0_18:2) levels	rs77404354	8	64,641,039	T	C	0.0085	-0.4784	0.1002	1.8417E-06	6,639	22.792	TRUE
ebi-PL179-GCST90277276	Phosphatidylcholine (15:0_18:2) levels	rs6769712	3	24,997,572	A	T	0.4789	-0.0843	0.0173	1.0713E-06	6,639	23.839	TRUE
ebi-PL179-GCST90277276	Phosphatidylcholine (15:0_18:2) levels	rs56144603	6	73,220,880	A	G	0.0315	0.2474	0.0513	1.4197E-06	6,639	23.294	TRUE
ebi-PL179-GCST90277276	Phosphatidylcholine (15:0_18:2) levels	rs55727637	16	15,051,395	T	C	0.2990	0.1110	0.0190	5.2160E-09	6,639	34.194	TRUE
ebi-PL179-GCST90277276	Phosphatidylcholine (15:0_18:2) levels	rs13101957	4	169,278,135	A	C	0.1301	-0.1217	0.0260	2.8913E-06	6,639	21.924	TRUE
ebi-PL179-GCST90277276	Phosphatidylcholine (15:0_18:2) levels	rs13281002	8	65,661,212	A	G	0.0268	-0.2616	0.0533	9.5871E-07	6,639	24.053	TRUE
ebi-PL179-GCST90277276	Phosphatidylcholine (15:0_18:2) levels	rs75097472	12	130,443,132	A	G	0.0115	-0.3829	0.0835	4.6503E-06	6,639	21.009	TRUE
ebi-PL179-GCST90277276	Phosphatidylcholine (15:0_18:2) levels	rs114498622	2	213,698,079	C	A	0.0156	0.3472	0.0755	4.3181E-06	6,639	21.154	TRUE
ebi-PL179-GCST90277276	Phosphatidylcholine (15:0_18:2) levels	rs174566	11	61,824,890	G	A	0.4084	0.3170	0.0173	1.4565E-73	6,639	337.472	TRUE
ebi-PL179-GCST90277276	Phosphatidylcholine (15:0_18:2) levels	rs139075653	6	114,209,369	C	T	0.0044	0.6456	0.1382	3.0402E-06	6,639	21.826	TRUE
ebi-PL179-GCST90277276	Phosphatidylcholine (15:0_18:2) levels	rs184289628	6	139,585,552	G	T	0.0182	0.2989	0.0645	3.6748E-06	6,639	21.463	TRUE
ebi-PL179-GCST90277276	Phosphatidylcholine (15:0_18:2) levels	rs12113214	7	6,530,143	A	T	0.3229	0.0931	0.0188	7.3366E-07	6,639	24.570	TRUE
ebi-PL179-GCST90277276	Phosphatidylcholine (15:0_18:2) levels	rs1168036	1	62,497,063	G	A	0.7379	0.1069	0.0196	4.7809E-08	6,639	29.871	TRUE
ebi-PL179-GCST90277276	Phosphatidylcholine (15:0_18:2) levels	rs10961143	9	13,564,220	G	A	0.0483	0.2035	0.0401	3.8608E-07	6,639	25.813	TRUE
ebi-PL179-GCST90277276	Phosphatidylcholine (15:0_18:2) levels	rs35960238	10	26,331,627	G	A	0.1266	-0.1310	0.0261	5.3665E-07	6,639	25.174	TRUE
ebi-PL179-GCST90277276	Phosphatidylcholine (15:0_18:2) levels	rs4382917	11	62,454,004	A	G	0.2558	-0.1008	0.0206	9.8434E-07	6,639	24.002	TRUE
ebi-PL179-GCST90277276	Phosphatidylcholine (15:0_18:2) levels	rs4335452	10	5,872,836	A	G	0.8001	-0.1035	0.0224	3.8767E-06	6,639	21.360	TRUE
ebi-PL179-GCST90277276	Phosphatidylcholine (15:0_18:2) levels	rs1488201	3	26,848,389	G	T	0.3497	0.0847	0.0183	3.6009E-06	6,639	21.501	TRUE
ebi-PL179-GCST90277276	Phosphatidylcholine (15:0_18:2) levels	rs633695	15	58,433,640	G	A	0.3139	0.0930	0.0189	9.2779E-07	6,639	24.116	TRUE
ebi-PL179-GCST90277276	Phosphatidylcholine (15:0_18:2) levels	rs551412	9	132,450,788	T	A	0.2860	0.0884	0.0193	4.6380E-06	6,639	21.013	TRUE
ebi-PL179-GCST90277276	Phosphatidylcholine (15:0_18:2) levels	rs174527	11	61,770,929	C	G	0.4443	0.1022	0.0178	9.3281E-09	6,639	33.057	TRUE
ebi-PL179-GCST90277276	Phosphatidylcholine (15:0_18:2) levels	rs17614174	5	16,862,310	C	T	0.0524	-0.1874	0.0405	3.7488E-06	6,639	21.424	TRUE
ebi-PL179-GCST90277264	Phosphatidylcholine (16:0_0:0) levels	rs12116819	1	163,474,114	A	G	0.0296	-0.2464	0.0508	1.2394E-06	7,174	23.554	TRUE
ebi-PL179-GCST90277264	Phosphatidylcholine (16:0_0:0) levels	rs62231613	3	4,762,993	A	G	0.0087	-0.4420	0.0931	2.1050E-06	7,174	22.532	TRUE
ebi-PL179-GCST90277264	Phosphatidylcholine (16:0_0:0) levels	rs75668753	5	117,857,598	G	A	0.0211	-0.2966	0.0592	5.4866E-07	7,174	25.129	TRUE
ebi-PL179-GCST90277264	Phosphatidylcholine (16:0_0:0) levels	rs73200277	8	18,126,210	T	C	0.0217	0.2842	0.0590	1.4542E-06	7,174	23.248	TRUE
ebi-PL179-GCST90277264	Phosphatidylcholine (16:0_0:0) levels	rs248443	5	151,194,171	T	C	0.6979	-0.0938	0.0183	3.1053E-07	7,174	26.231	TRUE
ebi-PL179-GCST90277264	Phosphatidylcholine (16:0_0:0) levels	rs76530357	3	77,008,436	T	G	0.0245	0.2560	0.0545	2.7024E-06	7,174	22.051	TRUE

Lipid GWAS ID	Lipid species	SNP	Chromosome	Position	Effect allele	Other allele	Effect-allele frequency	Beta	SE	P value	Sample size	F statistic	F > 10
ebi-PL179-GCST90277264	Phosphatidylcholine (16:0_0:0) levels	rs571344	1	98,498,507	C	T	0.6300	0.0818	0.0178	4.2284E-06	7,174	21.190	TRUE
ebi-PL179-GCST90277264	Phosphatidylcholine (16:0_0:0) levels	rs148313235	19	54,065,411	G	T	0.0168	-0.3446	0.0673	3.1552E-07	7,174	26.201	TRUE
ebi-PL179-GCST90277264	Phosphatidylcholine (16:0_0:0) levels	rs7168957	15	61,598,332	T	C	0.9367	-0.1685	0.0343	9.0198E-07	7,174	24.168	TRUE
ebi-PL179-GCST90277264	Phosphatidylcholine (16:0_0:0) levels	rs62067063	17	9,294,060	C	T	0.5803	0.0858	0.0178	1.5013E-06	7,174	23.184	TRUE
ebi-PL179-GCST90277264	Phosphatidylcholine (16:0_0:0) levels	rs2714309	6	7,298,054	A	G	0.3913	0.0797	0.0172	3.6883E-06	7,174	21.452	TRUE
ebi-PL179-GCST90277264	Phosphatidylcholine (16:0_0:0) levels	rs75563920	4	80,232,181	C	T	0.1174	-0.1257	0.0259	1.2889E-06	7,174	23.479	TRUE
ebi-PL179-GCST90277264	Phosphatidylcholine (16:0_0:0) levels	rs191695863	11	40,267,436	A	C	0.0057	-0.5456	0.1187	4.4053E-06	7,174	21.111	TRUE
ebi-PL179-GCST90277264	Phosphatidylcholine (16:0_0:0) levels	rs17477122	12	26,619,127	G	A	0.1420	-0.1103	0.0241	4.7707E-06	7,174	20.960	TRUE
ebi-PL179-GCST90277264	Phosphatidylcholine (16:0_0:0) levels	rs7529794	1	39,937,698	T	G	0.2498	0.1342	0.0193	4.3161E-12	7,174	48.137	TRUE
ebi-PL179-GCST90277277	Phosphatidylcholine (16:0_16:0) levels	rs148444916	1	92,970,296	C	T	0.0208	0.2886	0.0576	5.6697E-07	7,174	25.065	TRUE
ebi-PL179-GCST90277277	Phosphatidylcholine (16:0_16:0) levels	rs174546	11	61,802,358	T	C	0.4074	-0.1489	0.0169	1.6921E-18	7,174	77.435	TRUE
ebi-PL179-GCST90277277	Phosphatidylcholine (16:0_16:0) levels	rs73015021	19	11,082,239	G	A	0.0921	-0.1582	0.0287	3.4887E-08	7,174	30.477	TRUE
ebi-PL179-GCST90277277	Phosphatidylcholine (16:0_16:0) levels	rs3936021	5	85,099,087	G	A	0.1581	-0.1068	0.0234	4.9584E-06	7,174	20.883	TRUE
ebi-PL179-GCST90277277	Phosphatidylcholine (16:0_16:0) levels	rs5022521	4	110,207,431	T	C	0.7973	-0.0974	0.0212	4.2613E-06	7,174	21.175	TRUE
ebi-PL179-GCST90277277	Phosphatidylcholine (16:0_16:0) levels	rs12522374	5	159,237,696	A	G	0.3474	0.0812	0.0174	3.1139E-06	7,174	21.778	TRUE
ebi-PL179-GCST90277277	Phosphatidylcholine (16:0_16:0) levels	rs72974647	6	123,327,335	G	A	0.0822	0.1440	0.0300	1.6120E-06	7,174	23.047	TRUE
ebi-PL179-GCST90277277	Phosphatidylcholine (16:0_16:0) levels	rs3134950	6	32,159,700	A	C	0.5835	0.0967	0.0168	9.9571E-09	7,174	32.925	TRUE
ebi-PL179-GCST90277277	Phosphatidylcholine (16:0_16:0) levels	rs72733996	9	80,936,784	G	A	0.1978	0.1012	0.0208	1.2301E-06	7,174	23.571	TRUE
ebi-PL179-GCST90277277	Phosphatidylcholine (16:0_16:0) levels	rs56270325	1	5,899,241	T	A	0.2592	0.0906	0.0188	1.4909E-06	7,174	23.196	TRUE
ebi-PL179-GCST90277277	Phosphatidylcholine (16:0_16:0) levels	rs261290	15	58,386,521	C	T	0.6122	-0.1390	0.0169	2.3743E-16	7,174	67.594	TRUE
ebi-PL179-GCST90277277	Phosphatidylcholine (16:0_16:0) levels	rs13060992	3	186,944,804	G	T	0.9475	0.1859	0.0376	7.5652E-07	7,174	24.508	TRUE
ebi-PL179-GCST90277277	Phosphatidylcholine (16:0_16:0) levels	rs11591147	1	55,039,974	T	G	0.0332	-0.2345	0.0466	4.9387E-07	7,174	25.332	TRUE
ebi-PL179-GCST90277277	Phosphatidylcholine (16:0_16:0) levels	rs4744927	9	69,669,802	C	A	0.9979	0.9074	0.1920	2.3501E-06	7,174	22.326	TRUE
ebi-PL179-GCST90277277	Phosphatidylcholine (16:0_16:0) levels	rs2236514	9	136,677,616	G	C	0.6471	-0.0839	0.0177	2.3195E-06	7,174	22.345	TRUE
ebi-PL179-GCST90277277	Phosphatidylcholine (16:0_16:0) levels	rs643531	9	15,296,036	A	C	0.8896	0.1246	0.0261	1.9067E-06	7,174	22.723	TRUE
ebi-PL179-GCST90277277	Phosphatidylcholine (16:0_16:0) levels	rs1187583	14	54,570,145	A	G	0.9163	-0.1417	0.0300	2.4053E-06	7,174	22.275	TRUE
ebi-PL179-GCST90277277	Phosphatidylcholine (16:0_16:0) levels	rs78909142	22	40,993,330	A	G	0.0086	-0.4333	0.0945	4.5590E-06	7,174	21.044	TRUE
ebi-PL179-GCST90277278	Phosphatidylcholine (16:0_16:1) levels	rs113366516	6	138,148,100	T	G	0.0170	0.3082	0.0661	3.1398E-06	7,172	21.762	TRUE
ebi-PL179-GCST90277278	Phosphatidylcholine (16:0_16:1) levels	rs11647818	16	11,833,509	T	C	0.5746	0.0787	0.0170	3.6751E-06	7,172	21.459	TRUE
ebi-PL179-GCST90277278	Phosphatidylcholine (16:0_16:1) levels	rs80227620	2	167,832,629	T	C	0.1440	0.1186	0.0244	1.1942E-06	7,172	23.625	TRUE
ebi-PL179-GCST90277278	Phosphatidylcholine (16:0_16:1) levels	rs1854434	9	32,796,762	G	A	0.1540	-0.1099	0.0232	2.1453E-06	7,172	22.496	TRUE
ebi-PL179-GCST90277278	Phosphatidylcholine (16:0_16:1) levels	rs117571757	17	16,458,446	A	G	0.0135	-0.3342	0.0728	4.4382E-06	7,172	21.098	TRUE
ebi-PL179-GCST90277278	Phosphatidylcholine (16:0_16:1) levels	rs28524821	4	17,068,686	C	A	0.1455	0.1086	0.0235	3.9762E-06	7,172	21.308	TRUE
ebi-PL179-GCST90277278	Phosphatidylcholine (16:0_16:1) levels	rs10760286	9	123,419,309	C	T	0.5100	0.0789	0.0170	3.3164E-06	7,172	21.657	TRUE
ebi-PL179-GCST90277278	Phosphatidylcholine (16:0_16:1) levels	rs35235479	10	92,767,403	G	A	0.2141	-0.0962	0.0203	2.2375E-06	7,172	22.416	TRUE
ebi-PL179-GCST90277278	Phosphatidylcholine (16:0_16:1) levels	rs77007318	16	59,856,268	C	G	0.0289	-0.2411	0.0522	3.8750E-06	7,172	21.357	TRUE
ebi-PL179-GCST90277278	Phosphatidylcholine (16:0_16:1) levels	rs62098276	18	63,512,537	C	T	0.0797	-0.1421	0.0307	3.8030E-06	7,172	21.393	TRUE
ebi-PL179-GCST90277279	Phosphatidylcholine (16:0_17:1) levels	rs116911263	10	95,941,250	T	C	0.0136	-0.3515	0.0729	1.4668E-06	7,076	23.230	TRUE
ebi-PL179-GCST90277279	Phosphatidylcholine (16:0_17:1) levels	rs41488845	17	38,380,397	A	G	0.0072	-0.4958	0.1043	2.0430E-06	7,076	22.590	TRUE
ebi-PL179-GCST90277279	Phosphatidylcholine (16:0_17:1) levels	rs12475699	2	30,022,181	C	T	0.6634	0.0811	0.0177	4.8591E-06	7,076	20.927	TRUE
ebi-PL179-GCST90277279	Phosphatidylcholine (16:0_17:1) levels	rs2122922	8	101,050,430	T	C	0.2015	-0.0977	0.0213	4.6059E-06	7,076	21.026	TRUE
ebi-PL179-GCST90277279	Phosphatidylcholine (16:0_17:1) levels	rs16986523	20	24,390,991	A	G	0.0216	0.2684	0.0580	3.7758E-06	7,076	21.409	TRUE
ebi-PL179-GCST90277279	Phosphatidylcholine (16:0_17:1) levels	rs12749808	1	158,095,972	T	C	0.3558	-0.0836	0.0178	2.7546E-06	7,076	22.015	TRUE
ebi-PL179-GCST90277279	Phosphatidylcholine (16:0_17:1) levels	rs78395462	4	122,641,012	T	G	0.0238	0.2575	0.0552	3.1712E-06	7,076	21.745	TRUE
ebi-PL179-GCST90277279	Phosphatidylcholine (16:0_17:1) levels	rs6496787	15	91,344,956	G	T	0.6084	0.0929	0.0173	8.2689E-08	7,076	28.803	TRUE
ebi-PL179-GCST90277279	Phosphatidylcholine (16:0_17:1) levels	rs7177289	15	58,387,985	T	C	0.3935	0.0822	0.0171	1.5274E-06	7,076	23.146	TRUE
ebi-PL179-GCST90277280	Phosphatidylcholine (16:0_18:0) levels	rs117495065	6	147,174,911	G	C	0.0175	0.3017	0.0654	4.0166E-06	7,154	21.288	TRUE
ebi-PL179-GCST90277280	Phosphatidylcholine (16:0_18:0) levels	rs247617	16	56,956,804	A	C	0.2782	0.0919	0.0186	7.7657E-07	7,154	24.457	TRUE
ebi-PL179-GCST90277280	Phosphatidylcholine (16:0_18:0) levels	rs72723967	4	137,812,361	A	C	0.1631	0.1106	0.0227	1.1781E-06	7,154	23.652	TRUE
ebi-PL179-GCST90277280	Phosphatidylcholine (16:0_18:0) levels	rs662846	20	819,559	A	C	0.0233	-0.2717	0.0567	1.6559E-06	7,154	22.994	TRUE
ebi-PL179-GCST90277280	Phosphatidylcholine (16:0_18:0) levels	rs12052409	2	68,074,558	C	A	0.0516	-0.1932	0.0386	5.5670E-07	7,154	25.100	TRUE
ebi-PL179-GCST90277280	Phosphatidylcholine (16:0_18:0) levels	rs2214660	7	122,869,647	A	G	0.1850	-0.1019	0.0217	2.5591E-06	7,154	22.156	TRUE
ebi-PL179-GCST90277280	Phosphatidylcholine (16:0_18:0) levels	rs62330208	5	983,129	C	G	0.2398	-0.1028	0.0204	4.6942E-07	7,154	25.431	TRUE
ebi-PL179-GCST90277280	Phosphatidylcholine (16:0_18:0) levels	rs10853781	19	47,242,784	G	A	0.4956	0.0793	0.0170	3.3218E-06	7,154	21.653	TRUE
ebi-PL179-GCST90277280	Phosphatidylcholine (16:0_18:0) levels	rs4659619	1	236,035,687	C	T	0.3382	0.0849	0.0175	1.2057E-06	7,154	23.607	TRUE
ebi-PL179-GCST90277280	Phosphatidylcholine (16:0_18:0) levels	rs10890773	11	107,889,276	A	G	0.8603	-0.1170	0.0239	1.0229E-06	7,154	23.925	TRUE
ebi-PL179-GCST90277280	Phosphatidylcholine (16:0_18:0) levels	rs76413658	16	6,589,736	C	G	0.1331	-0.1116	0.0244	4.7251E-06	7,154	20.975	TRUE
ebi-PL179-GCST90277280	Phosphatidylcholine (16:0_18:0) levels	rs2126263	8	9,324,101	A	G	0.8498	0.1109	0.0234	2.2969E-06	7,154	22.363	TRUE

Lipid GWAS ID	Lipid species	SNP	Chromosome	Position	Effect allele	Other allele	Effect-allele frequency	Beta	SE	P value	Sample size	F statistic	F > 10
ebi-PL179-GCST90277280	Phosphatidylcholine (16:0_18:0) levels	rs11723712	4	169,304,745	A	G	0.8064	0.1032	0.0212	1.1095E-06	7,154	23.768	TRUE
ebi-PL179-GCST90277280	Phosphatidylcholine (16:0_18:0) levels	rs113076792	7	153,170,994	A	G	0.0136	-0.3376	0.0738	4.8386E-06	7,154	20.932	TRUE
ebi-PL179-GCST90277280	Phosphatidylcholine (16:0_18:0) levels	rs117528775	18	49,649,558	C	T	0.0049	0.5936	0.1254	2.2494E-06	7,154	22.404	TRUE
ebi-PL179-GCST90277280	Phosphatidylcholine (16:0_18:0) levels	rs174601	11	61,855,668	T	C	0.4190	0.1464	0.0169	6.0020E-18	7,154	74.911	TRUE
ebi-PL179-GCST90277281	Phosphatidylcholine (16:0_18:1) levels	rs28807772	5	165,822,116	G	A	0.2018	0.0952	0.0208	4.7244E-06	7,174	20.977	TRUE
ebi-PL179-GCST90277281	Phosphatidylcholine (16:0_18:1) levels	rs1109771	6	32,219,828	G	A	0.4968	-0.0827	0.0167	7.3689E-07	7,174	24.560	TRUE
ebi-PL179-GCST90277281	Phosphatidylcholine (16:0_18:1) levels	rs4538911	8	6,228,839	C	G	0.7034	-0.0905	0.0184	8.5435E-07	7,174	24.271	TRUE
ebi-PL179-GCST90277281	Phosphatidylcholine (16:0_18:1) levels	rs77027238	17	43,849,174	A	G	0.0496	0.1851	0.0385	1.5399E-06	7,174	23.135	TRUE
ebi-PL179-GCST90277281	Phosphatidylcholine (16:0_18:1) levels	rs116997061	12	19,869,115	T	C	0.0619	-0.1699	0.0360	2.3499E-06	7,174	22.320	TRUE
ebi-PL179-GCST90277282	Phosphatidylcholine (16:0_18:2) levels	rs1833172	16	6,626,446	T	C	0.0715	0.1573	0.0327	1.6002E-06	7,174	23.060	TRUE
ebi-PL179-GCST90277282	Phosphatidylcholine (16:0_18:2) levels	rs62039480	16	15,043,593	A	G	0.3320	0.1160	0.0177	6.5159E-11	7,174	42.786	TRUE
ebi-PL179-GCST90277282	Phosphatidylcholine (16:0_18:2) levels	rs10787711	10	116,647,273	A	G	0.6485	-0.0900	0.0175	2.7391E-07	7,174	26.474	TRUE
ebi-PL179-GCST90277282	Phosphatidylcholine (16:0_18:2) levels	rs17126640	12	52,311,207	A	G	0.2035	-0.0994	0.0208	1.8890E-06	7,174	22.741	TRUE
ebi-PL179-GCST90277282	Phosphatidylcholine (16:0_18:2) levels	rs1800588	15	58,431,476	T	C	0.2580	0.1377	0.0193	1.0910E-12	7,174	50.854	TRUE
ebi-PL179-GCST90277282	Phosphatidylcholine (16:0_18:2) levels	rs143066473	12	99,432,175	A	G	0.0249	0.2542	0.0552	4.2878E-06	7,174	21.163	TRUE
ebi-PL179-GCST90277282	Phosphatidylcholine (16:0_18:2) levels	rs13150924	4	169,277,659	A	G	0.1302	-0.1346	0.0250	7.1965E-08	7,174	29.071	TRUE
ebi-PL179-GCST90277282	Phosphatidylcholine (16:0_18:2) levels	rs2629180	3	3,947,204	A	G	0.0039	-0.6945	0.1476	2.5608E-06	7,174	22.154	TRUE
ebi-PL179-GCST90277282	Phosphatidylcholine (16:0_18:2) levels	rs56075928	11	63,135,902	A	G	0.0257	0.2447	0.0529	3.8496E-06	7,174	21.369	TRUE
ebi-PL179-GCST90277282	Phosphatidylcholine (16:0_18:2) levels	rs174574	11	61,832,870	C	A	0.5909	-0.3307	0.0166	5.6577E-86	7,174	396.747	TRUE
ebi-PL179-GCST90277282	Phosphatidylcholine (16:0_18:2) levels	rs13402814	2	137,186,441	T	A	0.0597	0.1643	0.0351	2.8865E-06	7,174	21.923	TRUE
ebi-PL179-GCST90277282	Phosphatidylcholine (16:0_18:2) levels	rs112569946	6	124,913,409	A	G	0.0032	0.7161	0.1557	4.3368E-06	7,174	21.143	TRUE
ebi-PL179-GCST90277282	Phosphatidylcholine (16:0_18:2) levels	rs11722429	4	136,284,098	A	G	0.0989	0.1333	0.0281	2.1771E-06	7,174	22.468	TRUE
ebi-PL179-GCST90277282	Phosphatidylcholine (16:0_18:2) levels	rs7936002	11	62,451,557	G	A	0.2572	-0.0993	0.0196	4.2743E-07	7,174	25.612	TRUE
ebi-PL179-GCST90277282	Phosphatidylcholine (16:0_18:2) levels	rs9436221	1	62,437,688	C	T	0.2614	-0.1267	0.0188	1.8228E-11	7,174	45.299	TRUE
ebi-PL179-GCST90277283	Phosphatidylcholine (16:0_18:3) levels	rs115079643	5	176,924,035	C	T	0.0236	-0.2824	0.0560	4.5980E-07	7,174	25.471	TRUE
ebi-PL179-GCST90277283	Phosphatidylcholine (16:0_18:3) levels	rs174598	11	61,853,722	A	G	0.3456	0.1117	0.0176	2.2419E-10	7,174	40.358	TRUE
ebi-PL179-GCST90277283	Phosphatidylcholine (16:0_18:3) levels	rs79189815	5	62,191,215	G	A	0.0091	0.4311	0.0910	2.1893E-06	7,174	22.456	TRUE
ebi-PL179-GCST90277283	Phosphatidylcholine (16:0_18:3) levels	rs1474236	3	117,112,654	C	T	0.8177	-0.1124	0.0228	8.2119E-07	7,174	24.349	TRUE
ebi-PL179-GCST90277283	Phosphatidylcholine (16:0_18:3) levels	rs192646452	17	27,754,096	T	A	0.0234	-0.2548	0.0555	4.5226E-06	7,174	21.061	TRUE
ebi-PL179-GCST90277283	Phosphatidylcholine (16:0_18:3) levels	rs138477772	18	62,042,253	A	G	0.0056	-0.5431	0.1166	3.2678E-06	7,174	21.685	TRUE
ebi-PL179-GCST90277283	Phosphatidylcholine (16:0_18:3) levels	rs147507697	3	76,641,189	G	A	0.0361	0.2093	0.0451	3.4639E-06	7,174	21.573	TRUE
ebi-PL179-GCST90277283	Phosphatidylcholine (16:0_18:3) levels	rs561601059	19	24,363,828	C	T	0.0390	-0.2089	0.0449	3.3841E-06	7,174	21.619	TRUE
ebi-PL179-GCST90277283	Phosphatidylcholine (16:0_18:3) levels	rs182611493	19	19,347,579	G	A	0.0538	-0.1887	0.0378	6.2822E-07	7,174	24.871	TRUE
ebi-PL179-GCST90277283	Phosphatidylcholine (16:0_18:3) levels	rs12255873	10	60,834,796	G	A	0.9008	-0.1394	0.0284	9.1089E-07	7,174	24.148	TRUE
ebi-PL179-GCST90277283	Phosphatidylcholine (16:0_18:3) levels	rs111506410	20	1,141,294	C	T	0.0277	0.2428	0.0516	2.6166E-06	7,174	22.112	TRUE
ebi-PL179-GCST90277284	Phosphatidylcholine (16:0_20:1) levels	rs1409892	6	51,400,810	A	G	0.4480	0.0789	0.0172	4.5353E-06	6,882	21.056	TRUE
ebi-PL179-GCST90277284	Phosphatidylcholine (16:0_20:1) levels	rs17715191	10	114,348,244	G	A	0.1829	0.1058	0.0231	4.7692E-06	6,882	20.957	TRUE
ebi-PL179-GCST90277284	Phosphatidylcholine (16:0_20:1) levels	rs174594	11	61,852,357	A	C	0.5815	-0.1146	0.0173	3.5787E-11	6,882	43.969	TRUE
ebi-PL179-GCST90277284	Phosphatidylcholine (16:0_20:1) levels	rs10995430	10	63,087,988	T	C	0.6505	-0.0843	0.0179	2.6056E-06	6,882	22.122	TRUE
ebi-PL179-GCST90277284	Phosphatidylcholine (16:0_20:1) levels	rs150608914	5	137,344,967	A	G	0.0497	0.1951	0.0395	8.2548E-07	6,882	24.340	TRUE
ebi-PL179-GCST90277284	Phosphatidylcholine (16:0_20:1) levels	rs2268493	3	8,759,154	C	T	0.3919	0.0845	0.0176	1.6001E-06	6,882	23.064	TRUE
ebi-PL179-GCST90277284	Phosphatidylcholine (16:0_20:1) levels	rs73728261	6	18,747,694	T	C	0.1287	0.1167	0.0250	3.1147E-06	6,882	21.779	TRUE
ebi-PL179-GCST90277284	Phosphatidylcholine (16:0_20:1) levels	rs12707188	7	134,906,346	T	C	0.4169	0.0922	0.0176	1.6582E-07	6,882	27.451	TRUE
ebi-PL179-GCST90277284	Phosphatidylcholine (16:0_20:1) levels	rs6007233	22	44,667,863	T	C	0.6785	0.0849	0.0184	4.0633E-06	6,882	21.269	TRUE
ebi-PL179-GCST90277285	Phosphatidylcholine (16:0_20:2) levels	rs2317091	11	63,193,288	A	T	0.0403	0.2182	0.0430	4.1058E-07	7,135	25.690	TRUE
ebi-PL179-GCST90277285	Phosphatidylcholine (16:0_20:2) levels	rs79718024	11	107,569,619	G	A	0.1546	-0.1125	0.0235	1.7556E-06	7,135	22.881	TRUE
ebi-PL179-GCST90277285	Phosphatidylcholine (16:0_20:2) levels	rs142392117	4	176,627,941	C	T	0.0216	-0.2767	0.0573	1.3956E-06	7,135	23.325	TRUE
ebi-PL179-GCST90277285	Phosphatidylcholine (16:0_20:2) levels	rs10160784	11	75,745,010	C	T	0.2324	-0.1096	0.0199	3.6055E-08	7,135	30.417	TRUE
ebi-PL179-GCST90277285	Phosphatidylcholine (16:0_20:2) levels	rs174592	11	61,851,136	G	A	0.4185	0.2617	0.0168	4.9569E-54	7,135	243.594	TRUE
ebi-PL179-GCST90277285	Phosphatidylcholine (16:0_20:2) levels	rs17268633	2	56,194,921	G	A	0.0203	-0.2853	0.0601	2.0682E-06	7,135	22.566	TRUE
ebi-PL179-GCST90277285	Phosphatidylcholine (16:0_20:2) levels	rs12700446	7	23,532,125	T	C	0.0161	0.3086	0.0674	4.7347E-06	7,135	20.972	TRUE
ebi-PL179-GCST90277285	Phosphatidylcholine (16:0_20:2) levels	rs34516410	6	99,968,458	T	G	0.1334	-0.1142	0.0248	4.1618E-06	7,135	21.221	TRUE
ebi-PL179-GCST90277285	Phosphatidylcholine (16:0_20:2) levels	rs1815763	11	67,075,266	A	G	0.0112	-0.3663	0.0797	4.3859E-06	7,135	21.119	TRUE
ebi-PL179-GCST90277285	Phosphatidylcholine (16:0_20:2) levels	rs72800299	10	65,395,218	A	T	0.2738	-0.0849	0.0185	4.6922E-06	7,135	20.990	TRUE
ebi-PL179-GCST90277285	Phosphatidylcholine (16:0_20:2) levels	rs78230994	4	26,558,575	A	G	0.0055	0.5946	0.1226	1.2476E-06	7,135	23.539	TRUE
ebi-PL179-GCST90277285	Phosphatidylcholine (16:0_20:2) levels	rs148069564	10	51,792,352	A	T	0.0030	-0.7772	0.1669	3.2790E-06	7,135	21.679	TRUE
ebi-PL179-GCST90277285	Phosphatidylcholine (16:0_20:2) levels	rs1168041	1	62,494,579	C	T	0.7333	0.0926	0.0189	9.2897E-07	7,135	24.111	TRUE
ebi-PL179-GCST90277285	Phosphatidylcholine (16:0_20:2) levels	rs113130033	14	89,719,965	G	A	0.0430	-0.1966	0.0411	1.7305E-06	7,135	22.910	TRUE

Lipid GWAS ID	Lipid species	SNP	Chromosome	Position	Effect allele	Other allele	Effect-allele frequency	Beta	SE	P value	Sample size	F statistic	F > 10
ebi-PL179-GCST90277285	Phosphatidylcholine (16:0_20:2) levels	rs76711488	18	8,971,803	A	G	0.0233	-0.2596	0.0560	3.6000E-06	7,135	21.499	TRUE
ebi-PL179-GCST90277285	Phosphatidylcholine (16:0_20:2) levels	rs150057262	19	19,210,016	G	C	0.0522	-0.2024	0.0387	1.7659E-07	7,135	27.326	TRUE
ebi-PL179-GCST90277285	Phosphatidylcholine (16:0_20:2) levels	rs113550786	16	13,890,509	G	A	0.0123	0.3931	0.0827	2.0601E-06	7,135	22.574	TRUE
ebi-PL179-GCST90277285	Phosphatidylcholine (16:0_20:2) levels	rs62081852	18	26,802,732	T	C	0.0177	0.3890	0.0677	9.3471E-09	7,135	33.049	TRUE
ebi-PL179-GCST90277286	Phosphatidylcholine (16:0_20:3) levels	rs968567	11	61,828,092	T	C	0.1130	0.2567	0.0264	3.0474E-22	7,174	94.689	TRUE
ebi-PL179-GCST90277286	Phosphatidylcholine (16:0_20:3) levels	rs6498540	16	15,036,737	G	A	0.3340	-0.1923	0.0176	1.9042E-27	7,174	118.788	TRUE
ebi-PL179-GCST90277286	Phosphatidylcholine (16:0_20:3) levels	rs138705401	12	43,610,337	T	C	0.0471	0.1827	0.0398	4.4753E-06	7,174	21.081	TRUE
ebi-PL179-GCST90277286	Phosphatidylcholine (16:0_20:3) levels	rs141883259	5	120,061,007	G	A	0.0102	0.3797	0.0830	4.8929E-06	7,174	20.909	TRUE
ebi-PL179-GCST90277286	Phosphatidylcholine (16:0_20:3) levels	rs7173306	15	92,337,647	C	T	0.6740	-0.0839	0.0181	3.7793E-06	7,174	21.406	TRUE
ebi-PL179-GCST90277286	Phosphatidylcholine (16:0_20:3) levels	rs10102672	8	39,183,266	G	C	0.2925	0.0865	0.0184	2.5716E-06	7,174	22.146	TRUE
ebi-PL179-GCST90277286	Phosphatidylcholine (16:0_20:3) levels	rs1410901	10	90,245,408	T	C	0.5049	0.0792	0.0170	3.2424E-06	7,174	21.700	TRUE
ebi-PL179-GCST90277287	Phosphatidylcholine (16:0_20:4) levels	rs79136768	11	61,594,967	G	A	0.0369	-0.2065	0.0448	4.0384E-06	7,174	21.277	TRUE
ebi-PL179-GCST90277287	Phosphatidylcholine (16:0_20:4) levels	rs1251003	8	8,854,096	A	T	0.0899	-0.1439	0.0296	1.2303E-06	7,174	23.568	TRUE
ebi-PL179-GCST90277287	Phosphatidylcholine (16:0_20:4) levels	rs4756323	11	36,469,520	T	C	0.4558	-0.0776	0.0166	3.2167E-06	7,174	21.716	TRUE
ebi-PL179-GCST90277287	Phosphatidylcholine (16:0_20:4) levels	rs1792909	11	62,170,073	A	G	0.3873	-0.0901	0.0171	1.4995E-07	7,174	27.633	TRUE
ebi-PL179-GCST90277287	Phosphatidylcholine (16:0_20:4) levels	rs141391095	17	10,763,022	G	A	0.0209	-0.2809	0.0597	2.5814E-06	7,174	22.138	TRUE
ebi-PL179-GCST90277287	Phosphatidylcholine (16:0_20:4) levels	rs147549994	16	1,939,264	T	G	0.0359	0.2463	0.0453	5.5129E-08	7,174	29.586	TRUE
ebi-PL179-GCST90277287	Phosphatidylcholine (16:0_20:4) levels	rs499974	11	75,743,976	A	C	0.2323	-0.1027	0.0198	2.2576E-07	7,174	26.853	TRUE
ebi-PL179-GCST90277287	Phosphatidylcholine (16:0_20:4) levels	rs72709529	14	101,397,558	A	C	0.0233	-0.2725	0.0559	1.1263E-06	7,174	23.740	TRUE
ebi-PL179-GCST90277287	Phosphatidylcholine (16:0_20:4) levels	rs3741252	11	61,744,026	T	C	0.1337	0.2104	0.0245	1.1695E-17	7,174	73.572	TRUE
ebi-PL179-GCST90277287	Phosphatidylcholine (16:0_20:4) levels	rs116643980	1	4,847,659	T	G	0.0556	0.1675	0.0366	4.8414E-06	7,174	20.930	TRUE
ebi-PL179-GCST90277287	Phosphatidylcholine (16:0_20:4) levels	rs3820438	1	201,955,621	C	G	0.2908	0.0891	0.0183	1.1453E-06	7,174	23.709	TRUE
ebi-PL179-GCST90277287	Phosphatidylcholine (16:0_20:4) levels	rs2943813	11	61,490,996	C	T	0.7830	-0.1390	0.0204	9.9642E-12	7,174	46.477	TRUE
ebi-PL179-GCST90277287	Phosphatidylcholine (16:0_20:4) levels	rs116438525	4	181,645,150	T	C	0.0071	-0.4702	0.1020	4.0531E-06	7,174	21.272	TRUE
ebi-PL179-GCST90277287	Phosphatidylcholine (16:0_20:4) levels	rs174528	11	61,776,027	C	T	0.4144	-0.5872	0.0155	1.0000E-200	7,174	1427.202	TRUE
ebi-PL179-GCST90277288	Phosphatidylcholine (16:0_20:5) levels	rs113917790	6	21,142,482	T	C	0.0132	0.3500	0.0743	2.5458E-06	7,173	22.166	TRUE
ebi-PL179-GCST90277288	Phosphatidylcholine (16:0_20:5) levels	rs2041149	12	101,716,327	G	A	0.4773	-0.0787	0.0168	2.7253E-06	7,173	22.035	TRUE
ebi-PL179-GCST90277288	Phosphatidylcholine (16:0_20:5) levels	rs7947954	11	42,657,198	C	T	0.1547	0.1078	0.0230	2.8047E-06	7,173	21.980	TRUE
ebi-PL179-GCST90277288	Phosphatidylcholine (16:0_20:5) levels	rs102274	11	61,790,354	C	T	0.4087	-0.3726	0.0165	4.3400E-109	7,173	509.866	TRUE
ebi-PL179-GCST90277288	Phosphatidylcholine (16:0_20:5) levels	rs28569162	11	87,975,724	G	A	0.1313	0.1153	0.0252	4.6844E-06	7,173	20.994	TRUE
ebi-PL179-GCST90277288	Phosphatidylcholine (16:0_20:5) levels	rs62025936	16	10,434,179	G	A	0.1649	0.1206	0.0227	1.1834E-07	7,173	28.103	TRUE
ebi-PL179-GCST90277288	Phosphatidylcholine (16:0_20:5) levels	rs3018617	11	62,435,462	G	A	0.3015	-0.0935	0.0188	7.0829E-07	7,173	24.632	TRUE
ebi-PL179-GCST90277288	Phosphatidylcholine (16:0_20:5) levels	rs59839417	3	39,249,420	A	G	0.2057	-0.0991	0.0208	2.0231E-06	7,173	22.608	TRUE
ebi-PL179-GCST90277288	Phosphatidylcholine (16:0_20:5) levels	rs113394924	11	62,314,821	G	T	0.0873	0.1407	0.0297	2.2767E-06	7,173	22.383	TRUE
ebi-PL179-GCST90277288	Phosphatidylcholine (16:0_20:5) levels	rs34822092	2	52,123,601	A	T	0.0225	-0.2832	0.0564	5.2143E-07	7,173	25.227	TRUE
ebi-PL179-GCST90277288	Phosphatidylcholine (16:0_20:5) levels	rs7780677	7	132,693,734	A	C	0.4090	-0.0862	0.0170	4.3317E-07	7,173	25.586	TRUE
ebi-PL179-GCST90277288	Phosphatidylcholine (16:0_20:5) levels	rs74754540	11	61,948,885	A	G	0.0492	-0.1890	0.0389	1.1805E-06	7,173	23.646	TRUE
ebi-PL179-GCST90277289	Phosphatidylcholine (16:0_22:4) levels	rs12528390	6	148,952,703	A	G	0.0557	0.1818	0.0374	1.2187E-06	6,874	23.586	TRUE
ebi-PL179-GCST90277289	Phosphatidylcholine (16:0_22:4) levels	rs570929	11	88,318,708	C	G	0.5482	-0.0787	0.0170	3.9767E-06	6,874	21.309	TRUE
ebi-PL179-GCST90277289	Phosphatidylcholine (16:0_22:4) levels	rs4856561	3	85,098,584	A	C	0.0467	0.2118	0.0402	1.4556E-07	6,874	27.703	TRUE
ebi-PL179-GCST90277289	Phosphatidylcholine (16:0_22:4) levels	rs4693564	4	83,110,085	C	A	0.8308	0.1152	0.0235	9.1868E-07	6,874	24.133	TRUE
ebi-PL179-GCST90277289	Phosphatidylcholine (16:0_22:4) levels	rs174551	11	61,806,212	C	T	0.4062	-0.2898	0.0171	3.6657E-63	6,874	287.293	TRUE
ebi-PL179-GCST90277289	Phosphatidylcholine (16:0_22:4) levels	rs6713301	2	30,362,772	T	C	0.0100	0.4212	0.0868	1.2546E-06	6,874	23.530	TRUE
ebi-PL179-GCST90277289	Phosphatidylcholine (16:0_22:4) levels	rs1393144	5	126,004,183	A	C	0.1725	-0.1038	0.0225	3.9939E-06	6,874	21.301	TRUE
ebi-PL179-GCST90277289	Phosphatidylcholine (16:0_22:4) levels	rs7007401	8	84,225,835	C	T	0.2822	-0.0886	0.0189	2.9461E-06	6,874	21.886	TRUE
ebi-PL179-GCST90277289	Phosphatidylcholine (16:0_22:4) levels	rs35040911	5	83,594,473	T	C	0.1614	-0.1120	0.0231	1.3408E-06	6,874	23.403	TRUE
ebi-PL179-GCST90277289	Phosphatidylcholine (16:0_22:4) levels	rs192523409	12	107,709,655	C	T	0.0249	0.2768	0.0557	6.8920E-07	6,874	24.689	TRUE
ebi-PL179-GCST90277289	Phosphatidylcholine (16:0_22:4) levels	rs4686798	3	186,727,647	T	C	0.3578	-0.0859	0.0175	9.6481E-07	6,874	24.039	TRUE
ebi-PL179-GCST90277289	Phosphatidylcholine (16:0_22:4) levels	rs115448758	4	136,353,788	C	T	0.0492	0.2005	0.0404	7.1079E-07	6,874	24.632	TRUE
ebi-PL179-GCST90277289	Phosphatidylcholine (16:0_22:4) levels	rs2730612	7	43,872,241	G	T	0.5957	0.0815	0.0174	2.9353E-06	6,874	21.893	TRUE
ebi-PL179-GCST90277289	Phosphatidylcholine (16:0_22:4) levels	rs1360225	9	23,713,150	C	T	0.2022	0.1023	0.0213	1.6565E-06	6,874	22.995	TRUE
ebi-PL179-GCST90277289	Phosphatidylcholine (16:0_22:4) levels	rs77730345	16	2,352,549	A	G	0.0386	0.2127	0.0452	2.5256E-06	6,874	22.183	TRUE
ebi-PL179-GCST90277289	Phosphatidylcholine (16:0_22:4) levels	rs140028072	9	6,890,384	T	C	0.0344	-0.2173	0.0474	4.6467E-06	6,874	21.010	TRUE
ebi-PL179-GCST90277289	Phosphatidylcholine (16:0_22:4) levels	rs61008604	2	101,652,331	T	G	0.2488	-0.1160	0.0198	5.2367E-09	6,874	34.184	TRUE
ebi-PL179-GCST90277290	Phosphatidylcholine (16:0_22:5) levels	rs73019765	6	160,908,659	C	T	0.1425	0.1121	0.0237	2.3081E-06	7,172	22.355	TRUE
ebi-PL179-GCST90277290	Phosphatidylcholine (16:0_22:5) levels	rs146509239	18	54,253,269	C	G	0.0161	0.3164	0.0673	2.6018E-06	7,172	22.124	TRUE
ebi-PL179-GCST90277290	Phosphatidylcholine (16:0_22:5) levels	rs62142428	2	35,106,778	A	C	0.0311	0.2362	0.0494	1.7719E-06	7,172	22.865	TRUE
ebi-PL179-GCST90277290	Phosphatidylcholine (16:0_22:5) levels	rs1800574	12	120,979,061	T	C	0.0457	0.2085	0.0397	1.5119E-07	7,172	27.628	TRUE

Lipid GWAS ID	Lipid species	SNP	Chromosome	Position	Effect allele	Other allele	Effect-allele frequency	Beta	SE	P value	Sample size	F statistic	F > 10
ebi-PL179-GCST90277290	Phosphatidylcholine (16:0_22:5) levels	rs2613167	4	30,161,140	T	G	0.3731	0.0808	0.0177	4.8307E-06	7,172	20.935	TRUE
ebi-PL179-GCST90277290	Phosphatidylcholine (16:0_22:5) levels	rs61653779	16	20,568,904	C	T	0.0201	0.2759	0.0602	4.6895E-06	7,172	20.990	TRUE
ebi-PL179-GCST90277290	Phosphatidylcholine (16:0_22:5) levels	rs7805803	7	50,185,795	A	G	0.4016	0.0807	0.0171	2.3303E-06	7,172	22.336	TRUE
ebi-PL179-GCST90277290	Phosphatidylcholine (16:0_22:5) levels	rs3096698	6	32,139,362	C	T	0.6310	-0.0836	0.0173	1.3880E-06	7,172	23.337	TRUE
ebi-PL179-GCST90277290	Phosphatidylcholine (16:0_22:5) levels	rs147057045	1	34,932,549	T	C	0.0153	-0.3409	0.0725	2.6290E-06	7,172	22.105	TRUE
ebi-PL179-GCST90277290	Phosphatidylcholine (16:0_22:5) levels	rs147797702	18	52,743,009	C	T	0.0094	-0.4773	0.0891	8.6445E-08	7,172	28.713	TRUE
ebi-PL179-GCST90277290	Phosphatidylcholine (16:0_22:5) levels	rs174533	11	61,781,553	A	G	0.4086	-0.4130	0.0163	5.0752E-135	7,172	638.416	TRUE
ebi-PL179-GCST90277290	Phosphatidylcholine (16:0_22:5) levels	rs80197029	12	78,247,210	T	G	0.0110	0.3921	0.0814	1.4816E-06	7,172	23.208	TRUE
ebi-PL179-GCST90277290	Phosphatidylcholine (16:0_22:5) levels	rs3019200	11	61,481,911	A	C	0.8031	-0.1086	0.0214	3.7680E-07	7,172	25.855	TRUE
ebi-PL179-GCST90277290	Phosphatidylcholine (16:0_22:5) levels	rs9918362	6	10,999,417	C	T	0.4581	0.0846	0.0168	5.0890E-07	7,172	25.274	TRUE
ebi-PL179-GCST90277290	Phosphatidylcholine (16:0_22:5) levels	rs508049	11	68,908,029	T	C	0.0980	-0.1669	0.0286	5.6162E-09	7,172	34.046	TRUE
ebi-PL179-GCST90277290	Phosphatidylcholine (16:0_22:5) levels	rs62229686	21	43,971,391	T	C	0.0391	-0.2463	0.0420	4.5384E-09	7,172	34.461	TRUE
ebi-PL179-GCST90277291	Phosphatidylcholine (16:0_22:6) levels	rs10498720	6	24,353,208	C	A	0.0294	0.2620	0.0503	2.0112E-07	7,170	27.073	TRUE
ebi-PL179-GCST90277291	Phosphatidylcholine (16:0_22:6) levels	rs61900787	11	42,654,689	C	T	0.4146	0.0786	0.0169	3.3372E-06	7,170	21.645	TRUE
ebi-PL179-GCST90277291	Phosphatidylcholine (16:0_22:6) levels	rs174544	11	61,800,281	A	C	0.3798	-0.1267	0.0173	2.3044E-13	7,170	53.928	TRUE
ebi-PL179-GCST90277291	Phosphatidylcholine (16:0_22:6) levels	rs4937784	11	133,501,713	T	C	0.1359	-0.1194	0.0248	1.5553E-06	7,170	23.115	TRUE
ebi-PL179-GCST90277291	Phosphatidylcholine (16:0_22:6) levels	rs77304558	5	153,118,411	C	T	0.0021	-0.9149	0.1961	3.1416E-06	7,170	21.760	TRUE
ebi-PL179-GCST90277291	Phosphatidylcholine (16:0_22:6) levels	rs2075083	7	17,680,332	T	G	0.5811	0.0811	0.0168	1.4215E-06	7,170	23.289	TRUE
ebi-PL179-GCST90277291	Phosphatidylcholine (16:0_22:6) levels	rs79508872	14	78,360,286	A	C	0.0202	-0.2758	0.0598	4.1356E-06	7,170	21.231	TRUE
ebi-PL179-GCST90277291	Phosphatidylcholine (16:0_22:6) levels	rs57634784	2	158,238,814	T	C	0.3310	-0.0874	0.0176	6.9257E-07	7,170	24.678	TRUE
ebi-PL179-GCST90277291	Phosphatidylcholine (16:0_22:6) levels	rs117061362	15	101,196,347	T	G	0.0449	0.2146	0.0411	1.8104E-07	7,170	27.278	TRUE
ebi-PL179-GCST90277291	Phosphatidylcholine (16:0_22:6) levels	rs3775850	4	119,529,863	A	G	0.2042	-0.0957	0.0208	4.1241E-06	7,170	21.238	TRUE
ebi-PL179-GCST90277291	Phosphatidylcholine (16:0_22:6) levels	rs117398583	10	125,205,699	A	G	0.0317	-0.2428	0.0509	1.8829E-06	7,170	22.746	TRUE
ebi-PL179-GCST90277291	Phosphatidylcholine (16:0_22:6) levels	rs10150075	14	72,571,809	T	C	0.0530	-0.1912	0.0382	5.8836E-07	7,170	24.994	TRUE
ebi-PL179-GCST90277292	Phosphatidylcholine (16:1_18:0) levels	rs2609122	2	6,080,522	C	G	0.0240	-0.2750	0.0572	1.5507E-06	6,524	23.123	TRUE
ebi-PL179-GCST90277292	Phosphatidylcholine (16:1_18:0) levels	rs76447935	19	58,583,007	T	C	0.0230	0.3069	0.0610	4.9421E-07	6,524	25.335	TRUE
ebi-PL179-GCST90277292	Phosphatidylcholine (16:1_18:0) levels	rs76712623	4	131,189,906	A	G	0.0109	-0.4164	0.0838	6.8383E-07	6,524	24.705	TRUE
ebi-PL179-GCST90277292	Phosphatidylcholine (16:1_18:0) levels	rs9934078	16	78,478,626	C	A	0.2708	-0.0953	0.0199	1.7414E-06	6,524	22.901	TRUE
ebi-PL179-GCST90277292	Phosphatidylcholine (16:1_18:0) levels	rs1533362	4	17,072,136	A	C	0.1455	0.1138	0.0246	3.8864E-06	6,524	21.355	TRUE
ebi-PL179-GCST90277292	Phosphatidylcholine (16:1_18:0) levels	rs174559	11	61,814,184	A	G	0.3140	0.0952	0.0190	5.3914E-07	6,524	25.167	TRUE
ebi-PL179-GCST90277292	Phosphatidylcholine (16:1_18:0) levels	rs117402498	21	14,678,032	T	C	0.0470	0.1986	0.0413	1.5881E-06	6,524	23.080	TRUE
ebi-PL179-GCST90277293	Phosphatidylcholine (16:1_18:1) levels	rs56316064	3	187,394,534	G	T	0.2919	-0.0902	0.0188	1.5789E-06	7,166	23.086	TRUE
ebi-PL179-GCST90277293	Phosphatidylcholine (16:1_18:1) levels	rs146934967	18	15,033,501	T	C	0.0131	0.4153	0.0737	1.8335E-08	7,166	31.733	TRUE
ebi-PL179-GCST90277293	Phosphatidylcholine (16:1_18:1) levels	rs5865	2	97,756,543	T	C	0.7115	0.0873	0.0184	2.1312E-06	7,166	22.508	TRUE
ebi-PL179-GCST90277293	Phosphatidylcholine (16:1_18:1) levels	rs145551604	7	107,240,375	T	C	0.0733	0.1547	0.0325	2.0280E-06	7,166	22.604	TRUE
ebi-PL179-GCST90277293	Phosphatidylcholine (16:1_18:1) levels	rs8033940	15	58,432,643	A	G	0.3367	0.0938	0.0180	2.0204E-07	7,166	27.065	TRUE
ebi-PL179-GCST90277293	Phosphatidylcholine (16:1_18:1) levels	rs4447106	10	100,600,003	A	G	0.1410	0.1280	0.0242	1.2908E-07	7,166	27.933	TRUE
ebi-PL179-GCST90277293	Phosphatidylcholine (16:1_18:1) levels	rs139935251	11	30,070,603	C	T	0.0030	-0.7228	0.1579	4.7808E-06	7,166	20.953	TRUE
ebi-PL179-GCST90277293	Phosphatidylcholine (16:1_18:1) levels	rs56156922	16	56,953,457	C	T	0.2787	0.1009	0.0186	5.9458E-08	7,166	29.442	TRUE
ebi-PL179-GCST90277293	Phosphatidylcholine (16:1_18:1) levels	rs72834725	10	62,688,203	T	C	0.0426	0.1964	0.0420	2.9246E-06	7,166	21.898	TRUE
ebi-PL179-GCST90277293	Phosphatidylcholine (16:1_18:1) levels	rs174560	11	61,814,292	C	T	0.3829	0.1757	0.0172	2.2814E-24	7,166	104.519	TRUE
ebi-PL179-GCST90277293	Phosphatidylcholine (16:1_18:1) levels	rs11189347	10	81,316,583	A	G	0.1327	-0.1165	0.0248	2.5703E-06	7,166	22.147	TRUE
ebi-PL179-GCST90277293	Phosphatidylcholine (16:1_18:1) levels	rs603424	10	100,315,722	A	G	0.1213	-0.1764	0.0260	1.2082E-11	7,166	46.105	TRUE
ebi-PL179-GCST90277294	Phosphatidylcholine (16:1_18:2) levels	rs7783799	7	6,533,456	G	T	0.3077	0.0860	0.0183	2.6341E-06	7,165	22.099	TRUE
ebi-PL179-GCST90277294	Phosphatidylcholine (16:1_18:2) levels	rs4122352	16	15,080,714	A	G	0.2811	-0.0878	0.0184	1.9574E-06	7,165	22.672	TRUE
ebi-PL179-GCST90277294	Phosphatidylcholine (16:1_18:2) levels	rs6876235	5	86,303,978	C	T	0.9049	0.1311	0.0284	3.8765E-06	7,165	21.358	TRUE
ebi-PL179-GCST90277294	Phosphatidylcholine (16:1_18:2) levels	rs216958	16	60,201,094	G	A	0.6168	-0.0795	0.0173	4.4717E-06	7,165	21.082	TRUE
ebi-PL179-GCST90277294	Phosphatidylcholine (16:1_18:2) levels	rs9480322	6	156,383,631	C	T	0.0372	-0.2198	0.0445	8.1598E-07	7,165	24.361	TRUE
ebi-PL179-GCST90277294	Phosphatidylcholine (16:1_18:2) levels	rs9839380	3	26,850,125	G	A	0.3348	0.0960	0.0177	6.3691E-08	7,165	29.309	TRUE
ebi-PL179-GCST90277294	Phosphatidylcholine (16:1_18:2) levels	rs62104079	18	71,672,897	G	C	0.2321	0.1039	0.0201	2.3370E-07	7,165	26.781	TRUE
ebi-PL179-GCST90277295	Phosphatidylcholine (16:1_20:4) levels	rs56317095	7	150,431,359	A	G	0.0311	0.2692	0.0561	1.6202E-06	5,564	23.047	TRUE
ebi-PL179-GCST90277295	Phosphatidylcholine (16:1_20:4) levels	rs56246023	3	159,995,286	T	G	0.1381	0.1372	0.0276	6.5766E-07	5,564	24.790	TRUE
ebi-PL179-GCST90277295	Phosphatidylcholine (16:1_20:4) levels	rs75878220	1	163,867,608	C	G	0.0255	-0.3063	0.0618	7.3157E-07	5,564	24.584	TRUE
ebi-PL179-GCST90277295	Phosphatidylcholine (16:1_20:4) levels	rs695112	11	75,745,535	T	A	0.2408	-0.1087	0.0225	1.3344E-06	5,564	23.424	TRUE
ebi-PL179-GCST90277295	Phosphatidylcholine (16:1_20:4) levels	rs10848122	12	130,508,437	C	G	0.7776	-0.1099	0.0233	2.5361E-06	5,564	22.183	TRUE
ebi-PL179-GCST90277295	Phosphatidylcholine (16:1_20:4) levels	rs174535	11	61,783,884	C	T	0.4091	-0.3277	0.0192	5.3348E-64	5,564	292.683	TRUE
ebi-PL179-GCST90277295	Phosphatidylcholine (16:1_20:4) levels	rs2446624	1	161,649,571	T	C	0.0854	0.1634	0.0348	2.7960E-06	5,564	21.995	TRUE
ebi-PL179-GCST90277295	Phosphatidylcholine (16:1_20:4) levels	rs6545883	2	61,545,122	G	A	0.6196	0.0953	0.0196	1.1660E-06	5,564	23.683	TRUE

Lipid GWAS ID	Lipid species	SNP	Chromosome	Position	Effect allele	Other allele	Effect-allele frequency	Beta	SE	P value	Sample size	F statistic	F > 10
ebi-PL179-GCST90277295	Phosphatidylcholine (16:1_20:4) levels	rs11650558	17	21,397,913	T	C	0.5368	0.0919	0.0191	1.4908E-06	5,564	23.207	TRUE
ebi-PL179-GCST90277295	Phosphatidylcholine (16:1_20:4) levels	rs11659363	18	22,904,677	G	T	0.2423	0.1039	0.0221	2.5963E-06	5,564	22.139	TRUE
ebi-PL179-GCST90277295	Phosphatidylcholine (16:1_20:4) levels	rs192436652	19	54,449,569	T	C	0.0526	0.2030	0.0433	2.7960E-06	5,564	21.996	TRUE
ebi-PL179-GCST90277296	Phosphatidylcholine (17:0_18:1) levels	rs61737926	16	87,644,718	T	C	0.0027	-0.7541	0.1636	4.0978E-06	7,073	21.251	TRUE
ebi-PL179-GCST90277296	Phosphatidylcholine (17:0_18:1) levels	rs139070595	1	247,024,993	A	T	0.0052	0.5918	0.1224	1.3502E-06	7,073	23.386	TRUE
ebi-PL179-GCST90277296	Phosphatidylcholine (17:0_18:1) levels	rs1106933	6	166,616,136	G	A	0.1362	0.1248	0.0245	3.4374E-07	7,073	26.034	TRUE
ebi-PL179-GCST90277296	Phosphatidylcholine (17:0_18:1) levels	rs78308012	9	137,586,795	G	T	0.1032	0.1532	0.0288	1.1091E-07	7,073	28.231	TRUE
ebi-PL179-GCST90277296	Phosphatidylcholine (17:0_18:1) levels	rs141245990	18	44,024,688	T	C	0.0260	-0.2474	0.0536	3.9682E-06	7,073	21.314	TRUE
ebi-PL179-GCST90277296	Phosphatidylcholine (17:0_18:1) levels	rs77534789	4	130,689,035	A	C	0.0376	-0.2076	0.0451	4.1222E-06	7,073	21.239	TRUE
ebi-PL179-GCST90277296	Phosphatidylcholine (17:0_18:1) levels	rs4692808	4	170,196,755	A	G	0.4363	0.0802	0.0169	2.0803E-06	7,073	22.555	TRUE
ebi-PL179-GCST90277296	Phosphatidylcholine (17:0_18:1) levels	rs7350789	15	58,387,469	A	G	0.3928	0.1126	0.0171	4.4633E-11	7,073	43.532	TRUE
ebi-PL179-GCST90277296	Phosphatidylcholine (17:0_18:1) levels	rs143506888	12	14,115,139	T	A	0.0184	0.2965	0.0643	4.1285E-06	7,073	21.236	TRUE
ebi-PL179-GCST90277296	Phosphatidylcholine (17:0_18:1) levels	rs2003890	20	61,233,003	G	A	0.2233	-0.0930	0.0203	4.5737E-06	7,073	21.038	TRUE
ebi-PL179-GCST90277296	Phosphatidylcholine (17:0_18:1) levels	rs140419225	20	61,849,802	A	G	0.0042	0.6462	0.1390	3.3972E-06	7,073	21.613	TRUE
ebi-PL179-GCST90277297	Phosphatidylcholine (17:0_18:2) levels	rs192537901	4	113,463,133	C	T	0.0260	-0.2572	0.0527	1.0956E-06	7,170	23.791	TRUE
ebi-PL179-GCST90277297	Phosphatidylcholine (17:0_18:2) levels	rs7437408	4	149,246,245	G	A	0.4779	0.0771	0.0168	4.5363E-06	7,170	21.054	TRUE
ebi-PL179-GCST90277297	Phosphatidylcholine (17:0_18:2) levels	rs73058910	7	14,912,136	C	T	0.0633	0.1689	0.0365	3.7999E-06	7,170	21.396	TRUE
ebi-PL179-GCST90277297	Phosphatidylcholine (17:0_18:2) levels	rs12548274	8	133,696,200	G	A	0.1704	0.1039	0.0221	2.6338E-06	7,170	22.101	TRUE
ebi-PL179-GCST90277297	Phosphatidylcholine (17:0_18:2) levels	rs2126260	8	9,327,571	C	T	0.7488	0.0913	0.0191	1.7193E-06	7,170	22.922	TRUE
ebi-PL179-GCST90277297	Phosphatidylcholine (17:0_18:2) levels	rs1168104	1	62,662,654	G	A	0.7377	0.0861	0.0188	4.9611E-06	7,170	20.883	TRUE
ebi-PL179-GCST90277297	Phosphatidylcholine (17:0_18:2) levels	rs422137	15	58,416,983	A	G	0.4926	0.0854	0.0168	3.6644E-07	7,170	25.911	TRUE
ebi-PL179-GCST90277297	Phosphatidylcholine (17:0_18:2) levels	rs57518551	2	42,156,698	C	T	0.1316	-0.1252	0.0246	3.7895E-07	7,170	25.845	TRUE
ebi-PL179-GCST90277297	Phosphatidylcholine (17:0_18:2) levels	rs115686032	5	40,282,763	T	C	0.0392	-0.1991	0.0428	3.3950E-06	7,170	21.612	TRUE
ebi-PL179-GCST90277297	Phosphatidylcholine (17:0_18:2) levels	rs4840981	8	8,532,922	G	C	0.9870	0.3388	0.0735	4.0526E-06	7,170	21.271	TRUE
ebi-PL179-GCST90277297	Phosphatidylcholine (17:0_18:2) levels	rs72747028	1	211,733,797	C	T	0.0727	-0.1510	0.0324	3.2487E-06	7,170	21.697	TRUE
ebi-PL179-GCST90277297	Phosphatidylcholine (17:0_18:2) levels	rs34489224	7	100,927,939	G	C	0.2659	0.0883	0.0191	3.7509E-06	7,170	21.419	TRUE
ebi-PL179-GCST90277297	Phosphatidylcholine (17:0_18:2) levels	rs78592970	19	44,938,026	T	G	0.1275	-0.1283	0.0255	5.1794E-07	7,170	25.241	TRUE
ebi-PL179-GCST90277298	Phosphatidylcholine (17:0_20:4) levels	rs600518	11	75,744,328	T	A	0.2323	-0.1130	0.0199	1.4232E-08	7,106	32.235	TRUE
ebi-PL179-GCST90277298	Phosphatidylcholine (17:0_20:4) levels	rs56399423	5	132,336,964	C	T	0.3234	0.0844	0.0179	2.4854E-06	7,106	22.212	TRUE
ebi-PL179-GCST90277298	Phosphatidylcholine (17:0_20:4) levels	rs143309746	1	27,599,934	G	A	0.0225	0.2702	0.0591	4.9439E-06	7,106	20.889	TRUE
ebi-PL179-GCST90277298	Phosphatidylcholine (17:0_20:4) levels	rs3110971	5	38,490,169	G	A	0.2079	0.0988	0.0207	1.7790E-06	7,106	22.855	TRUE
ebi-PL179-GCST90277298	Phosphatidylcholine (17:0_20:4) levels	rs585002	9	15,293,515	C	T	0.8620	0.1146	0.0242	2.3343E-06	7,106	22.333	TRUE
ebi-PL179-GCST90277298	Phosphatidylcholine (17:0_20:4) levels	rs117580248	9	137,477,951	A	G	0.1104	0.1308	0.0279	2.7762E-06	7,106	21.999	TRUE
ebi-PL179-GCST90277298	Phosphatidylcholine (17:0_20:4) levels	rs575960806	8	114,520,741	A	G	0.0138	-0.3515	0.0733	1.6477E-06	7,106	23.001	TRUE
ebi-PL179-GCST90277298	Phosphatidylcholine (17:0_20:4) levels	rs12928399	16	14,611,144	C	G	0.3612	-0.0856	0.0175	1.0810E-06	7,106	23.820	TRUE
ebi-PL179-GCST90277298	Phosphatidylcholine (17:0_20:4) levels	rs143060591	8	85,171,256	T	C	0.0743	-0.1499	0.0324	3.7182E-06	7,106	21.438	TRUE
ebi-PL179-GCST90277298	Phosphatidylcholine (17:0_20:4) levels	rs7945579	11	63,618,106	G	A	0.3277	-0.0929	0.0180	2.4397E-07	7,106	26.701	TRUE
ebi-PL179-GCST90277298	Phosphatidylcholine (17:0_20:4) levels	rs635196	18	44,179,195	C	T	0.1843	0.0999	0.0216	3.9137E-06	7,106	21.339	TRUE
ebi-PL179-GCST90277298	Phosphatidylcholine (17:0_20:4) levels	rs58983615	11	62,053,609	T	A	0.0208	-0.3286	0.0592	2.9460E-08	7,106	30.809	TRUE
ebi-PL179-GCST90277298	Phosphatidylcholine (17:0_20:4) levels	rs12417747	11	62,060,922	A	G	0.1148	-0.1456	0.0266	4.6031E-08	7,106	29.941	TRUE
ebi-PL179-GCST90277298	Phosphatidylcholine (17:0_20:4) levels	rs12429208	13	37,767,109	T	C	0.2739	0.0882	0.0191	3.7603E-06	7,106	21.414	TRUE
ebi-PL179-GCST90277298	Phosphatidylcholine (17:0_20:4) levels	rs73161879	7	152,050,140	A	G	0.1812	-0.1031	0.0223	3.7673E-06	7,106	21.413	TRUE
ebi-PL179-GCST90277298	Phosphatidylcholine (17:0_20:4) levels	rs2398428	12	128,751,021	G	A	0.2628	-0.0968	0.0197	8.9581E-07	7,106	24.181	TRUE
ebi-PL179-GCST90277298	Phosphatidylcholine (17:0_20:4) levels	rs142082962	6	158,517,411	A	C	0.0779	-0.1442	0.0311	3.4636E-06	7,106	21.573	TRUE
ebi-PL179-GCST90277298	Phosphatidylcholine (17:0_20:4) levels	rs74452899	12	88,630,462	C	T	0.0106	-0.4457	0.0883	4.6386E-07	7,106	25.454	TRUE
ebi-PL179-GCST90277298	Phosphatidylcholine (17:0_20:4) levels	rs6083838	20	25,397,682	G	A	0.5356	-0.0775	0.0169	4.5068E-06	7,106	21.068	TRUE
ebi-PL179-GCST90277298	Phosphatidylcholine (17:0_20:4) levels	rs11088781	21	20,132,472	A	G	0.9144	0.1446	0.0306	2.2714E-06	7,106	22.386	TRUE
ebi-PL179-GCST90277265	Phosphatidylcholine (18:0_0:0) levels	rs145366219	9	33,013,634	G	A	0.0668	-0.1585	0.0337	2.6762E-06	7,173	22.070	TRUE
ebi-PL179-GCST90277265	Phosphatidylcholine (18:0_0:0) levels	rs78392466	6	101,082,967	T	A	0.0377	-0.2088	0.0439	1.9807E-06	7,173	22.650	TRUE
ebi-PL179-GCST90277265	Phosphatidylcholine (18:0_0:0) levels	rs117140365	10	74,861,163	C	G	0.0160	-0.3290	0.0672	9.8333E-07	7,173	24.001	TRUE
ebi-PL179-GCST90277265	Phosphatidylcholine (18:0_0:0) levels	rs77751297	12	95,853,480	T	G	0.1648	0.1127	0.0226	6.4135E-07	7,173	24.826	TRUE
ebi-PL179-GCST90277265	Phosphatidylcholine (18:0_0:0) levels	rs143623180	1	206,781,181	T	C	0.0630	0.1584	0.0346	4.9033E-06	7,173	20.905	TRUE
ebi-PL179-GCST90277265	Phosphatidylcholine (18:0_0:0) levels	rs10095602	8	53,669,039	T	C	0.0518	0.1759	0.0380	3.7860E-06	7,173	21.402	TRUE
ebi-PL179-GCST90277265	Phosphatidylcholine (18:0_0:0) levels	rs1146461	1	94,942,936	A	G	0.4628	-0.0799	0.0166	1.5689E-06	7,173	23.098	TRUE
ebi-PL179-GCST90277265	Phosphatidylcholine (18:0_0:0) levels	rs59634655	16	78,457,680	T	G	0.1633	-0.1080	0.0229	2.4565E-06	7,173	22.234	TRUE
ebi-PL179-GCST90277265	Phosphatidylcholine (18:0_0:0) levels	rs1018070	8	18,416,117	A	T	0.0611	0.1775	0.0355	5.8272E-07	7,173	25.015	TRUE
ebi-PL179-GCST90277265	Phosphatidylcholine (18:0_0:0) levels	rs79713567	14	36,841,591	T	C	0.0504	-0.1866	0.0408	4.7540E-06	7,173	20.964	TRUE
ebi-PL179-GCST90277265	Phosphatidylcholine (18:0_0:0) levels	rs11582724	1	201,132,736	T	C	0.5751	-0.0783	0.0171	4.6413E-06	7,173	21.010	TRUE

Lipid GWAS ID	Lipid species	SNP	Chromosome	Position	Effect allele	Other allele	Effect-allele frequency	Beta	SE	P value	Sample size	F statistic	F > 10
ebi-PL179-GCST90277265	Phosphatidylcholine (18:0_0:0) levels	rs186790715	7	124,474,397	C	T	0.0168	0.3393	0.0657	2.4516E-07	7,173	26.689	TRUE
ebi-PL179-GCST90277265	Phosphatidylcholine (18:0_0:0) levels	rs76309137	7	95,573,476	T	C	0.0127	-0.3511	0.0757	3.6270E-06	7,173	21.484	TRUE
ebi-PL179-GCST90277265	Phosphatidylcholine (18:0_0:0) levels	rs7322909	13	81,451,428	A	C	0.5314	-0.0787	0.0168	2.9995E-06	7,173	21.850	TRUE
ebi-PL179-GCST90277265	Phosphatidylcholine (18:0_0:0) levels	rs77900286	21	40,397,531	C	T	0.0081	-0.4997	0.0973	2.8806E-07	7,173	26.376	TRUE
ebi-PL179-GCST90277265	Phosphatidylcholine (18:0_0:0) levels	rs76023792	20	9,964,250	A	T	0.0338	0.2174	0.0470	3.8652E-06	7,173	21.362	TRUE
ebi-PL179-GCST90277299	Phosphatidylcholine (18:0_18:1) levels	rs2797620	1	94,975,736	T	C	0.3326	-0.1023	0.0176	6.2302E-09	7,173	33.842	TRUE
ebi-PL179-GCST90277299	Phosphatidylcholine (18:0_18:1) levels	rs16916055	11	91,426,863	C	T	0.0088	0.4211	0.0915	4.2795E-06	7,173	21.166	TRUE
ebi-PL179-GCST90277299	Phosphatidylcholine (18:0_18:1) levels	rs34833702	1	218,198,038	T	C	0.0521	0.1859	0.0380	9.9636E-07	7,173	23.976	TRUE
ebi-PL179-GCST90277299	Phosphatidylcholine (18:0_18:1) levels	rs8034802	15	58,432,593	A	T	0.3106	0.0856	0.0183	2.9450E-06	7,173	21.886	TRUE
ebi-PL179-GCST90277299	Phosphatidylcholine (18:0_18:1) levels	rs181751380	7	50,773,538	A	G	0.0337	0.2277	0.0471	1.3448E-06	7,173	23.395	TRUE
ebi-PL179-GCST90277299	Phosphatidylcholine (18:0_18:1) levels	rs151331635	16	32,012,667	T	A	0.0206	0.3024	0.0641	2.4252E-06	7,173	22.259	TRUE
ebi-PL179-GCST90277299	Phosphatidylcholine (18:0_18:1) levels	rs2708081	12	121,025,485	C	T	0.4903	-0.0826	0.0168	8.3751E-07	7,173	24.310	TRUE
ebi-PL179-GCST90277299	Phosphatidylcholine (18:0_18:1) levels	rs2183640	1	232,142,866	A	G	0.1616	-0.1072	0.0227	2.2960E-06	7,173	22.365	TRUE
ebi-PL179-GCST90277299	Phosphatidylcholine (18:0_18:1) levels	rs77191869	16	70,566,761	C	T	0.0369	-0.2074	0.0445	3.1528E-06	7,173	21.754	TRUE
ebi-PL179-GCST90277299	Phosphatidylcholine (18:0_18:1) levels	rs150862068	11	44,029,081	T	C	0.0029	-0.7417	0.1607	3.9690E-06	7,173	21.310	TRUE
ebi-PL179-GCST90277299	Phosphatidylcholine (18:0_18:1) levels	rs7252513	19	1,991,747	A	G	0.0557	0.1723	0.0371	3.4789E-06	7,173	21.564	TRUE
ebi-PL179-GCST90277299	Phosphatidylcholine (18:0_18:1) levels	rs117207178	12	34,480,655	T	C	0.0972	0.1380	0.0286	1.3831E-06	7,173	23.342	TRUE
ebi-PL179-GCST90277299	Phosphatidylcholine (18:0_18:1) levels	rs535686	3	155,568,069	C	T	0.0336	-0.2215	0.0480	3.9952E-06	7,173	21.299	TRUE
ebi-PL179-GCST90277299	Phosphatidylcholine (18:0_18:1) levels	rs73215839	12	92,131,416	T	C	0.0335	0.2407	0.0473	3.6170E-07	7,173	25.935	TRUE
ebi-PL179-GCST90277299	Phosphatidylcholine (18:0_18:1) levels	rs2169387	8	9,323,885	G	A	0.8500	0.1073	0.0234	4.8236E-06	7,173	20.937	TRUE
ebi-PL179-GCST90277299	Phosphatidylcholine (18:0_18:1) levels	rs11197775	10	116,636,627	C	T	0.5317	0.0767	0.0167	4.5514E-06	7,173	21.048	TRUE
ebi-PL179-GCST90277299	Phosphatidylcholine (18:0_18:1) levels	rs612577	1	230,188,618	C	T	0.1798	-0.1275	0.0216	3.6340E-09	7,173	34.896	TRUE
ebi-PL179-GCST90277299	Phosphatidylcholine (18:0_18:1) levels	rs4629629	5	86,296,114	T	C	0.7650	0.0939	0.0197	1.8344E-06	7,173	22.797	TRUE
ebi-PL179-GCST90277300	Phosphatidylcholine (18:0_18:2) levels	rs6678964	1	95,228,712	G	A	0.9020	0.1997	0.0278	6.8372E-13	7,174	51.776	TRUE
ebi-PL179-GCST90277300	Phosphatidylcholine (18:0_18:2) levels	rs55917360	1	211,735,543	T	C	0.0734	-0.1734	0.0321	6.8951E-08	7,174	29.153	TRUE
ebi-PL179-GCST90277300	Phosphatidylcholine (18:0_18:2) levels	rs192934615	9	80,727,726	C	T	0.0142	-0.3379	0.0723	3.0713E-06	7,174	21.808	TRUE
ebi-PL179-GCST90277300	Phosphatidylcholine (18:0_18:2) levels	rs9987289	8	9,325,848	G	A	0.8518	0.1256	0.0234	8.7451E-08	7,174	28.692	TRUE
ebi-PL179-GCST90277300	Phosphatidylcholine (18:0_18:2) levels	rs77008693	17	44,755,436	A	G	0.1388	0.1112	0.0239	3.4020E-06	7,174	21.608	TRUE
ebi-PL179-GCST90277300	Phosphatidylcholine (18:0_18:2) levels	rs73126869	4	14,739,231	G	A	0.0993	0.1330	0.0286	3.4756E-06	7,174	21.567	TRUE
ebi-PL179-GCST90277300	Phosphatidylcholine (18:0_18:2) levels	rs675849	9	15,298,668	C	T	0.8988	0.1268	0.0273	3.5009E-06	7,174	21.553	TRUE
ebi-PL179-GCST90277300	Phosphatidylcholine (18:0_18:2) levels	rs72840078	2	85,365,218	G	A	0.1021	0.1241	0.0270	4.4939E-06	7,174	21.072	TRUE
ebi-PL179-GCST90277300	Phosphatidylcholine (18:0_18:2) levels	rs1440724	12	62,017,823	A	G	0.4030	0.0796	0.0168	2.1773E-06	7,174	22.469	TRUE
ebi-PL179-GCST90277300	Phosphatidylcholine (18:0_18:2) levels	rs10889352	1	62,633,352	C	T	0.2624	-0.1009	0.0188	8.4903E-08	7,174	28.751	TRUE
ebi-PL179-GCST90277300	Phosphatidylcholine (18:0_18:2) levels	rs34031934	18	13,728,480	T	C	0.3255	-0.0843	0.0175	1.4347E-06	7,174	23.271	TRUE
ebi-PL179-GCST90277300	Phosphatidylcholine (18:0_18:2) levels	rs150457719	1	212,247,960	G	A	0.0345	-0.2183	0.0456	1.7435E-06	7,174	22.895	TRUE
ebi-PL179-GCST90277300	Phosphatidylcholine (18:0_18:2) levels	rs112121830	21	32,410,680	T	C	0.0640	-0.1638	0.0351	3.0990E-06	7,174	21.786	TRUE
ebi-PL179-GCST90277301	Phosphatidylcholine (18:0_18:3) levels	rs4846841	1	230,166,915	G	A	0.7472	0.0912	0.0197	3.7898E-06	6,692	21.404	TRUE
ebi-PL179-GCST90277301	Phosphatidylcholine (18:0_18:3) levels	rs181264690	5	122,510,180	T	G	0.2810	-0.0900	0.0194	3.5268E-06	6,692	21.538	TRUE
ebi-PL179-GCST90277301	Phosphatidylcholine (18:0_18:3) levels	rs7139079	12	120,977,490	A	G	0.5468	-0.0806	0.0174	3.4953E-06	6,692	21.557	TRUE
ebi-PL179-GCST90277301	Phosphatidylcholine (18:0_18:3) levels	rs183478874	5	176,998,593	A	C	0.0235	-0.2954	0.0593	6.5678E-07	6,692	24.783	TRUE
ebi-PL179-GCST90277301	Phosphatidylcholine (18:0_18:3) levels	rs72867166	6	41,878,684	A	T	0.1183	0.1239	0.0271	4.8731E-06	6,692	20.918	TRUE
ebi-PL179-GCST90277301	Phosphatidylcholine (18:0_18:3) levels	rs10874911	1	91,728,966	A	G	0.3943	-0.0829	0.0176	2.4138E-06	6,692	22.273	TRUE
ebi-PL179-GCST90277301	Phosphatidylcholine (18:0_18:3) levels	rs4888557	16	76,816,258	A	T	0.0190	0.3057	0.0643	2.0024E-06	6,692	22.629	TRUE
ebi-PL179-GCST90277301	Phosphatidylcholine (18:0_18:3) levels	rs75593273	5	44,740,041	T	C	0.0952	0.1382	0.0299	3.9068E-06	6,692	21.345	TRUE
ebi-PL179-GCST90277301	Phosphatidylcholine (18:0_18:3) levels	rs16987303	19	56,525,734	G	C	0.2344	-0.0970	0.0204	2.1098E-06	6,692	22.529	TRUE
ebi-PL179-GCST90277301	Phosphatidylcholine (18:0_18:3) levels	rs62122127	19	14,102,503	A	G	0.0211	0.2898	0.0632	4.6464E-06	6,692	21.010	TRUE
ebi-PL179-GCST90277302	Phosphatidylcholine (18:0_20:2) levels	rs34955778	16	15,045,737	C	T	0.4362	0.0979	0.0188	2.0053E-07	5,778	27.092	TRUE
ebi-PL179-GCST90277302	Phosphatidylcholine (18:0_20:2) levels	rs12513382	4	43,912,586	T	C	0.6209	-0.0884	0.0191	3.6779E-06	5,778	21.461	TRUE
ebi-PL179-GCST90277302	Phosphatidylcholine (18:0_20:2) levels	rs174547	11	61,803,311	C	T	0.4074	0.2823	0.0186	4.0490E-51	5,778	230.655	TRUE
ebi-PL179-GCST90277302	Phosphatidylcholine (18:0_20:2) levels	rs9671254	14	24,946,787	A	T	0.7106	-0.0951	0.0206	3.8788E-06	5,778	21.365	TRUE
ebi-PL179-GCST90277302	Phosphatidylcholine (18:0_20:2) levels	rs8068923	17	54,741,041	A	G	0.9398	0.1897	0.0397	1.8070E-06	5,778	22.835	TRUE
ebi-PL179-GCST90277302	Phosphatidylcholine (18:0_20:2) levels	rs335182	5	123,115,129	A	G	0.1551	0.1209	0.0258	2.8552E-06	5,778	21.953	TRUE
ebi-PL179-GCST90277302	Phosphatidylcholine (18:0_20:2) levels	rs1422819	5	166,671,878	G	T	0.5932	-0.0897	0.0188	1.9725E-06	5,778	22.664	TRUE
ebi-PL179-GCST90277303	Phosphatidylcholine (18:0_20:3) levels	rs3134937	6	32,238,762	T	C	0.2001	-0.0951	0.0208	4.7804E-06	7,169	20.955	TRUE
ebi-PL179-GCST90277303	Phosphatidylcholine (18:0_20:3) levels	rs4808779	19	18,294,126	G	A	0.6664	-0.0884	0.0177	5.9280E-07	7,169	24.978	TRUE
ebi-PL179-GCST90277303	Phosphatidylcholine (18:0_20:3) levels	rs11847009	14	33,476,504	A	T	0.1738	-0.1108	0.0223	6.8697E-07	7,169	24.692	TRUE
ebi-PL179-GCST90277303	Phosphatidylcholine (18:0_20:3) levels	rs72827678	5	168,832,803	G	T	0.0856	0.1426	0.0301	2.1486E-06	7,169	22.493	TRUE
ebi-PL179-GCST90277303	Phosphatidylcholine (18:0_20:3) levels	rs112534228	12	6,260,820	C	A	0.0381	0.2096	0.0447	2.7766E-06	7,169	21.999	TRUE

Lipid GWAS ID	Lipid species	SNP	Chromosome	Position	Effect allele	Other allele	Effect-allele frequency	Beta	SE	P value	Sample size	F statistic	F > 10
ebi-PL179-GCST90277303	Phosphatidylcholine (18:0_20:3) levels	rs59739041	15	58,368,884	C	T	0.0479	0.1829	0.0387	2.3236E-06	7,169	22.342	TRUE
ebi-PL179-GCST90277303	Phosphatidylcholine (18:0_20:3) levels	rs7943728	11	61,779,596	A	G	0.1123	0.2361	0.0265	6.8674E-19	7,169	79.236	TRUE
ebi-PL179-GCST90277303	Phosphatidylcholine (18:0_20:3) levels	rs192117305	5	33,853,083	T	C	0.0409	-0.1970	0.0429	4.3825E-06	7,169	21.121	TRUE
ebi-PL179-GCST90277303	Phosphatidylcholine (18:0_20:3) levels	rs140123208	5	16,863,519	G	A	0.0383	-0.2038	0.0439	3.4518E-06	7,169	21.580	TRUE
ebi-PL179-GCST90277303	Phosphatidylcholine (18:0_20:3) levels	rs151178379	9	130,575,137	A	G	0.0194	0.2892	0.0614	2.4831E-06	7,169	22.214	TRUE
ebi-PL179-GCST90277303	Phosphatidylcholine (18:0_20:3) levels	rs56374730	16	15,773,795	G	T	0.2963	0.0885	0.0184	1.4442E-06	7,169	23.259	TRUE
ebi-PL179-GCST90277303	Phosphatidylcholine (18:0_20:3) levels	rs11648397	16	78,589,971	C	G	0.0783	0.1520	0.0316	1.5936E-06	7,169	23.068	TRUE
ebi-PL179-GCST90277304	Phosphatidylcholine (18:0_20:4) levels	rs938822	3	64,950,744	C	T	0.1462	-0.1055	0.0230	4.6639E-06	7,174	21.004	TRUE
ebi-PL179-GCST90277304	Phosphatidylcholine (18:0_20:4) levels	rs12275418	11	61,012,351	A	G	0.1337	0.1244	0.0247	4.6639E-07	7,174	25.443	TRUE
ebi-PL179-GCST90277304	Phosphatidylcholine (18:0_20:4) levels	rs72789541	16	15,033,677	A	T	0.3150	-0.0966	0.0179	6.3601E-08	7,174	29.308	TRUE
ebi-PL179-GCST90277304	Phosphatidylcholine (18:0_20:4) levels	rs223841	16	57,423,359	G	A	0.1718	-0.1025	0.0219	2.8854E-06	7,174	21.930	TRUE
ebi-PL179-GCST90277304	Phosphatidylcholine (18:0_20:4) levels	rs793864	19	7,552,489	C	G	0.8176	-0.1041	0.0216	1.4998E-06	7,174	23.180	TRUE
ebi-PL179-GCST90277304	Phosphatidylcholine (18:0_20:4) levels	rs35561534	12	91,657,130	T	C	0.1065	0.1257	0.0268	2.8097E-06	7,174	21.974	TRUE
ebi-PL179-GCST90277304	Phosphatidylcholine (18:0_20:4) levels	rs2990536	10	36,620,136	T	C	0.5776	-0.0782	0.0168	3.4793E-06	7,174	21.567	TRUE
ebi-PL179-GCST90277304	Phosphatidylcholine (18:0_20:4) levels	rs9462860	6	42,979,275	C	G	0.4580	0.0762	0.0166	4.6596E-06	7,174	21.006	TRUE
ebi-PL179-GCST90277304	Phosphatidylcholine (18:0_20:4) levels	rs1219539	11	75,722,099	A	G	0.4929	-0.0877	0.0168	1.7414E-07	7,174	27.353	TRUE
ebi-PL179-GCST90277304	Phosphatidylcholine (18:0_20:4) levels	rs6860806	5	132,304,843	G	A	0.4203	0.0792	0.0169	2.7769E-06	7,174	21.995	TRUE
ebi-PL179-GCST90277304	Phosphatidylcholine (18:0_20:4) levels	rs2070895	15	58,431,740	A	G	0.2666	0.0937	0.0190	8.7333E-07	7,174	24.233	TRUE
ebi-PL179-GCST90277304	Phosphatidylcholine (18:0_20:4) levels	rs1477491	18	73,092,300	G	A	0.7891	-0.1020	0.0203	5.0570E-07	7,174	25.286	TRUE
ebi-PL179-GCST90277305	Phosphatidylcholine (18:0_20:5) levels	rs2054498	4	37,392,086	G	A	0.5378	-0.0781	0.0167	2.7821E-06	7,121	21.995	TRUE
ebi-PL179-GCST90277305	Phosphatidylcholine (18:0_20:5) levels	rs71629271	3	176,095,628	C	T	0.0635	0.1680	0.0359	2.9742E-06	7,121	21.867	TRUE
ebi-PL179-GCST90277305	Phosphatidylcholine (18:0_20:5) levels	rs102275	11	61,790,331	C	T	0.4104	-0.4195	0.0165	3.3009E-137	7,121	649.927	TRUE
ebi-PL179-GCST90277305	Phosphatidylcholine (18:0_20:5) levels	rs147051683	1	170,134,570	G	C	0.0335	0.2353	0.0467	4.6757E-07	7,121	25.438	TRUE
ebi-PL179-GCST90277305	Phosphatidylcholine (18:0_20:5) levels	rs2431163	5	174,893,516	A	G	0.0267	0.2638	0.0563	2.8108E-06	7,121	21.975	TRUE
ebi-PL179-GCST90277305	Phosphatidylcholine (18:0_20:5) levels	rs35226034	3	139,980,152	G	A	0.0399	-0.2023	0.0430	2.5709E-06	7,121	22.147	TRUE
ebi-PL179-GCST90277305	Phosphatidylcholine (18:0_20:5) levels	rs9380073	6	11,061,917	T	C	0.1231	0.1192	0.0254	2.6505E-06	7,121	22.086	TRUE
ebi-PL179-GCST90277305	Phosphatidylcholine (18:0_20:5) levels	rs9587408	13	107,595,697	C	T	0.2187	0.0920	0.0201	4.8608E-06	7,121	20.923	TRUE
ebi-PL179-GCST90277305	Phosphatidylcholine (18:0_20:5) levels	rs80116939	15	94,356,658	A	G	0.0035	-0.7648	0.1562	1.0008E-06	7,121	23.968	TRUE
ebi-PL179-GCST90277306	Phosphatidylcholine (18:0_22:5) levels	rs628035	1	230,188,399	T	G	0.2135	-0.0972	0.0207	2.7233E-06	6,805	22.037	TRUE
ebi-PL179-GCST90277306	Phosphatidylcholine (18:0_22:5) levels	rs1465411	5	170,155,391	G	A	0.5057	0.0790	0.0171	3.7032E-06	6,805	21.446	TRUE
ebi-PL179-GCST90277306	Phosphatidylcholine (18:0_22:5) levels	rs1169289	12	120,978,819	G	C	0.4700	0.1077	0.0172	3.7278E-10	6,805	39.365	TRUE
ebi-PL179-GCST90277306	Phosphatidylcholine (18:0_22:5) levels	rs183410044	7	54,771,130	C	T	0.0159	-0.3292	0.0691	1.9021E-06	6,805	22.728	TRUE
ebi-PL179-GCST90277306	Phosphatidylcholine (18:0_22:5) levels	rs9374192	6	110,673,493	C	T	0.2717	-0.1016	0.0194	1.6912E-07	6,805	27.414	TRUE
ebi-PL179-GCST90277306	Phosphatidylcholine (18:0_22:5) levels	rs8068517	17	18,192,664	A	G	0.8140	-0.1047	0.0219	1.7352E-06	6,805	22.906	TRUE
ebi-PL179-GCST90277306	Phosphatidylcholine (18:0_22:5) levels	rs1220131	2	157,711,265	A	T	0.2002	0.1082	0.0214	4.4341E-07	6,805	25.543	TRUE
ebi-PL179-GCST90277306	Phosphatidylcholine (18:0_22:5) levels	rs115247383	3	148,282,274	T	C	0.0486	-0.2027	0.0425	1.8367E-06	6,805	22.797	TRUE
ebi-PL179-GCST90277306	Phosphatidylcholine (18:0_22:5) levels	rs7968201	12	93,538,379	T	G	0.2151	0.1000	0.0210	1.9839E-06	6,805	22.648	TRUE
ebi-PL179-GCST90277306	Phosphatidylcholine (18:0_22:5) levels	rs113847854	8	69,019,004	T	C	0.0303	0.2314	0.0494	2.8501E-06	6,805	21.950	TRUE
ebi-PL179-GCST90277306	Phosphatidylcholine (18:0_22:5) levels	rs117135394	18	54,154,713	T	C	0.0074	0.4921	0.1020	1.4470E-06	6,805	23.257	TRUE
ebi-PL179-GCST90277306	Phosphatidylcholine (18:0_22:5) levels	rs4841133	8	9,326,154	G	A	0.8518	0.1207	0.0240	5.1875E-07	6,805	25.239	TRUE
ebi-PL179-GCST90277307	Phosphatidylcholine (18:0_22:6) levels	rs2619330	3	124,802,671	T	G	0.1117	0.1260	0.0271	3.2584E-06	7,004	21.692	TRUE
ebi-PL179-GCST90277307	Phosphatidylcholine (18:0_22:6) levels	rs11792256	9	874,977	G	T	0.1030	-0.1326	0.0277	1.7333E-06	7,004	22.907	TRUE
ebi-PL179-GCST90277307	Phosphatidylcholine (18:0_22:6) levels	rs174530	11	61,779,120	G	A	0.4141	-0.1671	0.0171	2.2082E-22	7,004	95.348	TRUE
ebi-PL179-GCST90277307	Phosphatidylcholine (18:0_22:6) levels	rs7974440	12	59,019,021	A	G	0.1373	-0.1163	0.0247	2.5686E-06	7,004	22.149	TRUE
ebi-PL179-GCST90277307	Phosphatidylcholine (18:0_22:6) levels	rs62345669	4	147,476,374	A	G	0.0080	-0.4722	0.1000	2.3575E-06	7,004	22.314	TRUE
ebi-PL179-GCST90277307	Phosphatidylcholine (18:0_22:6) levels	rs143578075	12	46,987,694	A	G	0.0499	-0.1775	0.0384	3.9223E-06	7,004	21.336	TRUE
ebi-PL179-GCST90277307	Phosphatidylcholine (18:0_22:6) levels	rs2067613	3	176,189,554	C	T	0.7069	-0.0896	0.0187	1.6681E-06	7,004	22.982	TRUE
ebi-PL179-GCST90277307	Phosphatidylcholine (18:0_22:6) levels	rs7542307	1	222,093,891	T	C	0.3470	0.0845	0.0177	1.9637E-06	7,004	22.667	TRUE
ebi-PL179-GCST90277307	Phosphatidylcholine (18:0_22:6) levels	rs72827432	17	18,166,314	T	G	0.1805	0.1114	0.0218	3.3844E-07	7,004	26.065	TRUE
ebi-PL179-GCST90277307	Phosphatidylcholine (18:0_22:6) levels	rs139606388	5	134,851,174	T	C	0.0291	-0.2441	0.0499	1.0354E-06	7,004	23.902	TRUE
ebi-PL179-GCST90277307	Phosphatidylcholine (18:0_22:6) levels	rs4987173	6	42,963,486	A	G	0.3934	0.1022	0.0173	3.4755E-09	7,004	34.984	TRUE
ebi-PL179-GCST90277266	Phosphatidylcholine (18:1_0:0) levels	rs146435288	5	2,069,974	C	A	0.0702	0.1533	0.0334	4.3702E-06	7,174	21.127	TRUE
ebi-PL179-GCST90277266	Phosphatidylcholine (18:1_0:0) levels	rs61852444	1	246,692,321	C	G	0.3135	-0.0928	0.0180	2.4966E-07	7,174	26.654	TRUE
ebi-PL179-GCST90277266	Phosphatidylcholine (18:1_0:0) levels	rs71379351	17	49,270,778	C	T	0.4825	-0.0807	0.0168	1.5388E-06	7,174	23.135	TRUE
ebi-PL179-GCST90277266	Phosphatidylcholine (18:1_0:0) levels	rs11727825	4	24,133,433	A	G	0.2627	0.0929	0.0193	1.4257E-06	7,174	23.283	TRUE
ebi-PL179-GCST90277266	Phosphatidylcholine (18:1_0:0) levels	rs11720139	3	46,995,266	G	C	0.3841	0.0784	0.0171	4.9745E-06	7,174	20.877	TRUE
ebi-PL179-GCST90277266	Phosphatidylcholine (18:1_0:0) levels	rs35583283	8	18,396,999	C	G	0.1926	0.1019	0.0213	1.6986E-06	7,174	22.946	TRUE
ebi-PL179-GCST90277266	Phosphatidylcholine (18:1_0:0) levels	rs142604671	17	61,552,499	T	C	0.0929	0.1474	0.0307	1.5641E-06	7,174	23.104	TRUE

Lipid GWAS ID	Lipid species	SNP	Chromosome	Position	Effect allele	Other allele	Effect-allele frequency	Beta	SE	P value	Sample size	F statistic	F > 10
ebi-PL179-GCST90277266	Phosphatidylcholine (18:1_0:0) levels	rs6455722	6	161,340,057	G	T	0.3349	-0.0811	0.0176	4.2552E-06	7,174	21.178	TRUE
ebi-PL179-GCST90277266	Phosphatidylcholine (18:1_0:0) levels	rs7935110	11	41,087,025	G	C	0.6325	0.0823	0.0172	1.6990E-06	7,174	22.944	TRUE
ebi-PL179-GCST90277266	Phosphatidylcholine (18:1_0:0) levels	rs10833360	11	20,577,887	T	C	0.6702	-0.0836	0.0177	2.3757E-06	7,174	22.299	TRUE
ebi-PL179-GCST90277308	Phosphatidylcholine (18:1_18:1) levels	rs73241068	4	38,036,537	T	C	0.0338	-0.2183	0.0457	1.8505E-06	7,174	22.782	TRUE
ebi-PL179-GCST90277308	Phosphatidylcholine (18:1_18:1) levels	rs1077834	15	58,431,280	C	T	0.2663	0.0925	0.0192	1.5609E-06	7,174	23.109	TRUE
ebi-PL179-GCST90277308	Phosphatidylcholine (18:1_18:1) levels	rs144512980	6	18,685,527	C	T	0.0152	0.3700	0.0689	8.1687E-08	7,174	28.823	TRUE
ebi-PL179-GCST90277308	Phosphatidylcholine (18:1_18:1) levels	rs56057593	2	204,579,749	T	C	0.0315	-0.2273	0.0494	4.2981E-06	7,174	21.159	TRUE
ebi-PL179-GCST90277308	Phosphatidylcholine (18:1_18:1) levels	rs41441450	5	86,717,236	T	C	0.0098	-0.3938	0.0853	3.9249E-06	7,174	21.333	TRUE
ebi-PL179-GCST90277308	Phosphatidylcholine (18:1_18:1) levels	rs136061	22	46,945,349	G	C	0.2626	0.0890	0.0189	2.4136E-06	7,174	22.269	TRUE
ebi-PL179-GCST90277308	Phosphatidylcholine (18:1_18:1) levels	rs10779836	1	230,167,404	T	C	0.7769	0.0920	0.0200	4.1171E-06	7,174	21.242	TRUE
ebi-PL179-GCST90277308	Phosphatidylcholine (18:1_18:1) levels	rs10145681	14	68,688,212	G	A	0.9764	-0.2675	0.0566	2.3193E-06	7,174	22.345	TRUE
ebi-PL179-GCST90277308	Phosphatidylcholine (18:1_18:1) levels	rs41391244	1	111,314,654	C	T	0.2198	-0.0929	0.0201	3.8047E-06	7,174	21.393	TRUE
ebi-PL179-GCST90277309	Phosphatidylcholine (18:1_18:2) levels	rs147761802	7	5,810,475	C	G	0.0496	-0.1809	0.0392	4.0430E-06	7,174	21.278	TRUE
ebi-PL179-GCST90277309	Phosphatidylcholine (18:1_18:2) levels	rs1601935	15	58,379,566	T	G	0.6112	-0.1057	0.0170	5.3117E-10	7,174	38.669	TRUE
ebi-PL179-GCST90277309	Phosphatidylcholine (18:1_18:2) levels	rs6023985	20	55,260,213	C	T	0.0143	-0.3325	0.0721	4.0970E-06	7,174	21.250	TRUE
ebi-PL179-GCST90277309	Phosphatidylcholine (18:1_18:2) levels	rs142689129	6	18,587,903	T	C	0.0054	0.5797	0.1164	6.4247E-07	7,174	24.824	TRUE
ebi-PL179-GCST90277309	Phosphatidylcholine (18:1_18:2) levels	rs613808	11	116,840,252	G	A	0.6186	-0.0911	0.0173	1.4282E-07	7,174	27.738	TRUE
ebi-PL179-GCST90277309	Phosphatidylcholine (18:1_18:2) levels	rs564216	11	126,095,437	C	T	0.0550	-0.1679	0.0366	4.5135E-06	7,174	21.064	TRUE
ebi-PL179-GCST90277309	Phosphatidylcholine (18:1_18:2) levels	rs118108980	11	62,211,023	G	A	0.1964	-0.0996	0.0209	1.9268E-06	7,174	22.702	TRUE
ebi-PL179-GCST90277309	Phosphatidylcholine (18:1_18:2) levels	rs1077835	15	58,431,227	G	A	0.2663	0.1247	0.0191	7.9380E-11	7,174	42.398	TRUE
ebi-PL179-GCST90277309	Phosphatidylcholine (18:1_18:2) levels	rs9789400	2	57,690,087	T	C	0.2653	-0.0887	0.0189	2.7411E-06	7,174	22.027	TRUE
ebi-PL179-GCST90277309	Phosphatidylcholine (18:1_18:2) levels	rs13128807	4	169,311,729	A	G	0.1956	-0.1007	0.0211	1.8126E-06	7,174	22.821	TRUE
ebi-PL179-GCST90277309	Phosphatidylcholine (18:1_18:2) levels	rs7036554	9	14,382,621	C	T	0.3503	0.0906	0.0175	2.4304E-07	7,174	26.704	TRUE
ebi-PL179-GCST90277309	Phosphatidylcholine (18:1_18:2) levels	rs948727	18	13,715,335	G	A	0.3250	-0.0819	0.0175	2.7781E-06	7,174	22.000	TRUE
ebi-PL179-GCST90277309	Phosphatidylcholine (18:1_18:2) levels	rs2236605	21	37,563,977	C	G	0.4322	0.0839	0.0167	5.1415E-07	7,174	25.253	TRUE
ebi-PL179-GCST90277310	Phosphatidylcholine (18:1_18:3) levels	rs114797690	1	98,597,981	T	C	0.0234	0.2848	0.0622	4.8390E-06	6,339	20.932	TRUE
ebi-PL179-GCST90277310	Phosphatidylcholine (18:1_18:3) levels	rs140885981	16	75,302,415	A	G	0.0077	-0.4769	0.1032	3.8915E-06	6,339	21.357	TRUE
ebi-PL179-GCST90277310	Phosphatidylcholine (18:1_18:3) levels	rs9480321	6	156,378,387	T	A	0.0497	-0.2041	0.0409	6.1552E-07	6,339	24.912	TRUE
ebi-PL179-GCST90277310	Phosphatidylcholine (18:1_18:3) levels	rs1116931	3	100,870,486	A	C	0.2722	-0.0925	0.0197	2.7793E-06	6,339	21.998	TRUE
ebi-PL179-GCST90277310	Phosphatidylcholine (18:1_18:3) levels	rs10769099	11	5,586,584	A	G	0.3368	0.0883	0.0188	2.7331E-06	6,339	22.032	TRUE
ebi-PL179-GCST90277310	Phosphatidylcholine (18:1_18:3) levels	rs144128487	6	97,082,368	G	C	0.0120	-0.3986	0.0821	1.2317E-06	6,339	23.573	TRUE
ebi-PL179-GCST90277311	Phosphatidylcholine (18:1_20:2) levels	rs114139553	1	103,864,588	A	G	0.0138	-0.3735	0.0815	4.6317E-06	5,852	21.022	TRUE
ebi-PL179-GCST90277311	Phosphatidylcholine (18:1_20:2) levels	rs56208677	16	56,976,320	T	C	0.0777	0.1818	0.0353	2.6311E-07	5,852	26.563	TRUE
ebi-PL179-GCST90277311	Phosphatidylcholine (18:1_20:2) levels	rs74089903	12	54,160,565	A	G	0.0088	-0.5391	0.1027	1.5723E-07	5,852	27.563	TRUE
ebi-PL179-GCST90277311	Phosphatidylcholine (18:1_20:2) levels	rs35195855	11	36,094,558	T	C	0.1484	0.1299	0.0278	3.0980E-06	5,852	21.795	TRUE
ebi-PL179-GCST90277311	Phosphatidylcholine (18:1_20:2) levels	rs265114	1	217,457,286	T	C	0.0737	0.1781	0.0365	1.0646E-06	5,852	23.855	TRUE
ebi-PL179-GCST90277311	Phosphatidylcholine (18:1_20:2) levels	rs61893829	11	63,036,470	T	C	0.0811	-0.1571	0.0340	3.9632E-06	5,852	21.315	TRUE
ebi-PL179-GCST90277311	Phosphatidylcholine (18:1_20:2) levels	rs6984305	8	9,320,758	T	A	0.8375	0.1216	0.0251	1.2987E-06	5,852	23.471	TRUE
ebi-PL179-GCST90277311	Phosphatidylcholine (18:1_20:2) levels	rs80201535	7	10,826,150	A	G	0.0308	-0.2465	0.0535	4.1414E-06	5,852	21.237	TRUE
ebi-PL179-GCST90277311	Phosphatidylcholine (18:1_20:2) levels	rs10846753	12	124,836,915	G	T	0.2345	0.1060	0.0215	8.2113E-07	5,852	24.359	TRUE
ebi-PL179-GCST90277311	Phosphatidylcholine (18:1_20:2) levels	rs117534325	15	70,406,039	G	A	0.0049	0.6387	0.1355	2.4730E-06	5,852	22.231	TRUE
ebi-PL179-GCST90277311	Phosphatidylcholine (18:1_20:2) levels	rs73292027	20	40,441,104	G	T	0.1119	0.1513	0.0303	6.3122E-07	5,852	24.867	TRUE
ebi-PL179-GCST90277311	Phosphatidylcholine (18:1_20:2) levels	rs118150981	18	68,219,549	A	G	0.0193	-0.3060	0.0667	4.5440E-06	5,852	21.059	TRUE
ebi-PL179-GCST90277311	Phosphatidylcholine (18:1_20:2) levels	rs7041283	9	78,442,148	C	T	0.2451	-0.1161	0.0214	5.7944E-08	5,852	29.505	TRUE
ebi-PL179-GCST90277311	Phosphatidylcholine (18:1_20:2) levels	rs174580	11	61,839,170	G	A	0.4097	0.4314	0.0179	1.4906E-122	5,852	580.895	TRUE
ebi-PL179-GCST90277311	Phosphatidylcholine (18:1_20:2) levels	rs656095	11	75,734,293	G	A	0.2269	-0.1304	0.0222	4.2425E-09	5,852	34.612	TRUE
ebi-PL179-GCST90277312	Phosphatidylcholine (18:1_20:3) levels	rs7897746	10	5,578,436	T	C	0.9030	0.1329	0.0286	3.4415E-06	7,171	21.586	TRUE
ebi-PL179-GCST90277312	Phosphatidylcholine (18:1_20:3) levels	rs548874375	11	118,970,584	C	A	0.0273	0.2630	0.0517	3.7909E-07	7,171	25.844	TRUE
ebi-PL179-GCST90277312	Phosphatidylcholine (18:1_20:3) levels	rs10504795	8	84,222,419	G	C	0.2576	-0.0950	0.0191	6.3201E-07	7,171	24.854	TRUE
ebi-PL179-GCST90277312	Phosphatidylcholine (18:1_20:3) levels	rs10423742	19	19,117,327	A	G	0.7526	-0.0935	0.0195	1.7146E-06	7,171	22.928	TRUE
ebi-PL179-GCST90277312	Phosphatidylcholine (18:1_20:3) levels	rs79047909	2	10,043,013	G	C	0.0062	-0.4975	0.1067	3.2046E-06	7,171	21.723	TRUE
ebi-PL179-GCST90277312	Phosphatidylcholine (18:1_20:3) levels	rs6985657	8	59,010,827	G	A	0.0901	-0.1374	0.0293	2.7672E-06	7,171	22.005	TRUE
ebi-PL179-GCST90277312	Phosphatidylcholine (18:1_20:3) levels	rs822798	3	22,971,838	G	A	0.4161	0.0848	0.0168	4.9299E-07	7,171	25.337	TRUE
ebi-PL179-GCST90277312	Phosphatidylcholine (18:1_20:3) levels	rs182828998	4	115,193,660	C	A	0.0067	-0.4923	0.1045	2.5183E-06	7,171	22.187	TRUE
ebi-PL179-GCST90277312	Phosphatidylcholine (18:1_20:3) levels	rs641738	19	54,173,068	C	T	0.5928	-0.0922	0.0171	6.9706E-08	7,171	29.131	TRUE
ebi-PL179-GCST90277312	Phosphatidylcholine (18:1_20:3) levels	rs10889335	1	62,494,430	G	A	0.2847	-0.0979	0.0184	1.0563E-07	7,171	28.323	TRUE
ebi-PL179-GCST90277312	Phosphatidylcholine (18:1_20:3) levels	rs17092227	14	95,434,157	C	T	0.0442	0.1937	0.0411	2.5268E-06	7,171	22.179	TRUE
ebi-PL179-GCST90277312	Phosphatidylcholine (18:1_20:3) levels	rs61896141	11	61,788,567	C	A	0.1132	0.2236	0.0264	3.0352E-17	7,171	71.673	TRUE

Lipid GWAS ID	Lipid species	SNP	Chromosome	Position	Effect allele	Other allele	Effect-allele frequency	Beta	SE	P value	Sample size	F statistic	F > 10
ebi-PL179-GCST90277313	Phosphatidylcholine (18:1_20:4) levels	rs117052092	11	60,510,502	T	C	0.0281	-0.2339	0.0499	2.8814E-06	7,173	21.928	TRUE
ebi-PL179-GCST90277313	Phosphatidylcholine (18:1_20:4) levels	rs531117	11	75,745,089	T	C	0.2324	-0.1139	0.0198	9.9554E-09	7,173	32.933	TRUE
ebi-PL179-GCST90277313	Phosphatidylcholine (18:1_20:4) levels	rs79113397	2	101,570,369	C	T	0.2286	0.0995	0.0201	7.5898E-07	7,173	24.500	TRUE
ebi-PL179-GCST90277313	Phosphatidylcholine (18:1_20:4) levels	rs75976069	14	79,220,260	C	T	0.0262	-0.2607	0.0538	1.3056E-06	7,173	23.454	TRUE
ebi-PL179-GCST90277313	Phosphatidylcholine (18:1_20:4) levels	rs6850591	4	140,028,398	C	T	0.5413	-0.0807	0.0168	1.6400E-06	7,173	23.013	TRUE
ebi-PL179-GCST90277313	Phosphatidylcholine (18:1_20:4) levels	rs1555289	1	230,156,752	C	T	0.7683	0.0948	0.0196	1.3105E-06	7,173	23.446	TRUE
ebi-PL179-GCST90277313	Phosphatidylcholine (18:1_20:4) levels	rs61978272	14	94,523,321	T	C	0.2824	-0.0981	0.0186	1.4142E-07	7,173	27.757	TRUE
ebi-PL179-GCST90277313	Phosphatidylcholine (18:1_20:4) levels	rs8107974	19	19,277,691	T	A	0.0625	-0.1908	0.0344	2.9963E-08	7,173	30.780	TRUE
ebi-PL179-GCST90277313	Phosphatidylcholine (18:1_20:4) levels	rs173539	16	56,954,132	T	C	0.2827	0.0849	0.0184	4.2647E-06	7,173	21.173	TRUE
ebi-PL179-GCST90277267	Phosphatidylcholine (18:2_0:0) levels	rs149768482	2	204,687,512	T	C	0.0671	0.1983	0.0350	1.4592E-08	7,174	32.179	TRUE
ebi-PL179-GCST90277267	Phosphatidylcholine (18:2_0:0) levels	rs115972339	4	77,085,694	G	A	0.0350	-0.2254	0.0461	1.0434E-06	7,174	23.886	TRUE
ebi-PL179-GCST90277267	Phosphatidylcholine (18:2_0:0) levels	rs414383	17	31,622,748	T	A	0.6715	-0.0812	0.0177	4.3712E-06	7,174	21.125	TRUE
ebi-PL179-GCST90277267	Phosphatidylcholine (18:2_0:0) levels	rs139683825	11	30,336,511	G	A	0.0384	-0.2089	0.0444	2.5214E-06	7,174	22.184	TRUE
ebi-PL179-GCST90277267	Phosphatidylcholine (18:2_0:0) levels	rs59319733	1	246,693,388	A	G	0.3394	-0.0854	0.0176	1.2008E-06	7,174	23.615	TRUE
ebi-PL179-GCST90277267	Phosphatidylcholine (18:2_0:0) levels	rs8069148	17	68,491,696	C	A	0.2522	0.0894	0.0192	3.1366E-06	7,174	21.764	TRUE
ebi-PL179-GCST90277267	Phosphatidylcholine (18:2_0:0) levels	rs2610042	8	56,509,388	G	A	0.9767	0.2741	0.0564	1.1859E-06	7,174	23.639	TRUE
ebi-PL179-GCST90277267	Phosphatidylcholine (18:2_0:0) levels	rs150917153	4	182,275,394	T	C	0.0551	0.1761	0.0365	1.4544E-06	7,174	23.245	TRUE
ebi-PL179-GCST90277267	Phosphatidylcholine (18:2_0:0) levels	rs111336143	18	57,256,007	A	T	0.0272	0.2603	0.0545	1.8519E-06	7,174	22.779	TRUE
ebi-PL179-GCST90277314	Phosphatidylcholine (18:2_18:2) levels	rs1168127	1	62,670,407	A	C	0.7373	0.0917	0.0189	1.1703E-06	7,174	23.665	TRUE
ebi-PL179-GCST90277314	Phosphatidylcholine (18:2_18:2) levels	rs147002062	13	79,574,011	T	C	0.0025	-0.8393	0.1833	4.7698E-06	7,174	20.959	TRUE
ebi-PL179-GCST90277314	Phosphatidylcholine (18:2_18:2) levels	rs35958132	7	120,336,033	G	A	0.0695	0.1580	0.0344	4.4679E-06	7,174	21.084	TRUE
ebi-PL179-GCST90277314	Phosphatidylcholine (18:2_18:2) levels	rs62238391	3	10,450,834	T	C	0.3201	0.0864	0.0179	1.3837E-06	7,174	23.341	TRUE
ebi-PL179-GCST90277314	Phosphatidylcholine (18:2_18:2) levels	rs13078306	3	80,053,426	C	A	0.3281	-0.0819	0.0176	3.5056E-06	7,174	21.550	TRUE
ebi-PL179-GCST90277314	Phosphatidylcholine (18:2_18:2) levels	rs148498852	2	204,695,970	G	A	0.0772	0.1568	0.0328	1.7260E-06	7,174	22.916	TRUE
ebi-PL179-GCST90277314	Phosphatidylcholine (18:2_18:2) levels	rs59256568	2	227,805,415	G	A	0.0196	-0.2969	0.0628	2.3431E-06	7,174	22.326	TRUE
ebi-PL179-GCST90277314	Phosphatidylcholine (18:2_18:2) levels	rs117675848	20	46,799,183	A	G	0.0176	0.3209	0.0654	9.5375E-07	7,174	24.060	TRUE
ebi-PL179-GCST90277314	Phosphatidylcholine (18:2_18:2) levels	rs139598970	4	185,838,932	T	G	0.0100	0.4219	0.0896	2.5441E-06	7,174	22.167	TRUE
ebi-PL179-GCST90277314	Phosphatidylcholine (18:2_18:2) levels	rs7717591	5	52,415,905	G	A	0.6788	-0.0960	0.0179	8.4065E-08	7,174	28.769	TRUE
ebi-PL179-GCST90277314	Phosphatidylcholine (18:2_18:2) levels	rs7798734	7	91,299,442	T	G	0.3388	-0.0818	0.0177	3.7048E-06	7,174	21.444	TRUE
ebi-PL179-GCST90277314	Phosphatidylcholine (18:2_18:2) levels	rs56158036	3	95,414,411	A	G	0.0175	0.2957	0.0638	3.5800E-06	7,174	21.510	TRUE
ebi-PL179-GCST90277314	Phosphatidylcholine (18:2_18:2) levels	rs4271965	4	159,948,553	G	A	0.5672	0.0796	0.0168	2.1614E-06	7,174	22.481	TRUE
ebi-PL179-GCST90277315	Phosphatidylcholine (18:2_20:1) levels	rs174599	11	61,854,084	C	G	0.4192	0.3235	0.0187	3.9220E-65	5,521	298.284	TRUE
ebi-PL179-GCST90277315	Phosphatidylcholine (18:2_20:1) levels	rs56128146	3	87,312,021	T	C	0.4226	0.0894	0.0192	3.2474E-06	5,521	21.705	TRUE
ebi-PL179-GCST90277315	Phosphatidylcholine (18:2_20:1) levels	rs9402974	6	138,435,656	T	A	0.1538	-0.1310	0.0278	2.5504E-06	5,521	22.173	TRUE
ebi-PL179-GCST90277315	Phosphatidylcholine (18:2_20:1) levels	rs78098253	4	74,104,898	A	C	0.0303	0.2660	0.0565	2.5300E-06	5,521	22.189	TRUE
ebi-PL179-GCST90277315	Phosphatidylcholine (18:2_20:1) levels	rs182173873	11	61,678,244	C	A	0.0487	-0.2422	0.0453	9.1493E-08	5,521	28.620	TRUE
ebi-PL179-GCST90277315	Phosphatidylcholine (18:2_20:1) levels	rs200921913	8	128,749,313	T	C	0.3742	-0.0967	0.0198	1.1196E-06	5,521	23.766	TRUE
ebi-PL179-GCST90277315	Phosphatidylcholine (18:2_20:1) levels	rs17484	3	116,723,503	C	T	0.6153	0.0912	0.0197	3.7158E-06	5,521	21.448	TRUE
ebi-PL179-GCST90277315	Phosphatidylcholine (18:2_20:1) levels	rs4147976	17	68,929,782	T	C	0.5501	-0.0937	0.0193	1.2761E-06	5,521	23.509	TRUE
ebi-PL179-GCST90277315	Phosphatidylcholine (18:2_20:1) levels	rs2302263	11	64,254,349	T	C	0.1297	0.1511	0.0279	6.0990E-08	5,521	29.410	TRUE
ebi-PL179-GCST90277315	Phosphatidylcholine (18:2_20:1) levels	rs62315647	4	85,577,702	C	T	0.0178	0.3603	0.0780	3.9582E-06	5,521	21.326	TRUE
ebi-PL179-GCST90277316	Phosphatidylcholine (18:2_20:3) levels	rs2096994	18	52,821,119	A	C	0.2324	-0.1052	0.0208	4.5917E-07	6,615	25.477	TRUE
ebi-PL179-GCST90277316	Phosphatidylcholine (18:2_20:3) levels	rs138827081	5	99,176,070	A	G	0.0049	-0.5828	0.1224	1.9679E-06	6,615	22.666	TRUE
ebi-PL179-GCST90277316	Phosphatidylcholine (18:2_20:3) levels	rs11208004	1	62,679,768	A	G	0.2618	-0.0924	0.0198	3.1074E-06	6,615	21.782	TRUE
ebi-PL179-GCST90277316	Phosphatidylcholine (18:2_20:3) levels	rs10896384	11	68,952,542	A	G	0.3822	0.0879	0.0179	9.2329E-07	6,615	24.123	TRUE
ebi-PL179-GCST90277316	Phosphatidylcholine (18:2_20:3) levels	rs75719904	12	26,133,418	G	A	0.0434	0.2092	0.0442	2.2114E-06	6,615	22.440	TRUE
ebi-PL179-GCST90277316	Phosphatidylcholine (18:2_20:3) levels	rs2347672	4	43,914,879	C	A	0.6743	-0.0934	0.0185	4.4640E-07	6,615	25.529	TRUE
ebi-PL179-GCST90277316	Phosphatidylcholine (18:2_20:3) levels	rs76833802	7	155,488,156	T	C	0.0837	-0.1541	0.0328	2.5818E-06	6,615	22.141	TRUE
ebi-PL179-GCST90277317	Phosphatidylcholine (18:2_20:4) levels	rs34689885	3	138,128,558	T	A	0.2793	0.0956	0.0188	3.7591E-07	7,049	25.861	TRUE
ebi-PL179-GCST90277317	Phosphatidylcholine (18:2_20:4) levels	rs248968	19	19,760,295	C	T	0.5235	0.0805	0.0170	2.3843E-06	7,049	22.295	TRUE
ebi-PL179-GCST90277317	Phosphatidylcholine (18:2_20:4) levels	rs76780983	11	43,360,418	G	T	0.0305	0.2349	0.0495	2.1120E-06	7,049	22.526	TRUE
ebi-PL179-GCST90277317	Phosphatidylcholine (18:2_20:4) levels	rs673335	11	75,739,531	C	T	0.2455	-0.0975	0.0197	7.4304E-07	7,049	24.545	TRUE
ebi-PL179-GCST90277317	Phosphatidylcholine (18:2_20:4) levels	rs71425785	13	40,081,407	G	A	0.1199	-0.1339	0.0263	3.6581E-07	7,049	25.915	TRUE
ebi-PL179-GCST90277317	Phosphatidylcholine (18:2_20:4) levels	rs72830074	2	68,091,692	T	G	0.0513	-0.1811	0.0390	3.5292E-06	7,049	21.537	TRUE
ebi-PL179-GCST90277317	Phosphatidylcholine (18:2_20:4) levels	rs75528309	16	23,549,438	T	C	0.0475	0.2032	0.0426	1.8208E-06	7,049	22.811	TRUE
ebi-PL179-GCST90277317	Phosphatidylcholine (18:2_20:4) levels	rs836912	19	20,102,422	T	C	0.3427	-0.0837	0.0179	3.1238E-06	7,049	21.772	TRUE
ebi-PL179-GCST90277317	Phosphatidylcholine (18:2_20:4) levels	rs77985607	4	7,394,662	A	G	0.0563	-0.1734	0.0377	4.1815E-06	7,049	21.211	TRUE
ebi-PL179-GCST90277317	Phosphatidylcholine (18:2_20:4) levels	rs8736	19	54,173,495	C	T	0.5950	-0.0845	0.0172	9.9862E-07	7,049	23.973	TRUE

Lipid GWAS ID	Lipid species	SNP	Chromosome	Position	Effect allele	Other allele	Effect-allele frequency	Beta	SE	P value	Sample size	F statistic	F > 10
ebi-PL179-GCST90277317	Phosphatidylcholine (18:2_20:4) levels	rs117352906	7	18,606,087	T	C	0.0088	0.4319	0.0930	3.4715E-06	7,049	21.570	TRUE
ebi-PL179-GCST90277317	Phosphatidylcholine (18:2_20:4) levels	rs62482552	7	100,924,735	A	G	0.6081	-0.0819	0.0175	2.8138E-06	7,049	21.972	TRUE
ebi-PL179-GCST90277317	Phosphatidylcholine (18:2_20:4) levels	rs79450037	16	81,367,012	A	G	0.0391	-0.2207	0.0446	7.7211E-07	7,049	24.468	TRUE
ebi-PL179-GCST90277317	Phosphatidylcholine (18:2_20:4) levels	rs1033155	15	89,110,533	G	A	0.3983	-0.0968	0.0174	2.5017E-08	7,049	31.128	TRUE
ebi-PL179-GCST90277317	Phosphatidylcholine (18:2_20:4) levels	rs8131052	21	18,184,728	T	C	0.0936	-0.1400	0.0289	1.2506E-06	7,049	23.539	TRUE
ebi-PL179-GCST90277317	Phosphatidylcholine (18:2_20:4) levels	rs2973222	4	37,584,805	T	C	0.5056	0.0793	0.0171	3.3899E-06	7,049	21.614	TRUE
ebi-PL179-GCST90277317	Phosphatidylcholine (18:2_20:4) levels	rs139923737	7	153,908,913	T	C	0.0232	-0.3038	0.0574	1.2401E-07	7,049	28.014	TRUE
ebi-PL179-GCST90277268	Phosphatidylcholine (20:4_0:0) levels	rs62511275	8	116,151,644	G	C	0.0420	0.2204	0.0464	2.0815E-06	6,235	22.563	TRUE
ebi-PL179-GCST90277268	Phosphatidylcholine (20:4_0:0) levels	rs600626	11	75,744,264	G	A	0.2323	-0.1208	0.0212	1.2301E-08	6,235	32.523	TRUE
ebi-PL179-GCST90277268	Phosphatidylcholine (20:4_0:0) levels	rs9967725	2	56,382,464	T	A	0.3499	-0.1008	0.0187	6.7924E-08	6,235	29.187	TRUE
ebi-PL179-GCST90277268	Phosphatidylcholine (20:4_0:0) levels	rs7179330	15	23,837,617	T	A	0.9803	0.3129	0.0643	1.1489E-06	6,235	23.710	TRUE
ebi-PL179-GCST90277268	Phosphatidylcholine (20:4_0:0) levels	rs142999489	7	95,974,040	G	C	0.0562	-0.1940	0.0398	1.0913E-06	6,235	23.804	TRUE
ebi-PL179-GCST90277268	Phosphatidylcholine (20:4_0:0) levels	rs77620291	4	150,065,952	A	G	0.0081	0.4898	0.1023	1.7185E-06	6,235	22.930	TRUE
ebi-PL179-GCST90277268	Phosphatidylcholine (20:4_0:0) levels	rs174545	11	61,801,834	G	C	0.4076	-0.5340	0.0170	1.0000E-200	6,235	985.968	TRUE
ebi-PL179-GCST90277268	Phosphatidylcholine (20:4_0:0) levels	rs10733608	9	114,386,150	T	G	0.4849	0.0828	0.0178	3.3400E-06	6,235	21.654	TRUE
ebi-PL179-GCST90277268	Phosphatidylcholine (20:4_0:0) levels	rs58451960	11	62,059,600	T	C	0.1372	-0.1696	0.0263	1.2188E-10	6,235	41.573	TRUE
ebi-PL179-GCST90277268	Phosphatidylcholine (20:4_0:0) levels	rs12303	20	64,047,793	A	G	0.1964	-0.1040	0.0227	4.5650E-06	6,235	21.047	TRUE
ebi-PL179-GCST90277268	Phosphatidylcholine (20:4_0:0) levels	rs139516883	22	23,818,551	C	T	0.0163	-0.3532	0.0714	7.6096E-07	6,235	24.503	TRUE
ebi-PL179-GCST90277318	Phosphatidylcholine (O-16:0_16:0) levels	rs76494046	7	82,989,938	C	A	0.0480	-0.1886	0.0413	4.9690E-06	6,605	20.883	TRUE
ebi-PL179-GCST90277318	Phosphatidylcholine (O-16:0_16:0) levels	rs6675844	1	95,674,018	A	T	0.0813	-0.1488	0.0320	3.4991E-06	6,605	21.557	TRUE
ebi-PL179-GCST90277318	Phosphatidylcholine (O-16:0_16:0) levels	rs75248478	17	82,135,397	A	G	0.0356	0.2383	0.0482	7.8767E-07	6,605	24.436	TRUE
ebi-PL179-GCST90277318	Phosphatidylcholine (O-16:0_16:0) levels	rs34115485	18	8,859,056	G	A	0.3382	0.0860	0.0186	3.7458E-06	6,605	21.423	TRUE
ebi-PL179-GCST90277318	Phosphatidylcholine (O-16:0_16:0) levels	rs148625143	2	227,808,434	G	C	0.0430	-0.2060	0.0444	3.5386E-06	6,605	21.534	TRUE
ebi-PL179-GCST90277318	Phosphatidylcholine (O-16:0_16:0) levels	rs115294982	4	144,919,778	C	A	0.0373	-0.2227	0.0456	1.0804E-06	6,605	23.820	TRUE
ebi-PL179-GCST90277319	Phosphatidylcholine (O-16:0_16:1) levels	rs117421874	13	62,695,542	T	C	0.0075	-0.5213	0.1103	2.3590E-06	5,658	22.322	TRUE
ebi-PL179-GCST90277319	Phosphatidylcholine (O-16:0_16:1) levels	rs62311841	4	133,202,073	A	G	0.1236	-0.1618	0.0291	2.8840E-08	5,658	30.869	TRUE
ebi-PL179-GCST90277319	Phosphatidylcholine (O-16:0_16:1) levels	rs72973984	11	105,208,627	G	A	0.1094	-0.1446	0.0307	2.4314E-06	5,658	22.264	TRUE
ebi-PL179-GCST90277319	Phosphatidylcholine (O-16:0_16:1) levels	rs28609532	14	62,141,305	G	A	0.0732	-0.1659	0.0358	3.6075E-06	5,658	21.504	TRUE
ebi-PL179-GCST90277319	Phosphatidylcholine (O-16:0_16:1) levels	rs146085506	9	31,384,831	C	T	0.0584	0.2016	0.0415	1.1983E-06	5,658	23.628	TRUE
ebi-PL179-GCST90277319	Phosphatidylcholine (O-16:0_16:1) levels	rs77494629	2	101,572,416	A	C	0.2684	0.1065	0.0214	6.5383E-07	5,658	24.802	TRUE
ebi-PL179-GCST90277319	Phosphatidylcholine (O-16:0_16:1) levels	rs13394815	2	70,336,870	A	C	0.3891	-0.0943	0.0195	1.2895E-06	5,658	23.482	TRUE
ebi-PL179-GCST90277319	Phosphatidylcholine (O-16:0_16:1) levels	rs6453462	5	79,597,004	G	A	0.9341	0.1943	0.0390	6.6120E-07	5,658	24.780	TRUE
ebi-PL179-GCST90277320	Phosphatidylcholine (O-16:0_18:1) levels	rs876969	2	48,551,738	T	C	0.1500	-0.1113	0.0236	2.3950E-06	6,956	22.285	TRUE
ebi-PL179-GCST90277320	Phosphatidylcholine (O-16:0_18:1) levels	rs79037957	16	72,797,212	C	G	0.0345	0.2189	0.0477	4.4547E-06	6,956	21.090	TRUE
ebi-PL179-GCST90277320	Phosphatidylcholine (O-16:0_18:1) levels	rs34861396	8	31,640,951	A	G	0.2992	0.0871	0.0185	2.5203E-06	6,956	22.186	TRUE
ebi-PL179-GCST90277320	Phosphatidylcholine (O-16:0_18:1) levels	rs7845596	8	2,845,634	G	C	0.0769	0.1543	0.0321	1.5918E-06	6,956	23.070	TRUE
ebi-PL179-GCST90277320	Phosphatidylcholine (O-16:0_18:1) levels	rs77211823	11	12,450,600	C	T	0.0491	0.1893	0.0414	4.8315E-06	6,956	20.933	TRUE
ebi-PL179-GCST90277320	Phosphatidylcholine (O-16:0_18:1) levels	rs113746682	16	85,344,145	T	C	0.0230	0.2732	0.0593	4.1187E-06	6,956	21.242	TRUE
ebi-PL179-GCST90277320	Phosphatidylcholine (O-16:0_18:1) levels	rs9682490	3	4,949,091	C	T	0.4354	-0.0800	0.0173	3.8248E-06	6,956	21.384	TRUE
ebi-PL179-GCST90277320	Phosphatidylcholine (O-16:0_18:1) levels	rs606587	1	230,180,443	G	A	0.3740	-0.0824	0.0175	2.4870E-06	6,956	22.212	TRUE
ebi-PL179-GCST90277320	Phosphatidylcholine (O-16:0_18:1) levels	rs117449635	16	71,063,316	A	G	0.0152	0.3330	0.0720	3.8300E-06	6,956	21.380	TRUE
ebi-PL179-GCST90277320	Phosphatidylcholine (O-16:0_18:1) levels	rs7677028	4	137,022,381	G	C	0.0143	0.3933	0.0722	5.2902E-08	6,956	29.672	TRUE
ebi-PL179-GCST90277320	Phosphatidylcholine (O-16:0_18:1) levels	rs72923423	11	35,044,759	T	G	0.0736	-0.1516	0.0329	4.1683E-06	6,956	21.218	TRUE
ebi-PL179-GCST90277320	Phosphatidylcholine (O-16:0_18:1) levels	rs13194791	6	31,307,500	G	A	0.1451	0.1120	0.0241	3.5543E-06	6,956	21.522	TRUE
ebi-PL179-GCST90277321	Phosphatidylcholine (O-16:0_18:2) levels	rs6741227	2	207,017,121	T	C	0.3289	0.0848	0.0181	2.7756E-06	7,158	21.999	TRUE
ebi-PL179-GCST90277321	Phosphatidylcholine (O-16:0_18:2) levels	rs140661870	22	32,427,941	T	C	0.0151	-0.3208	0.0701	4.8381E-06	7,158	20.930	TRUE
ebi-PL179-GCST90277321	Phosphatidylcholine (O-16:0_18:2) levels	rs17086339	5	96,599,571	C	G	0.1302	-0.1167	0.0248	2.6721E-06	7,158	22.074	TRUE
ebi-PL179-GCST90277321	Phosphatidylcholine (O-16:0_18:2) levels	rs4579316	6	75,047,845	C	T	0.8335	-0.1076	0.0224	1.6620E-06	7,158	22.988	TRUE
ebi-PL179-GCST90277321	Phosphatidylcholine (O-16:0_18:2) levels	rs75804573	20	44,186,250	A	G	0.1498	0.1089	0.0237	4.5181E-06	7,158	21.063	TRUE
ebi-PL179-GCST90277321	Phosphatidylcholine (O-16:0_18:2) levels	rs11918522	3	112,816,210	A	G	0.1589	0.1058	0.0230	4.1037E-06	7,158	21.247	TRUE
ebi-PL179-GCST90277322	Phosphatidylcholine (O-16:0_20:3) levels	rs968742	7	29,412,521	G	A	0.5195	0.0808	0.0172	2.6809E-06	6,709	22.068	TRUE
ebi-PL179-GCST90277322	Phosphatidylcholine (O-16:0_20:3) levels	rs7412	19	44,908,822	T	C	0.0531	0.2245	0.0382	4.5227E-09	6,709	34.472	TRUE
ebi-PL179-GCST90277322	Phosphatidylcholine (O-16:0_20:3) levels	rs75111872	3	134,061,016	A	G	0.1621	-0.1089	0.0233	2.9285E-06	6,709	21.898	TRUE
ebi-PL179-GCST90277322	Phosphatidylcholine (O-16:0_20:3) levels	rs117435540	10	124,011,070	A	G	0.0070	0.5290	0.1142	3.6887E-06	6,709	21.452	TRUE
ebi-PL179-GCST90277323	Phosphatidylcholine (O-16:0_20:4) levels	rs6709442	2	123,584,690	A	G	0.4160	0.0947	0.0171	3.0042E-08	7,174	30.770	TRUE
ebi-PL179-GCST90277323	Phosphatidylcholine (O-16:0_20:4) levels	rs658580	9	113,637,327	A	C	0.7273	0.0898	0.0193	3.1774E-06	7,174	21.739	TRUE
ebi-PL179-GCST90277323	Phosphatidylcholine (O-16:0_20:4) levels	rs7554873	1	161,642,443	C	T	0.0504	0.1857	0.0391	2.1277E-06	7,174	22.511	TRUE
ebi-PL179-GCST90277323	Phosphatidylcholine (O-16:0_20:4) levels	rs3017103	11	62,406,721	G	A	0.8036	0.1156	0.0212	4.8656E-08	7,174	29.831	TRUE

Lipid GWAS ID	Lipid species	SNP	Chromosome	Position	Effect allele	Other allele	Effect-allele frequency	Beta	SE	P value	Sample size	F statistic	F > 10
ebi-PL179-GCST90277323	Phosphatidylcholine (O-16:0_20:4) levels	rs174568	11	61,826,344	T	C	0.4051	-0.3815	0.0165	1.1577E-114	7,174	537.148	TRUE
ebi-PL179-GCST90277323	Phosphatidylcholine (O-16:0_20:4) levels	rs79995028	14	41,287,117	T	G	0.0044	-0.6351	0.1323	1.6187E-06	7,174	23.039	TRUE
ebi-PL179-GCST90277323	Phosphatidylcholine (O-16:0_20:4) levels	rs76798309	7	110,642,820	A	G	0.0168	0.2976	0.0647	4.3564E-06	7,174	21.132	TRUE
ebi-PL179-GCST90277323	Phosphatidylcholine (O-16:0_20:4) levels	rs11057853	12	124,844,767	C	T	0.4535	-0.0881	0.0168	1.6885E-07	7,174	27.413	TRUE
ebi-PL179-GCST90277323	Phosphatidylcholine (O-16:0_20:4) levels	rs117215929	18	27,258,198	C	T	0.0416	-0.1999	0.0429	3.2712E-06	7,174	21.683	TRUE
ebi-PL179-GCST90277323	Phosphatidylcholine (O-16:0_20:4) levels	rs73422593	9	21,616,162	G	A	0.1118	-0.1298	0.0271	1.6822E-06	7,174	22.964	TRUE
ebi-PL179-GCST90277323	Phosphatidylcholine (O-16:0_20:4) levels	rs11173828	12	61,209,189	A	G	0.0688	-0.1596	0.0328	1.1693E-06	7,174	23.665	TRUE
ebi-PL179-GCST90277323	Phosphatidylcholine (O-16:0_20:4) levels	rs7982529	13	40,337,326	C	T	0.2144	0.0925	0.0201	4.3017E-06	7,174	21.157	TRUE
ebi-PL179-GCST90277323	Phosphatidylcholine (O-16:0_20:4) levels	rs2678932	2	180,057,085	C	T	0.4677	-0.0813	0.0167	1.0796E-06	7,174	23.820	TRUE
ebi-PL179-GCST90277323	Phosphatidylcholine (O-16:0_20:4) levels	rs6601694	8	8,390,548	A	G	0.3337	0.0830	0.0178	3.0390E-06	7,174	21.825	TRUE
ebi-PL179-GCST90277323	Phosphatidylcholine (O-16:0_20:4) levels	rs34881711	5	154,465,674	A	G	0.2290	0.1068	0.0198	6.8433E-08	7,174	29.168	TRUE
ebi-PL179-GCST90277323	Phosphatidylcholine (O-16:0_20:4) levels	rs77277881	1	19,722,845	A	G	0.0074	-0.4983	0.1064	2.8873E-06	7,174	21.921	TRUE
ebi-PL179-GCST90277323	Phosphatidylcholine (O-16:0_20:4) levels	rs145755646	17	59,341,291	G	C	0.0646	0.1843	0.0342	7.2337E-08	7,174	29.061	TRUE
ebi-PL179-GCST90277324	Phosphatidylcholine (O-16:0_22:5) levels	rs140792636	6	45,929,408	A	C	0.0042	-0.6728	0.1427	2.4697E-06	5,988	22.232	TRUE
ebi-PL179-GCST90277324	Phosphatidylcholine (O-16:0_22:5) levels	rs7103356	11	134,357,507	G	A	0.6436	0.0880	0.0192	4.8287E-06	5,988	20.943	TRUE
ebi-PL179-GCST90277324	Phosphatidylcholine (O-16:0_22:5) levels	rs10414438	19	55,193,090	G	A	0.1955	-0.1066	0.0233	4.9829E-06	5,988	20.880	TRUE
ebi-PL179-GCST90277324	Phosphatidylcholine (O-16:0_22:5) levels	rs55709923	1	16,964,634	G	C	0.1436	-0.1226	0.0263	3.1018E-06	5,988	21.796	TRUE
ebi-PL179-GCST90277324	Phosphatidylcholine (O-16:0_22:5) levels	rs4815298	20	2,539,461	G	A	0.5362	-0.0839	0.0183	4.8820E-06	5,988	20.919	TRUE
ebi-PL179-GCST90277324	Phosphatidylcholine (O-16:0_22:5) levels	rs145866244	5	76,222,760	A	T	0.0313	-0.2508	0.0522	1.5955E-06	5,988	23.075	TRUE
ebi-PL179-GCST90277324	Phosphatidylcholine (O-16:0_22:5) levels	rs56358871	15	58,387,188	C	G	0.4918	-0.0867	0.0180	1.5277E-06	5,988	23.151	TRUE
ebi-PL179-GCST90277324	Phosphatidylcholine (O-16:0_22:5) levels	rs118036933	20	18,282,575	T	G	0.0065	-0.5752	0.1158	6.9454E-07	5,988	24.681	TRUE
ebi-PL179-GCST90277324	Phosphatidylcholine (O-16:0_22:5) levels	rs184099437	22	43,089,231	G	C	0.0346	-0.2437	0.0515	2.2373E-06	5,988	22.418	TRUE
ebi-PL179-GCST90277325	Phosphatidylcholine (O-16:1_16:0) levels	rs78682716	12	95,184,329	A	G	0.0103	0.4651	0.0930	5.8172E-07	5,868	25.032	TRUE
ebi-PL179-GCST90277325	Phosphatidylcholine (O-16:1_16:0) levels	rs79425159	3	178,540,293	C	T	0.0392	-0.2176	0.0473	4.2570E-06	5,868	21.185	TRUE
ebi-PL179-GCST90277325	Phosphatidylcholine (O-16:1_16:0) levels	rs71423392	14	70,230,276	C	G	0.0529	-0.2002	0.0435	4.1755E-06	5,868	21.220	TRUE
ebi-PL179-GCST90277325	Phosphatidylcholine (O-16:1_16:0) levels	rs1023679	5	144,386,603	G	A	0.7513	0.1016	0.0215	2.2480E-06	5,868	22.413	TRUE
ebi-PL179-GCST90277325	Phosphatidylcholine (O-16:1_16:0) levels	rs11858902	15	88,324,326	C	A	0.3044	-0.0970	0.0212	4.7615E-06	5,868	20.966	TRUE
ebi-PL179-GCST90277325	Phosphatidylcholine (O-16:1_16:0) levels	rs79367190	12	69,475,924	T	C	0.0409	0.2173	0.0455	1.8515E-06	5,868	22.782	TRUE
ebi-PL179-GCST90277325	Phosphatidylcholine (O-16:1_16:0) levels	rs1077989	14	67,509,105	C	A	0.4675	-0.1828	0.0183	2.9146E-23	5,868	99.575	TRUE
ebi-PL179-GCST90277325	Phosphatidylcholine (O-16:1_16:0) levels	rs1247489	10	76,243,822	G	T	0.5486	0.0935	0.0185	4.7552E-07	5,868	25.416	TRUE
ebi-PL179-GCST90277326	Phosphatidylcholine (O-16:1_18:0) levels	rs9997698	4	141,036,586	C	T	0.0560	-0.1772	0.0378	2.7342E-06	6,751	22.026	TRUE
ebi-PL179-GCST90277326	Phosphatidylcholine (O-16:1_18:0) levels	rs77512406	11	66,971,292	T	C	0.1300	-0.1336	0.0259	2.6520E-07	6,751	26.542	TRUE
ebi-PL179-GCST90277326	Phosphatidylcholine (O-16:1_18:0) levels	rs11187130	10	80,809,816	T	C	0.0088	-0.4517	0.0945	1.7783E-06	6,751	22.858	TRUE
ebi-PL179-GCST90277326	Phosphatidylcholine (O-16:1_18:0) levels	rs1093316	10	94,349,017	C	T	0.0980	-0.1472	0.0292	4.6838E-07	6,751	25.437	TRUE
ebi-PL179-GCST90277326	Phosphatidylcholine (O-16:1_18:0) levels	rs1667515	14	85,227,560	A	G	0.7010	-0.0873	0.0187	3.2706E-06	6,751	21.685	TRUE
ebi-PL179-GCST90277326	Phosphatidylcholine (O-16:1_18:0) levels	rs12270739	11	36,410,769	A	G	0.1389	-0.1235	0.0257	1.6042E-06	6,751	23.059	TRUE
ebi-PL179-GCST90277326	Phosphatidylcholine (O-16:1_18:0) levels	rs1355105	17	53,585,624	C	T	0.5950	0.0810	0.0177	4.9497E-06	6,751	20.888	TRUE
ebi-PL179-GCST90277327	Phosphatidylcholine (O-16:1_18:1) levels	rs116438463	4	150,864,359	A	T	0.0575	-0.1794	0.0362	7.2815E-07	7,154	24.582	TRUE
ebi-PL179-GCST90277327	Phosphatidylcholine (O-16:1_18:1) levels	rs1441162	2	181,590,050	T	G	0.1200	-0.1263	0.0257	8.9542E-07	7,154	24.182	TRUE
ebi-PL179-GCST90277327	Phosphatidylcholine (O-16:1_18:1) levels	rs73162481	7	137,203,256	C	T	0.1639	0.1095	0.0228	1.6415E-06	7,154	23.012	TRUE
ebi-PL179-GCST90277327	Phosphatidylcholine (O-16:1_18:1) levels	rs158633	11	30,952,568	A	C	0.1119	0.1325	0.0266	6.5829E-07	7,154	24.777	TRUE
ebi-PL179-GCST90277327	Phosphatidylcholine (O-16:1_18:1) levels	rs62087966	18	369,878	G	A	0.0072	0.4856	0.1046	3.4739E-06	7,154	21.567	TRUE
ebi-PL179-GCST90277327	Phosphatidylcholine (O-16:1_18:1) levels	rs6672758	1	230,167,766	T	C	0.7419	0.1188	0.0189	3.7818E-10	7,154	39.330	TRUE
ebi-PL179-GCST90277327	Phosphatidylcholine (O-16:1_18:1) levels	rs117722620	11	131,747,965	A	G	0.0247	0.2736	0.0557	9.3062E-07	7,154	24.107	TRUE
ebi-PL179-GCST90277327	Phosphatidylcholine (O-16:1_18:1) levels	rs143615441	14	95,480,964	C	A	0.0101	-0.4434	0.0896	7.6912E-07	7,154	24.476	TRUE
ebi-PL179-GCST90277327	Phosphatidylcholine (O-16:1_18:1) levels	rs116943761	14	67,507,335	A	C	0.0822	-0.1598	0.0306	1.7388E-07	7,154	27.356	TRUE
ebi-PL179-GCST90277327	Phosphatidylcholine (O-16:1_18:1) levels	rs12426108	12	68,489,126	C	T	0.0828	-0.1559	0.0305	3.1767E-07	7,154	26.187	TRUE
ebi-PL179-GCST90277328	Phosphatidylcholine (O-16:1_18:2) levels	rs13403377	2	205,953,957	T	C	0.3559	0.0849	0.0176	1.3638E-06	7,096	23.370	TRUE
ebi-PL179-GCST90277328	Phosphatidylcholine (O-16:1_18:2) levels	rs61999264	5	149,246,504	A	G	0.0128	-0.3444	0.0745	3.8614E-06	7,096	21.365	TRUE
ebi-PL179-GCST90277328	Phosphatidylcholine (O-16:1_18:2) levels	rs117002628	12	46,480,289	A	G	0.0062	0.5106	0.1111	4.4054E-06	7,096	21.111	TRUE
ebi-PL179-GCST90277328	Phosphatidylcholine (O-16:1_18:2) levels	rs2670896	3	129,984,722	A	G	0.0303	-0.2337	0.0498	2.7557E-06	7,096	22.014	TRUE
ebi-PL179-GCST90277328	Phosphatidylcholine (O-16:1_18:2) levels	rs76103105	10	4,699,577	A	G	0.0357	-0.2115	0.0460	4.2348E-06	7,096	21.188	TRUE
ebi-PL179-GCST90277328	Phosphatidylcholine (O-16:1_18:2) levels	rs143533648	2	214,577,935	T	C	0.0262	0.2517	0.0536	2.7143E-06	7,096	22.042	TRUE
ebi-PL179-GCST90277328	Phosphatidylcholine (O-16:1_18:2) levels	rs180360	11	116,728,272	G	A	0.3235	0.0860	0.0180	1.6926E-06	7,096	22.954	TRUE
ebi-PL179-GCST90277328	Phosphatidylcholine (O-16:1_18:2) levels	rs2977197	8	1,742,309	C	G	0.9178	0.1445	0.0312	3.7514E-06	7,096	21.421	TRUE
ebi-PL179-GCST90277328	Phosphatidylcholine (O-16:1_18:2) levels	rs77172221	12	23,079,509	C	T	0.0898	-0.1373	0.0299	4.5258E-06	7,096	21.060	TRUE
ebi-PL179-GCST90277328	Phosphatidylcholine (O-16:1_18:2) levels	rs114517755	2	181,779,530	C	A	0.0051	0.6528	0.1290	4.3138E-07	7,096	25.593	TRUE
ebi-PL179-GCST90277328	Phosphatidylcholine (O-16:1_18:2) levels	rs4237708	11	15,423,060	A	G	0.8896	-0.1262	0.0270	3.0233E-06	7,096	21.835	TRUE

Lipid GWAS ID	Lipid species	SNP	Chromosome	Position	Effect allele	Other allele	Effect-allele frequency	Beta	SE	P value	Sample size	F statistic	F > 10
ebi-PL179-GCST90277328	Phosphatidylcholine (O-16:1_18:2) levels	rs627108	1	230,174,822	T	C	0.3958	-0.0859	0.0171	5.2044E-07	7,096	25.231	TRUE
ebi-PL179-GCST90277329	Phosphatidylcholine (O-16:1_20:3) levels	rs35556451	12	28,686,870	T	C	0.0357	-0.2264	0.0467	1.2809E-06	6,770	23.492	TRUE
ebi-PL179-GCST90277329	Phosphatidylcholine (O-16:1_20:3) levels	rs3795169	4	37,858,054	T	C	0.1439	0.1187	0.0247	1.5139E-06	6,770	23.172	TRUE
ebi-PL179-GCST90277329	Phosphatidylcholine (O-16:1_20:3) levels	rs6815866	4	95,119,717	C	G	0.8910	-0.1339	0.0274	1.0295E-06	6,770	23.914	TRUE
ebi-PL179-GCST90277329	Phosphatidylcholine (O-16:1_20:3) levels	rs56142013	14	56,431,229	T	C	0.0139	-0.3493	0.0760	4.3079E-06	6,770	21.155	TRUE
ebi-PL179-GCST90277329	Phosphatidylcholine (O-16:1_20:3) levels	rs686843	1	60,908,464	A	G	0.4797	0.0825	0.0178	3.8289E-06	6,770	21.382	TRUE
ebi-PL179-GCST90277329	Phosphatidylcholine (O-16:1_20:3) levels	rs9602749	13	85,434,789	G	T	0.1469	0.1141	0.0245	3.3020E-06	6,770	21.667	TRUE
ebi-PL179-GCST90277329	Phosphatidylcholine (O-16:1_20:3) levels	rs6817929	4	56,204,082	G	A	0.5880	0.0919	0.0176	1.8963E-07	6,770	27.188	TRUE
ebi-PL179-GCST90277329	Phosphatidylcholine (O-16:1_20:3) levels	rs28399664	19	44,821,499	G	C	0.0376	-0.2141	0.0462	3.6756E-06	6,770	21.463	TRUE
ebi-PL179-GCST90277329	Phosphatidylcholine (O-16:1_20:3) levels	rs34455876	7	130,154,739	A	G	0.3131	-0.0873	0.0188	3.4018E-06	6,770	21.608	TRUE
ebi-PL179-GCST90277329	Phosphatidylcholine (O-16:1_20:3) levels	rs80320850	12	61,219,406	A	G	0.0519	-0.1788	0.0387	3.9899E-06	6,770	21.303	TRUE
ebi-PL179-GCST90277329	Phosphatidylcholine (O-16:1_20:3) levels	rs144750125	17	15,541,920	T	C	0.0245	0.2585	0.0554	3.1732E-06	6,770	21.747	TRUE
ebi-PL179-GCST90277329	Phosphatidylcholine (O-16:1_20:3) levels	rs11883812	2	215,392,258	C	T	0.0486	-0.2199	0.0407	6.8583E-08	6,770	29.168	TRUE
ebi-PL179-GCST90277329	Phosphatidylcholine (O-16:1_20:3) levels	rs9872756	3	87,569,181	A	G	0.7488	0.0988	0.0198	6.2770E-07	6,770	24.874	TRUE
ebi-PL179-GCST90277330	Phosphatidylcholine (O-16:1_20:4) levels	rs114325590	1	107,442,367	T	C	0.0107	-0.4583	0.0909	4.7017E-07	6,025	25.436	TRUE
ebi-PL179-GCST90277330	Phosphatidylcholine (O-16:1_20:4) levels	rs138018897	3	19,181,916	G	A	0.0215	-0.2993	0.0653	4.7358E-06	6,025	20.981	TRUE
ebi-PL179-GCST90277330	Phosphatidylcholine (O-16:1_20:4) levels	rs12920974	16	56,959,113	T	G	0.3301	-0.1037	0.0195	1.1578E-07	6,025	28.155	TRUE
ebi-PL179-GCST90277330	Phosphatidylcholine (O-16:1_20:4) levels	rs73531625	19	18,613,744	T	C	0.2894	-0.0907	0.0198	4.8257E-06	6,025	20.942	TRUE
ebi-PL179-GCST90277330	Phosphatidylcholine (O-16:1_20:4) levels	rs2043085	15	58,388,755	C	T	0.5761	-0.0999	0.0183	4.7465E-08	6,025	29.892	TRUE
ebi-PL179-GCST90277330	Phosphatidylcholine (O-16:1_20:4) levels	rs56303140	2	157,798,940	A	G	0.0345	0.2333	0.0509	4.6527E-06	6,025	21.012	TRUE
ebi-PL179-GCST90277330	Phosphatidylcholine (O-16:1_20:4) levels	rs6752137	2	181,604,439	A	G	0.1200	-0.1289	0.0277	3.4579E-06	6,025	21.584	TRUE
ebi-PL179-GCST90277330	Phosphatidylcholine (O-16:1_20:4) levels	rs2015950	11	62,058,058	A	G	0.1372	-0.1343	0.0267	5.3217E-07	6,025	25.197	TRUE
ebi-PL179-GCST90277330	Phosphatidylcholine (O-16:1_20:4) levels	rs79740876	20	1,463,662	A	G	0.0483	0.1973	0.0429	4.2692E-06	6,025	21.177	TRUE
ebi-PL179-GCST90277331	Phosphatidylcholine (O-16:2_18:0) levels	rs6455474	6	167,990,307	C	G	0.0181	0.2986	0.0651	4.5357E-06	6,672	21.056	TRUE
ebi-PL179-GCST90277331	Phosphatidylcholine (O-16:2_18:0) levels	rs35807440	9	131,354,630	G	A	0.3481	0.0849	0.0184	4.0389E-06	6,672	21.287	TRUE
ebi-PL179-GCST90277331	Phosphatidylcholine (O-16:2_18:0) levels	rs1145605	5	158,277,324	G	A	0.1155	-0.1358	0.0279	1.1805E-06	6,672	23.652	TRUE
ebi-PL179-GCST90277331	Phosphatidylcholine (O-16:2_18:0) levels	rs588136	15	58,438,299	T	C	0.7584	-0.1905	0.0202	4.8862E-21	6,672	89.183	TRUE
ebi-PL179-GCST90277331	Phosphatidylcholine (O-16:2_18:0) levels	rs61703937	8	76,625,718	A	G	0.0876	-0.1433	0.0311	4.2606E-06	6,672	21.173	TRUE
ebi-PL179-GCST90277331	Phosphatidylcholine (O-16:2_18:0) levels	rs13433708	3	4,383,597	T	A	0.3293	-0.0939	0.0187	5.6100E-07	6,672	25.089	TRUE
ebi-PL179-GCST90277331	Phosphatidylcholine (O-16:2_18:0) levels	rs184729252	6	24,525,754	A	G	0.0026	0.8356	0.1761	2.1367E-06	6,672	22.507	TRUE
ebi-PL179-GCST90277331	Phosphatidylcholine (O-16:2_18:0) levels	rs3814533	9	81,637,892	T	C	0.9148	-0.1508	0.0315	1.7682E-06	6,672	22.869	TRUE
ebi-PL179-GCST90277332	Phosphatidylcholine (O-17:0_15:0) levels	rs150669352	2	238,794,542	T	C	0.0078	-0.5036	0.1095	4.3351E-06	6,140	21.147	TRUE
ebi-PL179-GCST90277332	Phosphatidylcholine (O-17:0_15:0) levels	rs145150348	1	62,121,729	A	G	0.0173	0.3367	0.0689	1.0510E-06	6,140	23.879	TRUE
ebi-PL179-GCST90277332	Phosphatidylcholine (O-17:0_15:0) levels	rs146967253	7	81,169,417	T	C	0.0261	0.2850	0.0600	2.0473E-06	6,140	22.592	TRUE
ebi-PL179-GCST90277332	Phosphatidylcholine (O-17:0_15:0) levels	rs4949186	1	30,929,301	C	T	0.6996	-0.0971	0.0196	7.4108E-07	6,140	24.554	TRUE
ebi-PL179-GCST90277332	Phosphatidylcholine (O-17:0_15:0) levels	rs74648736	3	173,729,514	T	A	0.0220	-0.3283	0.0717	4.7941E-06	6,140	20.954	TRUE
ebi-PL179-GCST90277332	Phosphatidylcholine (O-17:0_15:0) levels	rs144941484	2	158,921,003	T	C	0.0059	0.5922	0.1222	1.2830E-06	6,140	23.493	TRUE
ebi-PL179-GCST90277332	Phosphatidylcholine (O-17:0_15:0) levels	rs139939351	22	49,966,644	G	T	0.0043	0.7393	0.1595	3.6405E-06	6,140	21.483	TRUE
ebi-PL179-GCST90277332	Phosphatidylcholine (O-17:0_15:0) levels	rs72967793	11	95,940,105	T	A	0.0601	-0.1804	0.0386	2.9948E-06	6,140	21.859	TRUE
ebi-PL179-GCST90277332	Phosphatidylcholine (O-17:0_15:0) levels	rs112686420	13	42,902,737	G	A	0.0094	0.4703	0.0989	2.0199E-06	6,140	22.618	TRUE
ebi-PL179-GCST90277332	Phosphatidylcholine (O-17:0_15:0) levels	rs417916	7	41,274,867	T	C	0.4398	-0.0879	0.0188	3.0880E-06	6,140	21.800	TRUE
ebi-PL179-GCST90277333	Phosphatidylcholine (O-17:0_17:1) levels	rs12186559	5	3,950,508	T	C	0.0360	0.2270	0.0467	1.1949E-06	6,880	23.625	TRUE
ebi-PL179-GCST90277333	Phosphatidylcholine (O-17:0_17:1) levels	rs11691441	2	100,856,837	C	G	0.0473	-0.1829	0.0399	4.6736E-06	6,880	20.999	TRUE
ebi-PL179-GCST90277333	Phosphatidylcholine (O-17:0_17:1) levels	rs38820	7	118,070,482	C	G	0.9144	0.1398	0.0306	4.9563E-06	6,880	20.886	TRUE
ebi-PL179-GCST90277333	Phosphatidylcholine (O-17:0_17:1) levels	rs7223101	17	66,571,589	A	C	0.3820	0.0816	0.0178	4.3851E-06	6,880	21.124	TRUE
ebi-PL179-GCST90277333	Phosphatidylcholine (O-17:0_17:1) levels	rs17611110	4	168,219,402	C	T	0.2412	-0.0998	0.0200	5.8590E-07	6,880	25.002	TRUE
ebi-PL179-GCST90277333	Phosphatidylcholine (O-17:0_17:1) levels	rs17513056	6	158,732,893	C	T	0.2123	0.1041	0.0211	8.1139E-07	6,880	24.372	TRUE
ebi-PL179-GCST90277333	Phosphatidylcholine (O-17:0_17:1) levels	rs79648513	7	18,311,602	G	A	0.2293	0.0968	0.0206	2.6896E-06	6,880	22.063	TRUE
ebi-PL179-GCST90277333	Phosphatidylcholine (O-17:0_17:1) levels	rs7575011	2	113,675,313	G	T	0.6623	-0.0824	0.0180	4.6284E-06	6,880	21.017	TRUE
ebi-PL179-GCST90277333	Phosphatidylcholine (O-17:0_17:1) levels	rs4896718	6	123,163,417	C	T	0.7822	0.0972	0.0209	3.2757E-06	6,880	21.682	TRUE
ebi-PL179-GCST90277333	Phosphatidylcholine (O-17:0_17:1) levels	rs11068152	12	116,703,206	A	G	0.2233	-0.0943	0.0206	4.8906E-06	6,880	20.913	TRUE
ebi-PL179-GCST90277333	Phosphatidylcholine (O-17:0_17:1) levels	rs150269921	19	45,759,621	C	T	0.0072	-0.4635	0.1006	4.1475E-06	6,880	21.228	TRUE
ebi-PL179-GCST90277333	Phosphatidylcholine (O-17:0_17:1) levels	rs62072490	17	5,010,850	T	G	0.1938	-0.1079	0.0219	8.6931E-07	6,880	24.242	TRUE
ebi-PL179-GCST90277333	Phosphatidylcholine (O-17:0_17:1) levels	rs72495602	21	42,137,794	A	G	0.1253	0.1220	0.0259	2.5655E-06	6,880	22.152	TRUE
ebi-PL179-GCST90277334	Phosphatidylcholine (O-18:0_14:0) levels	rs9365258	6	161,187,390	C	G	0.8369	-0.1198	0.0239	5.7595E-07	6,462	25.040	TRUE
ebi-PL179-GCST90277334	Phosphatidylcholine (O-18:0_14:0) levels	rs79139024	14	95,941,109	C	G	0.0076	-0.4875	0.1067	4.9632E-06	6,462	20.885	TRUE
ebi-PL179-GCST90277334	Phosphatidylcholine (O-18:0_14:0) levels	rs146726414	16	78,809,113	T	G	0.0200	0.3118	0.0632	8.1622E-07	6,462	24.365	TRUE
ebi-PL179-GCST90277334	Phosphatidylcholine (O-18:0_14:0) levels	rs72637380	17	16,925,824	T	C	0.0665	-0.1688	0.0354	1.8386E-06	6,462	22.797	TRUE

Lipid GWAS ID	Lipid species	SNP	Chromosome	Position	Effect allele	Other allele	Effect-allele frequency	Beta	SE	P value	Sample size	F statistic	F > 10
ebi-PL179-GCST90277334	Phosphatidylcholine (O-18:0_14:0) levels	rs117246644	10	58,404,019	T	C	0.0100	-0.4419	0.0901	9.6013E-07	6,462	24.051	TRUE
ebi-PL179-GCST90277334	Phosphatidylcholine (O-18:0_14:0) levels	rs2115108	19	7,899,091	C	T	0.2430	0.0982	0.0205	1.7337E-06	6,462	22.910	TRUE
ebi-PL179-GCST90277334	Phosphatidylcholine (O-18:0_14:0) levels	rs77444850	3	127,976,198	C	A	0.0245	-0.2714	0.0587	3.8268E-06	6,462	21.385	TRUE
ebi-PL179-GCST90277334	Phosphatidylcholine (O-18:0_14:0) levels	rs116907632	17	81,307,290	A	G	0.0042	0.7062	0.1501	2.5810E-06	6,462	22.143	TRUE
ebi-PL179-GCST90277334	Phosphatidylcholine (O-18:0_14:0) levels	rs72835302	10	86,168,949	A	C	0.1017	0.1417	0.0304	3.0883E-06	6,462	21.798	TRUE
ebi-PL179-GCST90277334	Phosphatidylcholine (O-18:0_14:0) levels	rs8129049	21	38,363,308	G	A	0.2523	0.0962	0.0204	2.4043E-06	6,462	22.280	TRUE
ebi-PL179-GCST90277335	Phosphatidylcholine (O-18:0_16:1) levels	rs55969585	1	117,639,417	T	G	0.0459	0.2087	0.0456	4.7519E-06	5,959	20.977	TRUE
ebi-PL179-GCST90277335	Phosphatidylcholine (O-18:0_16:1) levels	rs4547920	5	124,738,074	A	G	0.1665	0.1265	0.0250	4.2589E-07	5,959	25.633	TRUE
ebi-PL179-GCST90277335	Phosphatidylcholine (O-18:0_16:1) levels	rs74644051	13	27,642,024	A	G	0.0169	-0.3440	0.0723	2.0352E-06	5,959	22.606	TRUE
ebi-PL179-GCST90277335	Phosphatidylcholine (O-18:0_16:1) levels	rs10243115	7	49,798,317	T	C	0.3922	0.0875	0.0187	3.0093E-06	5,959	21.847	TRUE
ebi-PL179-GCST90277335	Phosphatidylcholine (O-18:0_16:1) levels	rs2911458	16	88,778,601	T	C	0.3889	-0.0896	0.0186	1.5385E-06	5,959	23.139	TRUE
ebi-PL179-GCST90277335	Phosphatidylcholine (O-18:0_16:1) levels	rs149012657	2	229,375,003	C	T	0.0648	-0.1883	0.0370	3.8231E-07	5,959	25.837	TRUE
ebi-PL179-GCST90277335	Phosphatidylcholine (O-18:0_16:1) levels	rs12575589	11	96,017,012	T	C	0.2052	0.1118	0.0230	1.1693E-06	5,959	23.674	TRUE
ebi-PL179-GCST90277335	Phosphatidylcholine (O-18:0_16:1) levels	rs11009204	10	33,061,164	A	G	0.0984	0.1434	0.0313	4.5636E-06	5,959	21.053	TRUE
ebi-PL179-GCST90277336	Phosphatidylcholine (O-18:0_20:4) levels	rs6992951	8	18,172,468	A	C	0.3899	-0.0822	0.0173	2.0490E-06	6,956	22.586	TRUE
ebi-PL179-GCST90277336	Phosphatidylcholine (O-18:0_20:4) levels	rs174536	11	61,784,455	C	A	0.4085	-0.3084	0.0169	2.2814E-72	6,956	331.279	TRUE
ebi-PL179-GCST90277336	Phosphatidylcholine (O-18:0_20:4) levels	rs4782783	16	83,381,927	C	T	0.8880	0.1253	0.0270	3.6432E-06	6,956	21.479	TRUE
ebi-PL179-GCST90277336	Phosphatidylcholine (O-18:0_20:4) levels	rs143233558	7	64,330,243	A	G	0.1022	0.1494	0.0284	1.5460E-07	6,956	27.585	TRUE
ebi-PL179-GCST90277336	Phosphatidylcholine (O-18:0_20:4) levels	rs138202451	2	66,991,489	A	G	0.0062	-0.5761	0.1113	2.3243E-07	6,956	26.794	TRUE
ebi-PL179-GCST90277336	Phosphatidylcholine (O-18:0_20:4) levels	rs34382810	12	2,231,632	C	A	0.3646	0.0858	0.0176	1.1161E-06	6,956	23.756	TRUE
ebi-PL179-GCST90277336	Phosphatidylcholine (O-18:0_20:4) levels	rs148462060	2	130,169,813	G	A	0.0243	-0.2608	0.0560	3.3013E-06	6,956	21.667	TRUE
ebi-PL179-GCST90277336	Phosphatidylcholine (O-18:0_20:4) levels	rs79885747	9	79,739,961	T	C	0.0498	0.1821	0.0397	4.5468E-06	6,956	21.051	TRUE
ebi-PL179-GCST90277337	Phosphatidylcholine (O-18:1_16:0) levels	rs7450458	6	31,450,839	A	G	0.1443	-0.1142	0.0240	1.9921E-06	6,956	22.639	TRUE
ebi-PL179-GCST90277337	Phosphatidylcholine (O-18:1_16:0) levels	rs138542536	4	25,796,122	C	A	0.0133	-0.3866	0.0794	1.1358E-06	6,956	23.726	TRUE
ebi-PL179-GCST90277337	Phosphatidylcholine (O-18:1_16:0) levels	rs72683831	4	124,795,272	C	T	0.0710	0.1587	0.0335	2.2255E-06	6,956	22.425	TRUE
ebi-PL179-GCST90277337	Phosphatidylcholine (O-18:1_16:0) levels	rs2043082	15	58,382,109	A	G	0.4110	0.0966	0.0171	1.6146E-08	6,956	31.983	TRUE
ebi-PL179-GCST90277337	Phosphatidylcholine (O-18:1_16:0) levels	rs57493997	6	34,475,746	C	T	0.0814	-0.1439	0.0310	3.5657E-06	6,956	21.519	TRUE
ebi-PL179-GCST90277337	Phosphatidylcholine (O-18:1_16:0) levels	rs118138240	16	760,921	T	C	0.0334	-0.2295	0.0490	2.8881E-06	6,956	21.924	TRUE
ebi-PL179-GCST90277337	Phosphatidylcholine (O-18:1_16:0) levels	rs60331765	2	181,582,854	G	A	0.1804	-0.1027	0.0219	2.7954E-06	6,956	21.987	TRUE
ebi-PL179-GCST90277337	Phosphatidylcholine (O-18:1_16:0) levels	rs79674727	19	28,504,762	C	T	0.1111	0.1285	0.0274	2.7215E-06	6,956	22.038	TRUE
ebi-PL179-GCST90277337	Phosphatidylcholine (O-18:1_16:0) levels	rs246168	16	14,276,763	A	C	0.0673	0.1581	0.0343	4.0518E-06	6,956	21.272	TRUE
ebi-PL179-GCST90277337	Phosphatidylcholine (O-18:1_16:0) levels	rs187861964	20	61,244,195	G	C	0.0264	0.2601	0.0561	3.5439E-06	6,956	21.531	TRUE
ebi-PL179-GCST90277337	Phosphatidylcholine (O-18:1_16:0) levels	rs1503311	11	13,766,154	T	C	0.1972	0.0983	0.0212	3.4274E-06	6,956	21.594	TRUE
ebi-PL179-GCST90277337	Phosphatidylcholine (O-18:1_16:0) levels	rs7330980	13	60,824,670	A	G	0.0759	0.1485	0.0325	4.9031E-06	6,956	20.905	TRUE
ebi-PL179-GCST90277337	Phosphatidylcholine (O-18:1_16:0) levels	rs13030345	2	27,780,307	T	G	0.1863	-0.1078	0.0217	6.7348E-07	6,956	24.735	TRUE
ebi-PL179-GCST90277337	Phosphatidylcholine (O-18:1_16:0) levels	rs78194510	12	124,853,790	C	T	0.1173	0.1346	0.0264	3.3197E-07	6,956	26.102	TRUE
ebi-PL179-GCST90277337	Phosphatidylcholine (O-18:1_16:0) levels	rs7327319	13	40,354,480	A	C	0.1501	0.1171	0.0236	7.4341E-07	6,956	24.542	TRUE
ebi-PL179-GCST90277338	Phosphatidylcholine (O-18:1_18:2) levels	rs72709643	1	181,004,059	C	T	0.0362	-0.2127	0.0464	4.7378E-06	6,718	20.973	TRUE
ebi-PL179-GCST90277338	Phosphatidylcholine (O-18:1_18:2) levels	rs62331262	4	104,599,304	C	G	0.1731	0.1075	0.0230	3.1072E-06	6,718	21.784	TRUE
ebi-PL179-GCST90277338	Phosphatidylcholine (O-18:1_18:2) levels	rs2985394	11	34,887,145	G	A	0.6685	0.0867	0.0184	2.6337E-06	6,718	22.103	TRUE
ebi-PL179-GCST90277338	Phosphatidylcholine (O-18:1_18:2) levels	rs79040967	11	97,125,581	A	G	0.0444	0.2011	0.0429	2.7541E-06	6,718	22.017	TRUE
ebi-PL179-GCST90277338	Phosphatidylcholine (O-18:1_18:2) levels	rs58828781	19	2,786,104	A	T	0.0481	0.1973	0.0429	4.4231E-06	6,718	21.106	TRUE
ebi-PL179-GCST90277338	Phosphatidylcholine (O-18:1_18:2) levels	rs73133304	20	21,155,899	C	A	0.0053	0.5518	0.1206	4.8060E-06	6,718	20.946	TRUE
ebi-PL179-GCST90277338	Phosphatidylcholine (O-18:1_18:2) levels	rs2537611	7	17,693,175	T	C	0.3722	0.0875	0.0179	9.7749E-07	6,718	24.016	TRUE
ebi-PL179-GCST90277339	Phosphatidylcholine (O-18:1_20:3) levels	rs11236557	11	75,841,084	T	C	0.5820	0.0920	0.0179	2.6271E-07	6,836	26.556	TRUE
ebi-PL179-GCST90277339	Phosphatidylcholine (O-18:1_20:3) levels	rs12444786	16	82,643,141	G	A	0.8210	0.1076	0.0231	3.2858E-06	6,836	21.677	TRUE
ebi-PL179-GCST90277339	Phosphatidylcholine (O-18:1_20:3) levels	rs2170790	3	25,418,634	C	T	0.0052	0.6280	0.1298	1.3345E-06	6,836	23.415	TRUE
ebi-PL179-GCST90277339	Phosphatidylcholine (O-18:1_20:3) levels	rs62493656	7	158,353,254	T	C	0.0359	-0.2138	0.0463	3.8887E-06	6,836	21.352	TRUE
ebi-PL179-GCST90277339	Phosphatidylcholine (O-18:1_20:3) levels	rs111260004	20	35,695,325	C	G	0.0264	-0.2559	0.0558	4.5746E-06	6,836	21.040	TRUE
ebi-PL179-GCST90277339	Phosphatidylcholine (O-18:1_20:3) levels	rs12239821	1	245,438,211	A	G	0.1077	-0.1296	0.0283	4.6196E-06	6,836	21.020	TRUE
ebi-PL179-GCST90277339	Phosphatidylcholine (O-18:1_20:3) levels	rs9843297	3	22,449,600	G	A	0.1957	0.0998	0.0218	4.7472E-06	6,836	20.969	TRUE
ebi-PL179-GCST90277339	Phosphatidylcholine (O-18:1_20:3) levels	rs1540881	7	18,082,952	T	C	0.5403	0.0827	0.0179	3.7339E-06	6,836	21.431	TRUE
ebi-PL179-GCST90277340	Phosphatidylcholine (O-18:1_20:4) levels	rs4142412	6	34,451,115	A	G	0.7624	0.0937	0.0195	1.6557E-06	7,173	22.995	TRUE
ebi-PL179-GCST90277340	Phosphatidylcholine (O-18:1_20:4) levels	rs4618752	9	1,294,441	T	G	0.5059	0.0770	0.0166	3.5679E-06	7,173	21.517	TRUE
ebi-PL179-GCST90277341	Phosphatidylcholine (O-18:2_16:0) levels	rs185625951	1	49,528,668	T	G	0.0195	-0.2935	0.0623	2.5229E-06	7,158	22.183	TRUE
ebi-PL179-GCST90277341	Phosphatidylcholine (O-18:2_16:0) levels	rs188033349	4	28,252,716	A	G	0.0091	-0.4492	0.0942	1.8840E-06	7,158	22.746	TRUE
ebi-PL179-GCST90277341	Phosphatidylcholine (O-18:2_16:0) levels	rs76437425	1	245,514,505	T	G	0.1731	0.1062	0.0223	1.9573E-06	7,158	22.672	TRUE
ebi-PL179-GCST90277341	Phosphatidylcholine (O-18:2_16:0) levels	rs12123955	1	200,136,295	T	C	0.0612	-0.1615	0.0348	3.5915E-06	7,158	21.503	TRUE

Lipid GWAS ID	Lipid species	SNP	Chromosome	Position	Effect allele	Other allele	Effect-allele frequency	Beta	SE	P value	Sample size	F statistic	F > 10
ebi-PL179-GCST90277341	Phosphatidylcholine (O-18:2_16:0) levels	rs142235374	4	7,350,426	A	G	0.0177	0.3472	0.0645	7.4435E-08	7,158	29.008	TRUE
ebi-PL179-GCST90277341	Phosphatidylcholine (O-18:2_16:0) levels	rs149897686	8	31,250,608	T	C	0.0204	0.3013	0.0625	1.4619E-06	7,158	23.237	TRUE
ebi-PL179-GCST90277341	Phosphatidylcholine (O-18:2_16:0) levels	rs113016188	11	100,294,047	T	C	0.0026	-0.7956	0.1655	1.5558E-06	7,158	23.115	TRUE
ebi-PL179-GCST90277341	Phosphatidylcholine (O-18:2_16:0) levels	rs10768424	11	4,976,404	G	A	0.5725	0.0786	0.0170	3.9768E-06	7,158	21.307	TRUE
ebi-PL179-GCST90277341	Phosphatidylcholine (O-18:2_16:0) levels	rs9341454	6	74,169,888	G	A	0.6709	-0.0816	0.0178	4.3668E-06	7,158	21.126	TRUE
ebi-PL179-GCST90277341	Phosphatidylcholine (O-18:2_16:0) levels	rs7563526	2	81,754,844	T	C	0.0313	0.2432	0.0500	1.1658E-06	7,158	23.673	TRUE
ebi-PL179-GCST90277342	Phosphatidylcholine (O-18:2_18:1) levels	rs174564	11	61,820,833	G	A	0.4092	0.1466	0.0175	7.4742E-17	6,677	69.905	TRUE
ebi-PL179-GCST90277342	Phosphatidylcholine (O-18:2_18:1) levels	rs7642984	3	112,839,107	T	C	0.1574	0.1296	0.0238	5.0501E-08	6,677	29.763	TRUE
ebi-PL179-GCST90277342	Phosphatidylcholine (O-18:2_18:1) levels	rs10868635	9	87,561,451	A	G	0.2987	-0.0887	0.0188	2.5940E-06	6,677	22.132	TRUE
ebi-PL179-GCST90277342	Phosphatidylcholine (O-18:2_18:1) levels	rs117376487	11	35,071,655	A	G	0.0091	-0.4167	0.0895	3.3093E-06	6,677	21.663	TRUE
ebi-PL179-GCST90277342	Phosphatidylcholine (O-18:2_18:1) levels	rs546728326	7	53,119,305	TTA	T	0.0340	-0.2367	0.0478	7.7159E-07	6,677	24.472	TRUE
ebi-PL179-GCST90277342	Phosphatidylcholine (O-18:2_18:1) levels	rs62621679	17	66,778,240	G	C	0.0609	0.1748	0.0364	1.6146E-06	6,677	23.045	TRUE
ebi-PL179-GCST90277342	Phosphatidylcholine (O-18:2_18:1) levels	rs55975242	2	4,201,657	A	G	0.0373	-0.2164	0.0460	2.5757E-06	6,677	22.147	TRUE
ebi-PL179-GCST90277342	Phosphatidylcholine (O-18:2_18:1) levels	rs72840788	10	119,656,173	A	G	0.2294	0.0973	0.0209	3.1585E-06	6,677	21.753	TRUE
ebi-PL179-GCST90277343	Phosphatidylcholine (O-18:2_18:2) levels	rs191784724	1	43,502,581	C	A	0.0306	0.2336	0.0495	2.3794E-06	7,153	22.296	TRUE
ebi-PL179-GCST90277343	Phosphatidylcholine (O-18:2_18:2) levels	rs61448283	6	169,316,922	T	C	0.0543	-0.1746	0.0377	3.6892E-06	7,153	21.452	TRUE
ebi-PL179-GCST90277343	Phosphatidylcholine (O-18:2_18:2) levels	rs114401161	5	154,241,592	G	T	0.0417	0.1954	0.0419	3.1432E-06	7,153	21.760	TRUE
ebi-PL179-GCST90277343	Phosphatidylcholine (O-18:2_18:2) levels	rs35874447	12	124,967,043	G	A	0.2881	0.0959	0.0187	2.7971E-07	7,153	26.435	TRUE
ebi-PL179-GCST90277343	Phosphatidylcholine (O-18:2_18:2) levels	rs784655	9	107,072,174	G	A	0.5500	-0.0810	0.0169	1.5897E-06	7,153	23.073	TRUE
ebi-PL179-GCST90277343	Phosphatidylcholine (O-18:2_18:2) levels	rs28489182	14	77,828,999	A	G	0.1448	0.1124	0.0238	2.4613E-06	7,153	22.231	TRUE
ebi-PL179-GCST90277343	Phosphatidylcholine (O-18:2_18:2) levels	rs114930909	3	60,237,477	A	G	0.0119	0.3690	0.0784	2.5749E-06	7,153	22.143	TRUE
ebi-PL179-GCST90277344	Phosphatidylcholine (O-18:2_20:4) levels	rs2189991	7	82,556,403	A	G	0.5867	0.0873	0.0181	1.5323E-06	6,127	23.151	TRUE
ebi-PL179-GCST90277344	Phosphatidylcholine (O-18:2_20:4) levels	rs13401553	2	123,610,457	C	T	0.4275	0.0872	0.0184	2.1787E-06	6,127	22.472	TRUE
ebi-PL179-GCST90277344	Phosphatidylcholine (O-18:2_20:4) levels	rs16975847	16	75,555,692	A	G	0.1337	-0.1236	0.0263	2.7627E-06	6,127	22.014	TRUE
ebi-PL179-GCST90277344	Phosphatidylcholine (O-18:2_20:4) levels	rs12046045	1	47,591,044	A	G	0.0890	-0.1563	0.0321	1.1191E-06	6,127	23.758	TRUE
ebi-PL179-GCST90277344	Phosphatidylcholine (O-18:2_20:4) levels	rs6467318	7	130,802,167	G	A	0.5737	-0.0880	0.0188	2.8156E-06	6,127	21.978	TRUE
ebi-PL179-GCST90277344	Phosphatidylcholine (O-18:2_20:4) levels	rs76375697	9	32,337,256	G	T	0.0178	0.3402	0.0711	1.7674E-06	6,127	22.875	TRUE
ebi-PL179-GCST90277344	Phosphatidylcholine (O-18:2_20:4) levels	rs7118021	11	89,058,340	A	G	0.1593	-0.1170	0.0246	1.9544E-06	6,127	22.681	TRUE
ebi-PL179-GCST90277344	Phosphatidylcholine (O-18:2_20:4) levels	rs174537	11	61,785,208	T	G	0.4078	-0.1759	0.0184	1.9713E-21	6,127	91.043	TRUE
ebi-PL179-GCST90277344	Phosphatidylcholine (O-18:2_20:4) levels	rs141198865	17	17,676,043	T	C	0.0175	-0.3493	0.0757	4.0182E-06	6,127	21.293	TRUE
ebi-PL179-GCST90277344	Phosphatidylcholine (O-18:2_20:4) levels	rs113023745	13	66,979,217	A	G	0.0370	-0.2540	0.0475	9.2065E-08	6,127	28.601	TRUE
ebi-PL179-GCST90277344	Phosphatidylcholine (O-18:2_20:4) levels	rs6722606	2	191,752,537	C	A	0.0095	-0.4520	0.0960	2.5196E-06	6,127	22.192	TRUE
ebi-PL179-GCST90277344	Phosphatidylcholine (O-18:2_20:4) levels	rs17778967	15	43,258,624	A	G	0.0323	0.2501	0.0530	2.4585E-06	6,127	22.239	TRUE
ebi-PL179-GCST90277344	Phosphatidylcholine (O-18:2_20:4) levels	rs1980615	14	67,491,655	C	A	0.4551	-0.1033	0.0181	1.1150E-08	6,127	32.716	TRUE
ebi-PL179-GCST90277345	Phosphatidylethanolamine (16:0_18:2) levels	rs4269529	8	66,311,811	C	T	0.0100	-0.5044	0.0980	2.7593E-07	5,462	26.475	TRUE
ebi-PL179-GCST90277345	Phosphatidylethanolamine (16:0_18:2) levels	rs34278620	15	59,032,191	G	A	0.1250	0.2255	0.0285	2.9476E-15	5,462	62.660	TRUE
ebi-PL179-GCST90277345	Phosphatidylethanolamine (16:0_18:2) levels	rs510988	11	116,943,088	T	C	0.8287	-0.1462	0.0253	7.4007E-09	5,462	33.531	TRUE
ebi-PL179-GCST90277345	Phosphatidylethanolamine (16:0_18:2) levels	rs140904588	2	11,679,202	A	G	0.0612	0.1974	0.0422	2.8900E-06	5,462	21.931	TRUE
ebi-PL179-GCST90277345	Phosphatidylethanolamine (16:0_18:2) levels	rs7180275	15	74,379,820	G	A	0.1534	-0.1419	0.0273	2.0505E-07	5,462	27.055	TRUE
ebi-PL179-GCST90277345	Phosphatidylethanolamine (16:0_18:2) levels	rs12226389	11	62,056,158	C	T	0.1728	0.1179	0.0252	3.1086E-06	5,462	21.794	TRUE
ebi-PL179-GCST90277345	Phosphatidylethanolamine (16:0_18:2) levels	rs117099105	9	116,846,849	C	G	0.0109	0.4585	0.0977	2.7284E-06	5,462	22.043	TRUE
ebi-PL179-GCST90277345	Phosphatidylethanolamine (16:0_18:2) levels	rs61696472	7	43,464,229	G	A	0.3900	0.0922	0.0194	1.9936E-06	5,462	22.649	TRUE
ebi-PL179-GCST90277345	Phosphatidylethanolamine (16:0_18:2) levels	rs544282	15	58,729,903	A	G	0.2799	0.1061	0.0211	5.1815E-07	5,462	25.257	TRUE
ebi-PL179-GCST90277345	Phosphatidylethanolamine (16:0_18:2) levels	rs4129186	1	214,395,764	C	T	0.0957	-0.1531	0.0329	3.3738E-06	5,462	21.635	TRUE
ebi-PL179-GCST90277346	Phosphatidylethanolamine (16:0_20:4) levels	rs1193168	1	7,531,062	C	A	0.6550	0.0846	0.0185	4.6409E-06	6,666	21.014	TRUE
ebi-PL179-GCST90277346	Phosphatidylethanolamine (16:0_20:4) levels	rs115265221	3	141,158,645	G	C	0.0082	-0.4675	0.0967	1.3700E-06	6,666	23.363	TRUE
ebi-PL179-GCST90277346	Phosphatidylethanolamine (16:0_20:4) levels	rs139848079	10	59,368,742	T	C	0.0304	-0.2545	0.0531	1.6605E-06	6,666	22.992	TRUE
ebi-PL179-GCST90277346	Phosphatidylethanolamine (16:0_20:4) levels	rs75344234	8	138,164,717	C	T	0.0658	-0.1778	0.0374	1.9773E-06	6,666	22.655	TRUE
ebi-PL179-GCST90277346	Phosphatidylethanolamine (16:0_20:4) levels	rs4370152	4	121,541,914	A	G	0.4174	-0.0830	0.0177	2.7047E-06	6,666	22.051	TRUE
ebi-PL179-GCST90277346	Phosphatidylethanolamine (16:0_20:4) levels	rs1690333	15	57,616,988	C	T	0.4392	0.0996	0.0175	1.2029E-08	6,666	32.556	TRUE
ebi-PL179-GCST90277346	Phosphatidylethanolamine (16:0_20:4) levels	rs2684847	18	47,243,946	A	C	0.8528	-0.1180	0.0248	2.0182E-06	6,666	22.615	TRUE
ebi-PL179-GCST90277346	Phosphatidylethanolamine (16:0_20:4) levels	rs9943332	10	128,061,505	A	G	0.1351	0.1203	0.0257	3.0106E-06	6,666	21.846	TRUE
ebi-PL179-GCST90277346	Phosphatidylethanolamine (16:0_20:4) levels	rs79450270	2	138,745,944	A	G	0.0127	0.3833	0.0820	3.0349E-06	6,666	21.830	TRUE
ebi-PL179-GCST90277346	Phosphatidylethanolamine (16:0_20:4) levels	rs149460121	9	137,858,500	A	T	0.1090	0.1453	0.0291	5.9111E-07	6,666	24.987	TRUE
ebi-PL179-GCST90277346	Phosphatidylethanolamine (16:0_20:4) levels	rs61999891	15	58,007,401	A	G	0.1039	0.1610	0.0290	3.1037E-08	6,666	30.719	TRUE
ebi-PL179-GCST90277346	Phosphatidylethanolamine (16:0_20:4) levels	rs72679383	4	100,929,253	T	G	0.0216	0.3132	0.0608	2.6535E-07	6,666	26.539	TRUE
ebi-PL179-GCST90277346	Phosphatidylethanolamine (16:0_20:4) levels	rs62155727	2	101,740,535	G	A	0.1510	-0.1214	0.0240	4.4200E-07	6,666	25.551	TRUE
ebi-PL179-GCST90277346	Phosphatidylethanolamine (16:0_20:4) levels	rs10892181	11	117,828,871	G	T	0.4656	0.0803	0.0174	4.1670E-06	6,666	21.218	TRUE

Lipid GWAS ID	Lipid species	SNP	Chromosome	Position	Effect allele	Other allele	Effect-allele frequency	Beta	SE	P value	Sample size	F statistic	F > 10
ebi-PL179-GCST90277346	Phosphatidylethanolamine (16:0_20:4) levels	rs1815786	11	117,050,674	C	T	0.8662	-0.1340	0.0254	1.4314E-07	6,666	27.738	TRUE
ebi-PL179-GCST90277269	Phosphatidylethanolamine (18:0_0:0) levels	rs79577600	17	49,966,298	T	G	0.0958	0.1460	0.0312	2.8852E-06	6,258	21.930	TRUE
ebi-PL179-GCST90277269	Phosphatidylethanolamine (18:0_0:0) levels	rs12196051	6	142,892,562	A	G	0.0244	-0.2919	0.0592	8.5026E-07	6,258	24.288	TRUE
ebi-PL179-GCST90277269	Phosphatidylethanolamine (18:0_0:0) levels	rs6713691	2	9,689,106	G	A	0.1970	-0.1103	0.0231	1.7577E-06	6,258	22.886	TRUE
ebi-PL179-GCST90277269	Phosphatidylethanolamine (18:0_0:0) levels	rs4663191	2	235,266,114	G	A	0.7461	0.0972	0.0207	2.6420E-06	6,258	22.099	TRUE
ebi-PL179-GCST90277269	Phosphatidylethanolamine (18:0_0:0) levels	rs35152003	8	18,433,891	A	T	0.6619	-0.0969	0.0191	4.0498E-07	6,258	25.723	TRUE
ebi-PL179-GCST90277269	Phosphatidylethanolamine (18:0_0:0) levels	rs72757442	5	54,771,412	T	C	0.0489	0.2165	0.0433	5.8311E-07	6,258	25.017	TRUE
ebi-PL179-GCST90277269	Phosphatidylethanolamine (18:0_0:0) levels	rs16918167	9	99,053,057	T	G	0.1511	-0.1189	0.0250	1.9638E-06	6,258	22.671	TRUE
ebi-PL179-GCST90277269	Phosphatidylethanolamine (18:0_0:0) levels	rs12726330	1	155,135,691	A	G	0.0429	-0.2320	0.0450	2.5429E-07	6,258	26.624	TRUE
ebi-PL179-GCST90277269	Phosphatidylethanolamine (18:0_0:0) levels	rs116987919	7	70,998,624	G	C	0.0163	0.3398	0.0727	2.9829E-06	6,258	21.866	TRUE
ebi-PL179-GCST90277347	Phosphatidylethanolamine (18:0_18:2) levels	rs34733845	12	60,861,641	T	C	0.0592	0.1651	0.0357	3.8601E-06	7,128	21.366	TRUE
ebi-PL179-GCST90277347	Phosphatidylethanolamine (18:0_18:2) levels	rs419482	6	94,336,592	T	A	0.4080	-0.0782	0.0170	4.1208E-06	7,128	21.239	TRUE
ebi-PL179-GCST90277347	Phosphatidylethanolamine (18:0_18:2) levels	rs62434228	6	154,681,357	A	G	0.1603	0.1086	0.0230	2.3525E-06	7,128	22.318	TRUE
ebi-PL179-GCST90277347	Phosphatidylethanolamine (18:0_18:2) levels	rs62291572	4	4,996,200	T	C	0.2144	0.1002	0.0210	1.9563E-06	7,128	22.674	TRUE
ebi-PL179-GCST90277347	Phosphatidylethanolamine (18:0_18:2) levels	rs174567	11	61,825,533	G	A	0.4092	0.3431	0.0166	1.2512E-92	7,128	429.151	TRUE
ebi-PL179-GCST90277347	Phosphatidylethanolamine (18:0_18:2) levels	rs805292	6	31,722,232	A	G	0.3779	0.0850	0.0174	1.0979E-06	7,128	23.788	TRUE
ebi-PL179-GCST90277347	Phosphatidylethanolamine (18:0_18:2) levels	rs112129861	15	59,047,450	C	G	0.0648	0.1560	0.0338	3.9090E-06	7,128	21.341	TRUE
ebi-PL179-GCST90277347	Phosphatidylethanolamine (18:0_18:2) levels	rs13110318	4	38,137,235	A	G	0.1139	-0.1210	0.0261	3.7363E-06	7,128	21.425	TRUE
ebi-PL179-GCST90277347	Phosphatidylethanolamine (18:0_18:2) levels	rs10069895	5	86,297,284	C	T	0.7643	0.0972	0.0196	7.1828E-07	7,128	24.611	TRUE
ebi-PL179-GCST90277347	Phosphatidylethanolamine (18:0_18:2) levels	rs117376818	15	58,506,762	A	G	0.0158	0.6304	0.0704	4.1620E-19	7,128	80.236	TRUE
ebi-PL179-GCST90277347	Phosphatidylethanolamine (18:0_18:2) levels	rs111543310	15	59,239,619	C	T	0.0223	0.2803	0.0604	3.5515E-06	7,128	21.526	TRUE
ebi-PL179-GCST90277347	Phosphatidylethanolamine (18:0_18:2) levels	rs138283783	15	58,244,328	C	T	0.0266	0.3517	0.0536	5.5569E-11	7,128	43.100	TRUE
ebi-PL179-GCST90277347	Phosphatidylethanolamine (18:0_18:2) levels	rs73424597	15	58,607,410	C	T	0.0350	0.3547	0.0453	5.8835E-15	7,128	61.199	TRUE
ebi-PL179-GCST90277347	Phosphatidylethanolamine (18:0_18:2) levels	rs12928099	16	15,056,648	A	C	0.2744	-0.1126	0.0186	1.4952E-09	7,128	36.630	TRUE
ebi-PL179-GCST90277347	Phosphatidylethanolamine (18:0_18:2) levels	rs4296402	2	174,696,563	A	G	0.1596	0.1057	0.0230	4.4891E-06	7,128	21.074	TRUE
ebi-PL179-GCST90277347	Phosphatidylethanolamine (18:0_18:2) levels	rs117900629	11	62,205,328	A	G	0.0618	-0.2135	0.0345	6.2522E-10	7,128	38.348	TRUE
ebi-PL179-GCST90277348	Phosphatidylethanolamine (18:0_20:4) levels	rs10750098	11	116,834,852	T	G	0.8631	-0.1495	0.0245	1.0895E-09	7,164	37.254	TRUE
ebi-PL179-GCST90277348	Phosphatidylethanolamine (18:0_20:4) levels	rs116866162	8	11,624,851	A	G	0.0251	-0.2601	0.0548	2.0729E-06	7,164	22.561	TRUE
ebi-PL179-GCST90277348	Phosphatidylethanolamine (18:0_20:4) levels	rs28395406	15	58,337,150	G	A	0.1306	-0.1958	0.0247	2.3413E-15	7,164	63.035	TRUE
ebi-PL179-GCST90277348	Phosphatidylethanolamine (18:0_20:4) levels	rs17798605	18	47,575,930	G	A	0.0381	0.2149	0.0456	2.4323E-06	7,164	22.254	TRUE
ebi-PL179-GCST90277348	Phosphatidylethanolamine (18:0_20:4) levels	rs73964403	18	67,212,380	G	C	0.0318	-0.2267	0.0482	2.5539E-06	7,164	22.159	TRUE
ebi-PL179-GCST90277348	Phosphatidylethanolamine (18:0_20:4) levels	rs4272979	15	59,414,071	T	C	0.1497	0.1332	0.0237	1.8676E-08	7,164	31.698	TRUE
ebi-PL179-GCST90277348	Phosphatidylethanolamine (18:0_20:4) levels	rs11031419	11	31,584,349	T	A	0.0586	0.1766	0.0354	6.1290E-07	7,164	24.914	TRUE
ebi-PL179-GCST90277348	Phosphatidylethanolamine (18:0_20:4) levels	rs62549032	9	74,092,748	A	G	0.0307	0.2260	0.0488	3.7063E-06	7,164	21.443	TRUE
ebi-PL179-GCST90277348	Phosphatidylethanolamine (18:0_20:4) levels	rs6494025	15	58,584,587	T	C	0.0345	0.3421	0.0455	6.0428E-14	7,164	56.581	TRUE
ebi-PL179-GCST90277270	Phosphatidylethanolamine (18:1_0:0) levels	rs9826895	3	23,781,447	A	G	0.2723	-0.1031	0.0220	2.9534E-06	5,172	21.899	TRUE
ebi-PL179-GCST90277270	Phosphatidylethanolamine (18:1_0:0) levels	rs6768813	3	142,947,149	C	T	0.6288	0.1095	0.0204	7.7414E-08	5,172	28.948	TRUE
ebi-PL179-GCST90277270	Phosphatidylethanolamine (18:1_0:0) levels	rs7636653	3	73,256,915	A	T	0.4763	-0.0996	0.0196	4.1262E-07	5,172	25.697	TRUE
ebi-PL179-GCST90277270	Phosphatidylethanolamine (18:1_0:0) levels	rs149492888	5	99,192,949	A	G	0.0092	-0.5254	0.1130	3.3994E-06	5,172	21.624	TRUE
ebi-PL179-GCST90277270	Phosphatidylethanolamine (18:1_0:0) levels	rs11917269	3	50,443,604	T	G	0.2773	0.1085	0.0218	6.7494E-07	5,172	24.747	TRUE
ebi-PL179-GCST90277270	Phosphatidylethanolamine (18:1_0:0) levels	rs75388820	2	27,102,273	A	G	0.0468	0.2281	0.0469	1.1858E-06	5,172	23.654	TRUE
ebi-PL179-GCST90277270	Phosphatidylethanolamine (18:1_0:0) levels	rs35584640	6	73,616,238	A	G	0.2177	-0.1081	0.0236	4.8949E-06	5,172	20.921	TRUE
ebi-PL179-GCST90277270	Phosphatidylethanolamine (18:1_0:0) levels	rs7776911	7	50,816,756	A	G	0.8410	-0.1372	0.0268	3.2190E-07	5,172	26.176	TRUE
ebi-PL179-GCST90277270	Phosphatidylethanolamine (18:1_0:0) levels	rs2585266	17	5,310,096	T	C	0.0443	-0.2412	0.0487	7.6710E-07	5,172	24.502	TRUE
ebi-PL179-GCST90277349	Phosphatidylethanolamine (18:1_18:1) levels	rs16874868	5	5,149,248	T	A	0.1058	-0.1273	0.0278	4.6165E-06	7,045	21.021	TRUE
ebi-PL179-GCST90277349	Phosphatidylethanolamine (18:1_18:1) levels	rs78564380	2	13,358,463	A	G	0.0160	0.3463	0.0718	1.4638E-06	7,045	23.233	TRUE
ebi-PL179-GCST90277349	Phosphatidylethanolamine (18:1_18:1) levels	rs17174502	11	116,888,939	A	G	0.1744	0.1358	0.0223	1.1400E-09	7,045	37.165	TRUE
ebi-PL179-GCST90277349	Phosphatidylethanolamine (18:1_18:1) levels	rs12909585	15	57,610,766	A	C	0.4388	0.0962	0.0171	1.8625E-08	7,045	31.707	TRUE
ebi-PL179-GCST90277349	Phosphatidylethanolamine (18:1_18:1) levels	rs74899875	2	11,870,210	T	C	0.0516	0.1840	0.0397	3.5966E-06	7,045	21.502	TRUE
ebi-PL179-GCST90277349	Phosphatidylethanolamine (18:1_18:1) levels	rs2946819	3	118,500,064	T	G	0.9117	-0.1431	0.0303	2.3784E-06	7,045	22.300	TRUE
ebi-PL179-GCST90277349	Phosphatidylethanolamine (18:1_18:1) levels	rs72880096	17	77,018,065	C	T	0.0991	0.1364	0.0285	1.7545E-06	7,045	22.884	TRUE
ebi-PL179-GCST90277349	Phosphatidylethanolamine (18:1_18:1) levels	rs11633043	15	58,545,523	A	G	0.1909	0.1355	0.0217	4.3384E-10	7,045	39.057	TRUE
ebi-PL179-GCST90277349	Phosphatidylethanolamine (18:1_18:1) levels	rs140764699	9	92,627,750	A	G	0.0070	-0.4763	0.1020	3.0847E-06	7,045	21.797	TRUE
ebi-PL179-GCST90277349	Phosphatidylethanolamine (18:1_18:1) levels	rs76569455	15	59,137,959	A	G	0.0647	0.1950	0.0340	1.0260E-08	7,045	32.869	TRUE
ebi-PL179-GCST90277271	Phosphatidylethanolamine (18:2_0:0) levels	rs62076866	17	38,791,988	T	C	0.0128	0.3875	0.0766	4.3769E-07	6,905	25.568	TRUE
ebi-PL179-GCST90277271	Phosphatidylethanolamine (18:2_0:0) levels	rs6413913	1	189,909,464	A	G	0.3090	-0.0879	0.0183	1.4957E-06	6,905	23.192	TRUE
ebi-PL179-GCST90277271	Phosphatidylethanolamine (18:2_0:0) levels	rs1442407	8	3,234,062	T	G	0.1788	-0.1159	0.0221	1.6019E-07	6,905	27.518	TRUE
ebi-PL179-GCST90277271	Phosphatidylethanolamine (18:2_0:0) levels	rs11063393	12	4,892,157	G	A	0.1025	-0.1338	0.0287	3.2157E-06	6,905	21.717	TRUE

Lipid GWAS ID	Lipid species	SNP	Chromosome	Position	Effect allele	Other allele	Effect-allele frequency	Beta	SE	P value	Sample size	F statistic	F > 10
ebi-PL179-GCST90277271	Phosphatidylethanolamine (18:2_0:0) levels	rs1338449	1	218,177,981	C	T	0.3490	0.0822	0.0180	4.9831E-06	6,905	20.876	TRUE
ebi-PL179-GCST90277271	Phosphatidylethanolamine (18:2_0:0) levels	rs114899411	4	152,031,948	A	G	0.0575	-0.1885	0.0375	5.0822E-07	6,905	25.278	TRUE
ebi-PL179-GCST90277271	Phosphatidylethanolamine (18:2_0:0) levels	rs11045856	12	21,197,755	G	T	0.1661	-0.1184	0.0229	2.4916E-07	6,905	26.660	TRUE
ebi-PL179-GCST90277271	Phosphatidylethanolamine (18:2_0:0) levels	rs188404615	12	99,719,293	C	T	0.0068	0.5443	0.1036	1.5320E-07	6,905	27.603	TRUE
ebi-PL179-GCST90277271	Phosphatidylethanolamine (18:2_0:0) levels	rs146330404	8	2,533,493	C	G	0.0059	0.5605	0.1151	1.1420E-06	6,905	23.714	TRUE
ebi-PL179-GCST90277271	Phosphatidylethanolamine (18:2_0:0) levels	rs326048	5	996,057	G	A	0.2854	-0.0896	0.0195	4.5970E-06	6,905	21.030	TRUE
ebi-PL179-GCST90277271	Phosphatidylethanolamine (18:2_0:0) levels	rs35140833	18	5,841,984	G	A	0.1537	-0.1148	0.0243	2.2996E-06	6,905	22.363	TRUE
ebi-PL179-GCST90277271	Phosphatidylethanolamine (18:2_0:0) levels	rs111877698	1	201,480,758	C	A	0.0126	0.4228	0.0867	1.0960E-06	6,905	23.793	TRUE
ebi-PL179-GCST90277271	Phosphatidylethanolamine (18:2_0:0) levels	rs3741259	11	61,514,878	T	C	0.1158	0.1388	0.0266	1.8624E-07	6,905	27.225	TRUE
ebi-PL179-GCST90277350	Phosphatidylethanolamine (O-16:1_18:2) levels	rs311415	8	54,198,654	G	C	0.5882	-0.0918	0.0195	2.6500E-06	5,430	22.098	TRUE
ebi-PL179-GCST90277350	Phosphatidylethanolamine (O-16:1_18:2) levels	rs62294904	3	182,462,564	T	A	0.0976	0.1624	0.0349	3.3023E-06	5,430	21.676	TRUE
ebi-PL179-GCST90277350	Phosphatidylethanolamine (O-16:1_18:2) levels	rs9590678	13	42,237,414	A	T	0.4102	0.0982	0.0198	7.5365E-07	5,430	24.530	TRUE
ebi-PL179-GCST90277350	Phosphatidylethanolamine (O-16:1_18:2) levels	rs12997068	2	42,197,717	A	G	0.0294	0.2733	0.0565	1.3616E-06	5,430	23.385	TRUE
ebi-PL179-GCST90277350	Phosphatidylethanolamine (O-16:1_18:2) levels	rs602307	5	73,116,953	A	T	0.4031	0.0908	0.0196	3.8670E-06	5,430	21.373	TRUE
ebi-PL179-GCST90277350	Phosphatidylethanolamine (O-16:1_18:2) levels	rs78655391	1	110,525,431	C	T	0.0658	0.1901	0.0399	1.9707E-06	5,430	22.670	TRUE
ebi-PL179-GCST90277350	Phosphatidylethanolamine (O-16:1_18:2) levels	rs5757363	22	38,910,264	C	G	0.3218	-0.1034	0.0209	7.9313E-07	5,430	24.430	TRUE
ebi-PL179-GCST90277351	Phosphatidylethanolamine (O-16:1_20:4) levels	rs7445920	5	74,678,000	T	C	0.1931	0.0966	0.0211	4.8187E-06	7,169	20.938	TRUE
ebi-PL179-GCST90277351	Phosphatidylethanolamine (O-16:1_20:4) levels	rs9265944	6	31,347,332	C	T	0.3913	0.0835	0.0171	1.1267E-06	7,169	23.738	TRUE
ebi-PL179-GCST90277351	Phosphatidylethanolamine (O-16:1_20:4) levels	rs79337987	7	138,155,111	A	T	0.0195	-0.2942	0.0608	1.3202E-06	7,169	23.433	TRUE
ebi-PL179-GCST90277351	Phosphatidylethanolamine (O-16:1_20:4) levels	rs10494405	1	163,979,236	C	T	0.1063	-0.1266	0.0276	4.6204E-06	7,169	21.019	TRUE
ebi-PL179-GCST90277351	Phosphatidylethanolamine (O-16:1_20:4) levels	rs2573210	2	232,520,315	A	G	0.8378	0.1134	0.0227	6.3410E-07	7,169	24.848	TRUE
ebi-PL179-GCST90277351	Phosphatidylethanolamine (O-16:1_20:4) levels	rs13123798	4	183,919,435	C	G	0.5891	0.0847	0.0172	8.1050E-07	7,169	24.375	TRUE
ebi-PL179-GCST90277351	Phosphatidylethanolamine (O-16:1_20:4) levels	rs74677222	12	121,783,011	A	G	0.0830	0.1457	0.0305	1.7858E-06	7,169	22.848	TRUE
ebi-PL179-GCST90277351	Phosphatidylethanolamine (O-16:1_20:4) levels	rs204474	19	44,983,921	T	C	0.5984	-0.0841	0.0171	9.1521E-07	7,169	24.139	TRUE
ebi-PL179-GCST90277351	Phosphatidylethanolamine (O-16:1_20:4) levels	rs11725298	4	52,717,255	C	T	0.0223	0.2592	0.0567	4.9582E-06	7,169	20.884	TRUE
ebi-PL179-GCST90277351	Phosphatidylethanolamine (O-16:1_20:4) levels	rs3826884	19	55,226,813	A	G	0.1492	-0.1791	0.0236	3.5555E-14	7,169	57.632	TRUE
ebi-PL179-GCST90277351	Phosphatidylethanolamine (O-16:1_20:4) levels	rs2161365	10	12,818,748	G	A	0.4693	0.0785	0.0171	4.4925E-06	7,169	21.073	TRUE
ebi-PL179-GCST90277351	Phosphatidylethanolamine (O-16:1_20:4) levels	rs10234951	7	94,419,222	G	T	0.0800	-0.1509	0.0310	1.1689E-06	7,169	23.667	TRUE
ebi-PL179-GCST90277351	Phosphatidylethanolamine (O-16:1_20:4) levels	rs77758816	18	4,534,021	A	G	0.0288	-0.2409	0.0508	2.1563E-06	7,169	22.486	TRUE
ebi-PL179-GCST90277351	Phosphatidylethanolamine (O-16:1_20:4) levels	rs1034755	7	8,811,155	T	C	0.3194	0.0850	0.0179	2.1347E-06	7,169	22.504	TRUE
ebi-PL179-GCST90277351	Phosphatidylethanolamine (O-16:1_20:4) levels	rs97384	11	61,856,709	C	T	0.5803	0.2099	0.0169	4.3357E-35	7,169	154.391	TRUE
ebi-PL179-GCST90277351	Phosphatidylethanolamine (O-16:1_20:4) levels	rs11136432	8	1,843,472	G	T	0.6021	-0.0805	0.0171	2.7386E-06	7,169	22.026	TRUE
ebi-PL179-GCST90277352	Phosphatidylethanolamine (O-16:1_22:5) levels	rs190716231	7	133,098,676	C	G	0.0212	-0.3087	0.0669	3.9721E-06	5,609	21.319	TRUE
ebi-PL179-GCST90277352	Phosphatidylethanolamine (O-16:1_22:5) levels	rs41264497	1	39,624,750	G	A	0.0057	-0.6343	0.1269	5.9639E-07	5,609	24.978	TRUE
ebi-PL179-GCST90277352	Phosphatidylethanolamine (O-16:1_22:5) levels	rs7028232	9	114,255,513	T	C	0.3736	0.0934	0.0200	2.9528E-06	5,609	21.887	TRUE
ebi-PL179-GCST90277352	Phosphatidylethanolamine (O-16:1_22:5) levels	rs10187082	2	140,761,432	G	C	0.0067	-0.5267	0.1146	4.4010E-06	5,609	21.125	TRUE
ebi-PL179-GCST90277352	Phosphatidylethanolamine (O-16:1_22:5) levels	rs4974228	3	60,777,864	C	T	0.0921	0.1542	0.0327	2.4273E-06	5,609	22.271	TRUE
ebi-PL179-GCST90277353	Phosphatidylethanolamine (O-18:1_18:2) levels	rs190712692	19	44,921,921	A	G	0.0302	0.2449	0.0509	1.5053E-06	6,870	23.179	TRUE
ebi-PL179-GCST90277353	Phosphatidylethanolamine (O-18:1_18:2) levels	rs55683433	4	74,887,304	A	C	0.0424	-0.2100	0.0436	1.5161E-06	6,870	23.163	TRUE
ebi-PL179-GCST90277353	Phosphatidylethanolamine (O-18:1_18:2) levels	rs76085699	1	206,738,030	A	G	0.1343	-0.1222	0.0253	1.4247E-06	6,870	23.289	TRUE
ebi-PL179-GCST90277353	Phosphatidylethanolamine (O-18:1_18:2) levels	rs261334	15	58,434,545	C	G	0.7583	-0.1041	0.0200	1.9139E-07	6,870	27.172	TRUE
ebi-PL179-GCST90277353	Phosphatidylethanolamine (O-18:1_18:2) levels	rs7003304	8	14,334,458	T	C	0.1389	-0.1203	0.0250	1.5395E-06	6,870	23.137	TRUE
ebi-PL179-GCST90277353	Phosphatidylethanolamine (O-18:1_18:2) levels	rs10848158	12	130,607,421	C	T	0.4337	0.0934	0.0173	7.1656E-08	6,870	29.081	TRUE
ebi-PL179-GCST90277353	Phosphatidylethanolamine (O-18:1_18:2) levels	rs187748847	3	181,603,334	G	A	0.0052	-0.5869	0.1241	2.2901E-06	6,870	22.371	TRUE

Lipid GWAS ID	Lipid species	SNP	Chromosome	Position	Effect allele	Other allele	Effect-allele frequency	Beta	SE	P value	Sample size	F statistic	F > 10
ebi-PL179-GCST90277353	Phosphatidylethanolamine (O-18:1_18:2) levels	rs28844909	4	145,707,221	A	G	0.0666	-0.1670	0.0348	1.6929E-06	6,870	22.955	TRUE
ebi-PL179-GCST90277353	Phosphatidylethanolamine (O-18:1_18:2) levels	rs11936543	4	151,270,229	G	A	0.0998	-0.1430	0.0289	7.7045E-07	6,870	24.474	TRUE
ebi-PL179-GCST90277353	Phosphatidylethanolamine (O-18:1_18:2) levels	rs17019568	4	92,558,738	G	C	0.0880	0.1510	0.0304	7.0963E-07	6,870	24.633	TRUE
ebi-PL179-GCST90277353	Phosphatidylethanolamine (O-18:1_18:2) levels	rs111703312	2	204,509,075	G	A	0.0157	0.3344	0.0706	2.1916E-06	6,870	22.453	TRUE
ebi-PL179-GCST90277353	Phosphatidylethanolamine (O-18:1_18:2) levels	rs10811102	9	1,911,291	T	G	0.1954	-0.1065	0.0225	2.2778E-06	6,870	22.380	TRUE
ebi-PL179-GCST90277353	Phosphatidylethanolamine (O-18:1_18:2) levels	rs57684876	19	44,710,429	T	C	0.1874	0.1023	0.0218	2.6380E-06	6,870	22.101	TRUE
ebi-PL179-GCST90277353	Phosphatidylethanolamine (O-18:1_18:2) levels	rs117511403	18	35,300,850	A	G	0.0037	0.6970	0.1430	1.1229E-06	6,870	23.746	TRUE
ebi-PL179-GCST90277354	Phosphatidylethanolamine (O-18:1_20:4) levels	rs2277289	11	118,311,945	C	T	0.3263	0.0911	0.0178	3.3135E-07	7,172	26.106	TRUE
ebi-PL179-GCST90277354	Phosphatidylethanolamine (O-18:1_20:4) levels	rs113913626	12	76,255,614	T	C	0.0323	-0.2246	0.0482	3.2741E-06	7,172	21.682	TRUE
ebi-PL179-GCST90277354	Phosphatidylethanolamine (O-18:1_20:4) levels	rs71420341	2	59,638,953	G	A	0.0893	-0.1462	0.0295	7.5442E-07	7,172	24.513	TRUE
ebi-PL179-GCST90277354	Phosphatidylethanolamine (O-18:1_20:4) levels	rs1513795	2	123,647,385	C	T	0.4294	0.0800	0.0170	2.4741E-06	7,172	22.221	TRUE
ebi-PL179-GCST90277354	Phosphatidylethanolamine (O-18:1_20:4) levels	rs141622900	19	44,923,535	A	G	0.0303	0.2496	0.0501	6.5158E-07	7,172	24.797	TRUE
ebi-PL179-GCST90277354	Phosphatidylethanolamine (O-18:1_20:4) levels	rs35427954	16	76,463,097	C	T	0.0870	-0.1399	0.0296	2.2976E-06	7,172	22.364	TRUE
ebi-PL179-GCST90277354	Phosphatidylethanolamine (O-18:1_20:4) levels	rs204468	19	44,987,378	C	T	0.5950	-0.0953	0.0170	2.3490E-08	7,172	31.251	TRUE

Lipid GWAS ID	Lipid species	SNP	Chromosome	Position	Effect allele	Other allele	Effect-allele frequency	Beta	SE	P value	Sample size	F statistic	F > 10
ebi-PL179-GCST90277354	Phosphatidylethanolamine (O-18:1_20:4) levels	rs11062287	12	2,630,538	A	G	0.0339	0.2547	0.0504	4.4452E-07	7,172	25.536	TRUE
ebi-PL179-GCST90277354	Phosphatidylethanolamine (O-18:1_20:4) levels	rs17703316	13	75,596,558	T	G	0.0504	0.1792	0.0389	4.2452E-06	7,172	21.182	TRUE
ebi-PL179-GCST90277354	Phosphatidylethanolamine (O-18:1_20:4) levels	rs72635955	1	244,982,806	T	C	0.1246	0.1260	0.0251	5.1637E-07	7,172	25.247	TRUE
ebi-PL179-GCST90277354	Phosphatidylethanolamine (O-18:1_20:4) levels	rs117565387	16	5,924,670	T	C	0.0532	0.1749	0.0377	3.5094E-06	7,172	21.550	TRUE
ebi-PL179-GCST90277354	Phosphatidylethanolamine (O-18:1_20:4) levels	rs41376249	22	36,714,714	A	G	0.1459	-0.1121	0.0236	2.0793E-06	7,172	22.556	TRUE
ebi-PL179-GCST90277355	Phosphatidylethanolamine (O-18:2_18:1) levels	rs79878108	1	245,122,916	T	C	0.0291	-0.2606	0.0518	5.0762E-07	6,717	25.282	TRUE
ebi-PL179-GCST90277355	Phosphatidylethanolamine (O-18:2_18:1) levels	rs112243303	1	224,192,928	T	C	0.1115	0.1384	0.0277	5.8583E-07	6,717	25.002	TRUE
ebi-PL179-GCST90277355	Phosphatidylethanolamine (O-18:2_18:1) levels	rs2271489	3	39,235,168	G	C	0.0815	-0.1719	0.0318	6.6718E-08	6,717	29.220	TRUE
ebi-PL179-GCST90277355	Phosphatidylethanolamine (O-18:2_18:1) levels	rs17368528	1	9,264,154	T	C	0.0843	0.1609	0.0309	2.0445E-07	6,717	27.046	TRUE
ebi-PL179-GCST90277355	Phosphatidylethanolamine (O-18:2_18:1) levels	rs140484398	9	102,668,496	A	G	0.0069	0.5194	0.1062	1.0170E-06	6,717	23.938	TRUE
ebi-PL179-GCST90277355	Phosphatidylethanolamine (O-18:2_18:1) levels	rs28606541	19	6,934,007	T	C	0.0427	-0.2068	0.0432	1.7140E-06	6,717	22.932	TRUE
ebi-PL179-GCST90277355	Phosphatidylethanolamine (O-18:2_18:1) levels	rs35253870	2	12,232,463	G	A	0.1508	0.1182	0.0245	1.3938E-06	6,717	23.329	TRUE
ebi-PL179-GCST90277355	Phosphatidylethanolamine (O-18:2_18:1) levels	rs12496575	3	236,587	G	A	0.6934	-0.0897	0.0187	1.6474E-06	6,717	23.007	TRUE
ebi-PL179-GCST90277355	Phosphatidylethanolamine (O-18:2_18:1) levels	rs192913427	2	63,521,077	A	G	0.0030	0.7183	0.1522	2.4207E-06	6,717	22.265	TRUE
ebi-PL179-GCST90277355	Phosphatidylethanolamine (O-18:2_18:1) levels	rs13257713	8	112,417,407	T	C	0.1392	-0.1190	0.0250	2.0603E-06	6,717	22.573	TRUE
ebi-PL179-GCST90277355	Phosphatidylethanolamine (O-18:2_18:1) levels	rs6749797	2	120,398,486	A	G	0.2286	-0.0980	0.0205	1.7371E-06	6,717	22.903	TRUE
ebi-PL179-GCST90277355	Phosphatidylethanolamine (O-18:2_18:1) levels	rs8108277	19	44,702,607	C	T	0.1887	0.1062	0.0218	1.1313E-06	6,717	23.736	TRUE
ebi-PL179-GCST90277356	Phosphatidylethanolamine (O-18:2_18:2) levels	rs34683829	2	216,708,722	T	C	0.2061	-0.1041	0.0220	2.3608E-06	6,337	22.316	TRUE
ebi-PL179-GCST90277356	Phosphatidylethanolamine (O-18:2_18:2) levels	rs2727270	11	61,835,765	T	C	0.2576	0.1769	0.0202	2.4296E-18	6,337	76.760	TRUE
ebi-PL179-GCST90277356	Phosphatidylethanolamine (O-18:2_18:2) levels	rs79998109	12	109,070,443	T	C	0.0071	0.5401	0.1141	2.2418E-06	6,337	22.415	TRUE
ebi-PL179-GCST90277356	Phosphatidylethanolamine (O-18:2_18:2) levels	rs238237	17	4,999,663	A	G	0.7310	-0.0933	0.0201	3.6998E-06	6,337	21.449	TRUE
ebi-PL179-GCST90277356	Phosphatidylethanolamine (O-18:2_18:2) levels	rs4603290	11	15,301,207	C	G	0.0986	0.1532	0.0306	5.9378E-07	6,337	24.981	TRUE
ebi-PL179-GCST90277356	Phosphatidylethanolamine (O-18:2_18:2) levels	rs2550882	16	75,515,447	G	C	0.4135	-0.0850	0.0180	2.3610E-06	6,337	22.316	TRUE
ebi-PL179-GCST90277356	Phosphatidylethanolamine (O-18:2_18:2) levels	rs540822327	19	5,904,792	A	G	0.0314	-0.2612	0.0529	8.0635E-07	6,337	24.390	TRUE
ebi-PL179-GCST90277357	Phosphatidylethanolamine (O-18:2_20:4) levels	rs115623452	3	106,127,782	A	G	0.0027	-0.8035	0.1744	4.1441E-06	7,154	21.229	TRUE
ebi-PL179-GCST90277357	Phosphatidylethanolamine (O-18:2_20:4) levels	rs61792775	3	132,262,400	T	C	0.0730	-0.1529	0.0328	3.2291E-06	7,154	21.708	TRUE
ebi-PL179-GCST90277357	Phosphatidylethanolamine (O-18:2_20:4) levels	rs17864186	7	127,303,602	A	C	0.0743	0.1472	0.0322	4.9678E-06	7,154	20.879	TRUE
ebi-PL179-GCST90277357	Phosphatidylethanolamine (O-18:2_20:4) levels	rs71366199	17	15,393,870	G	A	0.1477	0.1213	0.0241	4.8728E-07	7,154	25.358	TRUE
ebi-PL179-GCST90277357	Phosphatidylethanolamine (O-18:2_20:4) levels	rs143745495	1	119,172,555	T	C	0.1724	-0.1044	0.0221	2.3779E-06	7,154	22.297	TRUE
ebi-PL179-GCST90277357	Phosphatidylethanolamine (O-18:2_20:4) levels	rs181750334	16	14,645,161	T	A	0.0075	-0.5375	0.1031	1.9077E-07	7,154	27.176	TRUE
ebi-PL179-GCST90277357	Phosphatidylethanolamine (O-18:2_20:4) levels	rs13280051	8	8,374,175	G	A	0.2466	0.0925	0.0194	1.8826E-06	7,154	22.745	TRUE
ebi-PL179-GCST90277357	Phosphatidylethanolamine (O-18:2_20:4) levels	rs174556	11	61,813,163	T	C	0.3796	-0.1826	0.0172	4.1597E-26	7,154	112.579	TRUE
ebi-PL179-GCST90277358	Phosphatidylinositol (16:0_18:1) levels	rs76902173	12	29,000,949	G	C	0.0217	-0.2934	0.0587	5.8084E-07	7,157	25.018	TRUE
ebi-PL179-GCST90277358	Phosphatidylinositol (16:0_18:1) levels	rs9564991	13	73,455,183	T	C	0.1297	-0.1187	0.0250	2.0584E-06	7,157	22.622	TRUE
ebi-PL179-GCST90277358	Phosphatidylinositol (16:0_18:1) levels	rs117226992	16	51,313,598	G	C	0.0032	0.7340	0.1586	3.7822E-06	7,157	21.405	TRUE
ebi-PL179-GCST90277358	Phosphatidylinositol (16:0_18:1) levels	rs1540037	18	49,656,294	G	A	0.7569	0.0995	0.0198	5.0721E-07	7,157	25.283	TRUE
ebi-PL179-GCST90277358	Phosphatidylinositol (16:0_18:1) levels	rs80310784	10	52,382,881	A	G	0.0600	0.1678	0.0361	3.4379E-06	7,157	21.588	TRUE
ebi-PL179-GCST90277359	Phosphatidylinositol (16:0_18:2) levels	rs2264782	12	120,994,800	T	C	0.3586	0.0846	0.0176	1.6520E-06	6,998	23.000	TRUE
ebi-PL179-GCST90277359	Phosphatidylinositol (16:0_18:2) levels	rs141824100	16	88,985,800	T	C	0.0505	-0.1846	0.0387	1.9251E-06	6,998	22.705	TRUE
ebi-PL179-GCST90277359	Phosphatidylinositol (16:0_18:2) levels	rs17427389	6	150,841,979	A	G	0.1912	0.1080	0.0215	4.9558E-07	6,998	25.327	TRUE
ebi-PL179-GCST90277359	Phosphatidylinositol (16:0_18:2) levels	rs34777947	7	105,583,433	C	T	0.1893	0.1027	0.0216	1.9915E-06	6,998	22.640	TRUE
ebi-PL179-GCST90277359	Phosphatidylinositol (16:0_18:2) levels	rs2274890	20	25,320,386	A	G	0.2649	0.1024	0.0191	8.3193E-08	6,998	28.789	TRUE
ebi-PL179-GCST90277359	Phosphatidylinositol (16:0_18:2) levels	rs3786247	18	49,592,553	G	T	0.0964	0.1582	0.0290	5.2491E-08	6,998	29.688	TRUE
ebi-PL179-GCST90277359	Phosphatidylinositol (16:0_18:2) levels	rs174549	11	61,803,910	A	G	0.3820	0.1641	0.0174	5.7491E-21	6,998	88.814	TRUE
ebi-PL179-GCST90277359	Phosphatidylinositol (16:0_18:2) levels	rs11669648	19	54,038,742	A	G	0.1725	0.1072	0.0229	2.7998E-06	6,998	21.983	TRUE

Lipid GWAS ID	Lipid species	SNP	Chromosome	Position	Effect allele	Other allele	Effect-allele frequency	Beta	SE	P value	Sample size	F statistic	F > 10
ebi-PL179-GCST90277359	Phosphatidylinositol (16:0_18:2) levels	rs115506950	2	36,921,090	A	T	0.0363	0.2222	0.0461	1.4607E-06	6,998	23.238	TRUE
ebi-PL179-GCST90277359	Phosphatidylinositol (16:0_18:2) levels	rs28675901	4	108,839,211	C	T	0.1282	0.1243	0.0255	1.1093E-06	6,998	23.769	TRUE
ebi-PL179-GCST90277359	Phosphatidylinositol (16:0_18:2) levels	rs7241918	18	49,634,583	T	G	0.8208	0.1016	0.0219	3.4206E-06	6,998	21.599	TRUE
ebi-PL179-GCST90277359	Phosphatidylinositol (16:0_18:2) levels	rs10466588	11	116,739,533	G	A	0.0958	0.1513	0.0285	1.1072E-07	6,998	28.233	TRUE
ebi-PL179-GCST90277359	Phosphatidylinositol (16:0_18:2) levels	rs57107736	16	76,627,797	T	C	0.1411	0.1152	0.0246	2.9404E-06	6,998	21.889	TRUE
ebi-PL179-GCST90277359	Phosphatidylinositol (16:0_18:2) levels	rs1748199	1	62,591,465	C	T	0.2621	-0.1135	0.0191	3.1957E-09	6,998	35.148	TRUE
ebi-PL179-GCST90277359	Phosphatidylinositol (16:0_18:2) levels	rs117113213	20	40,537,052	A	G	0.0714	0.1675	0.0344	1.1218E-06	6,998	23.747	TRUE
ebi-PL179-GCST90277359	Phosphatidylinositol (16:0_18:2) levels	rs73127058	5	77,835,346	A	G	0.0165	-0.3134	0.0683	4.4680E-06	6,998	21.083	TRUE
ebi-PL179-GCST90277360	Phosphatidylinositol (16:0_20:4) levels	rs4759322	12	54,071,880	G	A	0.3997	-0.0961	0.0194	7.7047E-07	5,519	24.485	TRUE
ebi-PL179-GCST90277360	Phosphatidylinositol (16:0_20:4) levels	rs145012974	11	127,595,711	A	G	0.0036	0.8599	0.1835	2.8297E-06	5,519	21.969	TRUE
ebi-PL179-GCST90277360	Phosphatidylinositol (16:0_20:4) levels	rs9653945	3	142,941,864	A	G	0.3641	-0.1108	0.0199	2.7165E-08	5,519	30.987	TRUE
ebi-PL179-GCST90277360	Phosphatidylinositol (16:0_20:4) levels	rs149878915	8	541,839	A	G	0.0400	0.2400	0.0483	6.9062E-07	5,519	24.694	TRUE
ebi-PL179-GCST90277360	Phosphatidylinositol (16:0_20:4) levels	rs578163523	9	126,297,765	T	G	0.0228	0.3034	0.0642	2.3111E-06	5,519	22.362	TRUE

Lipid GWAS ID	Lipid species	SNP	Chromosome	Position	Effect allele	Other allele	Effect-allele frequency	Beta	SE	P value	Sample size	F statistic	F > 10
ebi-PL179-GCST90277360	Phosphatidylinositol (16:0_20:4) levels	rs73197087	3	108,882,807	C	A	0.0935	-0.1620	0.0321	4.6804E-07	5,519	25.450	TRUE
ebi-PL179-GCST90277360	Phosphatidylinositol (16:0_20:4) levels	rs76609522	12	6,138,757	T	C	0.0232	0.3103	0.0660	2.6653E-06	5,519	22.088	TRUE
ebi-PL179-GCST90277360	Phosphatidylinositol (16:0_20:4) levels	rs12449710	17	52,850,260	C	A	0.5816	-0.0960	0.0194	7.4020E-07	5,519	24.561	TRUE
ebi-PL179-GCST90277360	Phosphatidylinositol (16:0_20:4) levels	rs144018078	6	116,375,468	G	C	0.0261	0.2866	0.0613	3.0328E-06	5,519	21.838	TRUE
ebi-PL179-GCST90277360	Phosphatidylinositol (16:0_20:4) levels	rs7782358	7	67,820	G	A	0.5926	-0.0979	0.0202	1.2533E-06	5,519	23.544	TRUE
ebi-PL179-GCST90277361	Phosphatidylinositol (18:0_18:1) levels	rs1340414	1	102,480,786	G	A	0.6458	0.0830	0.0175	2.2427E-06	7,162	22.410	TRUE
ebi-PL179-GCST90277361	Phosphatidylinositol (18:0_18:1) levels	rs12638256	3	142,936,448	G	A	0.6318	0.1603	0.0174	4.8848E-20	7,162	84.515	TRUE
ebi-PL179-GCST90277361	Phosphatidylinositol (18:0_18:1) levels	rs4804576	19	11,220,678	T	G	0.0624	-0.1672	0.0346	1.3984E-06	7,162	23.320	TRUE
ebi-PL179-GCST90277361	Phosphatidylinositol (18:0_18:1) levels	rs67835422	4	71,877,919	T	C	0.2769	-0.0883	0.0184	1.7211E-06	7,162	22.920	TRUE
ebi-PL179-GCST90277361	Phosphatidylinositol (18:0_18:1) levels	rs12122878	1	189,927,375	T	C	0.2894	-0.0856	0.0186	3.9794E-06	7,162	21.306	TRUE
ebi-PL179-GCST90277361	Phosphatidylinositol (18:0_18:1) levels	rs1560063	5	5,209,937	G	A	0.3282	0.0823	0.0179	4.5076E-06	7,162	21.067	TRUE
ebi-PL179-GCST90277361	Phosphatidylinositol (18:0_18:1) levels	rs7742350	6	14,803,818	T	C	0.0442	-0.1901	0.0415	4.8455E-06	7,162	20.927	TRUE
ebi-PL179-GCST90277361	Phosphatidylinositol (18:0_18:1) levels	rs11783515	8	58,407,830	G	A	0.6258	-0.0881	0.0172	3.0188E-07	7,162	26.285	TRUE
ebi-PL179-GCST90277361	Phosphatidylinositol (18:0_18:1) levels	rs7122803	11	107,883,610	G	C	0.8604	-0.1161	0.0239	1.1790E-06	7,162	23.651	TRUE
ebi-PL179-GCST90277362	Phosphatidylinositol (18:0_18:2) levels	rs2081687	8	58,476,006	C	T	0.6176	-0.0798	0.0170	2.6727E-06	7,170	22.073	TRUE
ebi-PL179-GCST90277362	Phosphatidylinositol (18:0_18:2) levels	rs114488287	8	84,010,633	A	T	0.0220	-0.2631	0.0571	4.1085E-06	7,170	21.245	TRUE
ebi-PL179-GCST90277362	Phosphatidylinositol (18:0_18:2) levels	rs41429348	15	73,927,659	T	C	0.0486	0.1828	0.0386	2.2922E-06	7,170	22.369	TRUE
ebi-PL179-GCST90277362	Phosphatidylinositol (18:0_18:2) levels	rs112713750	7	135,205,323	T	C	0.0205	-0.2672	0.0583	4.5750E-06	7,170	21.038	TRUE
ebi-PL179-GCST90277362	Phosphatidylinositol (18:0_18:2) levels	rs780093	2	27,519,736	C	T	0.6459	-0.0909	0.0173	1.5159E-07	7,170	27.620	TRUE
ebi-PL179-GCST90277362	Phosphatidylinositol (18:0_18:2) levels	rs7979473	12	120,982,457	G	A	0.5765	-0.1097	0.0168	7.3803E-11	7,170	42.541	TRUE
ebi-PL179-GCST90277362	Phosphatidylinositol (18:0_18:2) levels	rs72830461	6	10,040,346	C	T	0.0046	0.6376	0.1326	1.5577E-06	7,170	23.112	TRUE
ebi-PL179-GCST90277362	Phosphatidylinositol (18:0_18:2) levels	rs150684478	11	61,545,254	T	G	0.0240	0.3081	0.0572	7.4558E-08	7,170	29.001	TRUE
ebi-PL179-GCST90277362	Phosphatidylinositol (18:0_18:2) levels	rs7561644	2	78,784,774	T	G	0.4617	0.0862	0.0169	3.6264E-07	7,170	25.931	TRUE
ebi-PL179-GCST90277362	Phosphatidylinositol (18:0_18:2) levels	rs915894	6	32,222,613	G	T	0.3263	-0.0865	0.0177	1.0691E-06	7,170	23.839	TRUE
ebi-PL179-GCST90277362	Phosphatidylinositol (18:0_18:2) levels	rs77470505	11	61,458,997	G	T	0.0179	0.3112	0.0632	8.7009E-07	7,170	24.237	TRUE
ebi-PL179-GCST90277362	Phosphatidylinositol (18:0_18:2) levels	rs149615216	18	49,579,658	T	C	0.0049	0.6417	0.1155	2.8803E-08	7,170	30.853	TRUE
ebi-PL179-GCST90277362	Phosphatidylinositol (18:0_18:2) levels	rs6115094	20	25,230,191	G	A	0.5025	0.0791	0.0167	2.2644E-06	7,170	22.391	TRUE
ebi-PL179-GCST90277363	Phosphatidylinositol (18:0_20:3) levels	rs2819082	6	156,379,168	C	T	0.2946	-0.0881	0.0188	2.9708E-06	6,784	21.869	TRUE
ebi-PL179-GCST90277363	Phosphatidylinositol (18:0_20:3) levels	rs72914920	11	60,782,852	A	G	0.3060	-0.0957	0.0189	3.9776E-07	6,784	25.754	TRUE
ebi-PL179-GCST90277363	Phosphatidylinositol (18:0_20:3) levels	rs11769084	7	72,363,703	A	G	0.1048	0.1299	0.0281	3.9649E-06	6,784	21.315	TRUE
ebi-PL179-GCST90277363	Phosphatidylinositol (18:0_20:3) levels	rs1937711	10	51,855,519	G	C	0.6513	0.0908	0.0190	1.8398E-06	6,784	22.793	TRUE
ebi-PL179-GCST90277363	Phosphatidylinositol (18:0_20:3) levels	rs28514538	8	58,485,873	G	A	0.2459	-0.0925	0.0200	3.6746E-06	6,784	21.463	TRUE
ebi-PL179-GCST90277363	Phosphatidylinositol (18:0_20:3) levels	rs28456	11	61,822,009	G	A	0.3835	0.3493	0.0173	1.7016E-88	6,784	409.645	TRUE
ebi-PL179-GCST90277363	Phosphatidylinositol (18:0_20:3) levels	rs117511121	19	18,072,285	T	C	0.0124	-0.3654	0.0772	2.2793E-06	6,784	22.381	TRUE
ebi-PL179-GCST90277363	Phosphatidylinositol (18:0_20:3) levels	rs7845664	8	40,712,078	G	A	0.5760	-0.0822	0.0174	2.2609E-06	6,784	22.395	TRUE
ebi-PL179-GCST90277363	Phosphatidylinositol (18:0_20:3) levels	rs10897211	11	62,056,104	A	G	0.3351	0.0994	0.0183	5.8360E-08	6,784	29.482	TRUE
ebi-PL179-GCST90277363	Phosphatidylinositol (18:0_20:3) levels	rs12598816	16	80,253,481	T	G	0.2680	-0.0905	0.0194	3.1192E-06	6,784	21.778	TRUE
ebi-PL179-GCST90277363	Phosphatidylinositol (18:0_20:3) levels	rs67945876	4	78,054,777	C	T	0.2342	-0.0953	0.0205	3.2849E-06	6,784	21.678	TRUE
ebi-PL179-GCST90277363	Phosphatidylinositol (18:0_20:3) levels	rs6820980	4	4,988,836	A	G	0.5719	-0.0846	0.0178	1.9820E-06	6,784	22.649	TRUE
ebi-PL179-GCST90277363	Phosphatidylinositol (18:0_20:3) levels	rs76457276	4	37,896,323	G	A	0.0258	-0.2575	0.0559	4.2076E-06	6,784	21.201	TRUE
ebi-PL179-GCST90277363	Phosphatidylinositol (18:0_20:3) levels	rs12458441	18	49,595,788	C	A	0.0934	0.1467	0.0298	8.7395E-07	6,784	24.230	TRUE
ebi-PL179-GCST90277363	Phosphatidylinositol (18:0_20:3) levels	rs13147779	4	169,287,018	C	T	0.1894	-0.1044	0.0220	2.0262E-06	6,784	22.608	TRUE
ebi-PL179-GCST90277364	Phosphatidylinositol (18:0_20:4) levels	rs9848779	3	142,933,227	G	C	0.6306	-0.0941	0.0174	6.8956E-08	7,174	29.153	TRUE
ebi-PL179-GCST90277364	Phosphatidylinositol (18:0_20:4) levels	rs11899922	2	137,161,796	G	A	0.0598	0.1626	0.0350	3.4637E-06	7,174	21.574	TRUE
ebi-PL179-GCST90277364	Phosphatidylinositol (18:0_20:4) levels	rs13068826	3	171,396,003	A	G	0.5868	-0.0796	0.0167	2.0210E-06	7,174	22.613	TRUE
ebi-PL179-GCST90277364	Phosphatidylinositol (18:0_20:4) levels	rs4629190	2	68,915,026	C	T	0.4870	0.0825	0.0166	6.2953E-07	7,174	24.864	TRUE
ebi-PL179-GCST90277364	Phosphatidylinositol (18:0_20:4) levels	rs61872308	10	112,296,190	C	G	0.0855	-0.1444	0.0307	2.6522E-06	7,174	22.087	TRUE
ebi-PL179-GCST90277364	Phosphatidylinositol (18:0_20:4) levels	rs10138449	14	61,547,116	C	G	0.1325	0.1172	0.0247	2.0474E-06	7,174	22.583	TRUE
ebi-PL179-GCST90277364	Phosphatidylinositol (18:0_20:4) levels	rs11780610	8	18,402,366	T	C	0.6800	-0.0922	0.0178	2.2487E-07	7,174	26.857	TRUE
ebi-PL179-GCST90277364	Phosphatidylinositol (18:0_20:4) levels	rs2725021	8	4,623,841	G	C	0.5073	0.0844	0.0172	9.1227E-07	7,174	24.146	TRUE
ebi-PL179-GCST90277364	Phosphatidylinositol (18:0_20:4) levels	rs4912056	1	18,918,184	T	C	0.1520	0.1137	0.0232	1.0240E-06	7,174	23.924	TRUE
ebi-PL179-GCST90277364	Phosphatidylinositol (18:0_20:4) levels	rs112944184	1	239,312,130	T	C	0.0174	0.3027	0.0635	1.9176E-06	7,174	22.713	TRUE
ebi-PL179-GCST90277364	Phosphatidylinositol (18:0_20:4) levels	rs3004050	6	26,740,240	A	G	0.3557	0.0817	0.0175	3.2747E-06	7,174	21.682	TRUE
ebi-PL179-GCST90277364	Phosphatidylinositol (18:0_20:4) levels	rs144215405	19	53,935,764	T	C	0.0186	-0.3078	0.0656	2.7166E-06	7,174	22.043	TRUE
ebi-PL179-GCST90277364	Phosphatidylinositol (18:0_20:4) levels	rs10423460	19	31,666,796	G	A	0.8738	-0.1204	0.0258	3.2201E-06	7,174	21.714	TRUE
ebi-PL179-GCST90277364	Phosphatidylinositol (18:0_20:4) levels	rs6507942	18	49,666,690	A	T	0.4988	0.0788	0.0165	1.9751E-06	7,174	22.656	TRUE
ebi-PL179-GCST90277364	Phosphatidylinositol (18:0_20:4) levels	rs17699030	19	11,220,266	G	A	0.0469	-0.2308	0.0393	4.4845E-09	7,174	34.482	TRUE
ebi-PL179-GCST90277364	Phosphatidylinositol (18:0_20:4) levels	rs2425225	20	36,376,425	C	T	0.6297	0.0781	0.0171	4.8693E-06	7,174	20.920	TRUE

Lipid GWAS ID	Lipid species	SNP	Chromosome	Position	Effect allele	Other allele	Effect-allele frequency	Beta	SE	P value	Sample size	F statistic	F > 10
ebi-PL179-GCST90277364	Phosphatidylinositol (18:0_20:4) levels	rs56339947	18	49,628,409	C	T	0.1144	0.1882	0.0265	1.3590E-12	7,174	50.417	TRUE
ebi-PL179-GCST90277365	Phosphatidylinositol (18:1_18:1) levels	rs186422572	14	103,616,633	A	G	0.0132	0.3473	0.0753	4.0828E-06	7,164	21.256	TRUE
ebi-PL179-GCST90277365	Phosphatidylinositol (18:1_18:1) levels	rs62409317	4	25,950,031	A	G	0.0462	0.1946	0.0420	3.6466E-06	7,164	21.475	TRUE
ebi-PL179-GCST90277365	Phosphatidylinositol (18:1_18:1) levels	rs12428337	13	22,601,154	A	G	0.2381	0.0894	0.0195	4.5639E-06	7,164	21.043	TRUE
ebi-PL179-GCST90277365	Phosphatidylinositol (18:1_18:1) levels	rs116845732	14	38,415,942	C	T	0.0036	0.7148	0.1391	2.8607E-07	7,164	26.391	TRUE
ebi-PL179-GCST90277365	Phosphatidylinositol (18:1_18:1) levels	rs9652580	16	24,297,504	T	C	0.4234	-0.0805	0.0173	3.2982E-06	7,164	21.667	TRUE
ebi-PL179-GCST90277365	Phosphatidylinositol (18:1_18:1) levels	rs36634	19	54,142,105	A	G	0.5929	-0.1218	0.0169	6.2110E-13	7,164	51.966	TRUE
ebi-PL179-GCST90277365	Phosphatidylinositol (18:1_18:1) levels	rs72846591	2	141,712,670	C	T	0.3976	0.0800	0.0171	3.0028E-06	7,164	21.847	TRUE
ebi-PL179-GCST90277365	Phosphatidylinositol (18:1_18:1) levels	rs41418546	21	36,261,009	G	T	0.0762	0.1482	0.0324	4.7455E-06	7,164	20.968	TRUE
ebi-PL179-GCST90277366	Phosphatidylinositol (18:1_18:2) levels	rs11803708	1	236,637,819	A	G	0.0486	0.1971	0.0411	1.6862E-06	6,489	22.965	TRUE
ebi-PL179-GCST90277366	Phosphatidylinositol (18:1_18:2) levels	rs73109128	3	74,310,731	G	C	0.0278	0.2589	0.0546	2.2039E-06	6,489	22.448	TRUE
ebi-PL179-GCST90277366	Phosphatidylinositol (18:1_18:2) levels	rs17074477	5	172,222,516	G	A	0.1086	-0.1396	0.0280	6.5141E-07	6,489	24.801	TRUE
ebi-PL179-GCST90277366	Phosphatidylinositol (18:1_18:2) levels	rs144480172	12	32,548,599	T	C	0.0251	0.2791	0.0565	8.0727E-07	6,489	24.387	TRUE
ebi-PL179-GCST90277366	Phosphatidylinositol (18:1_18:2) levels	rs72753955	15	82,771,066	G	A	0.0107	-0.4169	0.0878	2.0854E-06	6,489	22.553	TRUE
ebi-PL179-GCST90277366	Phosphatidylinositol (18:1_18:2) levels	rs1544531	16	5,592,867	A	C	0.5165	0.0824	0.0174	2.3695E-06	6,489	22.309	TRUE
ebi-PL179-GCST90277366	Phosphatidylinositol (18:1_18:2) levels	rs10911365	1	183,589,970	A	G	0.0896	0.1444	0.0310	3.2887E-06	6,489	21.674	TRUE
ebi-PL179-GCST90277366	Phosphatidylinositol (18:1_18:2) levels	rs4848460	2	117,641,775	T	C	0.0502	0.1868	0.0405	3.9431E-06	6,489	21.327	TRUE
ebi-PL179-GCST90277366	Phosphatidylinositol (18:1_18:2) levels	rs79507869	2	42,173,936	A	C	0.1290	-0.1253	0.0262	1.7949E-06	6,489	22.843	TRUE
ebi-PL179-GCST90277366	Phosphatidylinositol (18:1_18:2) levels	rs72884421	18	13,582,870	C	G	0.0665	-0.1683	0.0357	2.3967E-06	6,489	22.286	TRUE
ebi-PL179-GCST90277366	Phosphatidylinositol (18:1_18:2) levels	rs365653	19	44,858,389	G	A	0.0908	-0.1406	0.0307	4.7975E-06	6,489	20.951	TRUE
ebi-PL179-GCST90277366	Phosphatidylinositol (18:1_18:2) levels	rs9953437	18	49,594,230	A	G	0.0962	0.1544	0.0301	2.9087E-07	6,489	26.365	TRUE
ebi-PL179-GCST90277367	Phosphatidylinositol (18:1_20:4) levels	rs2819083	6	156,379,682	A	G	0.4862	-0.0924	0.0184	4.9156E-07	5,882	25.355	TRUE
ebi-PL179-GCST90277367	Phosphatidylinositol (18:1_20:4) levels	rs117598891	14	95,031,978	C	T	0.0081	-0.4818	0.1053	4.9060E-06	5,882	20.914	TRUE
ebi-PL179-GCST90277367	Phosphatidylinositol (18:1_20:4) levels	rs670975	11	76,222,144	T	A	0.0669	0.1732	0.0376	4.2024E-06	5,882	21.209	TRUE
ebi-PL179-GCST90277367	Phosphatidylinositol (18:1_20:4) levels	rs4920209	1	234,028,770	A	G	0.4746	0.0877	0.0183	1.6290E-06	5,882	23.033	TRUE
ebi-PL179-GCST90277367	Phosphatidylinositol (18:1_20:4) levels	rs55976852	6	94,237,667	C	A	0.2735	-0.0988	0.0207	1.9203E-06	5,882	22.716	TRUE
ebi-PL179-GCST90277367	Phosphatidylinositol (18:1_20:4) levels	rs3741298	11	116,786,845	T	C	0.7654	-0.1268	0.0216	4.3485E-09	5,882	34.562	TRUE
ebi-PL179-GCST90277367	Phosphatidylinositol (18:1_20:4) levels	rs36061340	8	100,795,002	T	C	0.0521	0.1937	0.0414	2.9374E-06	5,882	21.898	TRUE
ebi-PL179-GCST90277367	Phosphatidylinositol (18:1_20:4) levels	rs75166839	18	73,825,578	G	A	0.0219	0.3008	0.0649	3.7040E-06	5,882	21.452	TRUE
ebi-PL179-GCST90277367	Phosphatidylinositol (18:1_20:4) levels	rs140854080	4	87,407,252	A	G	0.0140	0.3701	0.0785	2.4684E-06	5,882	22.233	TRUE
ebi-PL179-GCST90277367	Phosphatidylinositol (18:1_20:4) levels	rs61915733	12	18,171,023	A	G	0.0897	0.1522	0.0320	2.0889E-06	5,882	22.558	TRUE
ebi-PL179-GCST90277367	Phosphatidylinositol (18:1_20:4) levels	rs117210444	16	73,859,072	G	A	0.0345	-0.2868	0.0498	9.1872E-09	5,882	33.097	TRUE
ebi-PL179-GCST90277367	Phosphatidylinositol (18:1_20:4) levels	rs957142	18	49,619,347	A	G	0.0937	0.1517	0.0320	2.2381E-06	5,882	22.423	TRUE
ebi-PL179-GCST90277368	Sphingomyelin (d32:1) levels	rs6768493	3	4,819,419	C	G	0.5251	0.0785	0.0166	2.2736E-06	7,174	22.384	TRUE
ebi-PL179-GCST90277368	Sphingomyelin (d32:1) levels	rs75679663	17	4,764,677	A	C	0.0258	-0.2561	0.0537	1.8601E-06	7,174	22.770	TRUE
ebi-PL179-GCST90277368	Sphingomyelin (d32:1) levels	rs2604631	4	60,937,521	A	G	0.7536	-0.0896	0.0194	4.0252E-06	7,174	21.284	TRUE
ebi-PL179-GCST90277368	Sphingomyelin (d32:1) levels	rs2553631	2	124,364,198	T	A	0.0699	-0.1507	0.0327	4.2369E-06	7,174	21.185	TRUE
ebi-PL179-GCST90277368	Sphingomyelin (d32:1) levels	rs12192157	6	169,320,808	T	C	0.4652	0.0789	0.0168	2.7840E-06	7,174	21.994	TRUE
ebi-PL179-GCST90277368	Sphingomyelin (d32:1) levels	rs10975312	9	5,837,780	A	G	0.0312	0.2459	0.0487	4.5462E-07	7,174	25.493	TRUE
ebi-PL179-GCST90277368	Sphingomyelin (d32:1) levels	rs138440819	20	33,730,205	A	G	0.0149	-0.3284	0.0701	2.8886E-06	7,174	21.924	TRUE
ebi-PL179-GCST90277368	Sphingomyelin (d32:1) levels	rs58001441	9	6,764,828	A	G	0.1651	-0.1036	0.0225	4.2093E-06	7,174	21.199	TRUE
ebi-PL179-GCST90277368	Sphingomyelin (d32:1) levels	rs217184	16	72,072,066	C	T	0.2280	-0.0955	0.0198	1.4899E-06	7,174	23.199	TRUE
ebi-PL179-GCST90277368	Sphingomyelin (d32:1) levels	rs3741111	11	124,076,588	G	A	0.4828	0.0816	0.0167	1.0188E-06	7,174	23.932	TRUE
ebi-PL179-GCST90277368	Sphingomyelin (d32:1) levels	rs6444176	3	186,868,271	T	C	0.0520	0.1804	0.0375	1.5893E-06	7,174	23.074	TRUE
ebi-PL179-GCST90277368	Sphingomyelin (d32:1) levels	rs75418091	4	4,501,634	T	C	0.0119	0.4058	0.0792	3.0258E-07	7,174	26.280	TRUE
ebi-PL179-GCST90277368	Sphingomyelin (d32:1) levels	rs72649550	15	93,611,333	C	G	0.3322	0.0815	0.0178	4.7340E-06	7,174	20.972	TRUE
ebi-PL179-GCST90277368	Sphingomyelin (d32:1) levels	rs8017356	14	65,115,063	G	A	0.9863	-0.3477	0.0717	1.2866E-06	7,174	23.482	TRUE
ebi-PL179-GCST90277368	Sphingomyelin (d32:1) levels	rs117510063	16	55,539,036	G	A	0.0045	-0.5841	0.1274	4.5864E-06	7,174	21.033	TRUE
ebi-PL179-GCST90277368	Sphingomyelin (d32:1) levels	rs7247433	19	19,684,751	A	C	0.3944	-0.0800	0.0174	4.2066E-06	7,174	21.197	TRUE
ebi-PL179-GCST90277368	Sphingomyelin (d32:1) levels	rs3026120	17	5,384,095	A	G	0.0746	-0.1748	0.0312	2.1412E-08	7,174	31.431	TRUE
ebi-PL179-GCST90277369	Sphingomyelin (d34:0) levels	rs11631073	15	57,322,347	T	C	0.6184	-0.0912	0.0187	1.1084E-06	6,207	23.775	TRUE
ebi-PL179-GCST90277369	Sphingomyelin (d34:0) levels	rs112585713	4	72,876,701	A	G	0.0316	0.2615	0.0510	2.9623E-07	6,207	26.329	TRUE
ebi-PL179-GCST90277369	Sphingomyelin (d34:0) levels	rs55811539	3	113,313,552	C	T	0.0124	-0.3992	0.0836	1.8338E-06	6,207	22.803	TRUE
ebi-PL179-GCST90277369	Sphingomyelin (d34:0) levels	rs8071514	17	80,990,283	A	C	0.5685	0.0848	0.0181	2.8037E-06	6,207	21.984	TRUE
ebi-PL179-GCST90277369	Sphingomyelin (d34:0) levels	rs6683774	1	198,625,465	T	C	0.9338	0.1777	0.0365	1.1696E-06	6,207	23.673	TRUE
ebi-PL179-GCST90277369	Sphingomyelin (d34:0) levels	rs17497891	1	222,184,468	G	A	0.1504	0.1187	0.0252	2.5098E-06	6,207	22.197	TRUE
ebi-PL179-GCST90277369	Sphingomyelin (d34:0) levels	rs934198	2	21,055,901	T	G	0.2970	0.0978	0.0198	8.3951E-07	6,207	24.313	TRUE
ebi-PL179-GCST90277369	Sphingomyelin (d34:0) levels	rs80130206	10	106,229,406	T	C	0.0550	-0.1825	0.0390	2.9930E-06	6,207	21.859	TRUE

Lipid GWAS ID	Lipid species	SNP	Chromosome	Position	Effect allele	Other allele	Effect-allele frequency	Beta	SE	P value	Sample size	F statistic	F > 10
ebi-PL179-GCST90277369	Sphingomyelin (d34:0) levels	rs595587	21	43,371,358	C	T	0.3403	0.0900	0.0191	2.5575E-06	6,207	22.163	TRUE
ebi-PL179-GCST90277369	Sphingomyelin (d34:0) levels	rs2808569	9	98,537,277	C	A	0.2420	-0.0979	0.0211	3.5094E-06	6,207	21.552	TRUE
ebi-PL179-GCST90277369	Sphingomyelin (d34:0) levels	rs12880341	14	63,769,473	C	T	0.1252	-0.3274	0.0271	3.9402E-33	6,207	145.474	TRUE
ebi-PL179-GCST90277369	Sphingomyelin (d34:0) levels	rs55985238	17	3,769,209	A	T	0.0088	-0.5599	0.1006	2.7223E-08	6,207	30.974	TRUE
ebi-PL179-GCST90277370	Sphingomyelin (d34:1) levels	rs34087767	2	181,902,451	G	C	0.0054	0.5976	0.1250	1.7822E-06	7,174	22.855	TRUE
ebi-PL179-GCST90277370	Sphingomyelin (d34:1) levels	rs72794954	16	76,342,662	T	C	0.0072	-0.4993	0.1048	1.9258E-06	7,174	22.703	TRUE
ebi-PL179-GCST90277370	Sphingomyelin (d34:1) levels	rs117279275	12	52,892,255	A	G	0.0144	-0.3338	0.0694	1.5193E-06	7,174	23.166	TRUE
ebi-PL179-GCST90277370	Sphingomyelin (d34:1) levels	rs35577358	4	162,543,049	G	A	0.1354	0.1262	0.0246	2.8417E-07	7,174	26.401	TRUE
ebi-PL179-GCST90277370	Sphingomyelin (d34:1) levels	rs2306619	5	126,552,164	G	A	0.1828	-0.1014	0.0214	2.1448E-06	7,174	22.499	TRUE
ebi-PL179-GCST90277370	Sphingomyelin (d34:1) levels	rs56108088	14	86,739,725	C	T	0.5629	0.0771	0.0167	4.1084E-06	7,174	21.246	TRUE
ebi-PL179-GCST90277370	Sphingomyelin (d34:1) levels	rs116302332	4	74,505,174	T	C	0.0281	0.3765	0.0507	1.2770E-13	7,174	55.098	TRUE
ebi-PL179-GCST90277370	Sphingomyelin (d34:1) levels	rs722814	14	69,889,451	A	C	0.0789	0.1423	0.0311	4.7353E-06	7,174	20.975	TRUE
ebi-PL179-GCST90277370	Sphingomyelin (d34:1) levels	rs141286087	16	51,029,992	G	A	0.0408	0.2186	0.0452	1.3149E-06	7,174	23.439	TRUE
ebi-PL179-GCST90277370	Sphingomyelin (d34:1) levels	rs7575840	2	21,050,618	T	G	0.2959	0.1005	0.0185	5.4970E-08	7,174	29.597	TRUE
ebi-PL179-GCST90277370	Sphingomyelin (d34:1) levels	rs7032795	9	114,372,665	C	T	0.4807	0.0842	0.0167	4.4703E-07	7,174	25.522	TRUE
ebi-PL179-GCST90277370	Sphingomyelin (d34:1) levels	rs114483871	4	73,124,451	T	C	0.0381	0.2821	0.0448	3.2399E-10	7,174	39.636	TRUE
ebi-PL179-GCST90277370	Sphingomyelin (d34:1) levels	rs7012814	8	9,315,848	A	G	0.5118	0.0799	0.0168	2.0906E-06	7,174	22.545	TRUE
ebi-PL179-GCST90277370	Sphingomyelin (d34:1) levels	rs7909144	10	52,240,944	G	A	0.5931	0.0788	0.0169	3.2530E-06	7,174	21.693	TRUE
ebi-PL179-GCST90277370	Sphingomyelin (d34:1) levels	rs78143047	9	5,527,351	G	A	0.0149	0.3427	0.0705	1.1897E-06	7,174	23.633	TRUE
ebi-PL179-GCST90277370	Sphingomyelin (d34:1) levels	rs1934410	1	180,552,628	T	G	0.6269	0.0816	0.0174	2.8557E-06	7,174	21.944	TRUE
ebi-PL179-GCST90277370	Sphingomyelin (d34:1) levels	rs74915447	4	72,899,760	A	G	0.0378	0.2882	0.0442	7.4975E-11	7,174	42.513	TRUE
ebi-PL179-GCST90277370	Sphingomyelin (d34:1) levels	rs191640693	13	47,201,342	T	C	0.0272	-0.2441	0.0530	4.1301E-06	7,174	21.235	TRUE
ebi-PL179-GCST90277370	Sphingomyelin (d34:1) levels	rs2836524	21	38,573,905	A	G	0.0597	0.1674	0.0358	2.9041E-06	7,174	21.909	TRUE
ebi-PL179-GCST90277371	Sphingomyelin (d34:2) levels	rs111507730	13	33,753,876	C	T	0.0537	0.1708	0.0367	3.3290E-06	7,174	21.645	TRUE
ebi-PL179-GCST90277371	Sphingomyelin (d34:2) levels	rs939971	15	80,029,927	A	C	0.2111	-0.0962	0.0204	2.4291E-06	7,174	22.256	TRUE
ebi-PL179-GCST90277371	Sphingomyelin (d34:2) levels	rs113127305	10	70,837,164	A	G	0.0498	-0.2171	0.0383	1.4591E-08	7,174	32.179	TRUE
ebi-PL179-GCST90277371	Sphingomyelin (d34:2) levels	rs117009181	12	124,846,478	C	T	0.1499	0.1100	0.0231	2.0318E-06	7,174	22.599	TRUE
ebi-PL179-GCST90277371	Sphingomyelin (d34:2) levels	rs62412280	6	67,724,705	T	A	0.0039	-0.6559	0.1403	2.9731E-06	7,174	21.867	TRUE
ebi-PL179-GCST90277371	Sphingomyelin (d34:2) levels	rs1033030	14	99,309,314	A	G	0.1764	-0.1053	0.0222	2.1576E-06	7,174	22.484	TRUE
ebi-PL179-GCST90277371	Sphingomyelin (d34:2) levels	rs174561	11	61,815,236	C	T	0.3813	-0.1696	0.0172	7.2617E-23	7,174	97.587	TRUE
ebi-PL179-GCST90277371	Sphingomyelin (d34:2) levels	rs113722226	19	11,078,596	C	T	0.0943	-0.1863	0.0281	3.6977E-11	7,174	43.900	TRUE
ebi-PL179-GCST90277371	Sphingomyelin (d34:2) levels	rs17276940	14	20,017,956	T	C	0.0335	-0.2136	0.0460	3.4777E-06	7,174	21.564	TRUE
ebi-PL179-GCST90277371	Sphingomyelin (d34:2) levels	rs12952341	17	4,482,208	T	C	0.3263	0.0853	0.0186	4.8366E-06	7,174	20.931	TRUE
ebi-PL179-GCST90277372	Sphingomyelin (d36:1) levels	rs12464353	2	60,982,893	A	G	0.0326	0.2293	0.0479	1.7415E-06	7,174	22.898	TRUE
ebi-PL179-GCST90277372	Sphingomyelin (d36:1) levels	rs6829637	4	110,405,753	G	A	0.4158	-0.0787	0.0172	4.7721E-06	7,174	20.956	TRUE
ebi-PL179-GCST90277372	Sphingomyelin (d36:1) levels	rs117643293	17	5,065,669	T	C	0.0620	-0.1707	0.0341	5.5049E-07	7,174	25.122	TRUE
ebi-PL179-GCST90277372	Sphingomyelin (d36:1) levels	rs186039163	17	3,547,599	G	T	0.0101	-0.4156	0.0840	7.7362E-07	7,174	24.464	TRUE
ebi-PL179-GCST90277372	Sphingomyelin (d36:1) levels	rs2336171	19	8,209,156	C	G	0.3987	0.1953	0.0170	2.9396E-30	7,174	131.860	TRUE
ebi-PL179-GCST90277372	Sphingomyelin (d36:1) levels	rs1452773	2	50,050,811	G	T	0.1382	-0.1170	0.0240	1.1003E-06	7,174	23.784	TRUE
ebi-PL179-GCST90277372	Sphingomyelin (d36:1) levels	rs7248003	19	8,236,164	T	C	0.7209	0.1453	0.0190	2.6885E-14	7,174	58.186	TRUE
ebi-PL179-GCST90277372	Sphingomyelin (d36:1) levels	rs4809845	20	51,460,945	C	T	0.1337	0.1292	0.0250	2.4126E-07	7,174	26.720	TRUE
ebi-PL179-GCST90277372	Sphingomyelin (d36:1) levels	rs6558122	8	29,518,250	C	T	0.1007	-0.1397	0.0277	4.5833E-07	7,174	25.477	TRUE
ebi-PL179-GCST90277372	Sphingomyelin (d36:1) levels	rs114839471	5	90,249,218	G	A	0.0082	-0.4451	0.0946	2.5866E-06	7,174	22.135	TRUE
ebi-PL179-GCST90277372	Sphingomyelin (d36:1) levels	rs79146711	4	73,424,911	T	C	0.0451	0.2344	0.0409	1.0185E-08	7,174	32.880	TRUE
ebi-PL179-GCST90277372	Sphingomyelin (d36:1) levels	rs886813	2	241,457,278	T	G	0.4684	0.0811	0.0169	1.6140E-06	7,174	23.044	TRUE
ebi-PL179-GCST90277372	Sphingomyelin (d36:1) levels	rs12462619	19	8,607,097	C	T	0.2613	-0.1079	0.0193	2.3244E-08	7,174	31.274	TRUE
ebi-PL179-GCST90277372	Sphingomyelin (d36:1) levels	rs12675806	8	4,789,612	A	G	0.1116	0.1259	0.0264	1.9046E-06	7,174	22.725	TRUE
ebi-PL179-GCST90277373	Sphingomyelin (d36:2) levels	rs111906544	16	7,818,592	C	G	0.0222	-0.2689	0.0580	3.5937E-06	7,173	21.501	TRUE
ebi-PL179-GCST90277373	Sphingomyelin (d36:2) levels	rs7700432	5	130,859,001	A	G	0.3538	-0.0795	0.0173	4.5514E-06	7,173	21.048	TRUE
ebi-PL179-GCST90277373	Sphingomyelin (d36:2) levels	rs117546336	6	98,098,087	G	T	0.0473	-0.1897	0.0399	2.0030E-06	7,173	22.629	TRUE
ebi-PL179-GCST90277373	Sphingomyelin (d36:2) levels	rs9738226	12	120,985,856	G	A	0.5787	0.1008	0.0169	2.3701E-09	7,173	35.732	TRUE
ebi-PL179-GCST90277373	Sphingomyelin (d36:2) levels	rs7253584	19	8,222,034	C	T	0.6752	0.1809	0.0177	2.4758E-24	7,173	104.348	TRUE
ebi-PL179-GCST90277373	Sphingomyelin (d36:2) levels	rs78999781	2	203,425,314	C	T	0.0910	-0.1476	0.0291	3.9549E-07	7,173	25.763	TRUE
ebi-PL179-GCST90277373	Sphingomyelin (d36:2) levels	rs72879665	18	13,203,131	A	G	0.0105	0.3819	0.0834	4.7277E-06	7,173	20.975	TRUE
ebi-PL179-GCST90277373	Sphingomyelin (d36:2) levels	rs7247304	19	8,602,475	T	C	0.2673	-0.1032	0.0193	9.0742E-08	7,173	28.621	TRUE
ebi-PL179-GCST90277373	Sphingomyelin (d36:2) levels	rs148860058	22	48,612,019	A	G	0.0106	0.4008	0.0872	4.3667E-06	7,173	21.132	TRUE
ebi-PL179-GCST90277374	Sphingomyelin (d38:1) levels	rs116773966	2	145,963,613	A	G	0.0175	0.3006	0.0638	2.5170E-06	7,174	22.188	TRUE
ebi-PL179-GCST90277374	Sphingomyelin (d38:1) levels	rs7970512	12	67,513,820	A	C	0.0350	-0.2253	0.0478	2.4643E-06	7,174	22.227	TRUE

Lipid GWAS ID	Lipid species	SNP	Chromosome	Position	Effect allele	Other allele	Effect-allele frequency	Beta	SE	P value	Sample size	F statistic	F > 10
ebi-PL179-GCST90277374	Sphingomyelin (d38:1) levels	rs77645768	4	72,803,111	A	G	0.0256	0.3038	0.0536	1.5252E-08	7,174	32.088	TRUE
ebi-PL179-GCST90277374	Sphingomyelin (d38:1) levels	rs117146578	12	101,922,985	C	T	0.0482	0.1825	0.0392	3.2720E-06	7,174	21.683	TRUE
ebi-PL179-GCST90277374	Sphingomyelin (d38:1) levels	rs76174849	1	8,738,706	T	C	0.0140	0.3342	0.0719	3.4377E-06	7,174	21.588	TRUE
ebi-PL179-GCST90277374	Sphingomyelin (d38:1) levels	rs144440646	18	71,848,979	C	T	0.0113	-0.3939	0.0819	1.5401E-06	7,174	23.137	TRUE
ebi-PL179-GCST90277374	Sphingomyelin (d38:1) levels	rs73073606	3	194,176,577	C	A	0.2406	-0.0965	0.0201	1.6831E-06	7,174	22.964	TRUE
ebi-PL179-GCST90277374	Sphingomyelin (d38:1) levels	rs7478167	10	101,716,501	A	T	0.6694	-0.0830	0.0178	3.3597E-06	7,174	21.633	TRUE
ebi-PL179-GCST90277374	Sphingomyelin (d38:1) levels	rs374071816	6	160,682,773	A	T	0.0574	0.1901	0.0363	1.6222E-07	7,174	27.487	TRUE
ebi-PL179-GCST90277374	Sphingomyelin (d38:1) levels	rs13258507	8	125,480,355	G	T	0.2406	0.0926	0.0194	1.9235E-06	7,174	22.705	TRUE
ebi-PL179-GCST90277374	Sphingomyelin (d38:1) levels	rs147426822	8	23,959,870	T	C	0.0132	0.3590	0.0786	4.9736E-06	7,174	20.878	TRUE
ebi-PL179-GCST90277374	Sphingomyelin (d38:1) levels	rs715119	9	104,975,112	T	C	0.2325	-0.0919	0.0197	3.1119E-06	7,174	21.782	TRUE
ebi-PL179-GCST90277374	Sphingomyelin (d38:1) levels	rs7258249	19	8,206,837	A	G	0.3987	0.2168	0.0169	2.8079E-37	7,174	164.640	TRUE
ebi-PL179-GCST90277374	Sphingomyelin (d38:1) levels	rs76596580	21	34,990,612	T	C	0.1151	0.1212	0.0264	4.5815E-06	7,174	21.037	TRUE
ebi-PL179-GCST90277375	Sphingomyelin (d38:2) levels	rs72839716	10	122,833,455	A	G	0.1587	-0.1077	0.0229	2.5350E-06	7,150	22.176	TRUE
ebi-PL179-GCST90277375	Sphingomyelin (d38:2) levels	rs75098294	13	62,988,645	G	A	0.0062	0.5266	0.1071	9.0591E-07	7,150	24.160	TRUE
ebi-PL179-GCST90277375	Sphingomyelin (d38:2) levels	rs672959	10	14,418,295	G	A	0.0079	-0.4590	0.1002	4.6735E-06	7,150	20.997	TRUE
ebi-PL179-GCST90277375	Sphingomyelin (d38:2) levels	rs114717988	6	48,788,044	A	T	0.0457	0.1975	0.0396	6.3673E-07	7,150	24.841	TRUE
ebi-PL179-GCST90277375	Sphingomyelin (d38:2) levels	rs10405061	19	8,341,496	T	C	0.3087	-0.0946	0.0182	2.0116E-07	7,150	27.072	TRUE
ebi-PL179-GCST90277375	Sphingomyelin (d38:2) levels	rs113118892	15	59,274,186	T	C	0.0123	0.3829	0.0797	1.5765E-06	7,150	23.088	TRUE
ebi-PL179-GCST90277375	Sphingomyelin (d38:2) levels	rs10206976	2	210,749,914	T	G	0.2681	0.0905	0.0190	1.8794E-06	7,150	22.750	TRUE
ebi-PL179-GCST90277375	Sphingomyelin (d38:2) levels	rs3936109	3	184,623,650	G	A	0.1797	-0.1036	0.0221	2.7213E-06	7,150	22.039	TRUE
ebi-PL179-GCST90277375	Sphingomyelin (d38:2) levels	rs250507	19	8,268,177	C	T	0.2288	-0.1087	0.0202	7.4880E-08	7,150	28.992	TRUE
ebi-PL179-GCST90277375	Sphingomyelin (d38:2) levels	rs139150589	10	70,459,848	T	C	0.0077	0.5214	0.1000	1.8741E-07	7,150	27.210	TRUE
ebi-PL179-GCST90277375	Sphingomyelin (d38:2) levels	rs148683988	22	48,622,225	G	C	0.0144	0.3390	0.0735	4.0438E-06	7,150	21.277	TRUE
ebi-PL179-GCST90277376	Sphingomyelin (d40:1) levels	rs2552134	8	4,075,730	T	G	0.2207	-0.0928	0.0201	3.8761E-06	7,174	21.358	TRUE
ebi-PL179-GCST90277376	Sphingomyelin (d40:1) levels	rs113318071	11	46,848,527	T	C	0.0632	0.1617	0.0342	2.3272E-06	7,174	22.339	TRUE
ebi-PL179-GCST90277376	Sphingomyelin (d40:1) levels	rs9835223	3	186,868,495	G	C	0.8012	-0.1036	0.0211	9.2935E-07	7,174	24.110	TRUE
ebi-PL179-GCST90277376	Sphingomyelin (d40:1) levels	rs283819	2	79,000,117	C	A	0.8155	-0.1059	0.0216	1.0085E-06	7,174	23.954	TRUE
ebi-PL179-GCST90277376	Sphingomyelin (d40:1) levels	rs2275544	9	104,888,931	C	T	0.1251	-0.1227	0.0252	1.1344E-06	7,174	23.724	TRUE
ebi-PL179-GCST90277376	Sphingomyelin (d40:1) levels	rs8067235	17	81,050,837	A	G	0.3962	0.0788	0.0171	4.0974E-06	7,174	21.251	TRUE
ebi-PL179-GCST90277376	Sphingomyelin (d40:1) levels	rs77030061	4	168,898,979	T	C	0.0376	0.2248	0.0479	2.6765E-06	7,174	22.069	TRUE
ebi-PL179-GCST90277376	Sphingomyelin (d40:1) levels	rs4954960	2	138,816,573	C	T	0.7380	0.0906	0.0190	1.8961E-06	7,174	22.735	TRUE
ebi-PL179-GCST90277376	Sphingomyelin (d40:1) levels	rs111997200	19	44,963,068	C	T	0.5008	-0.0797	0.0167	1.9935E-06	7,174	22.635	TRUE
ebi-PL179-GCST90277376	Sphingomyelin (d40:1) levels	rs192913735	17	4,779,345	T	C	0.0091	-0.5621	0.0874	1.3583E-10	7,174	41.340	TRUE
ebi-PL179-GCST90277376	Sphingomyelin (d40:1) levels	rs59596298	20	41,301,845	C	G	0.4746	0.0887	0.0168	1.2711E-07	7,174	27.962	TRUE
ebi-PL179-GCST90277376	Sphingomyelin (d40:1) levels	rs80101718	4	72,375,030	A	G	0.0296	0.3344	0.0508	4.9660E-11	7,174	43.319	TRUE
ebi-PL179-GCST90277376	Sphingomyelin (d40:1) levels	rs4767003	12	112,753,113	C	T	0.6651	0.0873	0.0175	6.5240E-07	7,174	24.796	TRUE
ebi-PL179-GCST90277376	Sphingomyelin (d40:1) levels	rs76790474	17	4,498,679	A	G	0.2527	-0.1373	0.0194	1.6378E-12	7,174	50.048	TRUE
ebi-PL179-GCST90277376	Sphingomyelin (d40:1) levels	rs4949190	1	31,012,510	T	C	0.7727	-0.0986	0.0199	7.7798E-07	7,174	24.455	TRUE
ebi-PL179-GCST90277376	Sphingomyelin (d40:1) levels	rs8189230	11	48,806,055	A	G	0.0702	0.1563	0.0338	3.8765E-06	7,174	21.357	TRUE
ebi-PL179-GCST90277376	Sphingomyelin (d40:1) levels	rs10927285	1	236,024,983	G	C	0.3271	0.0823	0.0176	2.8621E-06	7,174	21.940	TRUE
ebi-PL179-GCST90277377	Sphingomyelin (d40:2) levels	rs2760512	1	192,592,415	G	A	0.3334	0.0835	0.0179	3.3117E-06	7,174	21.659	TRUE
ebi-PL179-GCST90277377	Sphingomyelin (d40:2) levels	rs79402526	4	168,864,851	C	T	0.0388	0.2138	0.0465	4.4345E-06	7,174	21.099	TRUE
ebi-PL179-GCST90277377	Sphingomyelin (d40:2) levels	rs56298236	2	210,741,300	T	C	0.2682	0.0957	0.0190	4.6117E-07	7,174	25.463	TRUE
ebi-PL179-GCST90277377	Sphingomyelin (d40:2) levels	rs10490233	2	50,201,990	A	T	0.1106	-0.1306	0.0264	7.6533E-07	7,174	24.486	TRUE
ebi-PL179-GCST90277377	Sphingomyelin (d40:2) levels	rs73641617	9	5,454,500	T	A	0.0176	0.2944	0.0637	3.8429E-06	7,174	21.370	TRUE
ebi-PL179-GCST90277377	Sphingomyelin (d40:2) levels	rs187144380	6	151,920,771	G	A	0.0111	0.3797	0.0816	3.3588E-06	7,174	21.631	TRUE
ebi-PL179-GCST90277377	Sphingomyelin (d40:2) levels	rs9839427	3	50,264,152	G	A	0.3253	-0.0812	0.0177	4.7512E-06	7,174	20.969	TRUE
ebi-PL179-GCST90277377	Sphingomyelin (d40:2) levels	rs115880518	3	20,089,961	G	A	0.0070	0.4675	0.1015	4.1994E-06	7,174	21.206	TRUE
ebi-PL179-GCST90277377	Sphingomyelin (d40:2) levels	rs17276933	14	20,017,746	A	T	0.0567	-0.1655	0.0361	4.6395E-06	7,174	21.011	TRUE
ebi-PL179-GCST90277378	Sphingomyelin (d42:2) levels	rs11610836	12	112,765,162	T	C	0.4225	0.0793	0.0166	1.7489E-06	7,174	22.891	TRUE
ebi-PL179-GCST90277378	Sphingomyelin (d42:2) levels	rs115350073	5	90,870,095	T	C	0.0181	0.2938	0.0633	3.5562E-06	7,174	21.523	TRUE
ebi-PL179-GCST90277378	Sphingomyelin (d42:2) levels	rs12530474	6	14,104,359	G	A	0.1335	-0.1108	0.0242	4.5578E-06	7,174	21.045	TRUE
ebi-PL179-GCST90277378	Sphingomyelin (d42:2) levels	rs515081	15	58,424,058	G	A	0.6879	-0.0917	0.0189	1.3061E-06	7,174	23.453	TRUE
ebi-PL179-GCST90277378	Sphingomyelin (d42:2) levels	rs4100654	9	104,906,960	C	T	0.0662	-0.1559	0.0337	3.7576E-06	7,174	21.418	TRUE
ebi-PL179-GCST90277378	Sphingomyelin (d42:2) levels	rs59916403	2	241,431,336	T	G	0.4133	0.0781	0.0169	3.8512E-06	7,174	21.370	TRUE
ebi-PL179-GCST90277378	Sphingomyelin (d42:2) levels	rs6843498	4	16,520,100	G	T	0.3119	0.0946	0.0188	4.9963E-07	7,174	25.313	TRUE
ebi-PL179-GCST90277378	Sphingomyelin (d42:2) levels	rs17110517	1	54,369,167	T	C	0.0610	-0.1671	0.0353	2.2601E-06	7,174	22.396	TRUE
ebi-PL179-GCST90277378	Sphingomyelin (d42:2) levels	rs17261772	2	135,153,852	C	T	0.3258	0.0900	0.0177	3.9386E-07	7,174	25.770	TRUE

Lipid GWAS ID	Lipid species	SNP	Chromosome	Position	Effect allele	Other allele	Effect-allele frequency	Beta	SE	P value	Sample size	F statistic	F > 10
ebi-PL179-GCST90277378	Sphingomyelin (d42:2) levels	rs1367117	2	21,041,028	A	G	0.2846	0.0995	0.0184	6.8810E-08	7,174	29.159	TRUE
ebi-PL179-GCST90277378	Sphingomyelin (d42:2) levels	rs62274367	3	103,515,604	C	T	0.0038	-0.6799	0.1455	3.0290E-06	7,174	21.833	TRUE
ebi-PL179-GCST90277378	Sphingomyelin (d42:2) levels	rs6680361	1	180,277,126	T	C	0.7949	0.0966	0.0211	4.5321E-06	7,174	21.056	TRUE
ebi-PL179-GCST90277378	Sphingomyelin (d42:2) levels	rs146331166	17	5,464,230	C	T	0.0596	-0.2186	0.0345	2.5553E-10	7,174	40.101	TRUE
ebi-PL179-GCST90277378	Sphingomyelin (d42:2) levels	rs3912622	11	103,250,744	C	A	0.2002	-0.1088	0.0209	1.9989E-07	7,174	27.086	TRUE
ebi-PL179-GCST90277378	Sphingomyelin (d42:2) levels	rs184845520	10	45,824,374	G	C	0.0052	0.5636	0.1228	4.4915E-06	7,174	21.075	TRUE
ebi-PL179-GCST90277378	Sphingomyelin (d42:2) levels	rs116893213	11	101,739,572	C	G	0.0370	0.2270	0.0448	4.1673E-07	7,174	25.664	TRUE
ebi-PL179-GCST90277378	Sphingomyelin (d42:2) levels	rs67718253	5	108,641,417	C	A	0.1765	-0.1016	0.0219	3.6479E-06	7,174	21.474	TRUE
ebi-PL179-GCST90277378	Sphingomyelin (d42:2) levels	rs73047641	19	44,954,120	A	G	0.4975	-0.0803	0.0168	1.7287E-06	7,174	22.911	TRUE
ebi-PL179-GCST90277238	Sterol ester (27:1/14:0) levels	rs4246215	11	61,796,827	T	G	0.4358	-0.0805	0.0169	1.8043E-06	7,174	22.829	TRUE
ebi-PL179-GCST90277238	Sterol ester (27:1/14:0) levels	rs7957768	12	101,754,541	T	G	0.5211	-0.0868	0.0167	2.2147E-07	7,174	26.885	TRUE
ebi-PL179-GCST90277238	Sterol ester (27:1/14:0) levels	rs11608	2	27,212,506	A	G	0.7234	0.0885	0.0188	2.5327E-06	7,174	22.176	TRUE
ebi-PL179-GCST90277238	Sterol ester (27:1/14:0) levels	rs1696359	12	120,950,756	C	T	0.5849	-0.0796	0.0170	2.8387E-06	7,174	21.954	TRUE
ebi-PL179-GCST90277238	Sterol ester (27:1/14:0) levels	rs6045698	20	1,965,583	C	A	0.2641	0.0898	0.0192	2.9575E-06	7,174	21.877	TRUE
ebi-PL179-GCST90277238	Sterol ester (27:1/14:0) levels	rs28601761	8	125,487,789	G	C	0.4100	-0.0841	0.0170	7.7075E-07	7,174	24.471	TRUE
ebi-PL179-GCST90277238	Sterol ester (27:1/14:0) levels	rs55764039	7	17,920,067	C	G	0.0527	-0.1808	0.0375	1.4143E-06	7,174	23.297	TRUE
ebi-PL179-GCST90277238	Sterol ester (27:1/14:0) levels	rs75922719	15	93,906,166	C	T	0.0068	0.4815	0.1027	2.7944E-06	7,174	21.987	TRUE
ebi-PL179-GCST90277238	Sterol ester (27:1/14:0) levels	rs116778355	2	224,951,925	T	A	0.0125	-0.3803	0.0777	1.0008E-06	7,174	23.967	TRUE
ebi-PL179-GCST90277238	Sterol ester (27:1/14:0) levels	rs62232045	22	43,503,538	C	T	0.3445	0.0859	0.0176	1.0447E-06	7,174	23.885	TRUE
ebi-PL179-GCST90277239	Sterol ester (27:1/15:0) levels	rs139857433	2	98,354,059	T	A	0.0087	-0.4601	0.1002	4.4923E-06	6,428	21.077	TRUE
ebi-PL179-GCST90277239	Sterol ester (27:1/15:0) levels	rs146236963	7	104,180,833	C	T	0.0165	-0.3272	0.0713	4.4914E-06	6,428	21.076	TRUE
ebi-PL179-GCST90277239	Sterol ester (27:1/15:0) levels	rs61859797	10	129,461,219	T	C	0.1776	0.1089	0.0232	2.6284E-06	6,428	22.108	TRUE
ebi-PL179-GCST90277239	Sterol ester (27:1/15:0) levels	rs55662279	5	175,777,765	T	C	0.0035	-0.7795	0.1660	2.7159E-06	6,428	22.045	TRUE
ebi-PL179-GCST90277239	Sterol ester (27:1/15:0) levels	rs113844909	4	26,516,933	C	G	0.0170	-0.3298	0.0703	2.7689E-06	6,428	22.009	TRUE
ebi-PL179-GCST90277240	Sterol ester (27:1/16:0) levels	rs10841310	12	19,677,962	T	G	0.2456	-0.0960	0.0194	7.8724E-07	7,174	24.431	TRUE
ebi-PL179-GCST90277240	Sterol ester (27:1/16:0) levels	rs7487904	12	53,231,170	G	C	0.1473	0.1094	0.0237	3.9414E-06	7,174	21.326	TRUE
ebi-PL179-GCST90277240	Sterol ester (27:1/16:0) levels	rs28627829	4	164,639,807	G	T	0.1345	-0.1187	0.0247	1.6286E-06	7,174	23.027	TRUE
ebi-PL179-GCST90277240	Sterol ester (27:1/16:0) levels	rs10031375	4	37,043,069	G	C	0.5578	0.0759	0.0165	4.1396E-06	7,174	21.230	TRUE
ebi-PL179-GCST90277240	Sterol ester (27:1/16:0) levels	rs56767222	3	3,342,886	C	T	0.0034	0.6674	0.1424	2.8394E-06	7,174	21.954	TRUE
ebi-PL179-GCST90277240	Sterol ester (27:1/16:0) levels	rs138270540	4	74,487,710	C	A	0.0281	0.3298	0.0507	8.3639E-11	7,174	42.291	TRUE
ebi-PL179-GCST90277240	Sterol ester (27:1/16:0) levels	rs1336770	6	124,502,870	G	A	0.5855	0.0796	0.0171	3.0732E-06	7,174	21.803	TRUE
ebi-PL179-GCST90277240	Sterol ester (27:1/16:0) levels	rs7167665	15	53,458,377	C	T	0.5384	0.0861	0.0167	2.3747E-07	7,174	26.751	TRUE
ebi-PL179-GCST90277240	Sterol ester (27:1/16:0) levels	rs7932326	11	43,971,474	A	G	0.6168	0.0788	0.0171	4.1252E-06	7,174	21.237	TRUE
ebi-PL179-GCST90277240	Sterol ester (27:1/16:0) levels	rs56336136	19	48,224,239	T	C	0.1045	0.1302	0.0275	2.3155E-06	7,174	22.349	TRUE
ebi-PL179-GCST90277240	Sterol ester (27:1/16:0) levels	rs10860778	12	101,714,807	T	C	0.4914	-0.0850	0.0167	3.8492E-07	7,174	25.814	TRUE
ebi-PL179-GCST90277240	Sterol ester (27:1/16:0) levels	rs11604424	11	116,780,399	T	C	0.7527	-0.1048	0.0192	5.0598E-08	7,174	29.757	TRUE
ebi-PL179-GCST90277240	Sterol ester (27:1/16:0) levels	rs9788072	12	4,796,048	G	A	0.0563	0.1697	0.0365	3.4067E-06	7,174	21.606	TRUE
ebi-PL179-GCST90277240	Sterol ester (27:1/16:0) levels	rs9924448	16	73,349,244	T	C	0.5573	-0.0845	0.0170	7.1520E-07	7,174	24.617	TRUE
ebi-PL179-GCST90277240	Sterol ester (27:1/16:0) levels	rs141836086	6	152,650,134	T	C	0.0334	0.2231	0.0463	1.4812E-06	7,174	23.209	TRUE
ebi-PL179-GCST90277240	Sterol ester (27:1/16:0) levels	rs3005923	1	56,335,870	A	G	0.0276	-0.2861	0.0529	6.5841E-08	7,174	29.245	TRUE
ebi-PL179-GCST90277241	Sterol ester (27:1/16:1) levels	rs274673	5	6,743,310	C	T	0.9517	0.1860	0.0393	2.2418E-06	7,172	22.411	TRUE
ebi-PL179-GCST90277241	Sterol ester (27:1/16:1) levels	rs201714080	16	71,095,785	A	C	0.0524	-0.1885	0.0381	7.6008E-07	7,172	24.500	TRUE
ebi-PL179-GCST90277241	Sterol ester (27:1/16:1) levels	rs142738732	1	1,329,726	A	T	0.0634	-0.1656	0.0350	2.1971E-06	7,172	22.449	TRUE
ebi-PL179-GCST90277241	Sterol ester (27:1/16:1) levels	rs116917626	17	51,561,964	T	C	0.0275	-0.2594	0.0525	7.8740E-07	7,172	24.426	TRUE
ebi-PL179-GCST90277241	Sterol ester (27:1/16:1) levels	rs7355269	2	62,072,939	C	T	0.0261	0.2432	0.0529	4.3512E-06	7,172	21.136	TRUE
ebi-PL179-GCST90277241	Sterol ester (27:1/16:1) levels	rs17603855	7	138,187,033	T	C	0.0575	0.1692	0.0357	2.1289E-06	7,172	22.510	TRUE
ebi-PL179-GCST90277241	Sterol ester (27:1/16:1) levels	rs11119973	1	212,561,372	A	C	0.1528	0.1107	0.0232	1.8434E-06	7,172	22.786	TRUE
ebi-PL179-GCST90277241	Sterol ester (27:1/16:1) levels	rs111656006	1	38,245,682	T	C	0.0483	0.1851	0.0400	3.8183E-06	7,172	21.384	TRUE
ebi-PL179-GCST90277242	Sterol ester (27:1/17:0) levels	rs10241012	7	104,334,784	G	A	0.1227	0.1182	0.0259	4.9765E-06	7,047	20.879	TRUE
ebi-PL179-GCST90277242	Sterol ester (27:1/17:0) levels	rs140015624	11	71,313,656	C	T	0.0158	0.3229	0.0690	2.9554E-06	7,047	21.878	TRUE
ebi-PL179-GCST90277242	Sterol ester (27:1/17:0) levels	rs10775274	16	25,212,475	C	T	0.9384	0.1704	0.0358	1.9327E-06	7,047	22.695	TRUE
ebi-PL179-GCST90277242	Sterol ester (27:1/17:0) levels	rs17791425	6	84,260,623	A	G	0.0500	0.1801	0.0394	4.8954E-06	7,047	20.906	TRUE
ebi-PL179-GCST90277242	Sterol ester (27:1/17:0) levels	rs635634	9	133,279,427	C	T	0.7979	-0.1002	0.0210	1.8383E-06	7,047	22.795	TRUE
ebi-PL179-GCST90277242	Sterol ester (27:1/17:0) levels	rs2280696	12	53,118,972	T	A	0.1262	0.2038	0.0252	6.3685E-16	7,047	65.624	TRUE
ebi-PL179-GCST90277242	Sterol ester (27:1/17:0) levels	rs79528439	15	48,709,000	A	T	0.1184	-0.1315	0.0266	7.5962E-07	7,047	24.499	TRUE
ebi-PL179-GCST90277242	Sterol ester (27:1/17:0) levels	rs28582101	22	36,797,580	G	C	0.8233	-0.1078	0.0228	2.2199E-06	7,047	22.430	TRUE
ebi-PL179-GCST90277242	Sterol ester (27:1/17:0) levels	rs11265455	1	160,650,361	G	A	0.1409	0.1193	0.0256	3.2981E-06	7,047	21.667	TRUE
ebi-PL179-GCST90277242	Sterol ester (27:1/17:0) levels	rs12921107	16	292,437	C	T	0.1415	-0.1253	0.0242	2.3800E-07	7,047	26.748	TRUE

Lipid GWAS ID	Lipid species	SNP	Chromosome	Position	Effect allele	Other allele	Effect-allele frequency	Beta	SE	P value	Sample size	F statistic	F > 10
ebi-PL179-GCST90277242	Sterol ester (27:1/17:0) levels	rs77943189	6	122,284,176	G	A	0.0160	0.3360	0.0707	2.0461E-06	7,047	22.587	TRUE
ebi-PL179-GCST90277242	Sterol ester (27:1/17:0) levels	rs1002079	16	76,465,100	G	C	0.1005	-0.1424	0.0280	3.8868E-07	7,047	25.797	TRUE
ebi-PL179-GCST90277242	Sterol ester (27:1/17:0) levels	rs13125535	4	12,263,787	G	T	0.5985	0.0861	0.0172	5.6070E-07	7,047	25.086	TRUE
ebi-PL179-GCST90277242	Sterol ester (27:1/17:0) levels	rs12296850	12	100,426,307	G	A	0.0543	0.1731	0.0376	4.2585E-06	7,047	21.176	TRUE
ebi-PL179-GCST90277243	Sterol ester (27:1/17:1) levels	rs40435	3	134,998,561	T	A	0.4058	-0.0786	0.0170	3.8081E-06	7,166	21.393	TRUE
ebi-PL179-GCST90277243	Sterol ester (27:1/17:1) levels	rs7957655	12	101,754,513	A	G	0.5210	-0.0779	0.0168	3.4087E-06	7,166	21.603	TRUE
ebi-PL179-GCST90277243	Sterol ester (27:1/17:1) levels	rs193228861	19	41,730,113	C	A	0.0084	-0.4230	0.0918	4.1409E-06	7,166	21.229	TRUE
ebi-PL179-GCST90277243	Sterol ester (27:1/17:1) levels	rs7151696	14	22,802,228	T	C	0.5698	0.0780	0.0170	4.3071E-06	7,166	21.154	TRUE
ebi-PL179-GCST90277243	Sterol ester (27:1/17:1) levels	rs76492272	18	70,340,195	A	G	0.0285	0.2578	0.0535	1.4973E-06	7,166	23.189	TRUE
ebi-PL179-GCST90277244	Sterol ester (27:1/18:0) levels	rs11130797	3	60,928,407	A	G	0.3003	0.0835	0.0182	4.5442E-06	7,157	21.051	TRUE
ebi-PL179-GCST90277244	Sterol ester (27:1/18:0) levels	rs55721138	3	13,583,299	T	C	0.0532	-0.1809	0.0376	1.5318E-06	7,157	23.145	TRUE
ebi-PL179-GCST90277244	Sterol ester (27:1/18:0) levels	rs17482800	3	137,801,985	A	G	0.0983	-0.1308	0.0280	2.9923E-06	7,157	21.853	TRUE
ebi-PL179-GCST90277244	Sterol ester (27:1/18:0) levels	rs144944992	1	164,790,578	T	C	0.0094	0.4946	0.0897	3.5845E-08	7,157	30.425	TRUE
ebi-PL179-GCST90277244	Sterol ester (27:1/18:0) levels	rs10735853	12	53,924,362	C	T	0.4822	-0.0790	0.0168	2.6140E-06	7,157	22.115	TRUE
ebi-PL179-GCST90277244	Sterol ester (27:1/18:0) levels	rs13405349	2	211,659,523	T	G	0.1851	-0.1006	0.0216	3.3456E-06	7,157	21.641	TRUE
ebi-PL179-GCST90277244	Sterol ester (27:1/18:0) levels	rs11154240	6	124,457,214	C	T	0.7827	0.0941	0.0202	3.1232E-06	7,157	21.772	TRUE
ebi-PL179-GCST90277244	Sterol ester (27:1/18:0) levels	rs134783	22	27,255,754	C	T	0.3981	0.0814	0.0171	2.1194E-06	7,157	22.518	TRUE
ebi-PL179-GCST90277244	Sterol ester (27:1/18:0) levels	rs30743	5	135,996,882	C	T	0.7633	-0.0951	0.0204	2.9882E-06	7,157	21.857	TRUE
ebi-PL179-GCST90277244	Sterol ester (27:1/18:0) levels	rs56228609	16	56,953,853	T	C	0.2755	-0.1038	0.0187	2.7326E-08	7,157	30.956	TRUE
ebi-PL179-GCST90277244	Sterol ester (27:1/18:0) levels	rs192928655	2	131,866,759	T	C	0.0236	-0.2851	0.0593	1.5844E-06	7,157	23.080	TRUE
ebi-PL179-GCST90277244	Sterol ester (27:1/18:0) levels	rs17725246	7	44,542,387	C	T	0.3388	0.0971	0.0177	4.1356E-08	7,157	30.147	TRUE
ebi-PL179-GCST90277244	Sterol ester (27:1/18:0) levels	rs11646327	16	29,117,117	G	A	0.2550	0.0933	0.0192	1.2425E-06	7,157	23.548	TRUE
ebi-PL179-GCST90277245	Sterol ester (27:1/18:1) levels	rs11687710	2	21,148,196	C	T	0.2571	0.0895	0.0192	3.0859E-06	7,174	21.795	TRUE
ebi-PL179-GCST90277245	Sterol ester (27:1/18:1) levels	rs73176681	13	31,500,828	G	A	0.0456	0.1910	0.0404	2.3268E-06	7,174	22.340	TRUE
ebi-PL179-GCST90277245	Sterol ester (27:1/18:1) levels	rs115419570	2	156,522,444	T	C	0.0204	0.2952	0.0597	7.7532E-07	7,174	24.460	TRUE
ebi-PL179-GCST90277245	Sterol ester (27:1/18:1) levels	rs701081	12	124,663,423	G	A	0.6033	-0.0836	0.0171	1.0898E-06	7,174	23.802	TRUE
ebi-PL179-GCST90277245	Sterol ester (27:1/18:1) levels	rs55833830	17	80,953,542	A	C	0.2292	-0.1070	0.0197	6.2127E-08	7,174	29.356	TRUE
ebi-PL179-GCST90277245	Sterol ester (27:1/18:1) levels	rs10078182	5	118,775,347	C	T	0.0218	0.2683	0.0587	4.9254E-06	7,174	20.896	TRUE
ebi-PL179-GCST90277245	Sterol ester (27:1/18:1) levels	rs4845593	1	154,078,023	C	T	0.0222	-0.2903	0.0571	3.8006E-07	7,174	25.838	TRUE
ebi-PL179-GCST90277245	Sterol ester (27:1/18:1) levels	rs117765824	18	53,959,046	C	G	0.0164	0.3183	0.0673	2.3198E-06	7,174	22.345	TRUE
ebi-PL179-GCST90277245	Sterol ester (27:1/18:1) levels	rs1061808	6	32,168,770	G	T	0.5841	-0.0814	0.0169	1.4724E-06	7,174	23.221	TRUE
ebi-PL179-GCST90277245	Sterol ester (27:1/18:1) levels	rs151274583	9	116,950,689	A	C	0.0176	0.2880	0.0630	4.9858E-06	7,174	20.873	TRUE
ebi-PL179-GCST90277246	Sterol ester (27:1/18:2) levels	rs17395333	2	21,100,497	G	A	0.2776	0.1037	0.0187	3.0556E-08	7,174	30.738	TRUE
ebi-PL179-GCST90277246	Sterol ester (27:1/18:2) levels	rs144064722	4	72,540,456	G	A	0.0324	0.2391	0.0475	4.8144E-07	7,174	25.382	TRUE
ebi-PL179-GCST90277246	Sterol ester (27:1/18:2) levels	rs117806675	10	5,384,541	A	G	0.0075	-0.4627	0.0983	2.5811E-06	7,174	22.140	TRUE
ebi-PL179-GCST90277246	Sterol ester (27:1/18:2) levels	rs10796911	1	37,622,895	C	G	0.8648	0.1167	0.0246	2.0633E-06	7,174	22.571	TRUE
ebi-PL179-GCST90277246	Sterol ester (27:1/18:2) levels	rs9330455	9	89,816,177	A	C	0.8285	0.1063	0.0231	4.3035E-06	7,174	21.155	TRUE
ebi-PL179-GCST90277246	Sterol ester (27:1/18:2) levels	rs9405846	6	5,542,174	G	A	0.5851	0.0776	0.0169	4.6283E-06	7,174	21.016	TRUE
ebi-PL179-GCST90277246	Sterol ester (27:1/18:2) levels	rs9304645	19	44,947,071	A	G	0.1656	-0.1227	0.0224	4.2013E-08	7,174	30.118	TRUE
ebi-PL179-GCST90277246	Sterol ester (27:1/18:2) levels	rs61796597	4	36,228,194	T	C	0.0376	-0.2085	0.0446	2.9729E-06	7,174	21.867	TRUE
ebi-PL179-GCST90277246	Sterol ester (27:1/18:2) levels	rs2524299	11	61,837,310	T	A	0.2594	0.1388	0.0190	3.2532E-13	7,174	53.247	TRUE
ebi-PL179-GCST90277246	Sterol ester (27:1/18:2) levels	rs73327386	5	172,483,791	C	T	0.0037	-0.6378	0.1377	3.6578E-06	7,174	21.469	TRUE
ebi-PL179-GCST90277247	Sterol ester (27:1/18:3) levels	rs174584	11	61,843,278	A	G	0.4099	-0.1832	0.0169	4.0219E-27	7,174	117.278	TRUE
ebi-PL179-GCST90277247	Sterol ester (27:1/18:3) levels	rs1980102	15	33,326,741	A	C	0.3745	0.0811	0.0171	2.2697E-06	7,174	22.387	TRUE
ebi-PL179-GCST90277247	Sterol ester (27:1/18:3) levels	rs2255531	12	120,977,112	A	G	0.3949	0.0934	0.0170	4.2466E-08	7,174	30.096	TRUE
ebi-PL179-GCST90277247	Sterol ester (27:1/18:3) levels	rs13172653	5	50,889,130	G	A	0.2281	0.0951	0.0202	2.6176E-06	7,174	22.113	TRUE
ebi-PL179-GCST90277247	Sterol ester (27:1/18:3) levels	rs58542926	19	19,268,740	T	C	0.0626	-0.1686	0.0344	1.0078E-06	7,174	23.953	TRUE
ebi-PL179-GCST90277247	Sterol ester (27:1/18:3) levels	rs34500711	9	130,101,985	T	C	0.0381	0.2095	0.0436	1.5873E-06	7,174	23.076	TRUE
ebi-PL179-GCST90277248	Sterol ester (27:1/20:2) levels	rs1404999	7	39,081,865	T	C	0.3137	-0.1019	0.0201	3.9936E-07	5,848	25.754	TRUE
ebi-PL179-GCST90277248	Sterol ester (27:1/20:2) levels	rs7933	2	43,225,044	G	A	0.3292	0.1056	0.0197	9.1351E-08	5,848	28.619	TRUE
ebi-PL179-GCST90277248	Sterol ester (27:1/20:2) levels	rs7853989	9	133,256,205	C	G	0.1559	-0.1333	0.0258	2.4088E-07	5,848	26.735	TRUE
ebi-PL179-GCST90277248	Sterol ester (27:1/20:2) levels	rs7202845	16	26,717,568	C	A	0.6705	0.0987	0.0198	6.4051E-07	5,848	24.840	TRUE
ebi-PL179-GCST90277248	Sterol ester (27:1/20:2) levels	rs56276984	8	23,548,281	T	C	0.0692	-0.1679	0.0362	3.5730E-06	5,848	21.521	TRUE
ebi-PL179-GCST90277248	Sterol ester (27:1/20:2) levels	rs36194627	2	225,135,785	A	G	0.0229	0.2930	0.0639	4.7040E-06	5,848	20.996	TRUE
ebi-PL179-GCST90277248	Sterol ester (27:1/20:2) levels	rs1903973	10	53,105,888	A	G	0.5558	0.0864	0.0184	2.6182E-06	5,848	22.119	TRUE
ebi-PL179-GCST90277248	Sterol ester (27:1/20:2) levels	rs6709904	2	43,853,185	G	A	0.0998	-0.2114	0.0319	3.7728E-11	5,848	43.893	TRUE
ebi-PL179-GCST90277248	Sterol ester (27:1/20:2) levels	rs118137821	10	7,348,594	T	C	0.0262	-0.2685	0.0587	4.9080E-06	5,848	20.910	TRUE
ebi-PL179-GCST90277248	Sterol ester (27:1/20:2) levels	rs55837134	7	44,532,058	A	G	0.2393	0.0994	0.0218	4.9480E-06	5,848	20.893	TRUE

Lipid GWAS ID	Lipid species	SNP	Chromosome	Position	Effect allele	Other allele	Effect-allele frequency	Beta	SE	P value	Sample size	F statistic	F > 10
ebi-PL179-GCST90277248	Sterol ester (27:1/20:2) levels	rs9504426	6	5,531,009	G	C	0.3777	-0.0923	0.0190	1.1641E-06	5,848	23.683	TRUE
ebi-PL179-GCST90277248	Sterol ester (27:1/20:2) levels	rs6490297	12	120,617,356	C	T	0.3132	-0.0942	0.0198	2.0423E-06	5,848	22.598	TRUE
ebi-PL179-GCST90277248	Sterol ester (27:1/20:2) levels	rs6755057	2	25,435,229	C	G	0.9345	-0.1799	0.0372	1.3305E-06	5,848	23.426	TRUE
ebi-PL179-GCST90277248	Sterol ester (27:1/20:2) levels	rs75823353	3	7,487,632	G	A	0.0729	0.1686	0.0357	2.3858E-06	5,848	22.298	TRUE
ebi-PL179-GCST90277248	Sterol ester (27:1/20:2) levels	rs4245791	2	43,847,292	T	C	0.7726	-0.2563	0.0217	9.1334E-32	5,848	139.179	TRUE
ebi-PL179-GCST90277248	Sterol ester (27:1/20:2) levels	rs567249312	2	61,591,159	A	G	0.0208	-0.3065	0.0641	1.8054E-06	5,848	22.838	TRUE
ebi-PL179-GCST90277248	Sterol ester (27:1/20:2) levels	rs4778971	15	79,180,180	G	A	0.6094	-0.0902	0.0197	4.6114E-06	5,848	21.029	TRUE
ebi-PL179-GCST90277249	Sterol ester (27:1/20:3) levels	rs1428498	5	38,698,557	G	A	0.3508	-0.0812	0.0172	2.4798E-06	7,171	22.216	TRUE
ebi-PL179-GCST90277249	Sterol ester (27:1/20:3) levels	rs685850	15	53,459,505	G	A	0.5565	0.0834	0.0168	7.0065E-07	7,171	24.655	TRUE
ebi-PL179-GCST90277249	Sterol ester (27:1/20:3) levels	rs4977342	9	19,882,242	G	C	0.2510	-0.0931	0.0198	2.4652E-06	7,171	22.228	TRUE
ebi-PL179-GCST90277249	Sterol ester (27:1/20:3) levels	rs2727271	11	61,835,886	T	A	0.2579	-0.1705	0.0190	3.9811E-19	7,171	80.325	TRUE
ebi-PL179-GCST90277249	Sterol ester (27:1/20:3) levels	rs1135999	16	15,038,105	G	A	0.3153	-0.2260	0.0178	1.9003E-36	7,171	160.746	TRUE
ebi-PL179-GCST90277249	Sterol ester (27:1/20:3) levels	rs7020	20	25,297,964	A	G	0.4458	0.0852	0.0169	4.6663E-07	7,171	25.442	TRUE
ebi-PL179-GCST90277249	Sterol ester (27:1/20:3) levels	rs80325645	9	131,892,383	C	T	0.0078	-0.4648	0.0966	1.5264E-06	7,171	23.152	TRUE
ebi-PL179-GCST90277249	Sterol ester (27:1/20:3) levels	rs112478356	2	173,688,057	C	G	0.0172	0.3158	0.0668	2.2943E-06	7,171	22.365	TRUE
ebi-PL179-GCST90277249	Sterol ester (27:1/20:3) levels	rs2352991	14	95,283,517	G	A	0.7348	-0.0889	0.0192	3.6140E-06	7,171	21.492	TRUE
ebi-PL179-GCST90277249	Sterol ester (27:1/20:3) levels	rs79771052	3	72,433,728	G	A	0.0120	0.3918	0.0807	1.2454E-06	7,171	23.544	TRUE
ebi-PL179-GCST90277249	Sterol ester (27:1/20:3) levels	rs3752149	19	15,729,265	C	A	0.2586	-0.0915	0.0193	2.2476E-06	7,171	22.406	TRUE
ebi-PL179-GCST90277249	Sterol ester (27:1/20:3) levels	rs530804537	1	55,117,537	A	G	0.0420	-0.2449	0.0417	4.3052E-09	7,171	34.566	TRUE
ebi-PL179-GCST90277249	Sterol ester (27:1/20:3) levels	rs111575978	14	46,693,947	A	G	0.0213	0.2891	0.0590	9.7042E-07	7,171	24.026	TRUE
ebi-PL179-GCST90277249	Sterol ester (27:1/20:3) levels	rs117754141	6	109,151,613	G	C	0.0226	0.2913	0.0598	1.1206E-06	7,171	23.748	TRUE
ebi-PL179-GCST90277249	Sterol ester (27:1/20:3) levels	rs117079226	9	109,704,483	A	G	0.0115	0.3756	0.0779	1.4657E-06	7,171	23.231	TRUE
ebi-PL179-GCST90277249	Sterol ester (27:1/20:3) levels	rs624605	11	76,043,576	T	G	0.4439	-0.0841	0.0174	1.3478E-06	7,171	23.392	TRUE
ebi-PL179-GCST90277249	Sterol ester (27:1/20:3) levels	rs62217148	20	18,988,572	T	C	0.0488	-0.1951	0.0405	1.4336E-06	7,171	23.273	TRUE
ebi-PL179-GCST90277250	Sterol ester (27:1/20:4) levels	rs117186386	16	87,676,514	C	T	0.0599	0.1780	0.0353	4.5985E-07	7,174	25.466	TRUE
ebi-PL179-GCST90277250	Sterol ester (27:1/20:4) levels	rs113248417	11	62,059,462	G	A	0.0369	-0.2680	0.0449	2.5950E-09	7,174	35.554	TRUE
ebi-PL179-GCST90277250	Sterol ester (27:1/20:4) levels	rs7837272	8	22,193,452	C	G	0.0021	0.8896	0.1937	4.4656E-06	7,174	21.086	TRUE
ebi-PL179-GCST90277250	Sterol ester (27:1/20:4) levels	rs186913978	2	17,147,264	T	G	0.0120	-0.4143	0.0773	8.5993E-08	7,174	28.728	TRUE
ebi-PL179-GCST90277250	Sterol ester (27:1/20:4) levels	rs11022403	11	12,542,442	T	C	0.3036	0.0845	0.0183	4.1492E-06	7,174	21.226	TRUE
ebi-PL179-GCST90277250	Sterol ester (27:1/20:4) levels	rs3767941	1	40,824,563	T	C	0.1665	0.1052	0.0221	2.0050E-06	7,174	22.624	TRUE
ebi-PL179-GCST90277250	Sterol ester (27:1/20:4) levels	rs2229738	11	68,794,860	T	C	0.1560	-0.1529	0.0234	7.0053E-11	7,174	42.645	TRUE
ebi-PL179-GCST90277250	Sterol ester (27:1/20:4) levels	rs9388335	6	124,438,655	A	T	0.5315	0.0782	0.0168	3.3458E-06	7,174	21.640	TRUE
ebi-PL179-GCST90277250	Sterol ester (27:1/20:4) levels	rs6812370	4	174,026,533	A	G	0.0166	-0.3443	0.0656	1.5490E-07	7,174	27.580	TRUE
ebi-PL179-GCST90277250	Sterol ester (27:1/20:4) levels	rs138482835	19	7,662,522	A	G	0.0612	-0.1723	0.0365	2.3946E-06	7,174	22.284	TRUE
ebi-PL179-GCST90277251	Sterol ester (27:1/20:5) levels	rs174581	11	61,839,211	A	G	0.4097	-0.4109	0.0164	3.5570E-133	7,174	629.250	TRUE
ebi-PL179-GCST90277251	Sterol ester (27:1/20:5) levels	rs449647	19	44,905,307	T	A	0.1364	-0.1334	0.0251	1.0706E-07	7,174	28.296	TRUE
ebi-PL179-GCST90277251	Sterol ester (27:1/20:5) levels	rs143678376	5	85,519,569	A	C	0.0777	0.1525	0.0313	1.1222E-06	7,174	23.745	TRUE
ebi-PL179-GCST90277251	Sterol ester (27:1/20:5) levels	rs2625222	1	204,934,830	C	T	0.1072	-0.1251	0.0271	3.8396E-06	7,174	21.375	TRUE
ebi-PL179-GCST90277251	Sterol ester (27:1/20:5) levels	rs11986253	8	132,397,243	C	G	0.1754	-0.1056	0.0226	3.0022E-06	7,174	21.848	TRUE
ebi-PL179-GCST90277251	Sterol ester (27:1/20:5) levels	rs34343838	1	169,678,964	A	G	0.1198	0.1241	0.0257	1.3314E-06	7,174	23.416	TRUE
ebi-PL179-GCST90277251	Sterol ester (27:1/20:5) levels	rs12515163	5	113,784,003	G	A	0.4003	-0.0820	0.0176	3.2105E-06	7,174	21.720	TRUE
ebi-PL179-GCST90277251	Sterol ester (27:1/20:5) levels	rs1481977	11	103,598,670	G	A	0.8610	0.1127	0.0244	3.7936E-06	7,174	21.398	TRUE
ebi-PL179-GCST90277251	Sterol ester (27:1/20:5) levels	rs980814	6	156,440,204	G	T	0.4547	0.0791	0.0170	3.5506E-06	7,174	21.526	TRUE
ebi-PL179-GCST90277252	Sterol ester (27:1/22:6) levels	rs9559135	13	107,677,268	G	A	0.2841	0.0855	0.0183	3.1689E-06	7,173	21.744	TRUE
ebi-PL179-GCST90277252	Sterol ester (27:1/22:6) levels	rs149229417	3	68,666,055	A	C	0.0171	0.3181	0.0660	1.4450E-06	7,173	23.257	TRUE
ebi-PL179-GCST90277252	Sterol ester (27:1/22:6) levels	rs116794590	4	13,024,335	C	T	0.0255	-0.2481	0.0533	3.2769E-06	7,173	21.680	TRUE
ebi-PL179-GCST90277252	Sterol ester (27:1/22:6) levels	rs11633878	15	93,239,374	A	G	0.7188	-0.0975	0.0205	1.9731E-06	7,173	22.658	TRUE
ebi-PL179-GCST90277252	Sterol ester (27:1/22:6) levels	rs75101588	6	24,387,133	C	T	0.0300	0.2589	0.0499	2.2285E-07	7,173	26.874	TRUE
ebi-PL179-GCST90277252	Sterol ester (27:1/22:6) levels	rs77456635	15	64,865,848	A	G	0.0242	-0.2682	0.0536	5.6775E-07	7,173	25.062	TRUE
ebi-PL179-GCST90277252	Sterol ester (27:1/22:6) levels	rs174554	11	61,811,991	G	A	0.4059	-0.2010	0.0169	2.2609E-32	7,173	141.703	TRUE
ebi-PL179-GCST90277252	Sterol ester (27:1/22:6) levels	rs4072727	18	48,039,184	C	T	0.3853	-0.0926	0.0172	7.1112E-08	7,173	29.093	TRUE
ebi-PL179-GCST90277252	Sterol ester (27:1/22:6) levels	rs72852391	17	78,966,695	T	C	0.0391	0.2131	0.0450	2.1798E-06	7,173	22.464	TRUE
ebi-PL179-GCST90277252	Sterol ester (27:1/22:6) levels	rs115647337	4	91,336,094	T	G	0.0412	-0.1980	0.0425	3.2360E-06	7,173	21.704	TRUE
ebi-PL179-GCST90277379	Triacylglycerol (46:1) levels	rs16853823	2	215,338,573	G	A	0.2233	0.1003	0.0219	4.5410E-06	6,070	21.059	TRUE
ebi-PL179-GCST90277379	Triacylglycerol (46:1) levels	rs12301917	12	81,672,829	T	C	0.1887	0.1133	0.0233	1.1645E-06	6,070	23.681	TRUE
ebi-PL179-GCST90277379	Triacylglycerol (46:1) levels	rs117591451	8	40,367,808	A	G	0.0131	-0.4015	0.0849	2.2727E-06	6,070	22.391	TRUE
ebi-PL179-GCST90277379	Triacylglycerol (46:1) levels	rs79152531	19	55,802,466	C	T	0.0603	-0.1951	0.0390	5.8891E-07	6,070	24.999	TRUE
ebi-PL179-GCST90277379	Triacylglycerol (46:1) levels	rs11160050	14	92,187,131	G	A	0.1163	-0.1331	0.0290	4.4175E-06	6,070	21.111	TRUE

Lipid GWAS ID	Lipid species	SNP	Chromosome	Position	Effect allele	Other allele	Effect-allele frequency	Beta	SE	P value	Sample size	F statistic	F > 10
ebi-PL179-GCST90277379	Triacylglycerol (46:1) levels	rs12225049	11	110,379,615	T	C	0.1686	-0.1167	0.0242	1.4396E-06	6,070	23.271	TRUE
ebi-PL179-GCST90277379	Triacylglycerol (46:1) levels	rs117730598	9	2,770,506	C	T	0.0299	0.2633	0.0541	1.1591E-06	6,070	23.691	TRUE
ebi-PL179-GCST90277379	Triacylglycerol (46:1) levels	rs35840156	17	77,816,410	T	C	0.0264	0.2735	0.0576	2.1397E-06	6,070	22.507	TRUE
ebi-PL179-GCST90277379	Triacylglycerol (46:1) levels	rs9796793	16	30,484,331	A	G	0.1248	-0.1309	0.0283	3.8060E-06	6,070	21.397	TRUE
ebi-PL179-GCST90277379	Triacylglycerol (46:1) levels	rs111568723	9	132,470,502	G	A	0.0259	0.2789	0.0593	2.6336E-06	6,070	22.106	TRUE
ebi-PL179-GCST90277379	Triacylglycerol (46:1) levels	rs1553368	17	53,571,810	G	A	0.6138	-0.0912	0.0187	1.1671E-06	6,070	23.675	TRUE
ebi-PL179-GCST90277379	Triacylglycerol (46:1) levels	rs16923137	8	58,158,358	T	A	0.0521	0.1862	0.0405	4.3719E-06	6,070	21.131	TRUE
ebi-PL179-GCST90277380	Triacylglycerol (46:2) levels	rs2084594	11	26,783,827	A	G	0.4606	0.0913	0.0191	1.7932E-06	5,448	22.853	TRUE
ebi-PL179-GCST90277380	Triacylglycerol (46:2) levels	rs778957	4	62,386,287	C	G	0.1196	-0.1434	0.0296	1.2981E-06	5,448	23.476	TRUE
ebi-PL179-GCST90277380	Triacylglycerol (46:2) levels	rs191616475	8	58,330,047	G	A	0.0481	0.2247	0.0441	3.6439E-07	5,448	25.937	TRUE
ebi-PL179-GCST90277380	Triacylglycerol (46:2) levels	rs78056622	1	184,416,563	G	C	0.0110	0.4724	0.0960	8.7238E-07	5,448	24.244	TRUE
ebi-PL179-GCST90277380	Triacylglycerol (46:2) levels	rs6474079	8	56,711,024	T	C	0.8168	-0.1151	0.0246	2.8788E-06	5,448	21.939	TRUE
ebi-PL179-GCST90277380	Triacylglycerol (46:2) levels	rs73226143	13	76,166,574	C	T	0.1362	0.1296	0.0279	3.5193E-06	5,448	21.552	TRUE
ebi-PL179-GCST90277380	Triacylglycerol (46:2) levels	rs6504835	17	53,554,728	A	T	0.4117	-0.0945	0.0196	1.5309E-06	5,448	23.158	TRUE
ebi-PL179-GCST90277380	Triacylglycerol (46:2) levels	rs2018873	19	15,080,523	T	C	0.6033	0.0927	0.0201	4.2774E-06	5,448	21.176	TRUE
ebi-PL179-GCST90277381	Triacylglycerol (48:0) levels	rs10218823	1	6,029,418	T	G	0.0174	-0.3517	0.0759	3.6320E-06	5,463	21.495	TRUE
ebi-PL179-GCST90277381	Triacylglycerol (48:0) levels	rs117264209	9	135,992,678	G	C	0.0190	-0.3315	0.0724	4.8367E-06	5,463	20.941	TRUE
ebi-PL179-GCST90277381	Triacylglycerol (48:0) levels	rs2180528	20	15,445,391	T	C	0.3885	0.0959	0.0195	9.3113E-07	5,463	24.118	TRUE
ebi-PL179-GCST90277381	Triacylglycerol (48:0) levels	rs76375876	19	49,524,378	T	C	0.0257	0.2959	0.0631	2.7836E-06	5,463	22.005	TRUE
ebi-PL179-GCST90277381	Triacylglycerol (48:0) levels	rs769432	17	3,420,961	T	C	0.5094	0.0910	0.0191	2.0370E-06	5,463	22.605	TRUE
ebi-PL179-GCST90277381	Triacylglycerol (48:0) levels	rs222507	12	101,725,095	T	G	0.9791	-0.3227	0.0697	3.6820E-06	5,463	21.464	TRUE
ebi-PL179-GCST90277381	Triacylglycerol (48:0) levels	rs4556833	17	53,567,083	C	A	0.4410	-0.0893	0.0194	4.1108E-06	5,463	21.252	TRUE
ebi-PL179-GCST90277381	Triacylglycerol (48:0) levels	rs62348866	4	186,453,244	A	G	0.1184	-0.1413	0.0297	2.0095E-06	5,463	22.633	TRUE
ebi-PL179-GCST90277381	Triacylglycerol (48:0) levels	rs4955801	3	179,092,711	C	T	0.7849	-0.1069	0.0232	4.0903E-06	5,463	21.263	TRUE
ebi-PL179-GCST90277381	Triacylglycerol (48:0) levels	rs2242104	9	2,640,492	G	A	0.7516	-0.1025	0.0222	4.1640E-06	5,463	21.229	TRUE
ebi-PL179-GCST90277381	Triacylglycerol (48:0) levels	rs10425025	19	9,009,145	A	T	0.0227	0.3048	0.0664	4.5484E-06	5,463	21.059	TRUE
ebi-PL179-GCST90277381	Triacylglycerol (48:0) levels	rs62235973	22	37,593,713	A	G	0.0372	0.2409	0.0512	2.5399E-06	5,463	22.180	TRUE
ebi-PL179-GCST90277382	Triacylglycerol (48:1) levels	rs75178403	8	2,825,696	C	T	0.0096	-0.4139	0.0899	4.1921E-06	7,019	21.206	TRUE
ebi-PL179-GCST90277382	Triacylglycerol (48:1) levels	rs10861498	12	105,758,014	A	G	0.2423	-0.0952	0.0194	9.0952E-07	7,019	24.152	TRUE
ebi-PL179-GCST90277382	Triacylglycerol (48:1) levels	rs3127050	13	60,832,603	A	G	0.7182	0.0883	0.0186	2.1771E-06	7,019	22.467	TRUE
ebi-PL179-GCST90277382	Triacylglycerol (48:1) levels	rs16970164	15	39,999,889	C	A	0.0394	-0.2024	0.0426	2.0428E-06	7,019	22.591	TRUE
ebi-PL179-GCST90277383	Triacylglycerol (48:2) levels	rs2546043	19	34,971,287	G	A	0.5326	-0.0802	0.0167	1.6563E-06	7,071	22.994	TRUE
ebi-PL179-GCST90277383	Triacylglycerol (48:2) levels	rs79213832	22	23,971,765	G	A	0.0600	0.1714	0.0358	1.6845E-06	7,071	22.962	TRUE
ebi-PL179-GCST90277383	Triacylglycerol (48:2) levels	rs113777805	18	23,684,659	C	G	0.0307	0.2381	0.0498	1.7753E-06	7,071	22.862	TRUE
ebi-PL179-GCST90277383	Triacylglycerol (48:2) levels	rs10084264	2	163,558,771	T	G	0.0591	0.1721	0.0364	2.2562E-06	7,071	22.399	TRUE
ebi-PL179-GCST90277383	Triacylglycerol (48:2) levels	rs72738698	9	80,090,365	A	G	0.1793	0.1016	0.0221	4.1588E-06	7,071	21.222	TRUE
ebi-PL179-GCST90277384	Triacylglycerol (48:3) levels	rs9837572	3	72,260,449	T	G	0.2355	-0.0925	0.0202	4.8248E-06	6,855	20.937	TRUE
ebi-PL179-GCST90277384	Triacylglycerol (48:3) levels	rs72846885	11	7,455,964	G	A	0.0865	0.1396	0.0305	4.9241E-06	6,855	20.898	TRUE
ebi-PL179-GCST90277384	Triacylglycerol (48:3) levels	rs56082136	12	52,243,392	T	C	0.0526	0.1892	0.0393	1.4859E-06	6,855	23.205	TRUE
ebi-PL179-GCST90277385	Triacylglycerol (49:1) levels	rs146238333	12	83,707,933	T	A	0.0052	0.6273	0.1337	2.7478E-06	5,843	22.027	TRUE
ebi-PL179-GCST90277385	Triacylglycerol (49:1) levels	rs8055088	16	6,818,646	T	A	0.5105	-0.0849	0.0186	4.9674E-06	5,843	20.887	TRUE
ebi-PL179-GCST90277385	Triacylglycerol (49:1) levels	rs33951980	7	73,615,107	T	C	0.1218	-0.1301	0.0284	4.9062E-06	5,843	20.911	TRUE
ebi-PL179-GCST90277386	Triacylglycerol (49:2) levels	rs76411296	15	59,517,420	T	G	0.1162	-0.1360	0.0287	2.2756E-06	6,076	22.385	TRUE
ebi-PL179-GCST90277386	Triacylglycerol (49:2) levels	rs143838492	5	114,892,988	A	G	0.0033	0.7577	0.1588	1.8779E-06	6,076	22.759	TRUE
ebi-PL179-GCST90277386	Triacylglycerol (49:2) levels	rs71556492	6	107,854,775	C	T	0.1647	0.1137	0.0248	4.5138E-06	6,076	21.071	TRUE
ebi-PL179-GCST90277386	Triacylglycerol (49:2) levels	rs4937417	11	129,348,709	G	A	0.4668	-0.0849	0.0183	3.3920E-06	6,076	21.618	TRUE
ebi-PL179-GCST90277386	Triacylglycerol (49:2) levels	rs1810258	17	66,539,562	C	T	0.4285	0.0848	0.0185	4.4939E-06	6,076	21.080	TRUE
ebi-PL179-GCST90277386	Triacylglycerol (49:2) levels	rs6846577	4	64,367,871	A	G	0.2154	-0.1010	0.0219	4.2710E-06	6,076	21.176	TRUE
ebi-PL179-GCST90277386	Triacylglycerol (49:2) levels	rs145055632	6	50,783,096	G	A	0.0064	-0.5156	0.1122	4.3606E-06	6,076	21.136	TRUE
ebi-PL179-GCST90277386	Triacylglycerol (49:2) levels	rs2736863	8	133,451,589	G	T	0.9921	0.4634	0.1013	4.8083E-06	6,076	20.948	TRUE
ebi-PL179-GCST90277387	Triacylglycerol (50:1) levels	rs62016118	16	6,791,691	C	A	0.0508	-0.1769	0.0384	4.0801E-06	7,143	21.259	TRUE
ebi-PL179-GCST90277387	Triacylglycerol (50:1) levels	rs79416109	1	102,847,102	G	T	0.1041	0.1335	0.0281	2.0472E-06	7,143	22.586	TRUE
ebi-PL179-GCST90277387	Triacylglycerol (50:1) levels	rs75931581	15	39,994,335	C	T	0.0419	-0.2018	0.0414	1.1307E-06	7,143	23.731	TRUE
ebi-PL179-GCST90277387	Triacylglycerol (50:1) levels	rs1399627	2	226,218,695	G	A	0.6316	0.0834	0.0174	1.7600E-06	7,143	22.876	TRUE
ebi-PL179-GCST90277387	Triacylglycerol (50:1) levels	rs61906420	11	132,660,922	A	G	0.0041	0.6752	0.1407	1.6359E-06	7,143	23.019	TRUE
ebi-PL179-GCST90277388	Triacylglycerol (50:2) levels	rs111430300	2	36,390,910	G	A	0.1355	-0.1124	0.0242	3.3627E-06	7,172	21.631	TRUE
ebi-PL179-GCST90277388	Triacylglycerol (50:2) levels	rs79934453	6	23,919,006	G	A	0.0040	-0.6431	0.1370	2.7152E-06	7,172	22.042	TRUE
ebi-PL179-GCST90277388	Triacylglycerol (50:2) levels	rs6033833	20	13,842,673	G	C	0.6400	-0.0784	0.0171	4.4805E-06	7,172	21.078	TRUE

Lipid GWAS ID	Lipid species	SNP	Chromosome	Position	Effect allele	Other allele	Effect-allele frequency	Beta	SE	P value	Sample size	F statistic	F > 10
ebi-PL179-GCST90277389	Triacylglycerol (50:3) levels	rs79954170	6	49,123,122	A	C	0.0081	-0.4402	0.0957	4.3151E-06	7,161	21.151	TRUE
ebi-PL179-GCST90277389	Triacylglycerol (50:3) levels	rs139499638	4	120,830,005	C	G	0.0044	-0.6429	0.1361	2.3506E-06	7,161	22.318	TRUE
ebi-PL179-GCST90277389	Triacylglycerol (50:3) levels	rs35368205	7	73,603,327	T	C	0.1666	-0.1028	0.0223	4.0327E-06	7,161	21.280	TRUE
ebi-PL179-GCST90277389	Triacylglycerol (50:3) levels	rs1428264	8	59,082,215	C	A	0.1975	0.0987	0.0209	2.2394E-06	7,161	22.413	TRUE
ebi-PL179-GCST90277389	Triacylglycerol (50:3) levels	rs10105606	8	19,970,337	A	C	0.2788	-0.1010	0.0185	5.1023E-08	7,161	29.739	TRUE
ebi-PL179-GCST90277390	Triacylglycerol (50:4) levels	rs116497138	3	87,180,483	T	G	0.0498	-0.1841	0.0392	2.6839E-06	7,166	22.064	TRUE
ebi-PL179-GCST90277390	Triacylglycerol (50:4) levels	rs12120691	1	227,027,789	C	T	0.1235	-0.1185	0.0256	3.7136E-06	7,166	21.439	TRUE
ebi-PL179-GCST90277390	Triacylglycerol (50:4) levels	rs190411877	10	7,871,139	C	T	0.0094	0.4157	0.0880	2.3849E-06	7,166	22.291	TRUE
ebi-PL179-GCST90277390	Triacylglycerol (50:4) levels	rs55798552	17	49,153,799	T	C	0.1210	0.1207	0.0253	1.8191E-06	7,166	22.814	TRUE
ebi-PL179-GCST90277390	Triacylglycerol (50:4) levels	rs11783556	8	764,450	G	A	0.2004	-0.0996	0.0211	2.3489E-06	7,166	22.321	TRUE
ebi-PL179-GCST90277390	Triacylglycerol (50:4) levels	rs1832326	9	33,656,919	C	A	0.7250	0.0856	0.0186	4.5094E-06	7,166	21.066	TRUE
ebi-PL179-GCST90277390	Triacylglycerol (50:4) levels	rs12335973	9	132,460,035	C	T	0.0481	0.1835	0.0390	2.5761E-06	7,166	22.142	TRUE
ebi-PL179-GCST90277390	Triacylglycerol (50:4) levels	rs301555	3	11,749,984	G	A	0.3396	0.0814	0.0177	4.3631E-06	7,166	21.129	TRUE
ebi-PL179-GCST90277390	Triacylglycerol (50:4) levels	rs668013	18	79,647,516	A	G	0.5452	0.0779	0.0169	4.3402E-06	7,166	21.139	TRUE
ebi-PL179-GCST90277391	Triacylglycerol (50:5) levels	rs187087759	5	176,875,666	G	C	0.0123	-0.4216	0.0920	4.6505E-06	5,491	21.016	TRUE
ebi-PL179-GCST90277391	Triacylglycerol (50:5) levels	rs2278983	16	70,692,892	A	C	0.5365	0.0885	0.0193	4.7571E-06	5,491	20.973	TRUE
ebi-PL179-GCST90277391	Triacylglycerol (50:5) levels	rs7241605	18	44,974,706	G	A	0.1039	0.1477	0.0315	2.8208E-06	5,491	21.980	TRUE
ebi-PL179-GCST90277391	Triacylglycerol (50:5) levels	rs888860	19	34,974,283	C	A	0.6206	-0.0945	0.0196	1.3922E-06	5,491	23.338	TRUE
ebi-PL179-GCST90277391	Triacylglycerol (50:5) levels	rs2915945	19	38,522,685	C	T	0.2602	0.1000	0.0216	3.8973E-06	5,491	21.357	TRUE
ebi-PL179-GCST90277391	Triacylglycerol (50:5) levels	rs571678190	11	21,669,490	G	A	0.0536	-0.2199	0.0439	5.4579E-07	5,491	25.152	TRUE
ebi-PL179-GCST90277391	Triacylglycerol (50:5) levels	rs67080320	8	139,794,453	C	T	0.0508	-0.2032	0.0436	3.2451E-06	5,491	21.709	TRUE
ebi-PL179-GCST90277391	Triacylglycerol (50:5) levels	rs254668	5	157,845,455	A	G	0.4415	0.1010	0.0191	1.2832E-07	5,491	27.963	TRUE
ebi-PL179-GCST90277391	Triacylglycerol (50:5) levels	rs3906419	5	108,951,913	T	A	0.2196	0.1082	0.0229	2.2785E-06	5,491	22.390	TRUE
ebi-PL179-GCST90277391	Triacylglycerol (50:5) levels	rs72646948	22	19,759,510	G	C	0.0042	0.8489	0.1626	1.8478E-07	5,491	27.252	TRUE
ebi-PL179-GCST90277392	Triacylglycerol (51:1) levels	rs7076657	10	132,832,356	G	C	0.0651	0.1796	0.0390	4.2131E-06	5,495	21.207	TRUE
ebi-PL179-GCST90277392	Triacylglycerol (51:1) levels	rs4979284	9	113,789,023	A	G	0.6774	-0.0967	0.0204	2.1283E-06	5,495	22.522	TRUE
ebi-PL179-GCST90277392	Triacylglycerol (51:1) levels	rs34496841	17	51,439,207	A	G	0.2367	-0.1124	0.0224	5.1492E-07	5,495	25.265	TRUE
ebi-PL179-GCST90277392	Triacylglycerol (51:1) levels	rs1928423	9	71,635,471	C	A	0.6474	-0.1017	0.0206	8.5584E-07	5,495	24.282	TRUE
ebi-PL179-GCST90277392	Triacylglycerol (51:1) levels	rs140828820	4	34,767,471	G	A	0.0106	0.4513	0.0952	2.1781E-06	5,495	22.478	TRUE
ebi-PL179-GCST90277392	Triacylglycerol (51:1) levels	rs11102221	1	111,242,194	A	G	0.1980	-0.1103	0.0238	3.7347E-06	5,495	21.438	TRUE
ebi-PL179-GCST90277392	Triacylglycerol (51:1) levels	rs2169099	4	62,360,675	C	T	0.1993	-0.1171	0.0241	1.2143E-06	5,495	23.605	TRUE
ebi-PL179-GCST90277392	Triacylglycerol (51:1) levels	rs4448624	10	85,979,448	A	G	0.4696	-0.0877	0.0192	4.8227E-06	5,495	20.946	TRUE
ebi-PL179-GCST90277393	Triacylglycerol (51:2) levels	rs6721083	2	170,225,142	C	G	0.9365	0.1607	0.0350	4.4336E-06	7,149	21.098	TRUE
ebi-PL179-GCST90277393	Triacylglycerol (51:2) levels	rs62043594	15	93,154,154	A	C	0.4178	-0.0796	0.0168	2.1573E-06	7,149	22.485	TRUE
ebi-PL179-GCST90277393	Triacylglycerol (51:2) levels	rs10147474	14	29,823,442	G	A	0.0372	-0.2271	0.0447	3.9817E-07	7,149	25.749	TRUE
ebi-PL179-GCST90277394	Triacylglycerol (51:3) levels	rs390082	19	44,913,574	G	T	0.0773	0.1507	0.0313	1.5127E-06	7,119	23.170	TRUE
ebi-PL179-GCST90277394	Triacylglycerol (51:3) levels	rs35397574	3	148,395,586	G	A	0.4833	-0.0799	0.0170	2.7175E-06	7,119	22.040	TRUE
ebi-PL179-GCST90277394	Triacylglycerol (51:3) levels	rs15285	8	19,967,156	T	C	0.2611	-0.1058	0.0190	2.6155E-08	7,119	31.042	TRUE
ebi-PL179-GCST90277394	Triacylglycerol (51:3) levels	rs146131769	6	49,493,994	G	A	0.0084	-0.4479	0.0952	2.5796E-06	7,119	22.141	TRUE
ebi-PL179-GCST90277394	Triacylglycerol (51:3) levels	rs10767577	11	26,777,276	A	T	0.4283	0.0778	0.0168	3.6847E-06	7,119	21.454	TRUE
ebi-PL179-GCST90277394	Triacylglycerol (51:3) levels	rs6692319	1	230,148,628	T	C	0.7398	-0.0882	0.0189	2.9291E-06	7,119	21.896	TRUE
ebi-PL179-GCST90277394	Triacylglycerol (51:3) levels	rs11728165	4	107,886,862	T	C	0.0804	-0.1483	0.0322	4.0782E-06	7,119	21.258	TRUE
ebi-PL179-GCST90277395	Triacylglycerol (51:4) levels	rs2853923	6	31,297,960	G	A	0.5052	0.0953	0.0195	1.0300E-06	5,237	23.925	TRUE
ebi-PL179-GCST90277395	Triacylglycerol (51:4) levels	rs116923150	9	112,114,209	A	G	0.0032	0.9554	0.1922	6.8396E-07	5,237	24.716	TRUE
ebi-PL179-GCST90277395	Triacylglycerol (51:4) levels	rs11782705	8	4,633,421	C	T	0.0590	-0.2001	0.0421	2.0201E-06	5,237	22.625	TRUE
ebi-PL179-GCST90277395	Triacylglycerol (51:4) levels	rs182295482	10	71,191,707	C	T	0.0078	-0.5467	0.1191	4.5394E-06	5,237	21.066	TRUE
ebi-PL179-GCST90277395	Triacylglycerol (51:4) levels	rs62111901	19	13,789,855	A	G	0.1713	-0.1250	0.0266	2.5726E-06	5,237	22.158	TRUE
ebi-PL179-GCST90277395	Triacylglycerol (51:4) levels	rs71605604	4	86,536,048	T	C	0.0301	0.2617	0.0568	4.1622E-06	5,237	21.231	TRUE
ebi-PL179-GCST90277395	Triacylglycerol (51:4) levels	rs112421492	13	24,038,710	T	A	0.0024	-0.9708	0.1967	8.2224E-07	5,237	24.362	TRUE
ebi-PL179-GCST90277395	Triacylglycerol (51:4) levels	rs62002515	15	59,004,639	C	T	0.0193	-0.3282	0.0706	3.4638E-06	5,237	21.589	TRUE
ebi-PL179-GCST90277395	Triacylglycerol (51:4) levels	rs149992851	3	55,269,076	G	A	0.0198	0.3379	0.0695	1.1718E-06	5,237	23.675	TRUE
ebi-PL179-GCST90277395	Triacylglycerol (51:4) levels	rs17514333	13	105,939,568	G	A	0.2092	0.1104	0.0238	3.4062E-06	5,237	21.622	TRUE
ebi-PL179-GCST90277395	Triacylglycerol (51:4) levels	rs966039	3	66,778,832	G	A	0.1640	-0.1268	0.0267	2.0855E-06	5,237	22.562	TRUE
ebi-PL179-GCST90277395	Triacylglycerol (51:4) levels	rs438811	19	44,913,484	T	C	0.2422	0.1149	0.0229	5.0941E-07	5,237	25.289	TRUE
ebi-PL179-GCST90277396	Triacylglycerol (52:2) levels	rs1010313	8	59,089,983	T	C	0.1980	0.0965	0.0208	3.4182E-06	7,168	21.599	TRUE
ebi-PL179-GCST90277396	Triacylglycerol (52:2) levels	rs34621709	1	220,806,736	A	C	0.1847	0.1078	0.0216	5.8670E-07	7,168	24.999	TRUE
ebi-PL179-GCST90277396	Triacylglycerol (52:2) levels	rs144241030	1	103,243,167	T	C	0.0772	0.1499	0.0322	3.2597E-06	7,168	21.690	TRUE
ebi-PL179-GCST90277396	Triacylglycerol (52:2) levels	rs78275146	1	196,028,959	T	C	0.0138	0.3496	0.0753	3.4939E-06	7,168	21.555	TRUE

Lipid GWAS ID	Lipid species	SNP	Chromosome	Position	Effect allele	Other allele	Effect-allele frequency	Beta	SE	P value	Sample size	F statistic	F > 10
ebi-PL179-GCST90277396	Triacylglycerol (52:2) levels	rs147987331	2	104,244,849	A	T	0.0227	-0.2610	0.0565	3.9584E-06	7,168	21.317	TRUE
ebi-PL179-GCST90277397	Triacylglycerol (52:3) levels	rs144072769	6	154,916,245	C	T	0.0682	0.1574	0.0336	2.8410E-06	7,173	21.955	TRUE
ebi-PL179-GCST90277397	Triacylglycerol (52:3) levels	rs571070279	5	114,162,441	T	C	0.0250	0.2470	0.0534	3.8827E-06	7,173	21.353	TRUE
ebi-PL179-GCST90277397	Triacylglycerol (52:3) levels	rs9997195	4	113,004,554	C	G	0.6128	-0.0810	0.0172	2.6142E-06	7,173	22.116	TRUE
ebi-PL179-GCST90277397	Triacylglycerol (52:3) levels	rs57500102	3	171,536,246	A	G	0.0073	-0.4989	0.1069	3.1030E-06	7,173	21.786	TRUE
ebi-PL179-GCST90277397	Triacylglycerol (52:3) levels	rs116843064	19	8,364,439	A	G	0.0259	-0.2415	0.0514	2.6985E-06	7,173	22.054	TRUE
ebi-PL179-GCST90277397	Triacylglycerol (52:3) levels	rs117950471	22	32,729,795	T	C	0.0512	0.1768	0.0379	3.2381E-06	7,173	21.703	TRUE
ebi-PL179-GCST90277397	Triacylglycerol (52:3) levels	rs2980888	8	125,495,066	C	T	0.7433	-0.0886	0.0191	3.5068E-06	7,173	21.550	TRUE
ebi-PL179-GCST90277397	Triacylglycerol (52:3) levels	rs75237020	17	72,177,274	C	G	0.0346	-0.2163	0.0473	4.9599E-06	7,173	20.883	TRUE
ebi-PL179-GCST90277398	Triacylglycerol (52:4) levels	rs56051325	17	1,615,646	G	A	0.3017	0.0884	0.0185	1.7245E-06	7,174	22.917	TRUE
ebi-PL179-GCST90277398	Triacylglycerol (52:4) levels	rs13259370	8	59,090,254	A	G	0.1975	0.0988	0.0208	2.1070E-06	7,174	22.530	TRUE
ebi-PL179-GCST90277399	Triacylglycerol (52:5) levels	rs113672231	19	33,135,815	C	G	0.0780	0.1421	0.0311	4.9501E-06	7,172	20.887	TRUE
ebi-PL179-GCST90277399	Triacylglycerol (52:5) levels	rs142146849	10	101,966,464	C	A	0.0548	0.1705	0.0362	2.4566E-06	7,172	22.235	TRUE
ebi-PL179-GCST90277399	Triacylglycerol (52:5) levels	rs72900657	11	45,826,596	A	G	0.0231	-0.2728	0.0576	2.2053E-06	7,172	22.443	TRUE
ebi-PL179-GCST90277400	Triacylglycerol (52:6) levels	rs56257715	7	71,530,474	A	G	0.0093	0.4284	0.0925	3.7039E-06	6,554	21.445	TRUE
ebi-PL179-GCST90277400	Triacylglycerol (52:6) levels	rs61905503	11	89,627,134	C	G	0.0498	-0.1897	0.0401	2.2250E-06	6,554	22.429	TRUE
ebi-PL179-GCST90277400	Triacylglycerol (52:6) levels	rs4508726	3	37,387,931	G	A	0.3646	-0.0871	0.0182	1.7651E-06	6,554	22.876	TRUE
ebi-PL179-GCST90277400	Triacylglycerol (52:6) levels	rs445925	19	44,912,383	A	G	0.0774	0.1576	0.0324	1.1485E-06	6,554	23.704	TRUE
ebi-PL179-GCST90277400	Triacylglycerol (52:6) levels	rs78178542	4	179,015,062	C	T	0.0196	-0.3131	0.0652	1.5926E-06	6,554	23.073	TRUE
ebi-PL179-GCST90277400	Triacylglycerol (52:6) levels	rs9591384	13	51,065,337	A	T	0.1236	-0.1285	0.0265	1.2451E-06	6,554	23.548	TRUE
ebi-PL179-GCST90277400	Triacylglycerol (52:6) levels	rs2546044	19	34,971,620	G	A	0.5326	-0.0798	0.0173	4.0962E-06	6,554	21.253	TRUE
ebi-PL179-GCST90277400	Triacylglycerol (52:6) levels	rs1017216	4	180,460,689	A	G	0.4593	0.0813	0.0173	2.7268E-06	6,554	22.036	TRUE
ebi-PL179-GCST90277400	Triacylglycerol (52:6) levels	rs78095833	1	237,378,917	T	C	0.0199	-0.3064	0.0644	2.0116E-06	6,554	22.623	TRUE
ebi-PL179-GCST90277401	Triacylglycerol (53:2) levels	rs75573052	13	21,038,010	T	C	0.0845	-0.1415	0.0309	4.7912E-06	6,996	20.951	TRUE
ebi-PL179-GCST90277401	Triacylglycerol (53:2) levels	rs72679249	4	110,201,006	G	A	0.2702	0.0918	0.0193	2.0568E-06	6,996	22.578	TRUE
ebi-PL179-GCST90277401	Triacylglycerol (53:2) levels	rs34021847	7	1,998,126	A	G	0.3201	-0.0884	0.0181	1.0265E-06	6,996	23.919	TRUE
ebi-PL179-GCST90277402	Triacylglycerol (53:3) levels	rs62291574	4	5,003,811	A	G	0.2145	0.0993	0.0211	2.6065E-06	7,113	22.121	TRUE
ebi-PL179-GCST90277402	Triacylglycerol (53:3) levels	rs12448223	16	4,104,550	C	T	0.4426	0.0774	0.0167	3.7706E-06	7,113	21.410	TRUE
ebi-PL179-GCST90277402	Triacylglycerol (53:3) levels	rs150741556	5	83,807,154	A	G	0.0262	0.2577	0.0525	9.2424E-07	7,113	24.125	TRUE
ebi-PL179-GCST90277402	Triacylglycerol (53:3) levels	rs11584070	1	150,322,476	G	A	0.1370	-0.1144	0.0244	2.8278E-06	7,113	21.963	TRUE
ebi-PL179-GCST90277402	Triacylglycerol (53:3) levels	rs1003966	13	21,038,020	T	C	0.0846	-0.1496	0.0304	9.1572E-07	7,113	24.139	TRUE
ebi-PL179-GCST90277402	Triacylglycerol (53:3) levels	rs12635725	3	1,895,465	G	A	0.5595	0.0807	0.0167	1.4641E-06	7,113	23.233	TRUE
ebi-PL179-GCST90277403	Triacylglycerol (53:4) levels	rs7846649	8	59,088,496	C	T	0.1906	0.1260	0.0223	1.5821E-08	6,479	32.028	TRUE
ebi-PL179-GCST90277403	Triacylglycerol (53:4) levels	rs71556711	7	73,440,219	T	C	0.0953	-0.1414	0.0300	2.4023E-06	6,479	22.281	TRUE
ebi-PL179-GCST90277403	Triacylglycerol (53:4) levels	rs35832030	17	1,624,088	T	G	0.1965	0.1038	0.0226	4.2984E-06	6,479	21.161	TRUE
ebi-PL179-GCST90277403	Triacylglycerol (53:4) levels	rs138987797	3	190,557,173	T	A	0.0034	0.7763	0.1588	1.0437E-06	6,479	23.889	TRUE
ebi-PL179-GCST90277403	Triacylglycerol (53:4) levels	rs2370511	12	78,389,910	C	T	0.1569	0.1165	0.0241	1.4415E-06	6,479	23.267	TRUE
ebi-PL179-GCST90277403	Triacylglycerol (53:4) levels	rs117922651	17	41,828,494	A	G	0.0571	-0.1769	0.0386	4.7913E-06	6,479	20.953	TRUE
ebi-PL179-GCST90277403	Triacylglycerol (53:4) levels	rs36174561	11	38,197,193	A	G	0.0870	-0.1489	0.0321	3.6373E-06	6,479	21.483	TRUE
ebi-PL179-GCST90277403	Triacylglycerol (53:4) levels	rs825692	16	73,556,815	A	G	0.1096	-0.1386	0.0289	1.6486E-06	6,479	23.007	TRUE
ebi-PL179-GCST90277403	Triacylglycerol (53:4) levels	rs1949011	2	141,741,698	A	C	0.4288	-0.0871	0.0179	1.1874E-06	6,479	23.641	TRUE
ebi-PL179-GCST90277403	Triacylglycerol (53:4) levels	rs79235240	3	42,816,309	A	C	0.0379	0.2181	0.0467	3.1350E-06	6,479	21.770	TRUE
ebi-PL179-GCST90277403	Triacylglycerol (53:4) levels	rs11570257	20	59,301,745	A	G	0.0195	0.3158	0.0684	3.9958E-06	6,479	21.302	TRUE
ebi-PL179-GCST90277404	Triacylglycerol (54:3) levels	rs2643912	1	2,352,403	T	G	0.4506	-0.0791	0.0168	2.6685E-06	7,162	22.076	TRUE
ebi-PL179-GCST90277404	Triacylglycerol (54:3) levels	rs3934642	4	81,683,582	G	C	0.1146	-0.1257	0.0267	2.5498E-06	7,162	22.163	TRUE
ebi-PL179-GCST90277404	Triacylglycerol (54:3) levels	rs2281721	1	230,161,390	T	C	0.5672	-0.0778	0.0169	4.4129E-06	7,162	21.107	TRUE
ebi-PL179-GCST90277404	Triacylglycerol (54:3) levels	rs2642442	1	220,800,221	T	C	0.6934	-0.1172	0.0182	1.4168E-10	7,162	41.260	TRUE
ebi-PL179-GCST90277404	Triacylglycerol (54:3) levels	rs17333900	10	88,675,523	T	G	0.1230	0.1182	0.0256	3.8374E-06	7,162	21.376	TRUE
ebi-PL179-GCST90277404	Triacylglycerol (54:3) levels	rs137871178	14	58,138,012	C	T	0.0173	0.3501	0.0660	1.1458E-07	7,162	28.166	TRUE
ebi-PL179-GCST90277404	Triacylglycerol (54:3) levels	rs3812316	7	73,606,007	G	C	0.1205	-0.1202	0.0255	2.4074E-06	7,162	22.273	TRUE
ebi-PL179-GCST90277404	Triacylglycerol (54:3) levels	rs144679576	8	2,896,369	C	T	0.0118	-0.3738	0.0802	3.1786E-06	7,162	21.740	TRUE
ebi-PL179-GCST90277405	Triacylglycerol (54:4) levels	rs73022136	6	165,581,086	T	C	0.0438	-0.1959	0.0429	4.9634E-06	7,165	20.883	TRUE
ebi-PL179-GCST90277405	Triacylglycerol (54:4) levels	rs2941755	2	79,833,869	C	T	0.9428	-0.1689	0.0367	4.2183E-06	7,165	21.194	TRUE
ebi-PL179-GCST90277405	Triacylglycerol (54:4) levels	rs115215688	19	33,148,516	G	C	0.0549	0.1682	0.0366	4.3050E-06	7,165	21.153	TRUE
ebi-PL179-GCST90277405	Triacylglycerol (54:4) levels	rs10779833	1	230,157,290	G	A	0.6568	-0.0933	0.0176	1.2141E-07	7,165	28.054	TRUE
ebi-PL179-GCST90277405	Triacylglycerol (54:4) levels	rs174604	11	61,858,798	G	C	0.3715	0.0983	0.0174	1.7060E-08	7,165	31.874	TRUE
ebi-PL179-GCST90277406	Triacylglycerol (54:5) levels	rs1456674	3	148,163,041	C	T	0.0088	-0.4506	0.0949	2.0958E-06	7,146	22.540	TRUE
ebi-PL179-GCST90277406	Triacylglycerol (54:5) levels	rs59017319	1	74,445,900	A	G	0.0469	0.1868	0.0406	4.1965E-06	7,146	21.204	TRUE

Lipid GWAS ID	Lipid species	SNP	Chromosome	Position	Effect allele	Other allele	Effect-allele frequency	Beta	SE	P value	Sample size	F statistic	F > 10
ebi-PL179-GCST90277406	Triacylglycerol (54:5) levels	rs13246993	7	73,608,416	A	G	0.1209	-0.1224	0.0255	1.5725E-06	7,146	23.095	TRUE
ebi-PL179-GCST90277406	Triacylglycerol (54:5) levels	rs74994260	19	33,147,680	G	C	0.0480	0.1906	0.0393	1.2784E-06	7,146	23.492	TRUE
ebi-PL179-GCST90277406	Triacylglycerol (54:5) levels	rs13178172	5	118,341,860	G	A	0.7389	0.0903	0.0188	1.5253E-06	7,146	23.154	TRUE
ebi-PL179-GCST90277407	Triacylglycerol (54:6) levels	rs114561185	2	204,066,660	G	A	0.0046	0.6169	0.1288	1.7046E-06	7,066	22.937	TRUE
ebi-PL179-GCST90277407	Triacylglycerol (54:6) levels	rs4057791	4	163,303,980	C	A	0.5462	0.0808	0.0169	1.7171E-06	7,066	22.925	TRUE
ebi-PL179-GCST90277408	Triacylglycerol (54:7) levels	rs76481684	1	217,318,646	G	A	0.0235	0.2650	0.0568	3.1846E-06	6,828	21.737	TRUE
ebi-PL179-GCST90277408	Triacylglycerol (54:7) levels	rs62534834	9	1,496,121	G	A	0.0605	-0.1671	0.0361	3.7178E-06	6,828	21.438	TRUE
ebi-PL179-GCST90277408	Triacylglycerol (54:7) levels	rs11927786	3	20,816,667	A	G	0.4112	-0.0831	0.0174	1.8952E-06	6,828	22.737	TRUE
ebi-PL179-GCST90277408	Triacylglycerol (54:7) levels	rs377037955	7	4,768,282	A	G	0.0031	0.7923	0.1716	3.9698E-06	6,828	21.314	TRUE
ebi-PL179-GCST90277408	Triacylglycerol (54:7) levels	rs2658623	10	57,917,923	T	C	0.7260	-0.0894	0.0192	3.5003E-06	6,828	21.554	TRUE
ebi-PL179-GCST90277408	Triacylglycerol (54:7) levels	rs111853669	1	230,959,661	A	G	0.0708	-0.1577	0.0335	2.5788E-06	6,828	22.142	TRUE
ebi-PL179-GCST90277408	Triacylglycerol (54:7) levels	rs417237	1	228,344,494	T	G	0.5896	0.0806	0.0172	3.0231E-06	6,828	21.837	TRUE
ebi-PL179-GCST90277408	Triacylglycerol (54:7) levels	rs12120498	1	246,000,079	C	T	0.6966	0.0939	0.0185	4.1029E-07	6,828	25.693	TRUE
ebi-PL179-GCST90277408	Triacylglycerol (54:7) levels	rs9645690	11	62,438,816	T	C	0.3216	-0.0851	0.0181	2.7387E-06	6,828	22.027	TRUE
ebi-PL179-GCST90277408	Triacylglycerol (54:7) levels	rs34942551	8	20,012,504	G	T	0.2532	-0.0916	0.0196	3.1077E-06	6,828	21.783	TRUE
ebi-PL179-GCST90277408	Triacylglycerol (54:7) levels	rs4970780	1	109,791,954	A	T	0.3269	0.0887	0.0191	3.4675E-06	6,828	21.573	TRUE
ebi-PL179-GCST90277408	Triacylglycerol (54:7) levels	rs56870907	14	104,290,084	C	G	0.1354	0.1219	0.0263	3.6124E-06	6,828	21.494	TRUE
ebi-PL179-GCST90277408	Triacylglycerol (54:7) levels	rs16996148	19	19,547,663	T	G	0.0658	-0.1608	0.0345	3.1630E-06	6,828	21.749	TRUE
ebi-PL179-GCST90277409	Triacylglycerol (56:3) levels	rs116846412	11	79,066,210	A	G	0.0322	-0.2442	0.0529	3.9219E-06	6,172	21.339	TRUE
ebi-PL179-GCST90277409	Triacylglycerol (56:3) levels	rs149199066	11	65,863,732	A	G	0.0444	0.2078	0.0444	2.9178E-06	6,172	21.909	TRUE
ebi-PL179-GCST90277409	Triacylglycerol (56:3) levels	rs181023072	16	62,982,329	A	C	0.0025	-0.8499	0.1775	1.7210E-06	6,172	22.926	TRUE
ebi-PL179-GCST90277409	Triacylglycerol (56:3) levels	rs4908327	1	99,527,521	T	C	0.8657	-0.1270	0.0267	2.0255E-06	6,172	22.612	TRUE
ebi-PL179-GCST90277409	Triacylglycerol (56:3) levels	rs11399	1	27,886,295	T	C	0.1004	0.1463	0.0301	1.1889E-06	6,172	23.640	TRUE
ebi-PL179-GCST90277409	Triacylglycerol (56:3) levels	rs117786044	11	128,757,016	A	G	0.0338	-0.2384	0.0509	2.8273E-06	6,172	21.970	TRUE
ebi-PL179-GCST90277409	Triacylglycerol (56:3) levels	rs9851701	3	103,282,265	A	G	0.1479	0.1190	0.0257	3.5835E-06	6,172	21.513	TRUE
ebi-PL179-GCST90277409	Triacylglycerol (56:3) levels	rs2980862	8	125,472,396	C	G	0.7433	-0.1014	0.0206	8.6751E-07	6,172	24.249	TRUE
ebi-PL179-GCST90277409	Triacylglycerol (56:3) levels	rs10058653	5	109,602,550	T	G	0.3017	-0.0888	0.0194	4.7743E-06	6,172	20.962	TRUE
ebi-PL179-GCST90277409	Triacylglycerol (56:3) levels	rs145737110	9	122,973,735	T	C	0.0143	0.3867	0.0845	4.7658E-06	6,172	20.966	TRUE
ebi-PL179-GCST90277410	Triacylglycerol (56:4) levels	rs10430989	11	43,542,682	A	G	0.2025	-0.1163	0.0234	6.6294E-07	5,820	24.773	TRUE
ebi-PL179-GCST90277410	Triacylglycerol (56:4) levels	rs1883350	22	43,932,163	C	T	0.3912	0.0989	0.0190	2.1208E-07	5,820	26.981	TRUE
ebi-PL179-GCST90277410	Triacylglycerol (56:4) levels	rs61942065	12	129,794,726	T	C	0.2164	0.1092	0.0224	1.1478E-06	5,820	23.713	TRUE
ebi-PL179-GCST90277410	Triacylglycerol (56:4) levels	rs10966037	9	23,714,477	T	A	0.2023	0.1094	0.0230	1.9359E-06	5,820	22.701	TRUE
ebi-PL179-GCST90277410	Triacylglycerol (56:4) levels	rs530715423	8	56,917,465	A	G	0.0517	0.2029	0.0419	1.3236E-06	5,820	23.436	TRUE
ebi-PL179-GCST90277411	Triacylglycerol (56:5) levels	rs182009884	1	182,723,688	C	G	0.0352	-0.2373	0.0519	4.9778E-06	5,490	20.884	TRUE
ebi-PL179-GCST90277411	Triacylglycerol (56:5) levels	rs78081886	4	27,694,435	A	G	0.0154	0.3584	0.0772	3.5167E-06	5,490	21.557	TRUE
ebi-PL179-GCST90277411	Triacylglycerol (56:5) levels	rs11623520	14	95,282,764	A	G	0.6501	-0.0921	0.0200	4.0201E-06	5,490	21.297	TRUE
ebi-PL179-GCST90277411	Triacylglycerol (56:5) levels	rs61960432	13	83,982,225	G	A	0.1148	-0.1506	0.0305	8.2161E-07	5,490	24.362	TRUE
ebi-PL179-GCST90277411	Triacylglycerol (56:5) levels	rs9682946	3	189,075,054	G	A	0.0882	0.1712	0.0342	5.6561E-07	5,490	25.083	TRUE
ebi-PL179-GCST90277411	Triacylglycerol (56:5) levels	rs73125564	4	32,591,008	T	A	0.2776	0.1050	0.0224	2.6808E-06	5,490	22.078	TRUE
ebi-PL179-GCST90277411	Triacylglycerol (56:5) levels	rs1441764	8	20,005,513	T	C	0.2805	-0.1332	0.0212	3.5974E-10	5,490	39.462	TRUE
ebi-PL179-GCST90277411	Triacylglycerol (56:5) levels	rs149473233	3	6,179,893	A	G	0.0052	0.6988	0.1486	2.6399E-06	5,490	22.100	TRUE
ebi-PL179-GCST90277411	Triacylglycerol (56:5) levels	rs9997979	4	99,524,202	T	C	0.0245	0.2851	0.0621	4.4406E-06	5,490	21.105	TRUE
ebi-PL179-GCST90277411	Triacylglycerol (56:5) levels	rs738409	22	43,928,847	G	C	0.2239	0.1451	0.0227	1.6453E-10	5,490	41.000	TRUE
ebi-PL179-GCST90277412	Triacylglycerol (56:6) levels	rs56013388	10	74,276,153	T	C	0.0103	-0.3792	0.0827	4.5897E-06	7,151	21.033	TRUE
ebi-PL179-GCST90277412	Triacylglycerol (56:6) levels	rs187665388	3	176,326,805	T	C	0.0274	-0.2478	0.0520	1.9325E-06	7,151	22.697	TRUE
ebi-PL179-GCST90277412	Triacylglycerol (56:6) levels	rs9578336	13	20,972,374	G	C	0.1823	0.1000	0.0217	4.0670E-06	7,151	21.265	TRUE
ebi-PL179-GCST90277412	Triacylglycerol (56:6) levels	rs4277987	6	168,930,674	G	A	0.2408	-0.0953	0.0196	1.1351E-06	7,151	23.723	TRUE
ebi-PL179-GCST90277412	Triacylglycerol (56:6) levels	rs56296027	2	20,934,251	C	T	0.2853	-0.0879	0.0183	1.6028E-06	7,151	23.057	TRUE
ebi-PL179-GCST90277412	Triacylglycerol (56:6) levels	rs2954021	8	125,469,835	G	A	0.5384	-0.0804	0.0168	1.6553E-06	7,151	22.996	TRUE
ebi-PL179-GCST90277412	Triacylglycerol (56:6) levels	rs10739560	9	120,268,566	C	T	0.9624	-0.2191	0.0461	2.0693E-06	7,151	22.565	TRUE
ebi-PL179-GCST90277412	Triacylglycerol (56:6) levels	rs114971431	2	56,529,283	G	A	0.0127	0.3479	0.0758	4.4488E-06	7,151	21.093	TRUE
ebi-PL179-GCST90277412	Triacylglycerol (56:6) levels	rs7863337	9	71,634,286	T	C	0.0949	-0.1369	0.0295	3.6523E-06	7,151	21.472	TRUE
ebi-PL179-GCST90277412	Triacylglycerol (56:6) levels	rs197730	3	37,455,234	G	A	0.4025	-0.0866	0.0170	3.3755E-07	7,151	26.071	TRUE
ebi-PL179-GCST90277413	Triacylglycerol (56:7) levels	rs592586	1	109,650,768	G	A	0.5947	0.0804	0.0169	2.0969E-06	7,148	22.539	TRUE
ebi-PL179-GCST90277413	Triacylglycerol (56:7) levels	rs150578762	22	28,083,968	A	G	0.0943	0.1326	0.0288	4.1069E-06	7,148	21.246	TRUE
ebi-PL179-GCST90277413	Triacylglycerol (56:7) levels	rs112214356	3	196,197,114	C	T	0.0609	0.1638	0.0353	3.4597E-06	7,148	21.576	TRUE
ebi-PL179-GCST90277413	Triacylglycerol (56:7) levels	rs6904245	6	134,421,023	A	G	0.0806	0.1448	0.0315	4.2801E-06	7,148	21.167	TRUE
ebi-PL179-GCST90277413	Triacylglycerol (56:7) levels	rs6699175	1	72,049,213	T	C	0.1136	0.1239	0.0266	3.2713E-06	7,148	21.683	TRUE

Lipid GWAS ID	Lipid species	SNP	Chromosome	Position	Effect allele	Other allele	Effect-allele frequency	Beta	SE	P value	Sample size	F statistic	F > 10
ebi-PL179-GCST90277413	Triacylglycerol (56:7) levels	rs75402627	9	130,364,541	C	T	0.0746	0.1559	0.0325	1.5919E-06	7,148	23.071	TRUE
ebi-PL179-GCST90277413	Triacylglycerol (56:7) levels	rs3916027	8	19,967,357	A	G	0.2526	-0.1256	0.0192	6.2203E-11	7,148	42.877	TRUE
ebi-PL179-GCST90277413	Triacylglycerol (56:7) levels	rs72623355	13	55,008,165	C	T	0.0969	-0.1333	0.0284	2.6365E-06	7,148	22.098	TRUE
ebi-PL179-GCST90277413	Triacylglycerol (56:7) levels	rs62419698	6	105,463,186	G	C	0.0605	-0.1724	0.0373	3.8128E-06	7,148	21.389	TRUE
ebi-PL179-GCST90277413	Triacylglycerol (56:7) levels	rs57706029	19	21,770,359	T	C	0.6053	0.0793	0.0172	3.9266E-06	7,148	21.332	TRUE
ebi-PL179-GCST90277413	Triacylglycerol (56:7) levels	rs513272	12	129,053,394	C	T	0.5635	0.0793	0.0174	4.9706E-06	7,148	20.881	TRUE
ebi-PL179-GCST90277413	Triacylglycerol (56:7) levels	rs483082	19	44,912,921	T	G	0.2423	0.1054	0.0196	7.9587E-08	7,148	28.874	TRUE
ebi-PL179-GCST90277414	Triacylglycerol (56:8) levels	rs7621165	3	166,672,944	A	G	0.4499	-0.0814	0.0173	2.7464E-06	6,800	22.020	TRUE
ebi-PL179-GCST90277414	Triacylglycerol (56:8) levels	rs10480656	7	104,552,272	G	C	0.2656	0.0922	0.0192	1.6256E-06	6,800	23.032	TRUE
ebi-PL179-GCST90277414	Triacylglycerol (56:8) levels	rs61994001	14	78,906,415	A	T	0.0055	-0.6102	0.1204	4.1224E-07	6,800	25.686	TRUE
ebi-PL179-GCST90277414	Triacylglycerol (56:8) levels	rs8028182	15	75,426,328	T	G	0.1881	-0.1026	0.0221	3.4454E-06	6,800	21.586	TRUE
ebi-PL179-GCST90277414	Triacylglycerol (56:8) levels	rs2165558	8	19,998,749	G	T	0.2709	-0.1113	0.0192	6.7043E-09	6,800	33.702	TRUE
ebi-PL179-GCST90277414	Triacylglycerol (56:8) levels	rs11649869	17	49,540,307	G	A	0.1825	0.1052	0.0227	3.7505E-06	6,800	21.425	TRUE
ebi-PL179-GCST90277414	Triacylglycerol (56:8) levels	rs62258666	3	35,239,965	A	G	0.0068	0.4991	0.1078	3.6913E-06	6,800	21.453	TRUE
ebi-PL179-GCST90277414	Triacylglycerol (56:8) levels	rs61967369	13	73,092,472	C	A	0.0644	-0.1610	0.0348	3.7114E-06	6,800	21.443	TRUE
ebi-PL179-GCST90277414	Triacylglycerol (56:8) levels	rs117564891	15	82,134,529	G	A	0.0366	-0.2122	0.0464	4.8014E-06	6,800	20.947	TRUE
ebi-PL179-GCST90277414	Triacylglycerol (56:8) levels	rs7031579	9	130,374,888	T	C	0.9195	-0.1467	0.0319	4.1758E-06	6,800	21.216	TRUE
ebi-PL179-GCST90277414	Triacylglycerol (56:8) levels	rs7815372	8	64,695,903	G	A	0.7916	0.0991	0.0211	2.6829E-06	6,800	22.068	TRUE
ebi-PL179-GCST90277415	Triacylglycerol (58:7) levels	rs1258712	12	31,313,616	T	G	0.9313	0.1799	0.0390	4.0775E-06	5,403	21.272	TRUE
ebi-PL179-GCST90277415	Triacylglycerol (58:7) levels	rs371927	20	44,634,395	A	G	0.9149	-0.1773	0.0368	1.5050E-06	5,403	23.191	TRUE
ebi-PL179-GCST90277415	Triacylglycerol (58:7) levels	rs3011337	10	120,774,281	G	A	0.6733	0.0991	0.0208	1.8507E-06	5,403	22.792	TRUE
ebi-PL179-GCST90277415	Triacylglycerol (58:7) levels	rs12700064	7	19,617,082	A	G	0.9002	0.1580	0.0333	2.1245E-06	5,403	22.526	TRUE
ebi-PL179-GCST90277415	Triacylglycerol (58:7) levels	rs141765432	14	76,516,128	T	G	0.0353	-0.2626	0.0532	8.3579E-07	5,403	24.329	TRUE
ebi-PL179-GCST90277416	Triacylglycerol (58:8) levels	rs4883863	13	70,301,753	T	C	0.4012	0.0854	0.0182	2.9027E-06	6,384	21.918	TRUE
ebi-PL179-GCST90277416	Triacylglycerol (58:8) levels	rs62321577	4	75,860,202	T	G	0.0502	-0.2061	0.0403	3.1251E-07	6,384	26.224	TRUE
ebi-PL179-GCST90277416	Triacylglycerol (58:8) levels	rs112792028	9	19,072,292	A	C	0.3216	-0.0928	0.0192	1.3431E-06	6,384	23.403	TRUE
ebi-PL179-GCST90277416	Triacylglycerol (58:8) levels	rs506912	1	109,649,355	A	C	0.7047	0.0929	0.0193	1.4574E-06	6,384	23.245	TRUE
ebi-PL179-GCST90277416	Triacylglycerol (58:8) levels	rs67872791	3	65,548,155	G	T	0.0070	0.5050	0.1070	2.4026E-06	6,384	22.281	TRUE
ebi-PL179-GCST90277416	Triacylglycerol (58:8) levels	rs11880266	19	55,460,024	G	A	0.3930	-0.0891	0.0187	2.0045E-06	6,384	22.631	TRUE
ebi-PL179-GCST90277416	Triacylglycerol (58:8) levels	rs116095614	3	2,139,069	A	G	0.0074	0.4950	0.1070	3.7779E-06	6,384	21.409	TRUE
ebi-PL179-GCST90277416	Triacylglycerol (58:8) levels	rs4726178	7	152,575,262	T	C	0.2011	-0.1065	0.0221	1.5353E-06	6,384	23.144	TRUE

Note: The table contains 1,887 retained instrument records. F statistics were calculated as beta squared divided by SE squared, and all retained instruments had F > 10. Abbreviations: F, F statistic; GWAS, genome-wide association study; SE, standard error; SNP, single-nucleotide polymorphism.
