# Supplementary material for: Genetically proxied circulating PD-1/PD-L1 levels and broadly defined myocarditis: A bidirectional Mendelian randomization study with exploratory lipidomic analyses
Source: Medicine (Baltimore). 2026 Aug 28;105(35):e50475. doi: 10.1097/MD.0000000000050475 (PMC13529170; doi:10.1097/MD.0000000000050475)
Supplement: Supplementary file 4 [file medi-105-e50475-s004.pdf]

Supplementary Table 5. Conventional-threshold, multiplicity, heterogeneity, pleiotropy, influence, and MR-RAPS sensitivity analyses.

Analysis	Sensitivity method	No. of SNPs	Estimate / statistic	Degrees of freedom	95% CI lower	95% CI upper	P value	Result
Primary UKB-PPP PD-L1 → FinnGen R10	IVW Cochran Q	28	22.7520	27			0.6980	No statistical evidence of heterogeneity
Primary UKB-PPP PD-L1 → FinnGen R10	MR-Egger intercept	28	-0.0117				0.3490	SE = 0.0123; no statistical evidence of directional horizontal pleiotropy
Primary UKB-PPP PD-L1 → FinnGen R10	MR-PRESSO global test	28					0.7560	No outlier detected; outlier-corrected and distortion tests not applicable
Primary UKB-PPP PD-L1 → FinnGen R10	Radial IVW after excluding rs579459	27	0.8540		0.741	0.984	0.0290	Inverse direction retained
Primary UKB-PPP PD-L1 → FinnGen R10	MR-RAPS	28	0.8430		0.718	0.989	0.0366	Inverse estimate; similar magnitude to primary IVW
Strict $P < 5 \times 10^{-8}$ UKB-PPP PD-L1 → FinnGen R10	IVW	12	0.8740		0.722	1.057	0.1650	Inverse but less precise
Strict $P < 5 \times 10^{-8}$ UKB-PPP PD-L1 → FinnGen R10	IVW Cochran Q	12	10.1300	11			0.5190	No statistical evidence of heterogeneity
Strict $P < 5 \times 10^{-8}$ UKB-PPP PD-L1 → FinnGen R10	MR-Egger intercept	12	0.0029				0.8960	SE = 0.0215; no statistical evidence of directional horizontal pleiotropy
Primary UKB-PPP PD-L1 → FinnGen R10	Leave-one-out analysis	28						No single variant reversed the overall inverse direction; plots are provided in Supplementary Figure S2
Primary UKB-PPP PD-L1 → FinnGen R10	BH-FDR correction across the two prespecified proteins	28					0.0882	Secondary multiplicity assessment; not retained at BH-FDR < 0.05

Note: Nonsignificant diagnostic tests indicate no statistical evidence of the tested violation but do not prove its absence. Abbreviations: BH-FDR, Benjamini-Hochberg false discovery rate; CI, confidence interval; IVW, inverse-variance weighted; MR-PRESSO, Mendelian randomization pleiotropy residual sum and outlier; MR-RAPS, Mendelian randomization robust adjusted profile score; OR, odds ratio; PD-L1, programmed death-ligand 1; SE, standard error; SNP, single-nucleotide polymorphism; UKB-PPP, UK Biobank Pharma Proteomics Project.
