# Supplementary material for: Genetically proxied circulating PD-1/PD-L1 levels and broadly defined myocarditis: A bidirectional Mendelian randomization study with exploratory lipidomic analyses
Source: Medicine (Baltimore). 2026 Aug 28;105(35):e50475. doi: 10.1097/MD.0000000000050475 (PMC13529170; doi:10.1097/MD.0000000000050475)
Supplement: Supplementary file 5 [file medi-105-e50475-s005.pdf]

Supplementary Table 4. Complete forward and reverse protein Mendelian randomization results.

Direction	Exposure	Protein source	Outcome	Method	No. of SNPs	Beta	OR	95% CI lower	95% CI upper	P value	Interpretation
Protein → myocarditis	PD-L1	UKB-PPP	FinnGen Release 10 broadly defined myocarditis	Inverse variance weighted	28	-0.1820	0.834	0.698	0.995	0.0441	Primary estimate
Protein → myocarditis	PD-L1	UKB-PPP	FinnGen Release 10 broadly defined myocarditis	MR Egger	28	-0.0910	0.913	0.706	1.179	0.4912	Complementary estimator
Protein → myocarditis	PD-L1	UKB-PPP	FinnGen Release 10 broadly defined myocarditis	Weighted median	28	-0.1690	0.845	0.676	1.054	0.1361	Complementary estimator
Protein → myocarditis	PD-L1	UKB-PPP	FinnGen Release 10 broadly defined myocarditis	Weighted mode	28	-0.1850	0.831	0.664	1.040	0.1179	Complementary estimator
Protein → myocarditis	PD-1	UKB-PPP	FinnGen Release 10 broadly defined myocarditis	Inverse variance weighted	59	0.0790	1.082	0.905	1.293	0.3883	Primary estimate
Protein → myocarditis	PD-1	UKB-PPP	FinnGen Release 10 broadly defined myocarditis	MR Egger	59	0.0010	1.001	0.708	1.415	0.9947	Complementary estimator
Protein → myocarditis	PD-1	UKB-PPP	FinnGen Release 10 broadly defined myocarditis	Weighted median	59	0.1630	1.177	0.886	1.564	0.2598	Complementary estimator
Protein → myocarditis	PD-1	UKB-PPP	FinnGen Release 10 broadly defined myocarditis	Weighted mode	59	0.2000	1.221	0.879	1.697	0.2377	Complementary estimator
Protein → myocarditis	PD-L1	INTERVAL	FinnGen Release 10 broadly defined myocarditis	Inverse variance weighted	5	-0.4790	0.619	0.434	0.883	0.0083	Primary estimate
Protein → myocarditis	PD-L1	INTERVAL	FinnGen Release 10 broadly defined myocarditis	MR Egger	5	-0.7020	0.496	0.090	2.726	0.4786	Complementary estimator
Protein → myocarditis	PD-L1	INTERVAL	FinnGen Release 10 broadly defined myocarditis	Weighted median	5	-0.4830	0.617	0.399	0.953	0.0297	Complementary estimator
Protein → myocarditis	PD-L1	INTERVAL	FinnGen Release 10 broadly defined myocarditis	Weighted mode	5	-0.4450	0.641	0.373	1.100	0.1817	Complementary estimator
Protein → myocarditis	PD-1	INTERVAL	FinnGen Release 10 broadly defined myocarditis	Inverse variance weighted	13	0.1410	1.151	0.919	1.442	0.2212	Primary estimate
Protein → myocarditis	PD-1	INTERVAL	FinnGen Release 10 broadly defined myocarditis	MR Egger	13	0.2170	1.242	0.773	1.998	0.3896	Complementary estimator
Protein → myocarditis	PD-1	INTERVAL	FinnGen Release 10 broadly defined myocarditis	Weighted median	13	0.1350	1.145	0.845	1.553	0.3842	Complementary estimator
Protein → myocarditis	PD-1	INTERVAL	FinnGen Release 10 broadly defined myocarditis	Weighted mode	13	0.0360	1.037	0.698	1.540	0.8594	Complementary estimator
Myocarditis → protein	FinnGen Release 10 broadly defined myocarditis	UKB-PPP	PD-L1	Inverse variance weighted	8	0.0150		-0.016	0.046	0.3350	Reverse MR
Myocarditis → protein	FinnGen Release 10 broadly defined myocarditis	UKB-PPP	PD-1	Inverse variance weighted	9	0.0160		-0.010	0.043	0.2244	Reverse MR
Myocarditis → protein	FinnGen Release 10 broadly defined myocarditis	INTERVAL	PD-L1	Inverse variance weighted	8	0.0130		-0.085	0.112	0.7932	Reverse MR
Myocarditis → protein	FinnGen Release 10 broadly defined myocarditis	INTERVAL	PD-1	Inverse variance weighted	8	-0.0300		-0.096	0.036	0.3792	Reverse MR

Note: Results are presented for the UKB-PPP and INTERVAL protein sources in both causal directions. The BH-FDR multiplicity assessment is reported in Supplementary Table 5. Abbreviations: BH-FDR, Benjamini-Hochberg false discovery rate; CI, confidence interval; IVW, inverse-variance weighted; MR, Mendelian randomization; OR, odds ratio; PD-1, programmed cell death protein 1; PD-L1, programmed death-ligand 1; SNP, single-nucleotide polymorphism; UKB-PPP, UK Biobank Pharma Proteomics Project.
