# Supplementary material for: Genetically proxied circulating PD-1/PD-L1 levels and broadly defined myocarditis: A bidirectional Mendelian randomization study with exploratory lipidomic analyses
Source: Medicine (Baltimore). 2026 Aug 28;105(35):e50475. doi: 10.1097/MD.0000000000050475 (PMC13529170; doi:10.1097/MD.0000000000050475)
Supplement: Supplementary file 6 [file medi-105-e50475-s006.pdf]

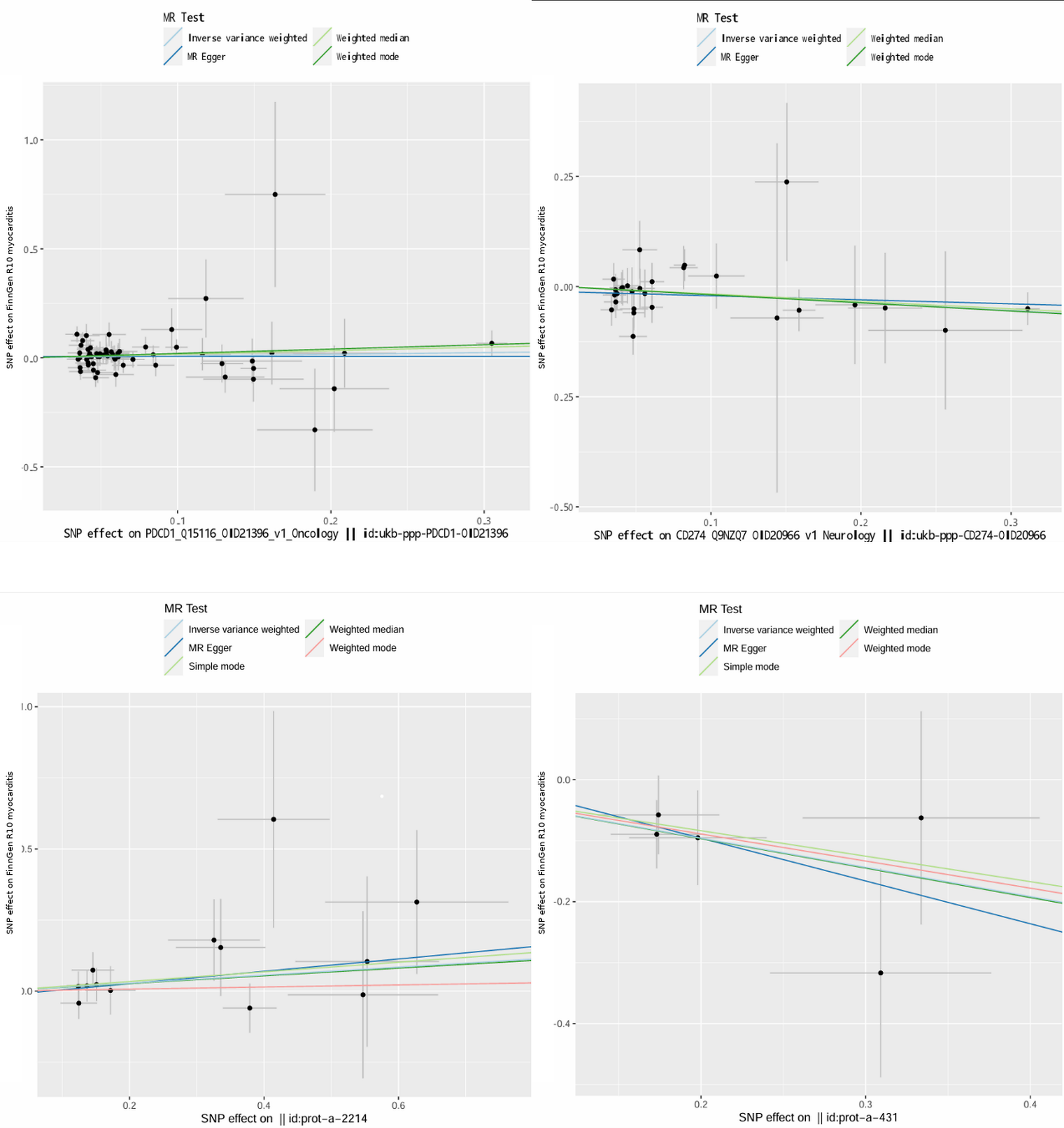
MR Test
Inverse variance weighted
MR Egger
Weighted median
Weighted mode
1.0
0.5
0.0
-0.5
0.1
0.2
0.3
SNP effect on FinnGen R10 myocarditis
SNP effect on PDCD1_Q15116_OID21396_v1_Oncology || id:ukb-ppp-PDCD1-OID21396
MR Test
Inverse variance weighted
MR Egger
Weighted median
Weighted mode
0.25
0.00
0.25
-0.50
0.1
0.2
0.3
SNP effect on FinnGen R10 myocarditis
SNP effect on CD274 Q9NZQ7 OID20966 v1 Neurology || id:ukb-ppp-CD274-OID20966
MR Test
Inverse variance weighted
MR Egger
Simple mode
Weighted median
Weighted mode
0.2
0.4
0.6
SNP effect on FinnGen R10 myocarditis
SNP effect on || id:prot-a-2214
MR Test
Inverse variance weighted
MR Egger
Simple mode
Weighted median
Weighted mode
0.0
-0.2
-0.4
0.2
0.3
0.4
SNP effect on FinnGen R10 myocarditis
SNP effect on || id:prot-a-431
