# Supplementary material for: Genetically proxied circulating PD-1/PD-L1 levels and broadly defined myocarditis: A bidirectional Mendelian randomization study with exploratory lipidomic analyses
Source: Medicine (Baltimore). 2026 Aug 28;105(35):e50475. doi: 10.1097/MD.0000000000050475 (PMC13529170; doi:10.1097/MD.0000000000050475)
Supplement: Supplementary file 7 [file medi-105-e50475-s007.pdf]

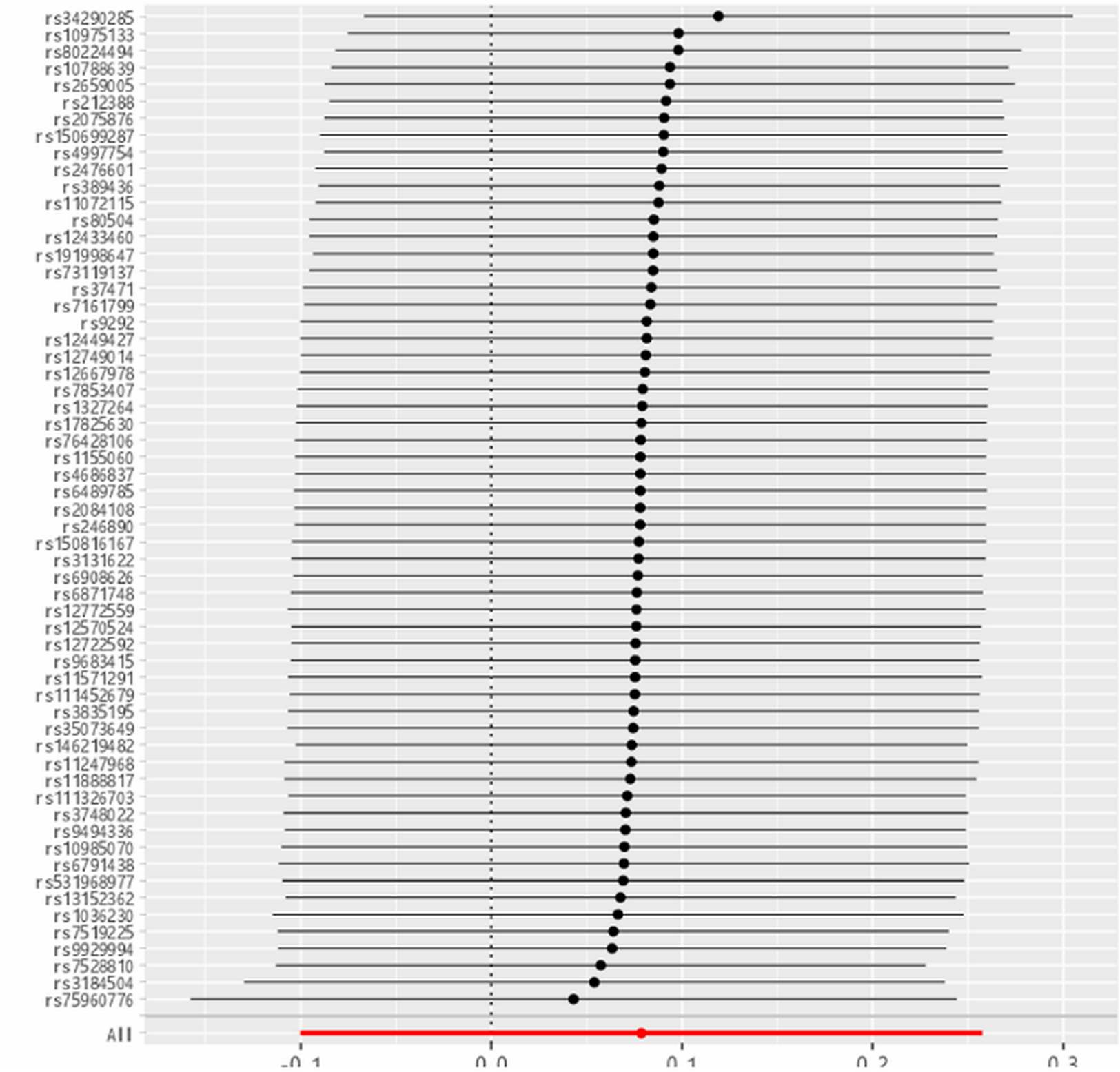

Leave-one-out: UKB-PPP PD-1 to FinnGen R10 myocarditis

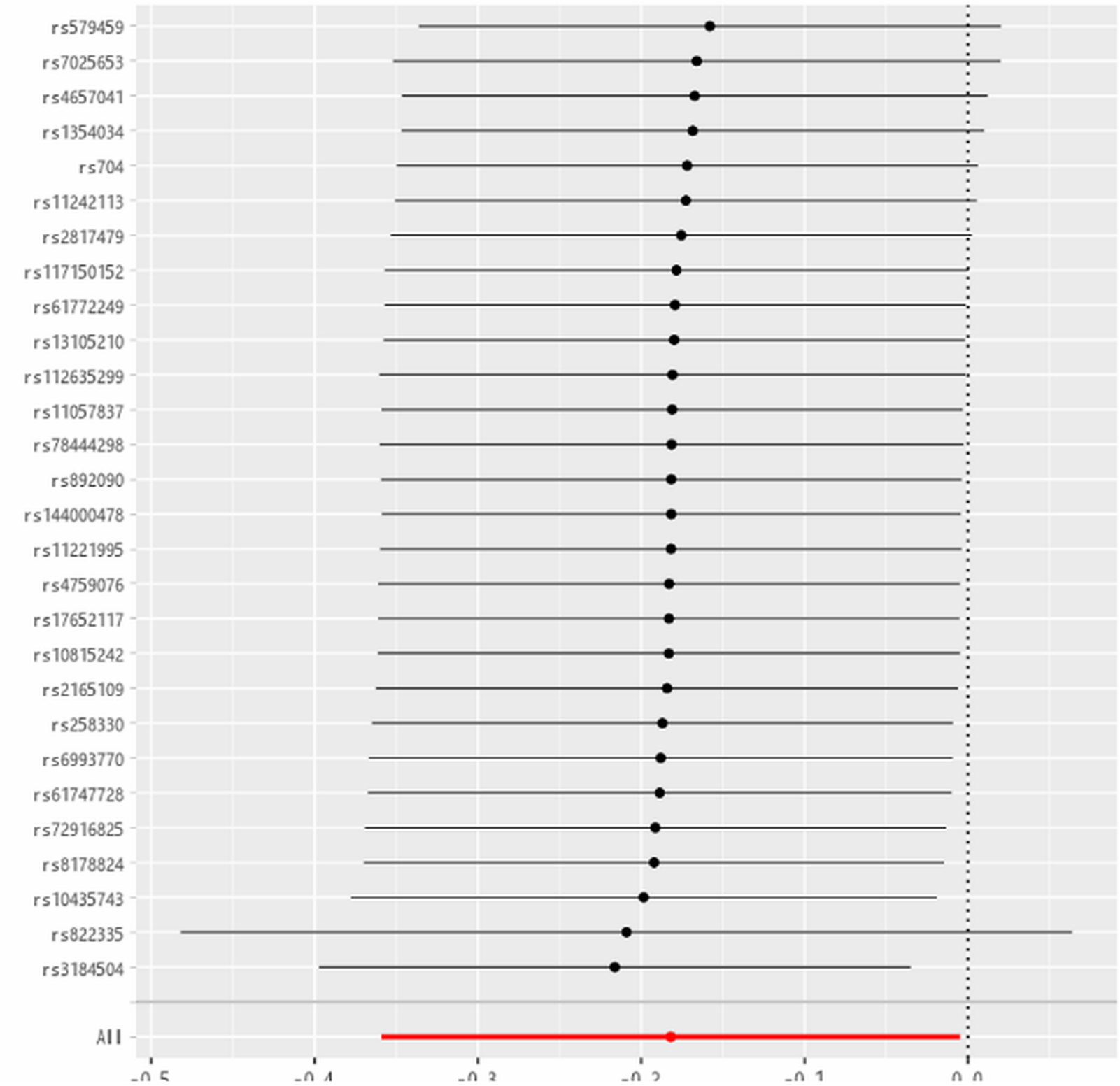

Leave-one-out: UKB-PPP PD-L1 to FinnGen R10 myocarditis

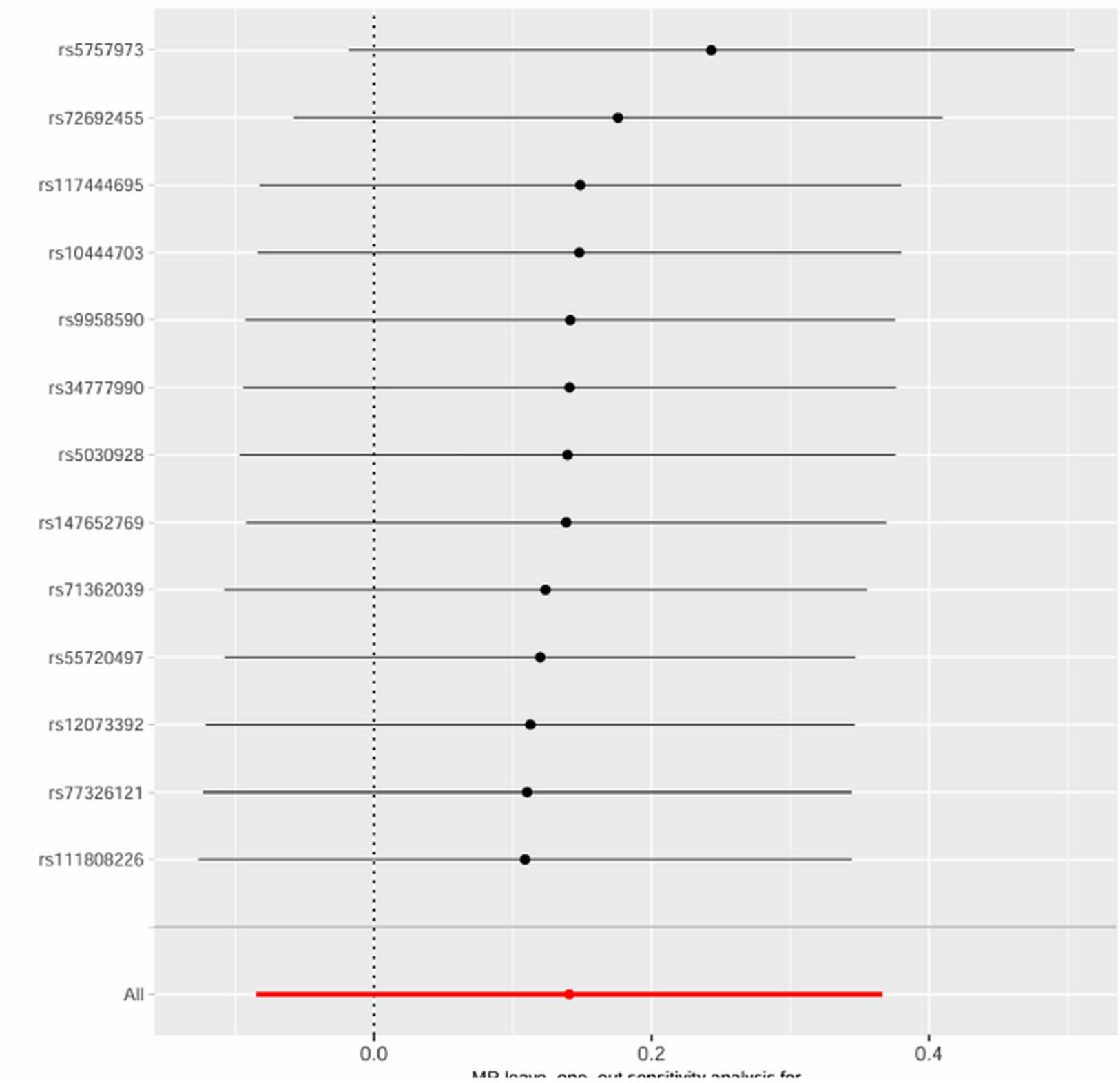

Leave-one-out: INTERVAL PD-1 to FinnGen R10 myocarditis

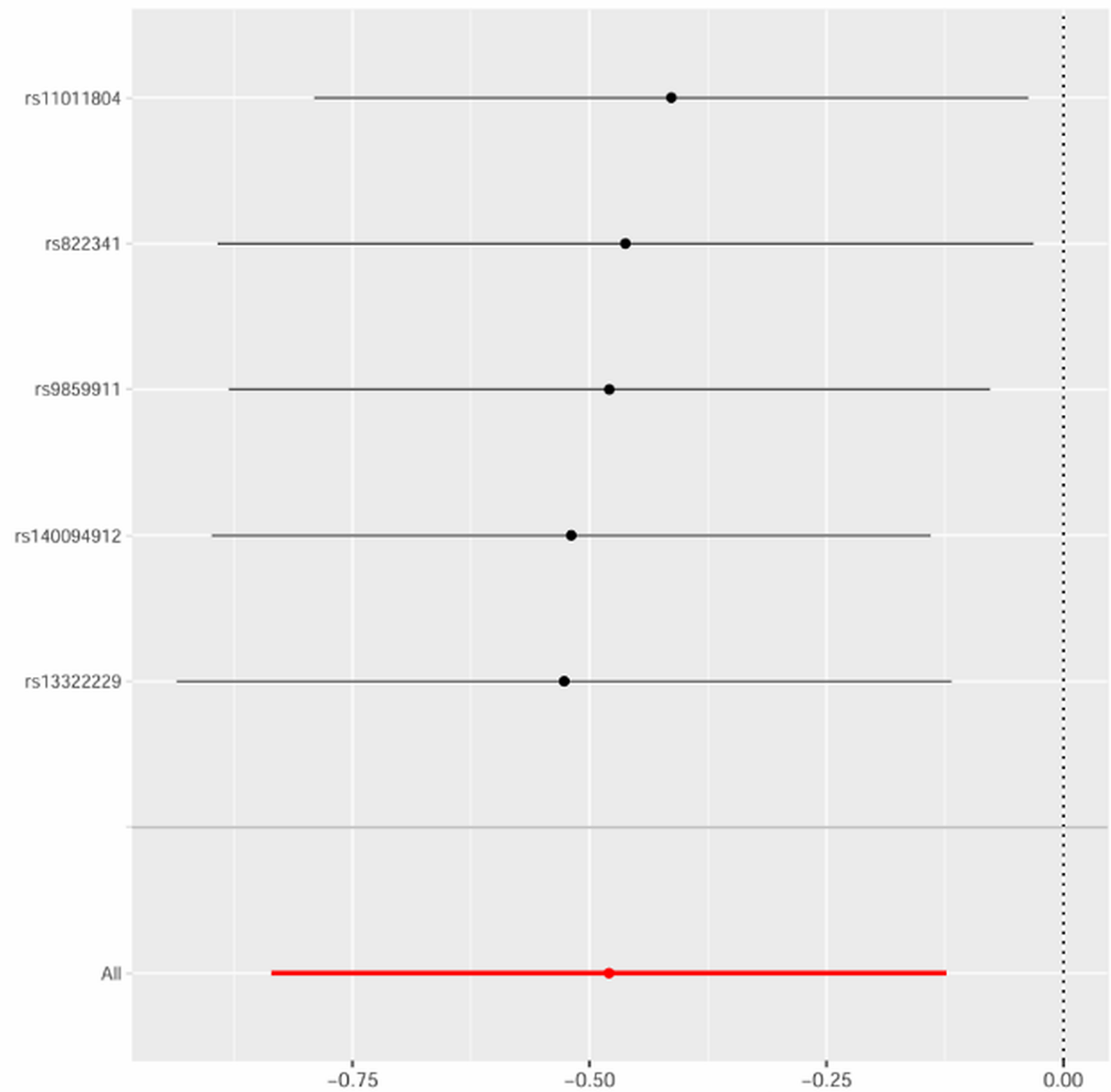

Leave-one-out: INTERVAL PD-L1 to FinnGen R10 myocarditis
