# Supplementary material for: Genetically proxied circulating PD-1/PD-L1 levels and broadly defined myocarditis: A bidirectional Mendelian randomization study with exploratory lipidomic analyses
Source: Medicine (Baltimore). 2026 Aug 28;105(35):e50475. doi: 10.1097/MD.0000000000050475 (PMC13529170; doi:10.1097/MD.0000000000050475)
Supplement: Supplementary file 8 [file medi-105-e50475-s008.pdf]

Supplementary Table 6. Independent myocarditis outcome validation and supportive PD-L1 meta-analysis.

Analysis family	Synthesis / analysis	Method	No. of SNPs or estimates	OR	95% CI lower	95% CI upper	P value	Result
Outcome validation	UKB-PPP PD-L1 → GCST90018882	IVW	18	0.781	0.642	0.951	0.0138	Inverse estimate
Outcome validation	INTERVAL PD-L1 → GCST90018882	Wald ratio	1	0.544	0.310	0.955	0.0340	Inverse estimate
Meta-analysis	UKB-PPP and INTERVAL PD-L1 estimates for FinnGen R10	Fixed-effect pooling	2	0.838	0.705	0.996	0.0450	Inverse pooled estimate
Meta-analysis sensitivity	UKB-PPP and INTERVAL PD-L1 estimates for FinnGen R10	Modified Hartung-Knapp	2	0.838	0.733	0.958	0.0380	mHK sensitivity estimate; interpret cautiously because only two estimates contributed
Correlation sensitivity	UKB-PPP and INTERVAL PD-L1 estimates for FinnGen R10	Variance-covariance model (ρ = 0.50)	2				0.0470	Inverse direction retained
Leave-one-estimate-out	Omit UKB-PPP estimate; retain INTERVAL estimate for FinnGen R10	Remaining INTERVAL estimate	1	0.619	0.434	0.883	0.0083	Inverse direction retained
Leave-one-estimate-out	Omit INTERVAL estimate; retain UKB-PPP estimate for FinnGen R10	Remaining UKB-PPP estimate	1	0.834	0.698	0.995	0.0441	Inverse direction retained
Meta-analysis	Both PD-L1 protein sources across FinnGen R10 and GCST90018882	Fixed-effect pooling	4	0.830	0.715	0.963	0.0140	Inverse pooled estimate
Meta-analysis sensitivity	Both PD-L1 protein sources across FinnGen R10 and GCST90018882	Modified Hartung-Knapp	4	0.797	0.651	0.975	0.0370	mHK sensitivity estimate; interpret cautiously because only four estimates contributed
Correlation sensitivity	Both PD-L1 protein sources across FinnGen R10 and GCST90018882	Variance-covariance model (ρ = 0.50)	4				0.0450	Inverse direction retained
Leave-one-estimate-out	Each contributing estimate omitted in turn across both protein and outcome sources	Fixed-effect pooling	3					Direction did not reverse in any omission analysis

Note: Fixed-effect and modified Hartung-Knapp estimates are reported separately. Cochran Q and I-squared did not indicate obvious heterogeneity, but the small number of estimates limited diagnostic power. Under rho = 0.50, P = 0.047 for the two-estimate synthesis and P = 0.045 for the four-estimate synthesis. Leave-one-estimate-out analysis did not reverse the pooled direction. Abbreviations: CI, confidence interval; IVW, inverse-variance weighted; mHK, modified Hartung-Knapp; OR, odds ratio; PD-L1, programmed death-ligand 1; SNP, single-nucleotide polymorphism; UKB-PPP, UK Biobank Pharma Proteomics Project.
