# Supplementary material for: Genetically proxied circulating PD-1/PD-L1 levels and broadly defined myocarditis: A bidirectional Mendelian randomization study with exploratory lipidomic analyses
Source: Medicine (Baltimore). 2026 Aug 28;105(35):e50475. doi: 10.1097/MD.0000000000050475 (PMC13529170; doi:10.1097/MD.0000000000050475)
Supplement: Supplementary file 9 [file medi-105-e50475-s009.pdf]

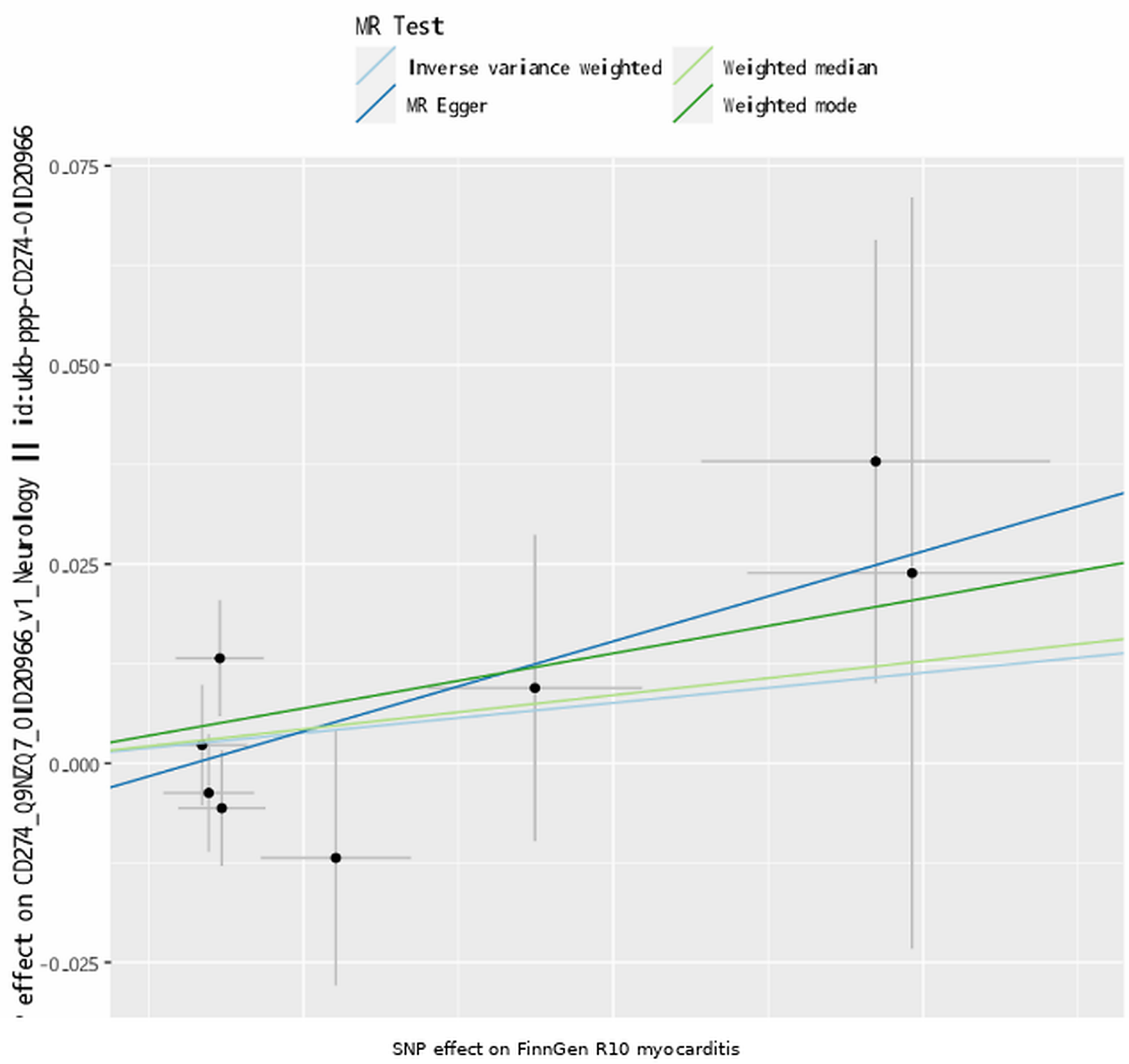
MR Test
Inverse variance weighted
MR Egger
Weighted median
Weighted mode
effect on CD274_Q9NZQ7_OID20966_v1_Neurology || id:ukb-ppp-CD274-OID20966
0.075
0.050
0.025
0.000
-0.025
SNP effect on FinnGen R10 myocarditis

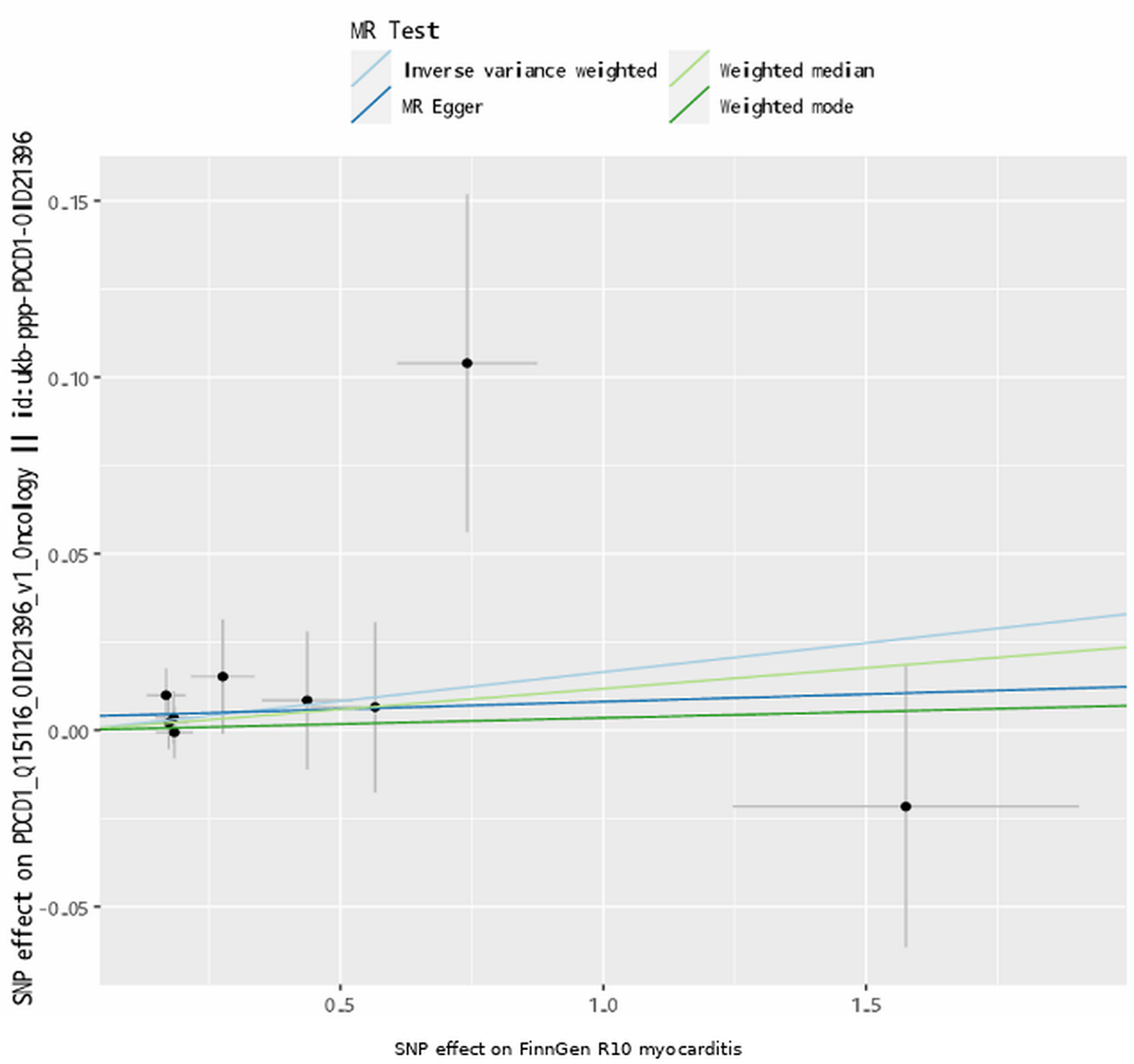
MR Test
Inverse variance weighted
MR Egger
Weighted median
Weighted mode
SNP effect on PDCD1_Q15116_OID21396_v1_Oncology || id:ukb-ppp-PDCD1-OID21396
0.15
0.10
0.05
0.00
-0.05
0.5
1.0
1.5
SNP effect on FinnGen R10 myocarditis

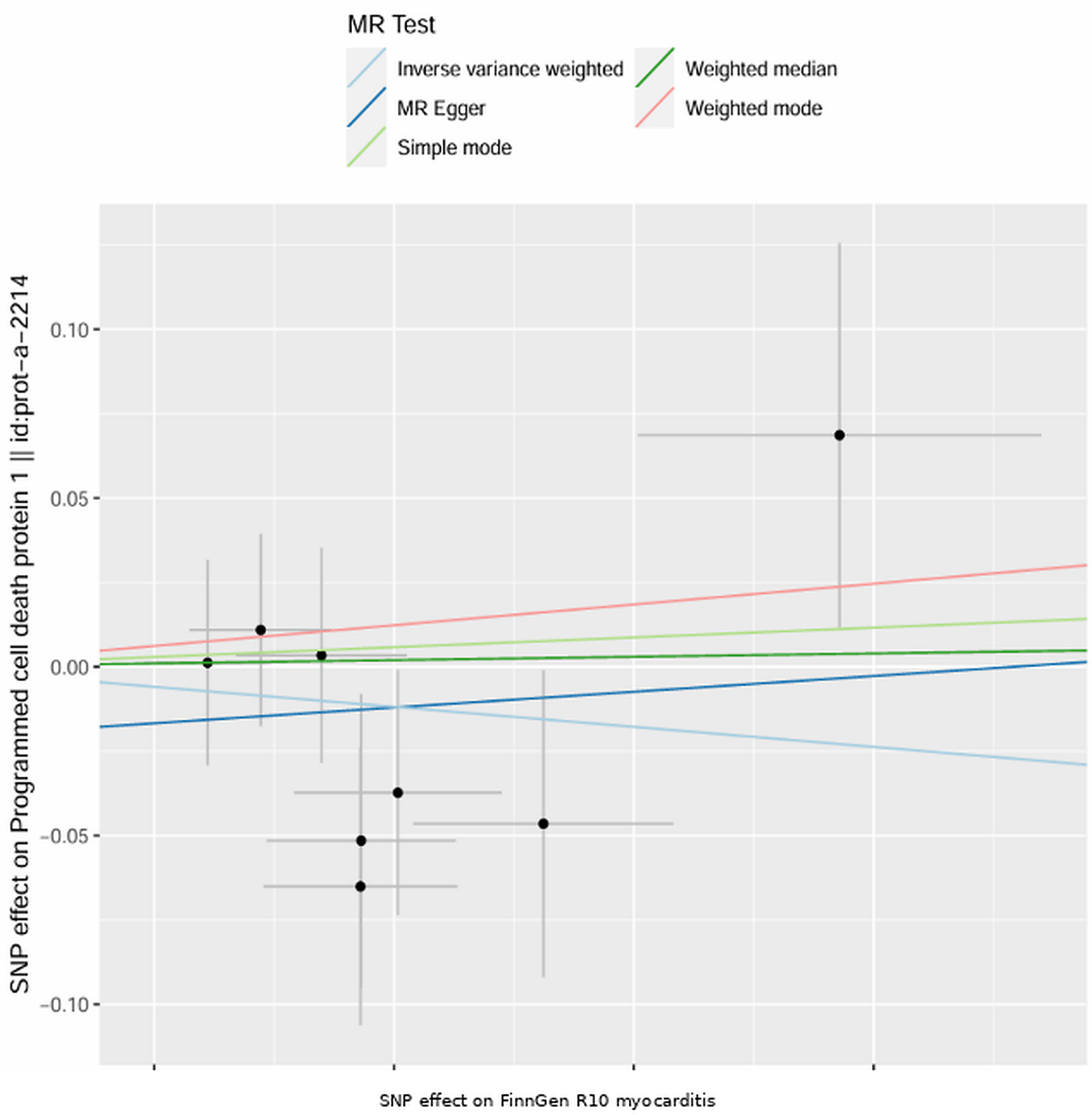
MR Test
Inverse variance weighted
MR Egger
Simple mode
Weighted median
Weighted mode
SNP effect on Programmed cell death protein 1 || id:prot-a-2214
0.10
0.05
0.00
-0.05
-0.10
SNP effect on FinnGen R10 myocarditis

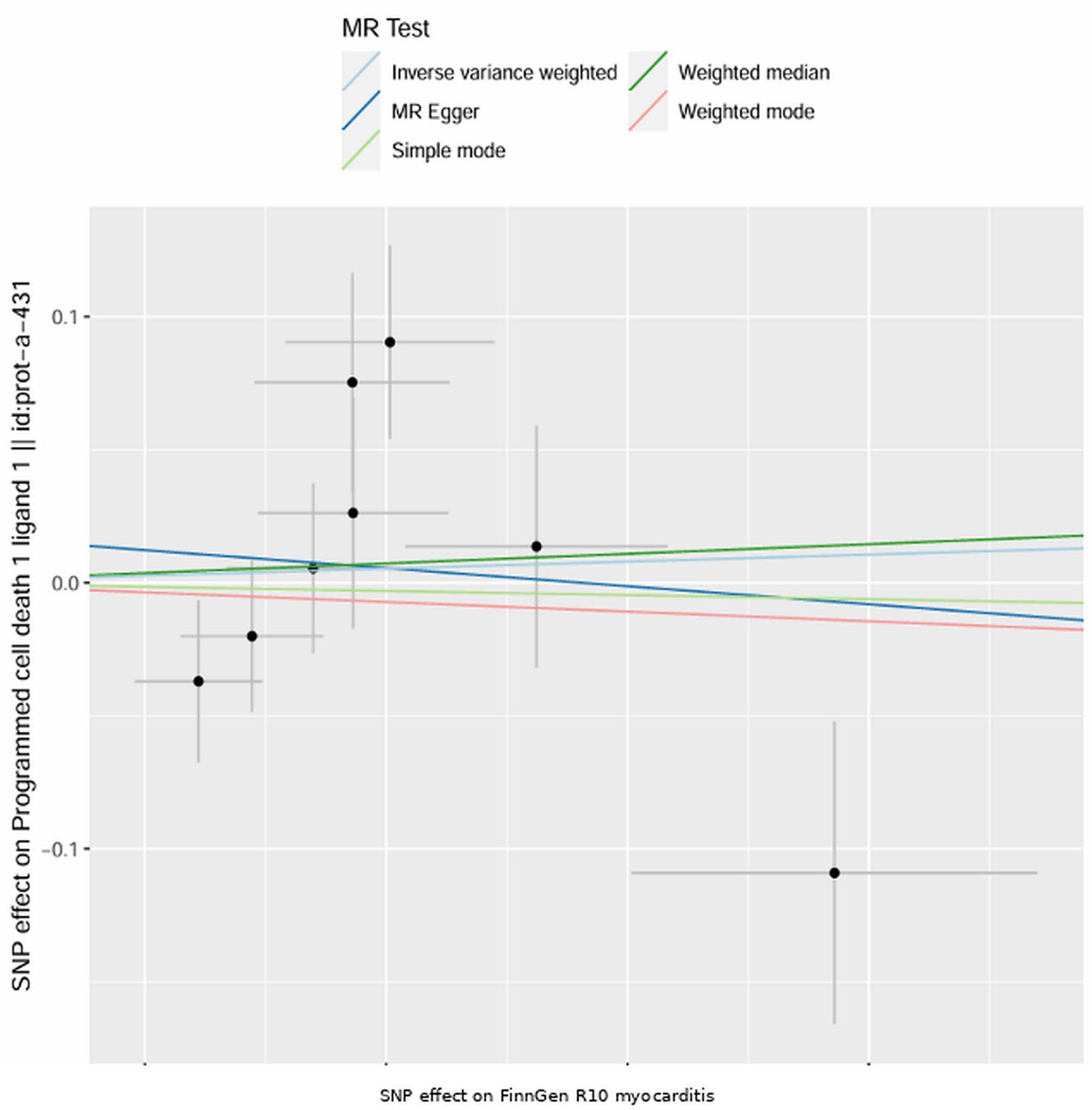
MR Test
Inverse variance weighted
MR Egger
Simple mode
Weighted median
Weighted mode
SNP effect on Programmed cell death 1 ligand 1 || id:prot-a-431
0.1
0.0
-0.1
SNP effect on FinnGen R10 myocarditis
