# Supplementary material for: Genetically proxied circulating PD-1/PD-L1 levels and broadly defined myocarditis: A bidirectional Mendelian randomization study with exploratory lipidomic analyses
Source: Medicine (Baltimore). 2026 Aug 28;105(35):e50475. doi: 10.1097/MD.0000000000050475 (PMC13529170; doi:10.1097/MD.0000000000050475)
Supplement: Supplementary file 10 [file medi-105-e50475-s010.pdf]

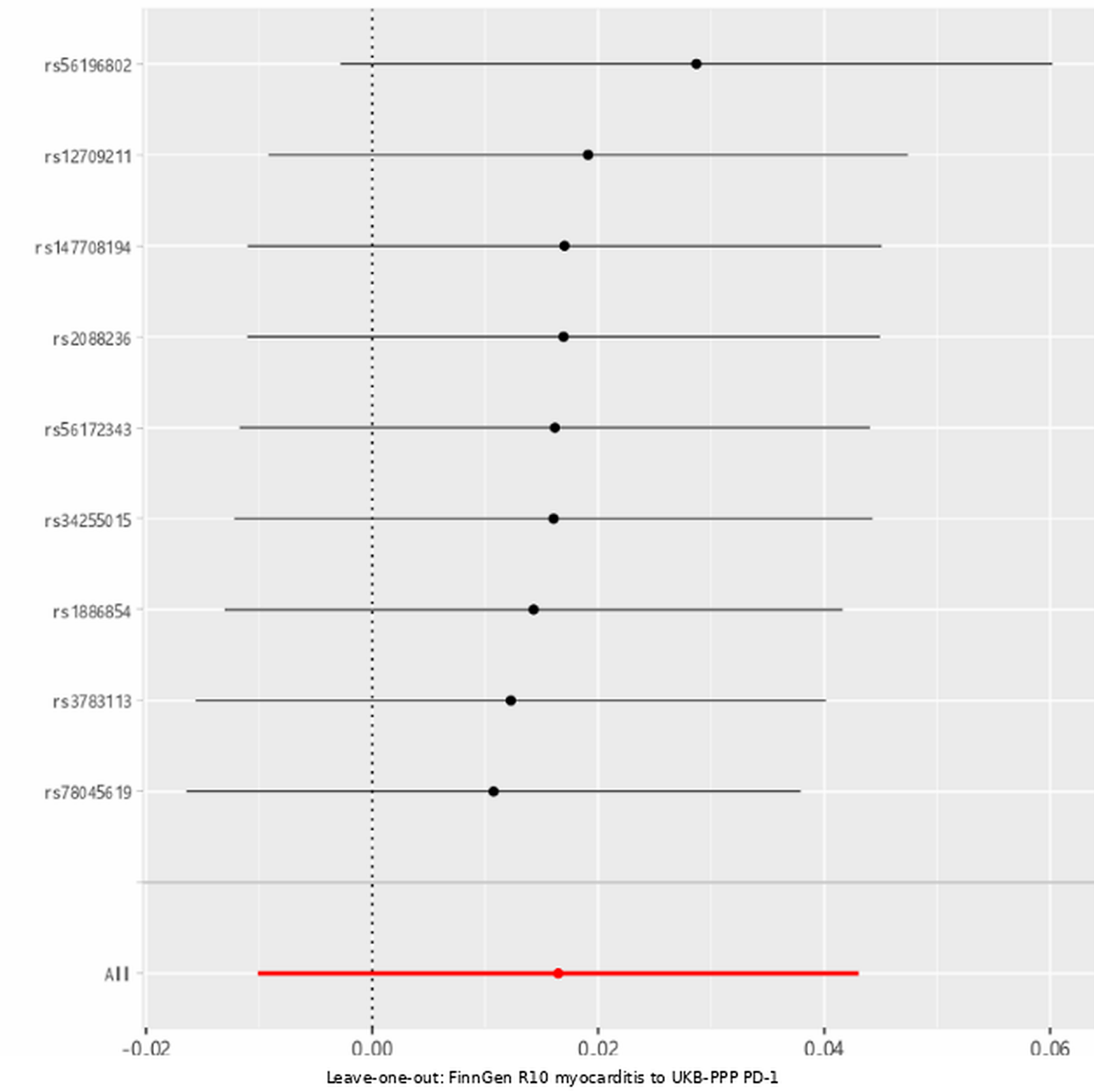
rs56196802
rs12709211
rs147708194
rs2088236
rs56172343
rs34255015
rs1886854
rs3783113
rs78045619
All
−0.02
0.00
0.02
0.04
0.06
Leave-one-out: FinnGen R10 myocarditis to UKB-PPP PD-1

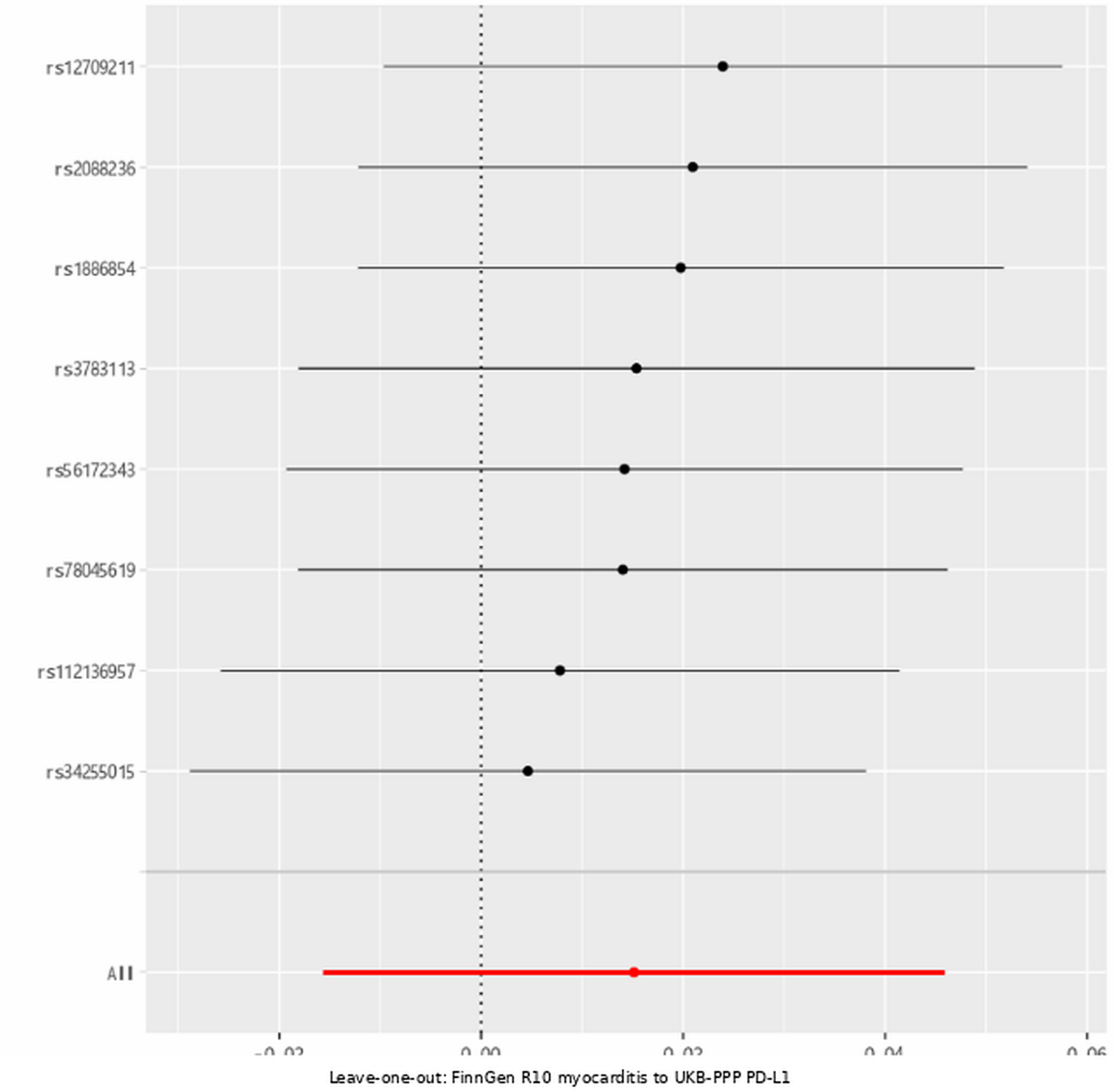
rs12709211
rs2088236
rs1886854
rs3783113
rs56172343
rs78045619
rs112136957
rs34255015
All
0.00
Leave-one-out: FinnGen R10 myocarditis to UKB-PPP PD-L1

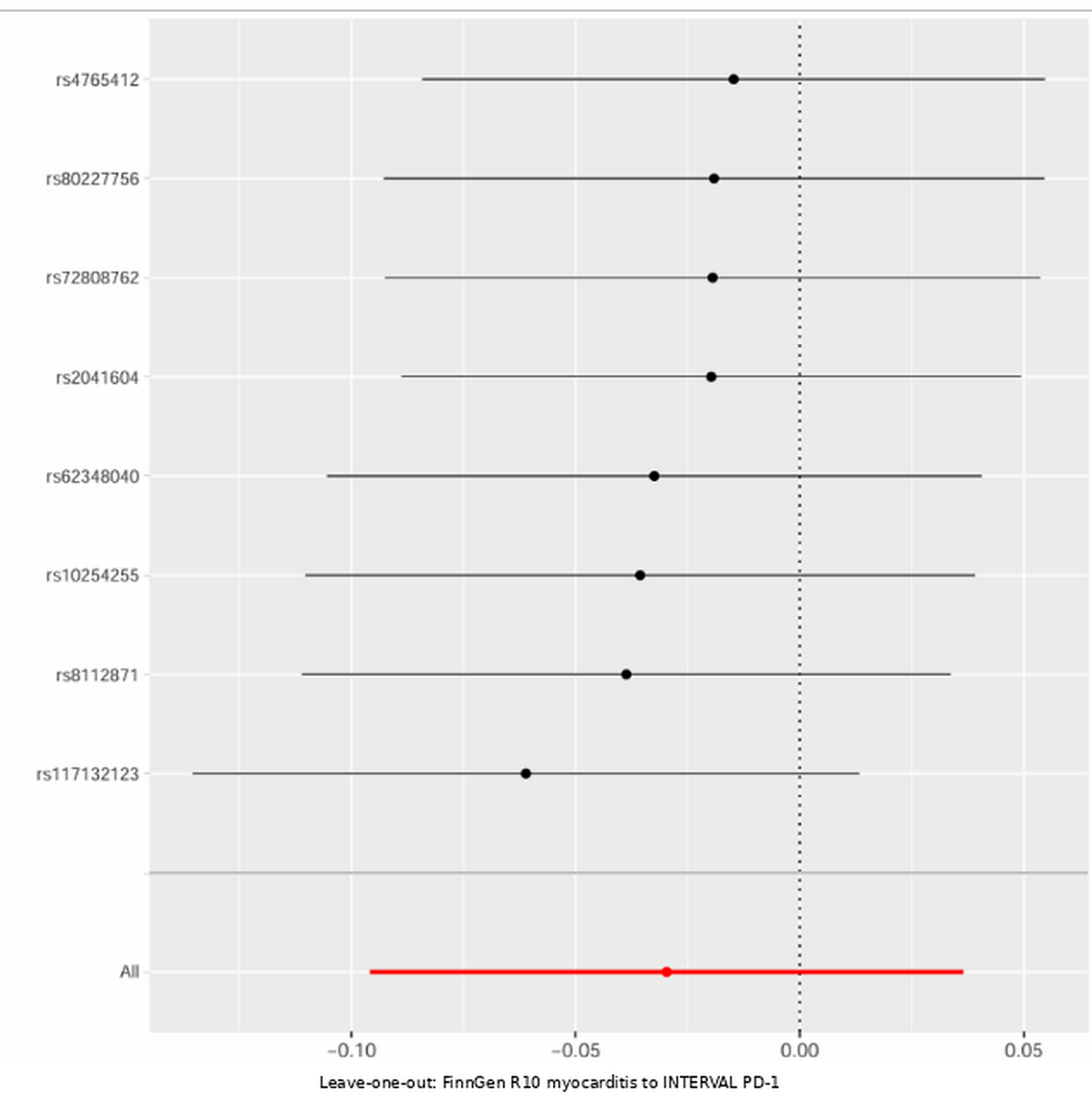
rs4765412
rs80227756
rs72808762
rs2041604
rs62348040
rs10254255
rs8112871
rs117132123
All
−0.10
−0.05
0.00
0.05
Leave-one-out: FinnGen R10 myocarditis to INTERVAL PD-1

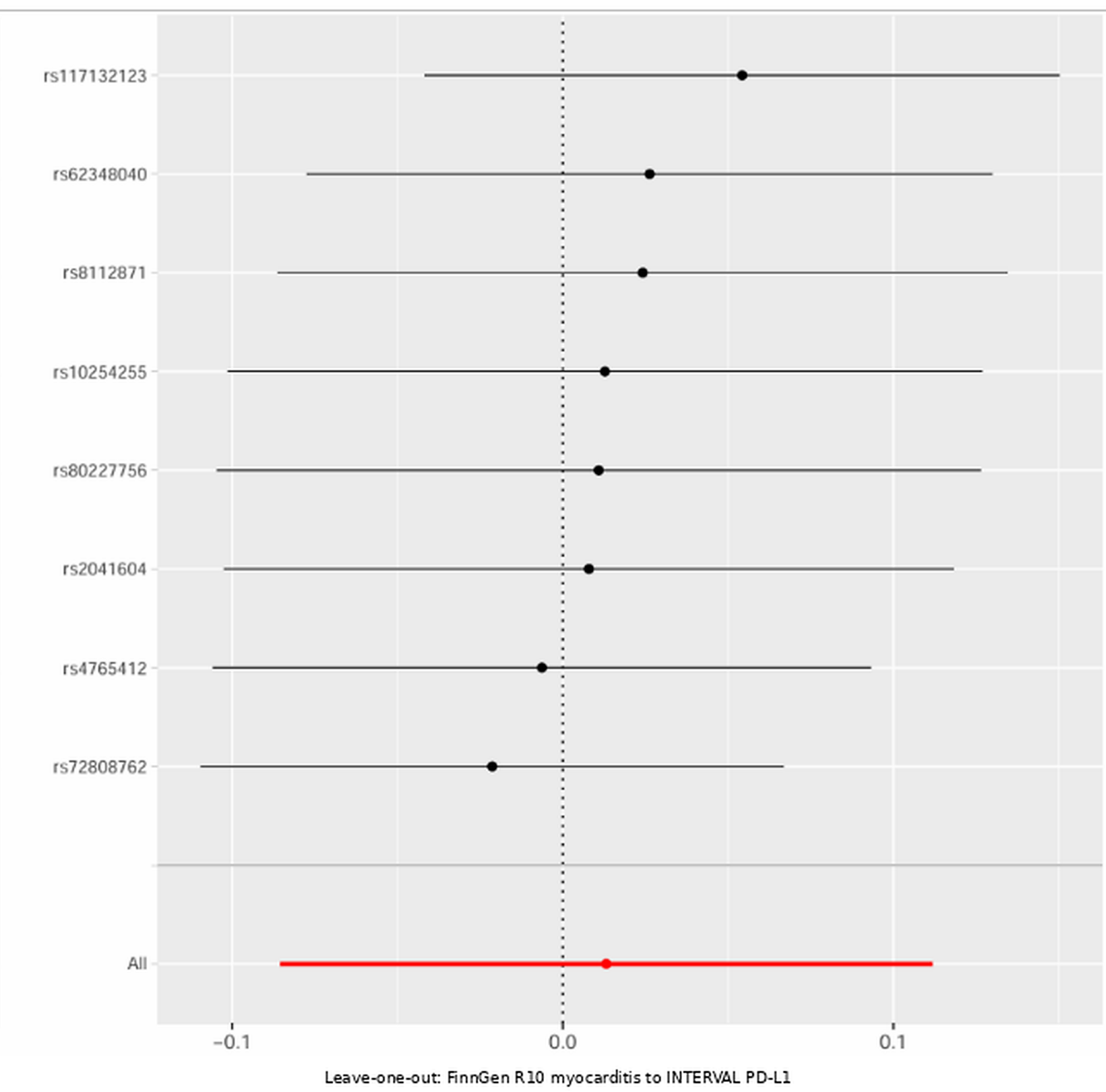
rs117132123
rs62348040
rs8112871
rs10254255
rs80227756
rs2041604
rs4765412
rs72808762
All
−0.1
0.0
0.1
Leave-one-out: FinnGen R10 myocarditis to INTERVAL PD-L1
