# Supplementary material for: Genetically proxied circulating PD-1/PD-L1 levels and broadly defined myocarditis: A bidirectional Mendelian randomization study with exploratory lipidomic analyses
Source: Medicine (Baltimore). 2026 Aug 28;105(35):e50475. doi: 10.1097/MD.0000000000050475 (PMC13529170; doi:10.1097/MD.0000000000050475)
Supplement: Supplementary file 11 [file medi-105-e50475-s011.pdf]

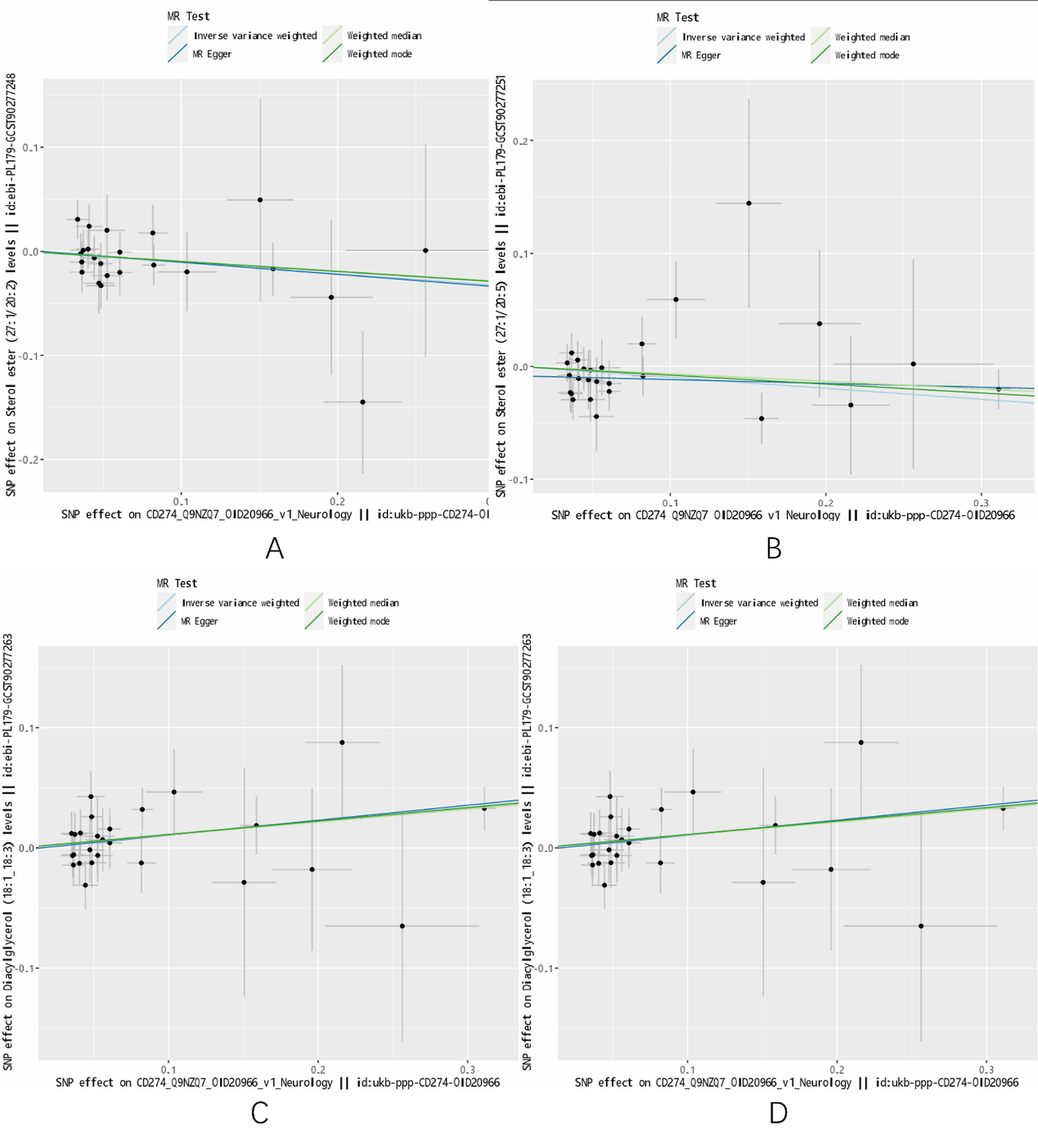
MR Test
Inverse variance weighted
MR Egger
Weighted median
Weighted mode
SNP effect on Sterol ester (27:1/20:2) levels || id:ebi-PL179-GCST90277248
SNP effect on CD274_Q9NZQ7_OID20966_v1_Neurology || id:ukb-ppp-CD274-OI
A
MR Test
Inverse variance weighted
MR Egger
Weighted median
Weighted mode
SNP effect on Sterol ester (27:1/20:5) levels || id:ebi-PL179-GCST90277251
SNP effect on CD274_Q9NZQ7_OID20966_v1_Neurology || id:ukb-ppp-CD274-OID20966
B
MR Test
Inverse variance weighted
MR Egger
Weighted median
Weighted mode
SNP effect on Diacylglycerol (18:1_18:3) levels || id:ebi-PL179-GCST90277263
SNP effect on CD274_Q9NZQ7_OID20966_v1_Neurology || id:ukb-ppp-CD274-OID20966
C
MR Test
Inverse variance weighted
MR Egger
Weighted median
Weighted mode
SNP effect on Diacylglycerol (18:1_18:3) levels || id:ebi-PL179-GCST90277263
SNP effect on CD274_Q9NZQ7_OID20966_v1_Neurology || id:ukb-ppp-CD274-OID20966
D
