# Supplementary material for: Genetically proxied circulating PD-1/PD-L1 levels and broadly defined myocarditis: A bidirectional Mendelian randomization study with exploratory lipidomic analyses
Source: Medicine (Baltimore). 2026 Aug 28;105(35):e50475. doi: 10.1097/MD.0000000000050475 (PMC13529170; doi:10.1097/MD.0000000000050475)
Supplement: Supplementary file 12 [file medi-105-e50475-s012.pdf]

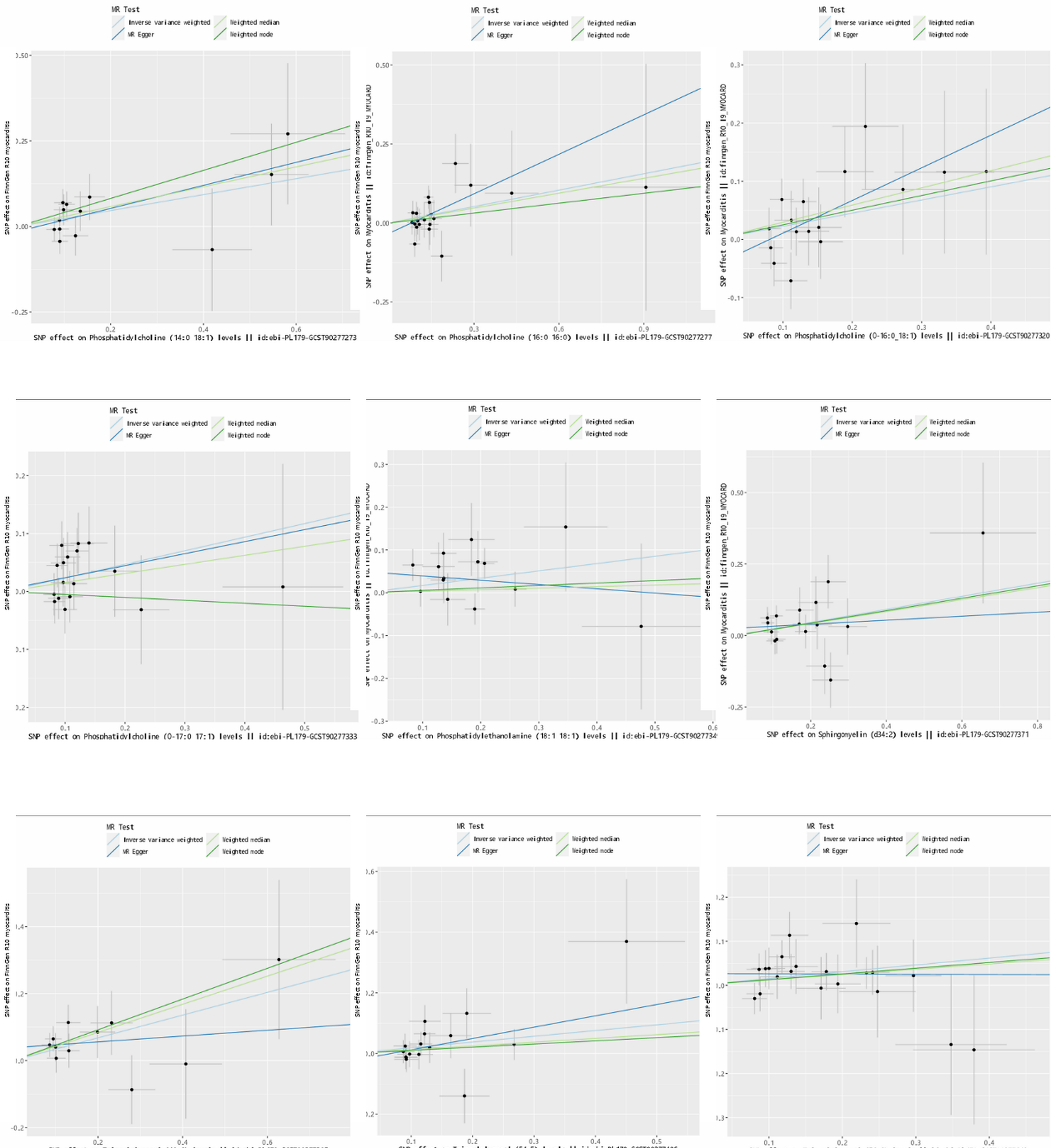

MR Test
Inverse variance weighted
MR Egger
Weighted median
Weighted mode
SNP effect on FinnGen R10 myocarditis
SNP effect on Phosphatidylcholine (14:0_18:1) levels || id:ebi-PL179-GCST90277273
MR Test
Inverse variance weighted
MR Egger
Weighted median
Weighted mode
SNP effect on Myocarditis || id:finngen_R10_I9_MYOCARD
SNP effect on Phosphatidylcholine (16:0_16:0) levels || id:ebi-PL179-GCST90277277
MR Test
Inverse variance weighted
MR Egger
Weighted median
Weighted mode
SNP effect on Myocarditis || id:finngen_R10_I9_MYOCARD
SNP effect on Phosphatidylcholine (O-16:0_18:1) levels || id:ebi-PL179-GCST90277320
MR Test
Inverse variance weighted
MR Egger
Weighted median
Weighted mode
SNP effect on FinnGen R10 myocarditis
SNP effect on Phosphatidylcholine (O-17:0_17:1) levels || id:ebi-PL179-GCST90277333
MR Test
Inverse variance weighted
MR Egger
Weighted median
Weighted mode
SNP effect on Phosphatidylethanolamine (18:1_18:1) levels || id:ebi-PL179-GCST9027734
MR Test
Inverse variance weighted
MR Egger
Weighted median
Weighted mode
SNP effect on Myocarditis || id:finngen_R10_I9_MYOCARD
SNP effect on Sphingomyelin (d34:2) levels || id:ebi-PL179-GCST90277371
MR Test
Inverse variance weighted
MR Egger
Weighted median
Weighted mode
SNP effect on FinnGen R10 myocarditis
SNP effect on Triacylglycerol (49:1) levels || id:ebi-PL179-GCST90277385
MR Test
Inverse variance weighted
MR Egger
Weighted median
Weighted mode
SNP effect on FinnGen R10 myocarditis
SNP effect on Triacylglycerol (54:5) levels || id:ebi-PL179-GCST90277406
MR Test
Inverse variance weighted
MR Egger
Weighted median
Weighted mode
SNP effect on FinnGen R10 myocarditis
SNP effect on Triacylglycerol (56:6) levels || id:ebi-PL179-GCST90277412
