# Supplementary material for: Genetically proxied circulating PD-1/PD-L1 levels and broadly defined myocarditis: A bidirectional Mendelian randomization study with exploratory lipidomic analyses
Source: Medicine (Baltimore). 2026 Aug 28;105(35):e50475. doi: 10.1097/MD.0000000000050475 (PMC13529170; doi:10.1097/MD.0000000000050475)
Supplement: Supplementary file 13 [file medi-105-e50475-s013.pdf]

Supplementary Table 7. Exploratory lipid Mendelian randomization associations and multiplicity interpretation.

Direction	Exposure	Outcome	Method	SNPs	Beta	95% CI	P value	Multiplicity interpretation
PD-L1 -> lipid	UKB-PPP PD-L1	Cholesteryl ester (27:1/20:2)	Inverse-variance weighted	24	-0.108	-0.203 to -0.012	0.0267	Not significant after BH-FDR/Bonferroni
PD-L1 -> lipid	UKB-PPP PD-L1	Cholesteryl ester (27:1/20:5)	Inverse-variance weighted	26	-0.097	-0.182 to -0.012	0.0246	Not significant after BH-FDR/Bonferroni
PD-L1 -> lipid	UKB-PPP PD-L1	Diacylglycerol (18:1_18:3)	Inverse-variance weighted	26	0.111	0.022 to 0.199	0.0143	Not significant after BH-FDR/Bonferroni
PD-L1 -> lipid	UKB-PPP PD-L1	Triacylglycerol (51:1)	Inverse-variance weighted	27	0.111	0.015 to 0.207	0.0237	Not significant after BH-FDR/Bonferroni
Lipid -> myocarditis	Phosphatidylcholine (14:0_18:1)	FinnGen Release 10 myocarditis	Inverse-variance weighted	13	0.233	0.017 to 0.449	0.0345	Not significant after BH-FDR/Bonferroni
Lipid -> myocarditis	Phosphatidylcholine (16:0_16:0)	FinnGen Release 10 myocarditis	Inverse-variance weighted	20	0.173	0.005 to 0.341	0.0435	Not significant after BH-FDR/Bonferroni
Lipid -> myocarditis	Phosphatidylcholine (O-16:0_18:1)	FinnGen Release 10 myocarditis	Inverse-variance weighted	16	0.226	0.027 to 0.424	0.0257	Not significant after BH-FDR/Bonferroni
Lipid -> myocarditis	Phosphatidylcholine (O-17:0_17:1)	FinnGen Release 10 myocarditis	Inverse-variance weighted	17	0.234	0.035 to 0.434	0.021	Not significant after BH-FDR/Bonferroni
Lipid -> myocarditis	Phosphatidylethanolamine (18:1_18:1)	FinnGen Release 10 myocarditis	Inverse-variance weighted	14	0.17	0.020 to 0.321	0.0268	Not significant after BH-FDR/Bonferroni
Lipid -> myocarditis	Sphingomyelin (d34:2)	FinnGen Release 10 myocarditis	Inverse-variance weighted	16	0.228	0.041 to 0.415	0.0166	Not significant after BH-FDR/Bonferroni
Lipid -> myocarditis	Triacylglycerol (49:1)	FinnGen Release 10 myocarditis	Inverse-variance weighted	11	0.339	0.104 to 0.574	0.0046	Not significant after BH-FDR/Bonferroni
Lipid -> myocarditis	Triacylglycerol (54:5)	FinnGen Release 10 myocarditis	Inverse-variance weighted	16	0.189	0.007 to 0.372	0.0422	Not significant after BH-FDR/Bonferroni
Lipid -> myocarditis	Triacylglycerol (56:6)	FinnGen Release 10 myocarditis	Inverse-variance weighted	20	0.154	0.013 to 0.296	0.032	Not significant after BH-FDR/Bonferroni

Note: The table includes four nominal PD-L1-to-lipid associations and nine nominal lipid-to-myocarditis associations. These analyses were exploratory across 179 lipid species in each direction, and none survived BH-FDR or Bonferroni correction. Abbreviations: BH-FDR, Benjamini-Hochberg false discovery rate; CI, confidence interval; IVW, inverse-variance weighted; MR, Mendelian randomization; SNP, single-nucleotide polymorphism.
